# RegA Plays a Key Role in Oxygen-Dependent Establishment of Persistence and in Isocitrate Lyase Activity, a Critical Determinant of *In vivo Brucella suis* Pathogenicity

**DOI:** 10.3389/fcimb.2017.00186

**Published:** 2017-05-18

**Authors:** Elias Abdou, María P. Jiménez de Bagüés, Ignacio Martínez-Abadía, Safia Ouahrani-Bettache, Véronique Pantesco, Alessandra Occhialini, Sascha Al Dahouk, Stephan Köhler, Véronique Jubier-Maurin

**Affiliations:** ^1^Institut de Recherche en Infectiologie de Montpellier UMR9004, Centre National de la Recherche Scientifique, Université de MontpellierMontpellier, France; ^2^Unidad de Tecnología en Producción y Sanidad Animal, Centro de Investigación y Tecnología Agroalimentaria, Instituto Agroalimentario de Aragón (CITA-Universidad de Zaragoza)Zaragoza, Spain; ^3^Institut de Médecine Régénératrice et Biothérapie—U1183 Institut National de la Santé et de la Recherche MédicaleMontpellier, France; ^4^Department of Biological Safety, German Federal Institute for Risk AssessmentBerlin, Germany

**Keywords:** *Brucella*, two-component system, RegA, oxygen, persistence, isocitrate lyase, infection, energy metabolism

## Abstract

For aerobic human pathogens, adaptation to hypoxia is a critical factor for the establishment of persistent infections, as oxygen availability is low inside the host. The two-component system RegB/A of *Brucella suis* plays a central role in the control of respiratory systems adapted to oxygen deficiency, and in persistence *in vivo*. Using an original “*in vitro* model of persistence” consisting in gradual oxygen depletion, we compared transcriptomes and proteomes of wild-type and Δ*regA* strains to identify the RegA-regulon potentially involved in the set-up of persistence. Consecutive to oxygen consumption resulting in growth arrest, 12% of the genes in *B. suis* were potentially controlled directly or indirectly by RegA, among which numerous transcriptional regulators were up-regulated. In contrast, genes or proteins involved in envelope biogenesis and in cellular division were repressed, suggesting a possible role for RegA in the set-up of a non-proliferative persistence state. Importantly, the greatest number of the RegA-repressed genes and proteins, including *aceA* encoding the functional IsoCitrate Lyase (ICL), were involved in energy production. A potential consequence of this RegA impact may be the slowing-down of the central metabolism as *B. suis* progressively enters into persistence. Moreover, ICL is an essential determinant of pathogenesis and long-term interactions with the host, as demonstrated by the strict dependence of *B. suis* on ICL activity for multiplication and persistence during *in vivo* infection. RegA regulates gene or protein expression of all functional groups, which is why RegA is a key regulator of *B. suis* in adaptation to oxygen depletion. This function may contribute to the constraint of bacterial growth, typical of chronic infection. Oxygen-dependent activation of two-component systems that control persistence regulons, shared by several aerobic human pathogens, has not been studied in *Brucella* sp. before. This work therefore contributes significantly to the unraveling of persistence mechanisms in this important zoonotic pathogen.

## Introduction

Oxygen deprivation has a major impact on the physiological state adopted by obligate aerobic human bacterial pathogens to maintain long-term interactions with their host organisms. Chronic infections represent a critical health problem since persistent bacteria are or become resistant to the clinically used antibiotics. This is exemplified by *Mycobacterium tuberculosis* and the opportunistic pathogen *Pseudomonas aeruginosa*, and has been shown more recently for *Brucella suis*, our model of interest. The intracellular pathogen *M. tuberculosis* induces its dormancy regulon under hypoxia, via the two-component system DosST/R (Boshoff and Barry, 2005; Honaker et al., 2009; Boon and Dick, 2012), to become able to persist *in vitro* (Voskuil et al., 2003), and evidences flexibility of its energy metabolism during mouse lung infection (Shi et al., 2005). *P. aeruginosa* shelters in nutrient- and oxygen-limited biofilms that allow the bacteria to persist in chronic infections. This bacterium modifies the transcriptional program of its metabolic pathways during the time course of infection in cystic fibrosis (CF) patients up to the stage of long-term persistence, to adapt to the physiological conditions of CF lung including denitrifying anaerobiosis (Palmer et al., 2007; Hoboth et al., 2009). Another characteristic of *P. aeruginosa* lung infections is the continuous recruitment of polymorphonuclear neutrophils (PMNs) (Jensen et al., 2007). The response regulator of the two-component system RoxS/R, functional homolog of the redox-responsive system RegB/A first discovered in *Rhodobacter* species, plays a crucial role in their trans-epithelial migration (Hurley et al., 2010). The regulon of this system may still be underestimated in *P. aeruginosa*, since it was characterized under aerobic conditions (Kawakami et al., 2010).

RegB/A has representatives in many alphaproteobacteria (Elsen et al., 2004; Wu and Bauer, 2008), among which *Brucella* spp. belong to the group of bacteria possessing a system with the highest similarity to that of *Rhodobacter capsulatus* and *Rhodobacter sphaeroides*.

*Brucella* is a facultative intracellular bacterium responsible for brucellosis, a zoonosis that affects livestock, causing abortion and sterility in animals, but also transmissible to humans provoking a debilitating febrile disease known as Malta fever. Brucellosis is considered as a major cause of economic loss and a re-emerging disease whose impact could increase since the pathogenic potential of the six recently described *Brucella* species for livestock and humans is yet unknown (Pappas, 2010; Scholz et al., 2016). *Brucella* possesses a type IV secretion system (T4SS) encoded by the *virB* operon (O'Callaghan et al., 1999) and responsible for the set-up of the replicative niche (Celli, 2006), characterized by nutritional paucity and low oxygen tension (Köhler et al., 2002). Without adequate treatment, *Brucella* can establish chronic infections with focal complications such as sacroiliitis, the most frequent osteoarticular involvement (Turan et al., 2011), endocarditis or neurobrucellosis (Ariza et al., 2001; Sohn et al., 2003). At the chronic stage, bacteria can be found in hypoxic organs within granulomas or abscesses where anoxic conditions predominate. Therefore, the capacity of this aerobic microorganism to adapt and persist under oxygen deficiency could be of major interest for novel therapeutic approaches. Indeed, our previous studies demonstrated that *Brucella suis*, one of the *Brucella* species most pathogenic for humans, expresses systems allowing this adaptation. On one hand, the oxidative respiration can use two high-oxygen-affinity terminal oxidases, the *cbb3*-type cytochrome *c* and the *bd* ubiquinol oxidases, on the other hand, the complete denitrification pathway enables utilization of nitrogen oxides as alternate electron acceptors in an anaerobic or microaerobic respiration process (Loisel-Meyer et al., 2005; Haine et al., 2006). Both respiratory systems are crucial for virulence and/or persistence *in vivo*. Lack of the *cbb3*-type oxidase or of its transcriptional activator FnrN (Loisel-Meyer et al., 2005), a potential direct oxygen sensor, caused strong attenuation of *B. suis* during the chronic phase of infection in oxygen-deficient organs of mice (Jiménez de Bagüés et al., 2007; Abdou et al., 2013). Replication of *B. suis* and *Brucella melitensis* strains devoid of nitric oxide reductase, or of its regulator NnrA in the latter, was altered within activated macrophages due to the defective detoxification of cellular NO, and in a murine model of *in vivo* infection (Haine et al., 2006; Loisel-Meyer et al., 2006). Moreover, activity of NtrYX as a redox sensor two-component system involved in oxygen sensing and in regulation of the denitrification enzymes was described in *Brucella abortus* (Carrica et al., 2012). Recently, we showed the central role of the two-component system RegB/A in the coordinated control of oxidative respiration and denitrification in *B. suis* (Abdou et al., 2013). Extensively studied, this redox sensing system also regulates the respiratory systems as well as many metabolic pathways of *Rhodobacter* species (Wu and Bauer, 2008). In response to hypoxic conditions, a signal is transmitted, via the redox state of the quinone pool and the cytosolic cysteine, to the sensor histidine kinase RegB. Redox sensor function of PrrB/A, the RegB/A ortholog in *B. abortus*, and signal transmission were comprehensively demonstrated (Carrica et al., 2013). In *B. suis* and *B. abortus*, identical RegB/A or PrrB/A regulate the expression of *nirK* encoding the nitrite reductase, the two operons *cyd* and *cco* encoding the *bd* and *cbb3* oxidases, respectively, and *fnrN* under different conditions of oxygenation (Abdou et al., 2013; Carrica et al., 2013). *B. suis* RegA is required for bacterial growth or survival under oxygen deficiency and, more importantly, is a critical determinant of pathogenesis, as a Δ*regA* strain is unable to trigger a chronic infection in mice (Abdou et al., 2013), suggesting a key role in the establishment of the state of persistence. The present study was undertaken to identify and characterize the RegA-dependent genes potentially involved in the set-up of the persistence state in *B. suis*. Because large-scale expression analyses are technically difficult to implement *in vivo*, particularly at the persistence phase when bacteremia is becoming extremely low (Abdou et al., 2013), we developed a novel experimental model in *Brucella* called “*in vitro* model of persistence.” This model allowed evaluation of the impact of *regA* inactivation during long-lasting *in vitro* persistence of *B. suis*. Transcriptomic and proteomic approaches were performed to compare wild-type and Δ*regA* mutant strains at the time point where anaerobic conditions become established. In *R. sphaeroides*, such analyses revealed that PrrA (RegA) regulates as a “master controller” directly or indirectly ~25% of the genes (Eraso et al., 2008). In the case of *B. suis*, we demonstrated that under low oxygen tension, the bacteria reduce their basal metabolism as growth decreases or stops and activate glycolysis and denitrification to maintain energy production (Al Dahouk et al., 2009). According to these results, we correctly predicted numerous systems under the control of RegA and potentially needed for persistence, which will contribute to give an insight into the mechanisms enabling *B. suis* to set up persistence. Assays were performed to biologically validate the impact of the response regulator on the activity of enzymes involved in energy production. As a significant candidate among other factors potentially essential for *in vitro B. suis* long-term survival, isocitrate lyase (ICL), the first enzyme of the glyoxylate shunt, was assessed by analysis of the corresponding mutant, using our model of persistence. The role of ICL in *B. suis*-host interaction was evaluated during mice infection.

## Materials and methods

### Bacterial strains and media

The *Brucella* reference strain used in this study, *B. suis* 1330 (ATCC 23444), and derived mutants were grown in Tryptic Soy (TS) at 37°C under BSL3 containment according to national laws and under control of the French National Agency for Drugs and Health Products Security (ANSM). Ultra-competent *Escherichia coli* DH5α (Invitrogen, Carlsbad, CA, U.S.A.) were used for cloning, and plasmid production after culture in Luria Bertani (LB) broth. For strains carrying a kanamycin resistance gene, 50 μg ml^−1^ antibiotic was used. The knock-out Δ*regA* mutant and its complemented strain were constructed previously (Abdou et al., 2013). For persistence assays, *B. suis* strains were adapted and grown in Gerhardt's minimal medium (GMM) (Gerhardt and Wilson, 1948), prepared as follows (per liter): glycerol (30 g), dipotassium phosphate (10 g), sodium chloride (7.5 g), lactic acid 85% (5.8 g), sodium thiosulfate (0.1 g), L-glutamic acid (1.5 g), and supplemented with vitaminic compounds [thiamine (0.2 mg), nicotinic acid (0.2 mg), pantothenic acid (0.04 mg), biotin (0.1 μg), MgSO_4_(H_2_O)_7_ (20 mg), MnSO_4_H_2_O (0.1 mg), FeSO_4_(H_2_O)_7_ (0.1 mg)].

### Construction of the *B. suis aceA* mutant and of its complemented strain

*aceA* (BR1614) mutant was constructed from an initial PCR product cloned into pUC18, comprising the complete coding sequence of *aceA* with 5′- and 3′-flanking sequences of 550 and 800 bp, respectively. Sequences specific to restriction enzymes *Sph*I and *Xba*I were included at the 5′-ends (underlined) of the forward (GCGGCATGCTCGCCGATTTCCACCAGTTCC) and reverse (GCTCTAGAAGCGAACTCAAACTGCGAACG) primers used, respectively. After introduction of the deletion (*Hinc*II+*Nae*I: 866 bp), the 1.2-kb blunted kanamycin resistance gene (*kan*^*r*^) was inserted, the recombinant plasmid was delivered to *B. suis*, and Δ*aceA B. suis* clones were selected and verified as previously described (Abdou et al., 2013). The Δ*aceA* strain of *B. suis* was complemented *in trans* with the native *aceA* gene produced from the initial pUC18-clone amplified with the initial forward primer (see above) and the Comp BR1614 reverse primer CGCGAGCTCGGTACTGGTCCTCCTGGTTC including the *Sac*I restriction site (underlined). The native PCR product was cloned as a 1,407 bp *Sma*I (85 bp upstream the intiation codon) + *Sac*I fragment into the replicative plasmid pBBR1MCS, under the control of the *lacZ* promoter.

### Bacterial adaptation to GMM

Fresh colonies of *B. suis* strains were inoculated in 10 ml of GMM at an initial optical density (OD) of 0.2 and grown at 37°C, with continuous agitation (160 RPM), for 8 days until cultures reached an OD ~0.8. Cultures were then diluted (1:4 or 1:5) and subcultured in 10 ml of GMM for 2 days to obtain an OD = 0.8. These subcultures were supplemented with 20% glycerol and stored at –80°C for further use.

### *In vitro* model of persistence

The model was adapted from the Wayne's model of *M. tuberculosis* persistence (Wayne and Sohaskey, 2001). The method consists in growing a bacterial suspension in airtight tubes (Sterilin, UK) containing GMM, respecting a head space ratio of 0.5, with slow stirring for continuous homogenization. Ten milliliters-precultures from the stored bacteria (see above) were grown in GMM to an OD_600_ = 0.7. Precultures were then harvested and diluted 1:30 in fresh GMM to reach a bacterial density of ~2 × 10^8^ CFU (colony forming units)/ml. Twenty milliliters of the dilution were distributed in tubes of 30 ml. Bacteria were incubated at 37°C with slow agitation (130 RPM) to ensure homogenization. An additional control tube with an anaerobic indicator strip was prepared to detect the establishment of anaerobic conditions. At each time point, the number of viable bacteria within the corresponding tube was determined by plating serial dilutions on TS agar, and bacteria were collected for RNA extraction.

### Isolation of RNA from *B. suis*

At each time point, bacterial samples were recovered after addition of 2 ml ethanol/phenol solution (9:1) to 18 ml of the cultures, to stop *de novo* RNA synthesis and to prevent its degradation. Purified RNAs were controlled quantitatively and qualitatively, and for absence of contaminating DNA in 0.5 μg samples as previously described (Abdou et al., 2013).

### Double stranded cDNA synthesis

Double-stranded (ds) cDNAs were prepared according to an adapted protocol of SuperScript Double-Stranded cDNA Synthesis Kit (Invitrogen, UK). Briefly, 10 μg of each denatured RNA sample were reverse-transcribed with random primers (252 pmoles), 0.5 mM dNTP and 400 units of SuperScript III reverse transcriptase, at 45°C overnight. The second strand was synthesized from the integral first-strand reaction according to the manufacturer's instructions. RNAse clean-up was performed with 4 μg of RNase A, at 37°C for 10 min. ds cDNAs were purified with the QIAquick PCR purification kit (Qiagen, USA), their concentration was measured with NanoDrop (170–240 ng/μl) and their quality was determined by using the Agilent 2100 bioanalyzer.

### Microarrays hybridations

Specific *B. suis* 1330 arrays ((A4325-00-01), Roche Nimblegen, France) consisted of glass slides on which 19 distinct 60-mer oligonucleotides specific to each of the 3 271 open reading frames of *B. suis* were spotted in triplicates. All the microarray experiments were performed at the “GeT-Biopuces” platform (Toulouse, France). By using the One Color DNA Labeling Kit (Roche NimbleGen) and according to the manufacturer's procedures, 1 μg of ds cDNA was labeled by random priming with 100 units of Klenow fragment and Cy3 random primers. After precipitation by isopropanol, 3 μg of Cy3-labeled cDNAs were added to the hybridization mix (Roche NimbleGen) for each microarray in the Hybridization System of Roche NimbleGen, during 17 h at 42°C. After washing with the Wash Buffer Kit (Roche NimbleGen), the slides were automatically scanned with the MS200 Microarray Scanner (TECAN, Männedorf, Switzerland). Images were analyzed with the NimbleScan V 2.6 software. A RMA (Robust Multi-array Average) analysis was performed to normalize background-subtracted raw data.

### Microarrays statistical analyses

Briefly, the two groups of three Δ*regA* (R) samples and three wild-type (W) samples data were compared by using Significance Analysis of Microarrays (SAM) tool (Tusher et al., 2001). A two-class unpaired supervised analysis was computed with SAM between the R group and the W group, applying the Wilcoxon statistic test and a setting of 300 permutations. SAM generated a list of significant genes with a fold change ≥ 2 and a false discovery rate (FDR) ≤ 5 (Figure S1).

### Data availability statement

The data discussed in this publication have been deposited in NCBI's Gene Expression Omnibus in the MIAME-compliant format and are accessible through GEO Series accession number GSE87538 (https://www.ncbi.nlm.nih.gov/geo/query/acc.cgi?acc=GSE87538).

### COG functional group analysis

The COG (cluster of orthologous groups) classification of CDS of the *B. suis* genome was based on the database of Comprehensive Microbial Resource (CMR) or on that of Genoscope found at http://www.genoscope.cns.fr/agc/microscope/mage/. All genes induced or repressed by RegA in the wild-type strain, having respectively a ratio WT/Δ*regA* mean value of ≥ 2-fold or ≤ 0.5-fold, were classified according to the functional category to which they belong.

### RT-qPCR analysis

Five hundred nanograms of the RNA samples used for microarray hybridizations were reverse-transcribed as reported in Abdou et al. (2013), and quantitative PCR experiments (RT-qPCR) were performed using the Light Cycler 480 with SYBR green chemistry (Roche). Primers (Sigma Genosys) were designed using Primer 3 Software (Table S5). Aliquots of cDNA samples were diluted 20- and 2,000-fold to quantify expression of the selected mRNAs and of the constitutively expressed 16S rRNA, respectively, the latter being used to normalize expression values. For the three biological replicates, normalized threshold cycles ΔCt from averaged three technical replicates were used for calculating the fold change using the ΔΔCt method (Table S3) and the relative fold change (WT/Δ*regA*) = 2^−ΔΔCt^. BR0756, characterized by constant expression in both strains, was also used as reference gene and provided the same results as those obtained with 16S rRNA gene for amplification of the target genes BR1614, BRA0703 and BRA0299. For comparison with the RT-qPCR results, microarray data were expressed as the mean log2 values of the hybridization ratio (WT/Δ*regA*) (Table S3).

### *In silico* search for RegA-binding sequences

We used the consensus sequence (C/T)-(G/C)-C-G-G-(C/G)-N_(0−10)_-G-(T/A)-C-(G/A)-(C/A) (Mao et al., 2005) of the DNA sites recognized by RegA/PrrA from *R. sphaeroides* and *R. capsulatus* to perform a manual search for potential RegA-binding sites upstream (1 kb upstream of the initiation codon) of 15 *B. suis* genes previously validated as being induced by RegA. Only motifs having at least one half-site 100% homologous to that of *R*. *sphaeroides* were taken into account. 44 potential RegA binding motifs were detected and used to generate 11 Positional Weight Matrices (PWM) corresponding to each possible spacer length N_(0−10)_. These PWM were validated in our set of 15 *B. suis* sequences by using the MAST program (Bailey and Gribskov, 1998), which allowed to retrieve most of the 44 motifs initially found. Using these 11 PWM, putative RegA-binding motifs were searched for in DNA sequences either 1 kb upstream or within 200 bp upstream and downstream of the start codon of RegA-dependent individual genes or of the first gene in each operon. The structures of operons were obtained from the literature or from predictions based on the transcription directions of the genes and their intergenic sequence lengths as indicated in DOOR (Mao et al., 2014) and BioCyc databases (Romero and Karp, 2004). The MAST program was used to scan the target sequences to search for the motifs.

### Preparation of protein samples for proteome analyses

Triplicates of eight culture samples (20 ml) of each *B. suis* WT and Δ*regA* strains have been maintained under conditions of persistence (see above) for 90 h. Bacteria from the three independent cultures were harvested and lysed in ice-cold phosphate-buffered saline (PBS), containing 10% trichlororacetic acid. The precipitated bacterial proteins were washed twice with acetone prior to air-drying. Proteins were resuspended and sonicated in the buffer previously described (Al Dahouk et al., 2009). After centrifugation, the protein concentration of each supernatant was determined with a Bradford assay and adjusted to 5 mg/ml.

### DIGE (differential in gel electrophoresis) labeling

The protein to dye ratio was 50 μg of total protein to 400 pmol dye. The internal standard was prepared by mixing equal amounts of all samples and was labeled with Cy2. For each Cy3 and Cy5 dye, 50 μg of total protein of wild-type and Δ*regA B. suis* strain were used, respectively. A dye swap was performed for the second biological replicate. The labeling was performed as reported in Al Dahouk et al. (2009).

### 2D gel electrophoresis and image acquisition

The Cy2-, Cy3-, and Cy5-labeled samples were pooled and, after addition of DTT and pharmalytes 3–10, immediately loaded onto the IPG strips to be separated on 2D-DIGE gels (one for each biological replicate) with a non-linear pH gradient of 3–10. Isoelectric Focusing was performed as previously described (Al Dahouk et al., 2009). Second dimension separation was then carried out on hand-made 13%-acrylamide gels using a SDS-glycine-Tris buffer. The 2D-DIGE gels were scanned with an Ettan DIGE Imager at a resolution of 100 μm.

### Image analysis

Protein spots were detected using the Differential In-gel Analysis mode of the DeCyder software (GE Healthcare, version 6.5.1.1). The spot detection was done estimating 5,000 spots, in the Biological Variation Analysis mode allowing inter-gel matching on the basis of the in-gel standards (Cy2) (Al Dahouk et al., 2009).

### Protein identification by peptide mass fingerprinting and MALDI-TOF/TOF-MS

In order to have sufficient material for the mass spectrometry analysis, a preparative gel was prepared (1,000 μg protein load), and stained with Coomassie Blue. Spots, that showed either a quantitative regulation or a statistical significance (*P* < 0.05), but also those that depicted a strong regulation in two biological replicates but not in the third, were selected for MS identification. The spots were manually excised and destained, then in-gel trypsin digestion and peptides enrichment were performed as described elsewhere (Al Dahouk et al., 2009). MALDI-TOF mass spectra were acquired using the 4800 Proteomics Analyzer MALDI-TOF/TOF mass spectrometer (AB Sciex) operating in the positive reflector mode (detection range 700–4,500 Da). For tandem mass spectrometry, the same instrument was used and fragmentation was based on Collision-Induced Dissociation (CID). The raw spectra were processed with the Data Explorer software (version 4.9, AB Sciex) or the GPS Explorer software (version 3.6, AB Sciex). The measured monoisotopic peptide masses and the tandem MS spectra were combined and compared to all sequences of *B. suis* in a customized database using the software Mascot (Table S7). The database consisted of the combination of four fasta files (NC_004310, NC_004311, NC_010167, NC_010169). Furthermore, all sequences were randomized and added to the database for evaluation of false positive rate.

### ICPL labeling and cleavage

In the ICPL (Isotope Coded Protein Labeling) experiment, each biological replicate was analyzed separately as technical duplicate, using the quadruplex ICPL experimental setup according to the manufacturer's procedures. WT replicates were labeled with ICPL_0_ and ICPL_6_, and Δ*regA* with ICPL_4_ and ICPL_10_. One hundred micrograms of total protein were used for each ICPL labeling reaction, performed according to the manufacturer's instructions (SERVA Electrophoresis GmbH, Germany). The enzymatic cleavage utilized trypsin (sequencing grade, porcine, SERVA Electrophoresis) and endoproteinase Glu-C (MS grade, Protea Biosciences, Inc.), with an enzyme to substrate ratio of 1:50 and 1:75, respectively. After cleavage, the peptides were acidified to 1% formic acid for subsequent mass spectrometry analysis.

### Protein identification by mass spectrometry using LC-ESI-MS/MS

For nano-LC-ESI (Electrospray ionization)-MS/MS, peptides were separated on an analytical column (C18, 25 cm length, 35°C) with a linear gradient of 5–50% solution B (A: 0.1% formic acid, B: 80% ACN, and 0.1% formic acid). Mass spectrometry was performed on a linear ion trap mass spectrometer (LTQ Orbitrap Velos, Thermo Scientific) operating in positive polarity mode online coupled to the nano-LC system. The MS method consisted of a cycle combining one full MS scan (Mass range: 300–1,500 m/z) with 10 data-dependent MS/MS events (CID; 35% collision energy). Splitting of the three biological replicates in separate MS runs allowed further statistical data analysis.

### ICPL quantification and database queries

The raw data were converted to mzXML format using a software tool from the Trans-Proteomic Pipeline. Peak detection, deconvolution, deisotoping, and quantification of the peaks were done using ICPL-ESIQuant. The quadruplet detection was performed for each run separately. Four separate database queries were done using the Mascot software as in 2D-DIGE analysis (see above). For each biological replicate, about 165 proteins were found for which the quantification was based on at least two different quadruplets and had a coefficient of variance below 40%. Furthermore, the ratios between the technical duplicates were very similar. The threshold for a regulated protein was set to 1.5 and 0.66-fold. For statistical evaluation of the biological data, the average and standard deviation of all three biological data were calculated using all four combinations of the wild-type to *regA* ratio. A one-sided *T*-Test was calculated using these ratios compared to the ICPL10/ICPL4 and ICPL6/0 that represent the technical variation applying an equal variance of the data (Table S6). Methods relative to proteome analyses are available at http://www.toplab.de/Downloads.html.

### Measurement of *in vitro* sensitivity to superoxide production

Triplicates of bacterial cultures were harvested at day 3 of our *in vitro* model of persistence, and adjusted to a density of ~10^7^ CFU/ml in PBS. ${\text{O}}_{2}^{-}$ was produced during the xanthine oxidase reaction in cell suspensions, according to a procedure, adapted in Hanna et al. (2013). At specific time points after initiating the reaction, the number of surviving bacteria was determined. The means from three independent platings of each bacterial cell suspension were calculated, and the data obtained were expressed as log10 CFU/ml at each sampling time, ± standard deviations.

### Bacterial lysates preparation

Eighty milliliters of bacterial cultures were harvested, then washed with ice-cold PBS, and bacterial pellets were resuspended in 1,200 μl of ice-cold 50 mM imidazole buffer pH = 6.8 supplemented with 1 mM DTT. After sonication of bacterial suspensions, the supernatant was obtained by centrifugation for 3 min at 13,000 RPM and filtered (0.2 μm) (Millex, France). The lysates were conserved at −20°C before further use.

### Nitrite reductase and nitric oxide reductase assay

To assess the activity of nitrite and nitric oxide reductases, ${\text{NO}}_{2}^{-}$ and NO^−^ (spontaneously transformed to NO_2_) consumption was determined by measuring the concentration of nitrite in 50 μl of each *B. suis* lysates supplemented with 10 mM NO_2_ in a final volume of 200 μl. The reaction was performed under anaerobiosis generated by GENbox packs (oxygen concentration < 0.1%) (bioMérieux, Marcy l'Etoile, France). After 30 min, the nitrite concentration was measured in 100 μl of the samples added to 100 μl of Griess reagent (Loisel-Meyer et al., 2006).

### Isocitrate lyase assay

Activity of ICL was evaluated in crude lysates according to a method adapted from the Sigma Aldrich protocol developed for purified ICL. Glyoxylate produced by the enzyme from isocitrate was detected in the presence of phenylhydrazine, by measurement of increasing absorbance at 324 nm due to accumulation of the glyoxylic acid phenylhydrazone product. Briefly, 20 μl of bacterial lysate were incubated at 30°C in 1 ml containing 25 μl imidazole 1 M, 33 μl MgCl_2_ 150 mM, 100 μl EDTA 10 mM, 33 μl phenylhydrazine 50 mM. After 30 s, 80 μl of isocitrate 250 mM were added to the reaction, and absorbance was recorded for 330 s. Activity of ICL in lysates was calculated by subtraction of the background values obtained in a reaction without isocitrate.

### Infection and growth of *B. suis* strains in the BALB/c murine model

Survival of wild-type, Δ*aceA* and the complemented Δ*aceA* strains was measured in 7-weeks-old female BALB/c mice inoculated intraperitoneally with 10^5^ CFU of either strain. On days 2, 7, 14, 28, 56, and 84, spleens and livers of six mice per *B. suis* strain were aseptically removed. After homogenization of the whole organ in PBS, bacterial counts were determined by plating serial dilutions on TS agar supplemented with antibiotics, when needed.

### Ethics statement

Mice experiments were conducted following the guidelines from the Federation of Laboratory Animal Science Associations (FELASA) which adheres to the European Directive 2010/63/EU on the protection of animals used for scientific purposes. The protocol was approved by the Centro de Investigación y Tecnología Agroalimentaria (CITA) ethical animal experiment committee (protocol 2010-01).

### Statistical methods

For *in vitro* analyses, Student's *t*-test was applied to the two sets of the three independent experiments to be compared. Variations were considered to be statistically significant at *P*-value ≤ 0.05. In mice experiments, statistical comparisons between parental strain and the *aceA* mutant were performed by a one-way ANOVA and by the Fisher's protected least significant differences (PLSD) tests. *P*-values ≤ 0.05 were considered significant.

## Results

### RegA is required for survival in an *in vitro* model of persistence

**Figure 1:**
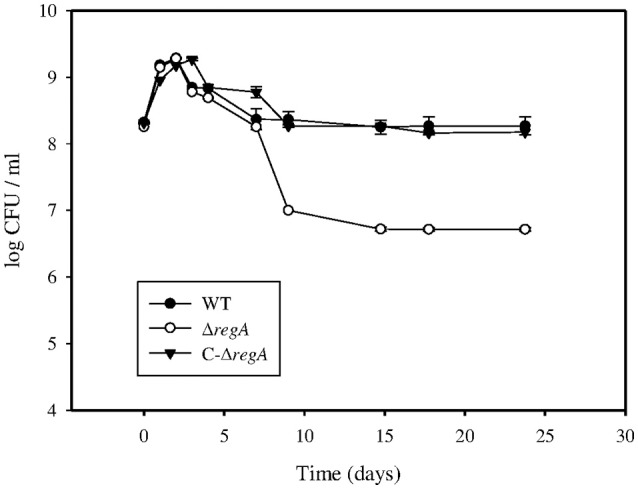
**Bacterial growth of strains in the “***in vitro*** model of persistence.”** *B. suis* 1330 wild-type (•), Δ*regA* mutant (○), and complemented Δ*regA* (C-Δ*regA*) (▼) strains were grown in minimal medium supplemented with 5 mM (NH_4_)_2_SO_4_ for 24 days. At day 1, 2, 3, 4, 7, 9, 15, 18, and 24, triplicate tubes were removed for determining viable bacteria counts. The experiment was reproduced independently three times, in triplicates each. This figure represents data means ± standard deviation of one of these experiments.

Our “*in vitro* model of persistence” consisted of progressive oxygen depletion consecutive to bacterial growth in minimal medium cultures. Several important parameters had to be adjusted during the development of the model, such as the inoculum dose (2 × 10^8^ CFU/ml) and the head space ratio (0.5). Addition of 5 mM ammonium sulfate enhanced persistence of the wild-type strain. During the first day of the experiment (Figure 1), replication of both *B. suis* wild-type (WT) and Δ*regA* strains resulted in the consumption of oxygen, which was evidenced by the following decrease of the replication rate (day 1 to day 2). A slight difference between the two strains appeared around day 3 when bacterial counts declined, indicating the onset of anaerobiosis, which was achieved at day 4 as detected by a complete color change of the indicator strip. After day 7, WT counts were found unchanged until the end of the experiment (day 24), indicating that the bacteria entered a non-proliferative persistence phase. In contrast, Δ*regA* showed a significant decrease in CFU numbers from day 7 to day 9, and bacterial counts were 1.5-log lower (*P* < 0.005) than those of the WT beyond day 15 (Figure 1): Δ*regA* was therefore strongly affected during the persistence phase. Aside a slight delay in the decrease of bacterial counts at days 4 and 9, probably linked to *regA* overexpression, the survival phenotypes of complemented Δ*regA* and WT were not significantly different (Figure 1). These results indicated that *regA* is required to allow optimal *B. suis* survival in our model of *in vitro* persistence.

### Differential expression during *in vitro* persistence of *B. suis*

According to Figure 1, genes potentially involved in the establishment of the persistent state were expected to be expressed after the initial phase of bacterial growth (day 2) and before the detection of phenotypic differences between the strains studied (day 4). Expression of RegA-dependent genes specifically induced under microaerobiosis or anaerobiosis (*fnrN, ccoN*, and *nirK*) (Abdou et al., 2013) or participating in nucleic acids metabolism (*ada* and *rpoZ*) was therefore quantified during the first 4 days. Accordingly, the whole-genome microarray analysis was performed with three independent wild-type and Δ*regA* cultures harvested at day 3, when anaerobic conditions become established but the viability of both strains was still rather similar. 447 genes were detected with a variation level of the hybridization intensities superior to 2, indicating a potential regulation by RegA (Table S1). In the wild-type strain, 203 (45%) and 244 (55%) genes were found potentially induced and repressed, respectively. Whole proteomes were compared using the 2D-DIGE and ICPL techniques to quantify proteins prepared from subsequent triplicate cultures of both strains at 90 h. Most strikingly, 30% (28) and 70% (66) of the RegA-dependent proteins increased and decreased in concentration, respectively (Table S2).

**Figure 2:**
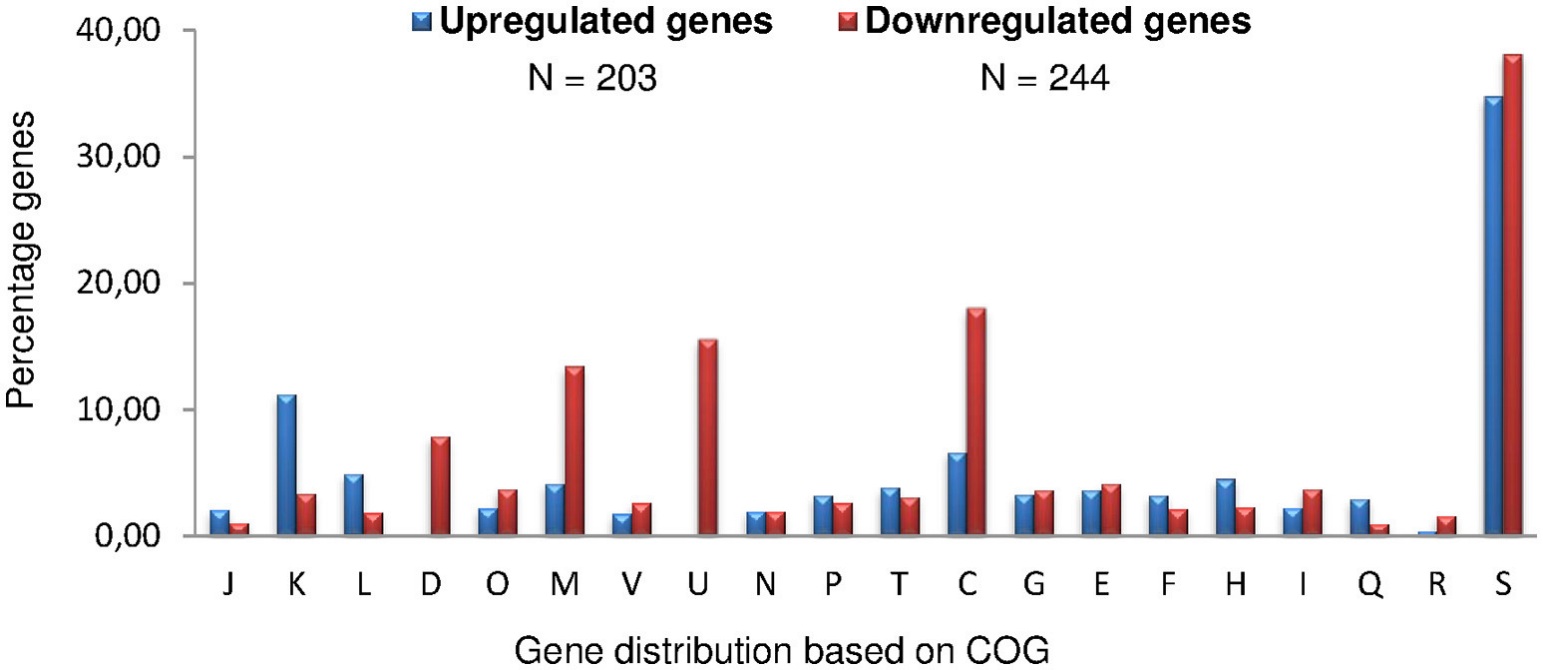
**Distribution of ***B. suis*** genes under RegA control in COG functional categories**. A total of 447 genes were differentially expressed at day 3 of our *in vitro* model of persistence. Numbers of up-regulated (blue bars) and down-regulated (red bars) genes in the wild-type strain refer to the presence of a functional *regA*. J, Translation, ribosomal structure, and biogenesis; K, Transcription; L, DNA replication, recombination, and repair; D, Cell division and chromosome partitioning; O, Post-translational modification, protein turnover, chaperones; M, Cell envelope biogenesis, outer membrane; V, Defense mechanisms; U, Intracellular trafficking, secretion, and vesicular transport; N, Cell motility and secretion; P, Inorganic ion transport and metabolism; T, Signal transduction mechanisms; C, Energy production and conversion; G, carbohydrate transport and metabolism; E, amino acid transport and metabolism; F, nucleotide transport and metabolism; H, coenzyme metabolism; I, Lipid metabolism; Q, Secondary metabolites biosynthesis, transport, and catabolism; R, General function prediction only; S, Function unknown.

According to the COGs classification (Material and Methods), differentially expressed genes were distributed over all functional groups (Figure 2). A very strong representation of genes encoding proteins with unknown functions (34%) was detected. Taking into account genes with annotated functions only, the functional categories K: transcription (12.2%), M: cell envelope biogenesis (11.9%), and C: energy production and conversion (16.7%) were the most frequently represented (Table S1). A large number of genes involved in transcription were induced by RegA (11.6% of all class K genes), whereas 14 and 17.5% of genes involved in bacterial envelope biogenesis (M) and in energy metabolism (C), respectively, were repressed (Figure 2). Moreover, all the differentially expressed genes belonging to class D (cell division and chromosome partitioning) were found down-regulated in the WT strain (~8% of total category D genes). Eight of the nine genes belonging to intracellular trafficking and secretion (class U) were part of the *virB* operon and were down-regulated in the WT strain (Figure 2). Importantly, protein quantifications obtained by 2D-DIGE and ICPL (Table S2) confirmed the transcriptome data concerning class C genes. Abundance of all class M proteins and most of those belonging to J, E, and G categories (translation, amino acid, and carbohydrate metabolism, respectively), detected as being RegA-controlled, was reduced (Table S2). Taken together, transcriptome and proteome data confirmed that RegA acts mainly as a repressor, consistent with growth arrest and establishment of persistence.

Genetic validation of a selection of 62 differentially expressed genes representing most of the COG functional categories, was performed by quantitative PCR (RT-qPCR) (Table S3). Only seven genes were not validated, because of opposite (BR1781 and BR1043) or below threshold (≤1.5) (BR0111, BR1017, BR1358, BR1648, and BRA0066) fold-change values. It has to be noticed that BR1358 (*ureC-2*) and BRA0066 (*virB4*) belong to operons which were validated by RT-qPCR targeting other genes (Table S3). Forty-six genes showed a |−ΔΔCt|≥1 (representing a fold change ≥ 2 or ≤ 0.5) (Table S3), and 9 other genes were also found adequately regulated but with a slightly lower rate, between −0.74 and −0.97 (Table S3). Namely, 55 genes (88.7%) having a significant fold change were validated, as both microarrays and RT-qPCR approaches confirmed identical patterns of RegA regulation. The transcriptome analysis identified 447 genes, of which 396 are potentially regulated by RegA, and therefore we can postulate that ~12% of the 3,271 genes of *B. suis* may be under the control of RegA.

### Possible function of RegA as both direct and indirect regulator

Strikingly, among the differentially expressed genes involved in transcription (K class) and induced by RegA (78%), all of them except two (i.e., 26/28) encode transcriptional regulators (Table S1). Hence, RegA can exert its regulatory function also indirectly by positively interacting with expression of genes encoding regulatory proteins which are implicated in transcription of genes under RegA control.

Since RegA of *R. capsulatus, R. sphaeroides* (PrrA), *B. suis, B. melitensis* (Elsen et al., 2004), and *B. abortus* (PrrA) (Wu and Bauer, 2008) share about 70% sequence identity and identical putative HTH (Helix-Turn-Helix) DNA-binding domains, we hypothesized that RegA activity in *B. suis* may be mediated through binding to highly conserved DNA sequences. The *R. sphaeroides* RegA-binding sequence is present upstream of the gene in the case of gene induction (possibly more than 900 bp from the putative initiation codon; Mao et al., 2005) or at its 5′-end vicinity, in the case of gene repression (Eraso et al., 2008). PWM (Positional Weight Matrices) (Material and Methods) were used to scan genes validated as being regulated by RegA using RT-qPCR (Table S3). Only putative RegA-binding motifs detected with the MAST program in DNA sequences located up to 1 kb upstream or close to the putative initiation codon (±150 bp) of induced or repressed genes were considered (Table S3).

Interestingly, all the induced genes possess potential RegA-binding sites in the regulatory regions, whereas sites in a position susceptible to impair correct transcription initiation were detected in only 64% of the repressed genes, regulators excluded (Table S3). This indicated that indirect action of RegA may be more frequent in the case of gene repression.

### Up-regulation by RegA in the *in vitro* model of persistence

**Figure 3:**
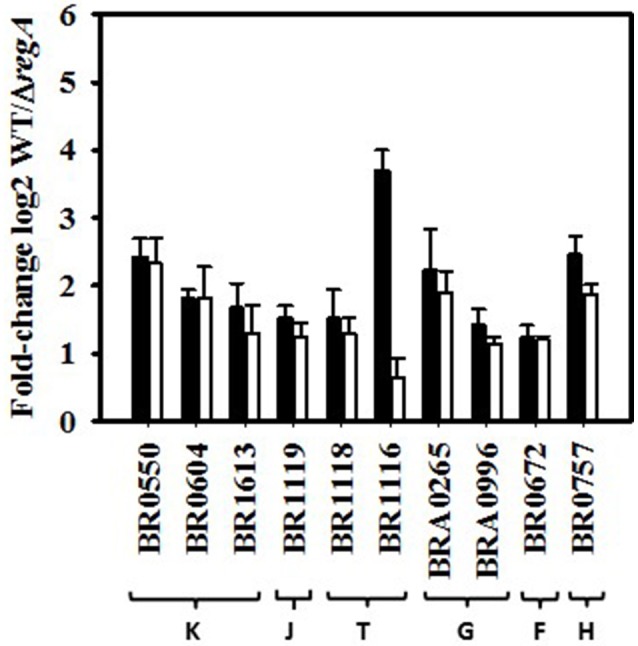
**Fold-change of RegA-up-regulated genes calculated from normalized microarray data and RT-qPCR**. Increased expression levels in wild-type strain with respect to the Δ*regA* mutant are expressed as log2-values of the normalized hybridization ratios (white bars) compared to those obtained by RT-qPCR (−ΔΔCt) (black bars). Each qPCR was performed in technical triplicates. Genes represented are selected from the following functional groups. K, Transcription; J, Translation, ribosomal structure, and biogenesis; T, Signal transduction mechanisms; G, carbohydrate transport and metabolism; F, nucleotide transport and metabolism; H, coenzyme metabolism. (BR0604: *feuP*, BR1119: *nifR3*, BR1118: *ntrB*, BR1116: *ntrY*, BRA0996: *rbsB-3*, BR0672: Mut/nudix, BR0757: *hemB*). BR1116 showed a hybridization ratio below threshold (1.6). The mean ± standard deviations (error bars) of microarrays and RT-qPCR data were calculated from three independent experiments.

The K class (transcription) possesses the highest number of up-regulated genes (Figure 2), the vast majority of which encodes transcriptional regulators of different pathways (Table S1) or of two-component systems, such as BR0604 (*feuP*)/BR0605 (*feuQ*) (Figure 3) (Tables S1, S3). RegA induced BR1118 (*ntrB*) and BR1119 (*nifR3*) (Figure 3) of the *ntrBC* operon, encoding the two-component system involved in regulation of nitrogen metabolism and depending on glutamine availability (Ronneau et al., 2016). Since the downstream operon *ntrYX* is also regulated by PrrA (RegA) in *B. abortus* (Carrica et al., 2013), we compared *ntrY* (BR1116) expression levels in the WT and Δ*regA* strains by RT-qPCR. The 3.7-fold change (−ΔΔCt) obtained (Figure 3) confirmed a strong positive impact of *B. suis* RegA on *ntrYX* expression in our model.

Detection of only one regulator, MucR, by the ICPL proteome analysis (Table S2) could be explained by transitory expression, as for *fnrN* used as a sentinel gene for determination of the time-point chosen for transcriptome analysis, and/or low-level expression of transcription factors. Hypoxia may therefore be considered as another stress factor potentially able to affect MucR expression, in addition to osmotic stress (Mirabella et al., 2013) and nutrient starvation (Al Dahouk et al., 2013). MucR is known to activate a ferrous iron transporter in *B. abortus* (Caswell et al., 2013; Elhassanny et al., 2013). Interestingly, RegA was found to induce two operons, BR1344/1347 and BRA0675/0678 encoding iron compound ABC transporters (Table S1) and two proteins (Table S2), Dps, a ferritin-like protein (Calhoun and Kwon, 2011), and bacterioferritin, all of the P class (inorganic ion transport and metabolism). Despite the fact that our proteome and transcriptome analyses did not detect pairs of corresponding proteins and genes, both approaches yielded coinciding results. The production profiles of MucR and both Dps and bacterioferritin proteins were strictly parallel to those previously determined under long-term harsh nutrient starvation of *B. suis* (Al Dahouk et al., 2013). These proteins could allow iron storage and homeostasis to contribute to long-term viability in media containing the same low ferrous iron concentrations. The RegA-dependent induction of MucR and of operons or proteins participating in iron uptake and storage in *B. suis* may therefore be linked.

All eight RegA-induced genes involved in DNA metabolism (class L) encode enzymes participating in DNA recombination and repair [recombinases or transposases (Table S1)], possibly to counteract potential DNA-damage occurring in bacteria exposed to low oxygen levels. Similarly, two up-regulated genes (BRA0607 and BR0672) of the nucleotide metabolism (class F) produce “house-cleaning” hydrolases of the MutT/nudix family (Table S1, Figure 3) that target damaged metabolites accumulated during stress, for example mutagenic forms of dGTP (Bessman et al., 1996).

The majority of the RegA-induced genes involved in carbohydrate (class G), or amino acid transport and metabolism (class E) encode ABC transporters for sugars, such as BRA0995/0996 with high affinity for D-ribose (Lopilato et al., 1984), or for peptides and amino acids, respectively (Tables S1, S3, Figure 3).

### Genes and proteins down-regulated by RegA in the *in vitro* model of persistence

**Figure 4:**
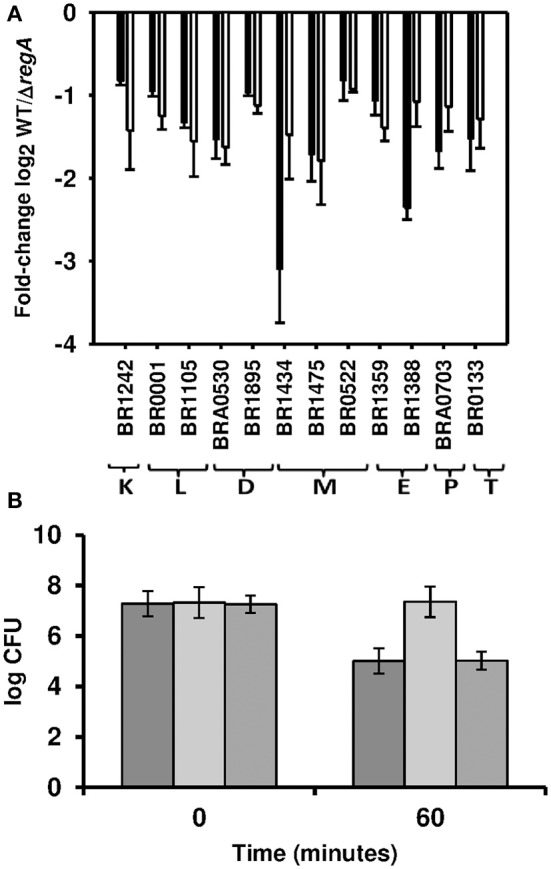
**Validation of down-regulation by RegA. (A)** Fold-change of RegA-down-regulated genes are expressed as log2-values of normalized microarray data (white bars) and of RT-qPCR (−ΔΔCt) (black bars). Each quantitative PCR was performed in technical triplicates of three independent samples. Genes represented are selected from the following functional groups K, transcription; L, DNA replication, recombination, and repair; D, Cell division and chromosome partitioning; E, amino acid transport and metabolism; M, Cell envelope biogenesis; P, Inorganic ion transport and metabolism; T, Signal transduction mechanisms. (BR1242: *rpoC*, BR0001: *dnaA*, BR1434: *mraY*, BR1475: *omp28*, BR0522: *gmd*, BR1359: *ureE-2*, BR1388: *ilvH*, BRA0703: *sodC*, BR0133: *regB*). **(B)** SodC activity on day 3 in the *in vitro* model of persistence. Enumeration of the surviving bacteria after 60 min of exposure to ${\text{O}}_{2}^{-}$ generated by the xanthine oxidase reaction of *B. suis* wild-type (dark gray bars), Δ*regA* mutant (light gray bars), and complemented *regA* mutant (medium gray bars) strains. Standard deviations are reported for the means of three independent experiments.

The predicted individual gene (Romero and Karp, 2004; Mao et al., 2014) *regB* (BR0133) encoding the sensor histidine kinase was repressed in the WT strain (Figure 4A). This suggests that RegA (BR0137) could exert a negative feedback on *regB*, as reported for the homologs in *R. sphaeroides*, where autoregulation prevents excessive production of PrrB (RegB) and thereby of activated PrrA (RegA) and its target genes (Oh et al., 2001). The functional class of energy production (C) (see below) and of envelope biogenesis (M) contain the highest percentages of RegA-down-regulated genes with defined annotations (Figure 2), both having a very high proportion of repressed RegA-dependent genes (37/49 and 27/35, respectively) (Table S1). Five of the six genes belonging to an operon involved in LPS biosynthesis were repressed by RegA: BR0518, BR0519/0520 of class G producing the O-antigen export system, BR0521/0522 (Table S3, Figure 4A) encoding perosamine synthase and GDP-mannose 4,6-dehydratase (Table S1). Proteome data were perfectly consistent since all identified class M proteins (Table S2) were down-regulated, BR0522 included. MurB (Table S2) and four genes of the *murEF*-*mraY*-*murD*-*ftsW* operon were found to be repressed by RegA (Tables S1, S3, Figure 4A), as well as *ddlA* (BR1271) and BR1172. These results confirmed that peptidoglycan biosynthesis was down-regulated by RegA during establishment of persistence. **Three** major outer membrane proteins, Omp25, Omp28, Omp31, together with the corresponding genes of the latter two [BR1475 (Figure 4A) and BR1622 (Table S1)], were also under negative RegA control. Omp31 is a porin and its down-regulation could indicate a RegA-dependent protection of the bacterium.

RT-qPCR-validation (Table S3) confirmed that, under our conditions, RegA repressed the *virB* operon (all *virB* genes, except *virB*7-9, and *virB*12) (Table S1) and its activator, *vjbR*. Identification of a putative RegA-binding site 90 bp downstream of the initiation codon of *vjbR* but not at the 5′ ends of *virB1* and *virB2* (Table S3) suggested indirect regulation of the *virB* operon, possibly via direct repression of *vjbR*.

Transcriptome and proteome analyses converged to the observation of a RegA-dependent decrease of transcriptional activity. In fact, ICPL experiments (Table S2) validated down-regulation of the RNA polymerase subunit beta' (*rpoC*, BR1242) (Table S1, Figure 4A), and both proteomic methods (Table S2) also detected reduced abundance of the catalytic subunit beta. The class J (translation)-derived proteins were predominantly down-regulated (Table S2), while this category was underrepresented in the transcriptome data. We confirmed by RT-qPCR the RegA-dependent down-regulation of genes BR0001 (*dnaA*) and BR1105 playing a role in DNA replication (class L), and of the three genes involved in cell division (class D) (Tables S1, S3, Figure 4A).

In class E (amino acid transport and metabolism), 25 genes were found to be down-regulated by RegA. One of the four detected genes belonging to the *ure-2* operon was validated by RT-qPCR (Tables S1, S3, Figure 4A), they encode urease accessory proteins responsible for urea and nickel transport in *B. abortus* (Sangari et al., 2010). Bacteria in a neutral medium may not require elevated expression of the urea transporter, necessary for the acid-dependent induction of urease activity in *B. abortus*. Conversely, RegA may participate in the basal urease activity, exclusively produced from the *ure-1* operon in both *B. suis* and *B. abortus* (Bandara et al., 2007; Sangari et al., 2007), as indicated by the higher production of the subunit gamma (UreA-1) (Table S2) in the WT strain.

Numerous genes (Table S1) and enzymes (Table S2) belonging to various amino acids biosynthesis pathways were found under the negative control of RegA. The superpathway of isoleucine, leucine and valine synthesis was identified at both the transcript and protein levels, validated by RT-qPCR targeting BR1388 (*ilvH*) (Figure 4A). Genes involved in biosynthesis of histidine, lysine or methionine and enzymes participating in arginine or serine metabolism were also down-regulated by RegA. BR0765 (*glyA*) and its product responsible for transformation of serine into glycine were found coherently regulated (Tables S1, S2). Repression of genes or proteins involved indicated that *B. suis* limited utilization of the relevant pathways under anaerobic conditions, in contrast to what could be expected during growth in a minimal medium devoid of amino acid sources.

### SodC is down-regulated by RegA under *in vitro* depletion of oxygen

The inorganic ion transport and metabolism category (P) comprises 9 genes down-regulated in the WT strain (Table S1). Notably, we detected BRA0703 (*sodC*) encoding a Cu, Zn superoxide dismutase, confirmed by RT-qPCR (Figure 4A), and the catalase (BRA0355) (Table S2), both participating in detoxification of oxidative compounds. Activity of the superoxide dismutase was estimated by the quantification of bacterial survival to artificially produced superoxide anions, which showed that survival of Δ*regA* was 100-fold higher (*P* < 0.001) than that of WT (Figure 4B). Survival of the mutant decreased following complementation with the intact *regA* gene and was identical to that of the WT strain (Figure 4B). These results corroborated the repression of *sodC* expression by RegA at day 3 of our model of persistence, which reflects the lack of oxygen resulting in a reduced production of reactive oxygen intermediates.

### RegA coordinates bacterial adaptation to low oxygen levels

**Figure 5:**
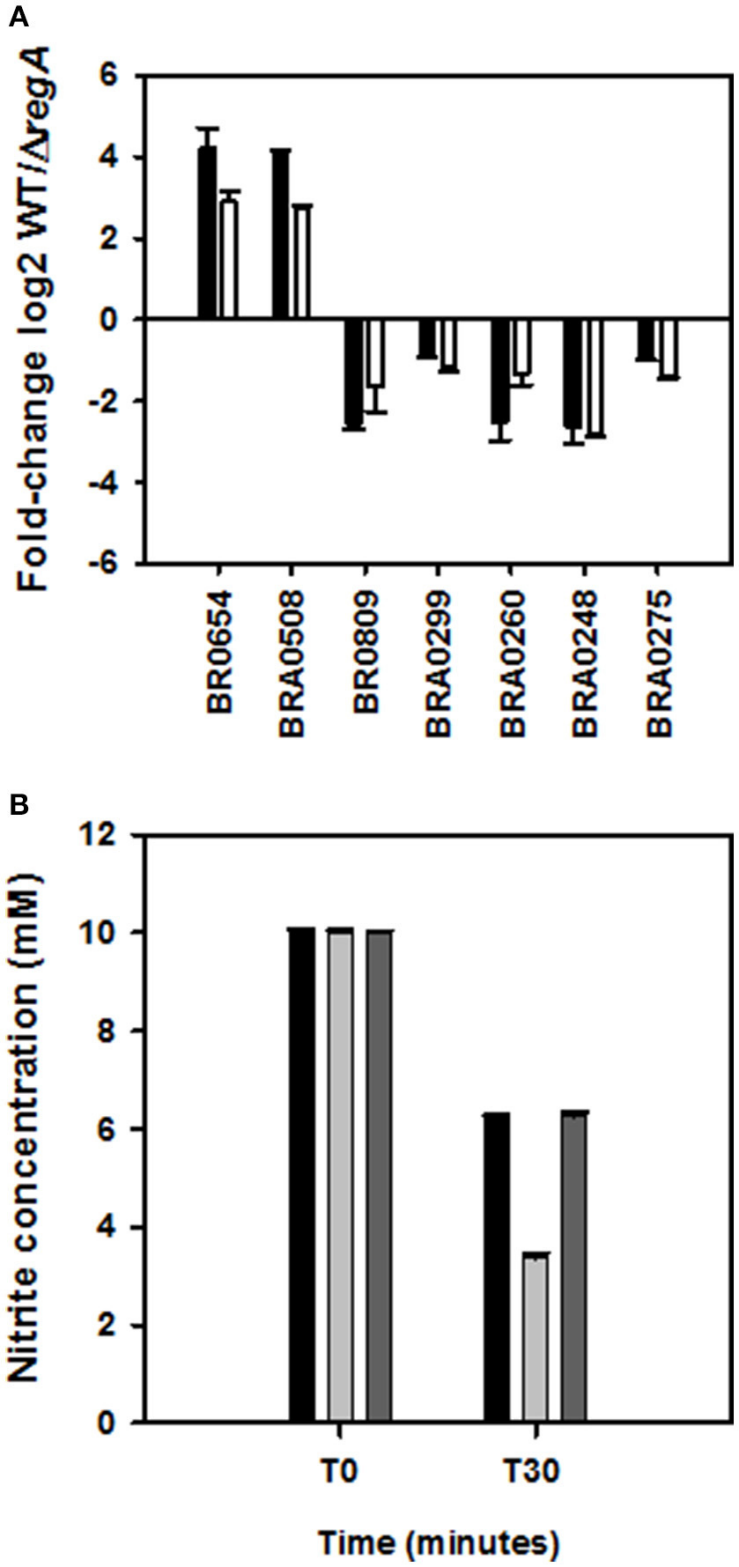
**Validation of differentially expressed genes involved in adaptation to low oxygen levels. (A)** Expression ratios of RegA-dependent genes are shown as log2-values of the normalized hybridization ratios (white bars) and of RT-qPCR (−ΔΔCt) (black bars). Each quantitative PCR was performed in technical triplicates of three independent samples. (BR0654: *fnrN*, BRA0508: *cydD*, BR0809: *nuoH*, BRA0299: *narG*, BRA0260: *nirK*, BRA0248: *norC*, BRA0275: *nosZ*). **(B)** Nitrite and nitric oxide consumption in wild-type (black bars), Δ*regA* mutant (gray bars), and complemented *regA* mutant (dark gray bars) after 30 min under anaerobiosis. The mean ± standard deviations (error bars) of three independent experiments are shown.

In the *in vitro* model of persistence, ~15% (37/244) and 5.9% (12/203) of the respectively down- and up-regulated genes were identified as being involved in energy production and conversion (class C) (Table S1). The oxygen-dependent regulator *fnrN* (BR0654), *cydD* (BRA0508) and *cydC* (BRA0509) (Table S1) of the *cyd* operon encoding the *bd* ubiquinol oxidase of high affinity for oxygen were very strongly induced by RegA, as shown previously (Abdou et al., 2013). One and five potential RegA-binding sites were accurately detected in *cydD* and *fnrN* upstream sequences, respectively (Table S3). RT-qPCR results (Table S3, Figure 5A) proved a *fnrN* induction fold-change similar to that obtained under direct anaerobiosis, thus indicating that oxygen concentration was diminishing toward anoxic conditions at day 3 of the *in vitro* model of persistence. We also confirmed that RegA induced expression of both ubiquinol and cytochrome *c* oxidases under these conditions (Abdou et al., 2013). In fact, strong induction of cytochrome *bc1* components (complex III of the respiratory pathway; operon BR1543/1541) (Tables S1–S3), of cytochrome *c* itself (BR0039), as well as of genes participating in its biogenesis (BR0096 and BR0607/0608, category O) (Tables S1, S3), indicated that *B. suis* may use an electron transport chain terminating with a cytochrome *c* oxidase. Nevertheless, the only cytochrome *c* oxidase under strong positive control of RegA was the *aa*_*3*_-type (BR0467 and BR0468) (Tables S1, S3), a quite unexpected finding given that it possesses a lower oxygen affinity than the *cbb*_*3*_-type (Ekici et al., 2012). The operon encoding the latter (BR0363/0360) was, however, also well-expressed in the WT strain (microarray data, GEO accession number GSE87538). The *aa*_*3*_-type oxidase of *B. suis* could be active under our conditions, in accordance with detection of the cytochromes *a* and *a*_*3*_ only in late-log phase *B. abortus* (Rest and Robertson, 1975). It was suggested that its counterpart in *P. aeruginosa* participates in energy preservation, given the highly efficient proton translocation by the studied *aa3*-type oxidases (Kawakami et al., 2010). However, the *nuoA-N* operon encoding NADH dehydrogenase (complex I of the respiratory chain) was repressed (Table S1, Figure 5A), and one potential RegA-binding site was accurately detected in the vicinity of the initiation codon (Table S3). This possibly reflected low residual activity of the respiratory chain, with energy production sufficient for persistence.

Genes BRA0299, BRA0260, BRA0248, and BRA0275 encoding catalytic subunits (except BRA0248) of the four reductases Nar, Nir, Nor, and Nos involved in denitrification, were detected as being repressed (Figure 5A), as well as additional genes belonging to the four operons (Table S1). In agreement, consumption of NO_2_/NO correlated with the respective nitrite and NO reductase activities in lysates of strains obtained at day 3, since Δ*regA* eliminated 6.6 mM ± 0.1 mM of nitrites, instead of only 3.8 mM ± 0.05 mM used by the WT and complemented strains (Figure 5B). *nnrA*, encoding the activator of *nir, nor*, and *nos* (Haine et al., 2006; Carrica et al., 2013), was also found adequately regulated (Table S3). Potential RegA-binding sites were found in relevant positions of *nnrA*, but also of each of the four operons (Table S3), indicating that down-regulation of the three aforementioned operons could be direct or possibly acting via repression of *nnrA*. Unexpectedly, these results were opposed to RegA-mediated *nirK* induction observed under direct anaerobic denitrifying conditions (Abdou et al., 2013). Interestingly, they also indicate that the same *nirK* gene can be under positive or negative control by RegA, as reported in *R. sphaeroides* (Laratta et al., 2002), probably to ensure the best adaptive response depending on the availability of metabolites and on experimental conditions. In fact, the initial (day 1) RegA-dependent *nirK*-induction in the WT strain, a probable adaptation to hypoxia, reversed to repression at day 2 until at least day 4. This might be associated with lack of nitrate in the medium and the use of an alternative electron acceptor such as fumarate (see below).

At the protein level, RegA-dependent repression of the beta-subunit of the ATP-synthase (Table S2) also indicated reduced energy production in the WT strain entering the persistence phase. These data lead us to the conclusion that RegA is a key player in efficient adaptation of *Brucella* to oxygen deficiency.

### RegA contributes significantly to the regulation of energy metabolism

**Figure 6:**
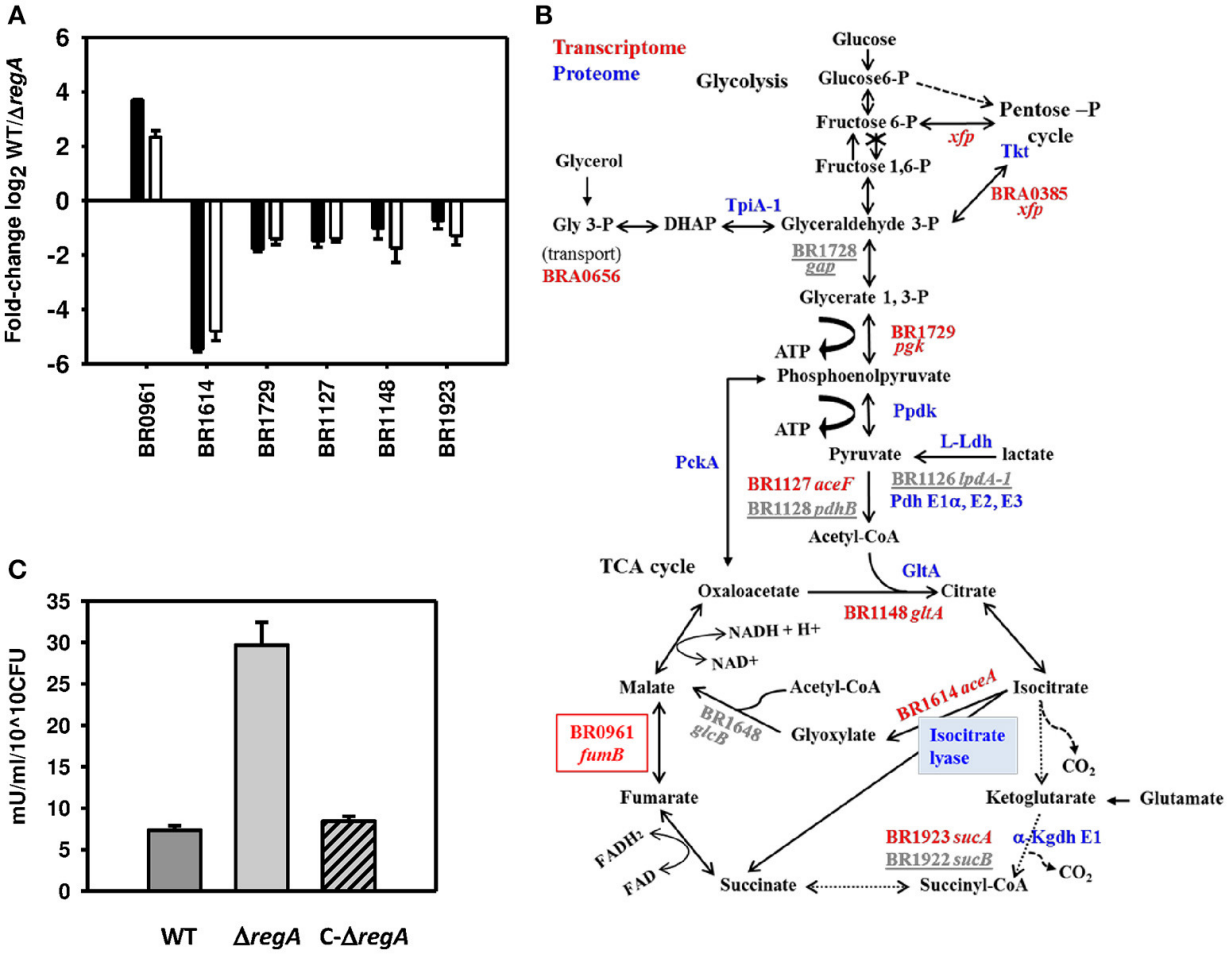
**RegA-dependent regulation of pathways involved in energy production. (A)** Fold-change of differentially expressed genes (BR0961: *fumB*, BR1614: *aceA*, BR1729: *pgk*, BR1127: *aceF*, BR1148: *gltA*, and BR1923: *sucA*) are represented as the log2-values of hybridization ratios (white bars) and of the RT-qPCR (−ΔΔCt) (black bars). Each quantitative PCR was performed in technical triplicates of three independent samples. The mean ± standard deviations (error bars) of the three independent experiments are shown. **(B)** Representation of the pathways under the control of RegA. The crossed out third step of glycolysis indicates the lack of the corresponding enzyme phosphofructokinase. Dashed arrows represent the shunted reactions when the glyoxylate bypass is active. RegA–dependent proteins (in blue) and genes validated by RT-qPCR (in red) or not tested (underlined gray) are shown. The red box represents the only gene up-regulated by RegA. BR1781 (*pyc*) and BR1017 (*maeB*), not validated, were omitted for clarity. **(C)** ICL activity in crude lysates of the wild-type (WT) (dark gray bar), Δ*regA* mutant (Δ*regA*) (gray bar) and complemented Δ*regA* mutant (C−Δ*regA*) (dashed gray bar) strains was measured as described in Methods. Means and standard deviations of three independent experiments are reported.

Remarkably, 75.5 (37/49) and 76.5 (13/17)%, respectively, of the RegA-dependent genes and proteins belonging to the COG category C (energy production and conversion) were found to be repressed. This incited us to measure transcription levels of numerous genes engaged in central carbon metabolism (Table S3, Figure 6A). Interestingly, the genes and proteins involved in glycolysis and tricarboxylic acid (TCA) cycle were all down-regulated in the WT (Figure 6B), except for the triosephosphate isomerase TpiA1 (Table S2) and *fumB*-encoded fumarate hydratase, the latter being strongly induced by RegA (Figure 6A). The relative contradiction to our previous study, suggesting that low oxygen conditions applied on cultures in rich medium can activate glycolysis (Al Dahouk et al., 2009), is most likely caused by differences in medium and conditions selected. The strong repression of the genes encoding each subunit of the glycerol-3-phosphate ABC transporter (Tables S1, S3) may be the consequence of a presumably fair level of glycerol import from the medium (30 g/L) (see discussion), possibly phosphorylated by the glycerol kinase (BRA0433). Glycerol-3-P may then be used by a specific dehydrogenase (BR1889, *gpsA*) to produce dihydroxyacetone-phosphate (DHAP), the substrate of TpiA1 (Figure 6B) whose induction may therefore be in line with repression of the glycerol-3P transporter. Instead, slight induction of this glycerol-3P transporter by anaerobiosis under denitrifying conditions and by nutrient starvation may be linked to the lack of glycerol in medium used in our previous analyses (Al Dahouk et al., 2009, 2013). Nevertheless, our studies concur with a possible use of glycerol as carbon source by *B. suis* under both conditions of oxygen deficiency.

Increased production of TpiA1 isomerase could sustain synthesis of glyceraldehyde-3-P from DHAP (Figure 6B), possibly providing enough of this essential intermediate of glycolysis under our particular conditions. This might make the pentose phosphate cycle (PPC) dispensable, although essential for glycolysis in *Brucella* devoid of phosphofructokinase (Figure 6B). In agreement, ABC-transporter periplasmic binding proteins (BRA0858 and BRA1150) specific for erythritol and xylose (Table S2), whose respective metabolites erythrose-4-P and xylulose-5-P can be used in carbohydrate metabolism solely via the PPC, were repressed by RegA. This is in contrast with the induction of the large majority of genes encoding sugar ABC transporters, possibly reflecting the lack of substrates in the medium. Moreover, *xfp* (BRA0385) producing phosphoketolase (Tables S1, S3) and transketolase (Tkt, BR1727) (Table S2), were found negatively controlled by RegA, confirming the down-regulation of enzymes of the PPC. Three enzymes, phosphoenolpyruvate carboxykinase (PckA), pyruvate phosphate dikinase (PpdK), and L-lactate dehydrogenase (L-Ldh), which catalyze reactions potentially supplying phosphoenolpyruvate or pyruvate for gluconeogenesis and for amino acids synthesis (Figure 6B), were also repressed by RegA (Table S2). RT-qPCR validated repression of five out of nine genes encoding enzymes of the central metabolism (Table S3, Figure 6A), among which BR1729 (*pgk*) whose product, phosphoglycerate kinase, generates ATP. Corresponding genes [BR1127 (*aceF*), BR1126 (*lpdA*-1)] (Tables S1, S3, Figure 6A) and proteic components E2 and E3 (dihydrolipoamide dehydrogenase) of the pyruvate dehydrogenase complex as well as its E1 α-subunit (BR1129) (Table S2), and BR1128 (*pdhB*) encoding E1 βsubunit (Table S1) were also identified as being repressed by RegA (Figure 6B). Moreover, down-regulation of genes BR1148 (*gltA*), BR1923 (*sucA*), and BR1922 (*sucB*), encoding the citrate synthase, first enzyme of the TCA cycle, and the E1 and E2 components of the α-ketoglutarate dehydrogenase complex (Tables S1, S3), respectively, were further validated by proteome analyses, except E2 (Table S2, Figures 6A,B). Lower concentrations of the E3 component (dihydrolipoamide dehydrogenase; see above) may also contribute to a reduced activity of this enzymatic complex. Furthermore, ICL and malate synthase of the glyoxylate shunt (Figure 6B) were also identified by microarray analysis as being down-regulated in the WT strain. BR1614 (*aceA*), encoding ICL which catalyzes the production of glyoxylate and succinate (Figure 6B), was the most down-regulated gene, with a log2-value of the hybridization ratios of –4.8. Validation by RT-qPCR (−ΔΔCt = −5.4) (Table S3, Figure 6A) showed that this gene is indeed expressed in the WT (ratio of mRNA concentrations [*aceA*/(16S × 100) = 0.5 ± 0.08], and its high level of expression in Δ*regA* (ratio of mRNA concentrations [*aceA*/(16S × 100) = 20.1 ± 0.7] therefore led us to consider the *aceA* “repression” as an over-expression in the mutant. Proteome analyses confirmed higher ICL production by this strain (Table S2). At day 3 of our model, the 4-fold higher ICL activity in the Δ*regA* strain (*P* < 0.001) (Figure 6C), and restoration of the wild-type level after complementation with the intact *regA* gene, confirmed ICL over-production in the mutant. As a control, the Δ*aceA* mutant (Material and Methods) showed no ICL activity in the presence or the absence of isocitrate. Interestingly, up-regulation of a single gene from the TCA cycle, BR0961 (*fumB*) encoding fumarate hydratase B, was confirmed in the WT strain (Figures 6A,B). In *E. coli*, this anaerobic enzyme produces fumarate used as an alternative electron acceptor under anoxia.

### Isocitrate lyase is essential for *in vitro* persistence and *in vivo* virulence of *B. suis*

The role of ICL was evaluated by studying the phenotype of Δ*aceA* in the “*in vitro* persistence model” either in the absence or in the presence of fatty acids, expected to stimulate the glyoxylate bypass in *B. suis*, as their oxidation results in high levels of acetyl-CoA (Figure 6B).

**Figure 7:**
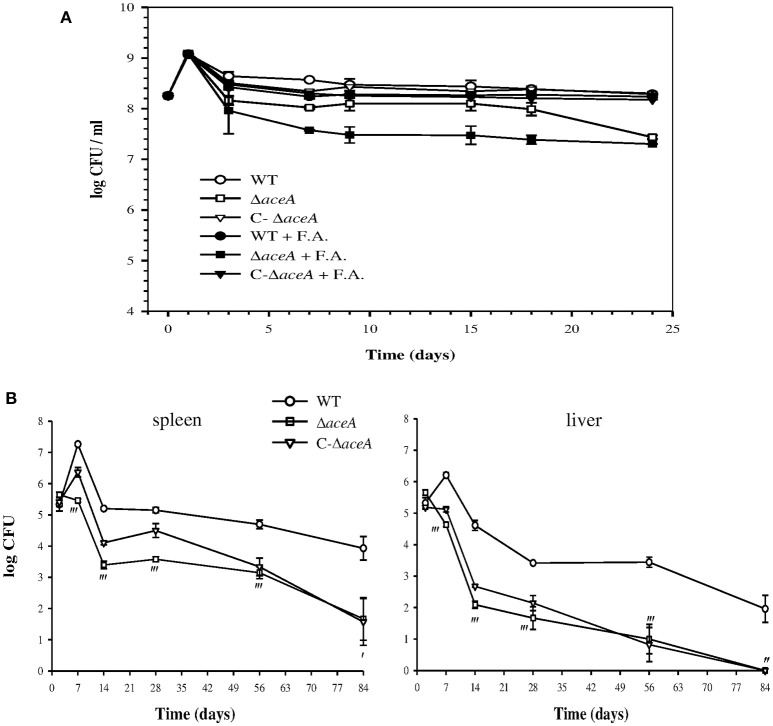
**Impact of ***aceA*** on ***B. suis*** survival during ***in vitro*** persistence and mice infection. (A)** *B. suis* 1330 wild-type (WT), Δ*aceA* mutant and complemented Δ*aceA* (C-Δ*aceA*) strains were grown for 24 days in minimal medium containing 5 mM (NH_4_)_2_SO_4_, supplemented (+ F. A.) or not with 0.05 mM sodium palmitate. At each time point, tubes were removed to determine viable bacteria counts. Means and standard deviations of three independent experiments are reported. **(B)** The above-cited strains were used to inoculate BALB/c mice intraperitoneally with 10^5^ CFU and were recovered from spleens (left) and livers (right) at 2, 7, 14, 28, 56, and 84 days post-infection. Error bars represent the standard error of the mean. Results of statistical analysis are indicated by apostrophes (′*P* < 0.05; ″*P* < 0.01; ‴*P* < 0.001).

After comparable growth followed by a drop in bacterial counts, supplementation with fatty acids, regardless their nature [sodium palmitate or palmitic acid (C16), capric acid (C8), or oleate (C18 saturated)] and concentration (0.05, 0.5, 2, or 5 mM), gave rise to an increasing defect in survival of Δ*aceA*, ~10-fold beyond day 3 (Figure 7A). Without fatty acids, the persistence of Δ*aceA* shifted down between day 15 and day 25, indicating that conditions of the persistence model evolve with time. The complementation of Δ*aceA* (C-*aceA* in Figure 7A) allowed the restoration of the WT phenotype, and of the enzymatic activity (see above and data not shown). These results confirmed the loss of ICL activity as a direct consequence of *aceA* inactivation and the requirement of a functional ICL for optimal bacterial persistence under anoxia in the presence of low fatty acid concentrations.

The behavior of WT, Δ*aceA* and complemented Δ*aceA* strains was similar during infection of both resting or IFNγ- and LPS-activated J774A.1 murine macrophage-like cells (Loisel-Meyer et al., 2006), under standard and microaerobic conditions. A possible role of *aceA* in pathogenesis *in vivo* was then assessed in a murine model of infection. In contrast to the WT strain, Δ*aceA* did not display bacterial growth during the acute phase (day 7), a phenotype possibly correlated to the lower oxygenation inside the target organs than in the first step of our *in vitro* model (Jiménez de Bagüés et al., 2007). In the spleen, after a persistence phase (days 14–56) parallel to that of the WT, elimination of the mutant was accentuated during the last 4 weeks of infection, resulting in a decrease of 2.3 logs in bacterial counts (*P* < 0.05), and two animals cleared infection (Figure 7B). The *aceA* mutant was more attenuated in the liver where it was eliminated shortly after colonization (Figure 7B), resulting in a higher difference in viable counts between the two strains than in the spleen at day 14 [2.5 logs (*P* < 0.001) vs. 1.8 log (*P* < 0.001)]. The mutant was unable to establish a chronic infection and was totally eliminated at 12 weeks post-infection (Figure 7B). The stronger impact of ICL loss in the liver is in line with the lower oxygen concentration within this organ, potentially aggravated by the formation of granuloma during chronic infection (Abdou et al., 2013). Another non-exclusive hypothesis could be an increased requirement of ICL activity consecutive to the possible use of fatty acids present in the liver. Complementation of the mutant strain resulted in partial restoration of the parental phenotype in the spleen only, with intermediate growth in the acute phase but survival similar to that of the mutant starting at day 56 (Figure 7B). Having checked the presence of the plasmid ensuring complementation in bacteria recovered at day 28, we propose that early strong overexpression of *aceA* may result in over-activation of the glyoxylate pathway. High ICL activity could be detrimental to replication and persistence in the liver due to overproduction of succinate (Figure 6B), leading to succinate dehydrogenase dysfunction (stimulated or inhibited, depending on the TCA cycle direction) with a probable consecutive imbalance in reduced cofactors. Moreover, succinate accumulation would prevent the complemented mutant from using fumarate as an alternative electron acceptor in the absence of oxygen (see discussion), which may result in elimination of the strain (Figure 7B).

## Discussion

### Global regulation of the oxygen-dependent setting up of persistence in *Brucella suis*

The RegB/A two-component system has been identified in other studies and in our previous work as a redox state sensor and a regulator of both the oxidative respiration and the denitrification pathways in *Brucella*. The sensor PrrB (RegB) of *B. abortus* (Carrica et al., 2013) and the transcriptional regulator RegA of *B. suis* were found dispensable within spleens of infected mice, whereas we discovered *B. suis* RegA as being essential for bacterial persistence within low-oxygenated livers (Abdou et al., 2013). This earlier result raised our interest in the role of RegA in setting up the persistence state of *Brucella* which has been addressed for the first time in the present work.

We have shown that survival of a *B. suis* strain devoid of RegA was strongly affected in our model creating a gradual decrease in oxygen concentration, which correlates with its behavior observed *in vivo* (Abdou et al., 2013). In addition, stable viability of the *B. suis* WT strain over a long period makes it a suitable bacterial candidate for the study of *in vitro* long-term persistence. Based on this *in vitro* model, we conducted whole-genome microarray transcription profiling of *B. suis*, in conjunction with two different proteome analyses, to identify the full RegA regulon (12% of the genes) potentially involved in the establishment of the persistence state. RegA of *B. suis* modulates expression of genes belonging to all functional groups, acting predominantly as a repressor under the conditions applied. As initially expected, RegA of *B. suis* may play a role analogous to that of its ortholog in *Rhodobacter* (Eraso et al., 2008), which makes the two-component system RegB/A a central regulatory system required for adaptation to oxygen depletion. This function may contribute to the constraint of bacterial growth, typical of chronic infection. In agreement, both transcriptome and proteome analyses evidenced the down-regulation of cell envelope biogenesis and cellular division.

**Figure 8:**
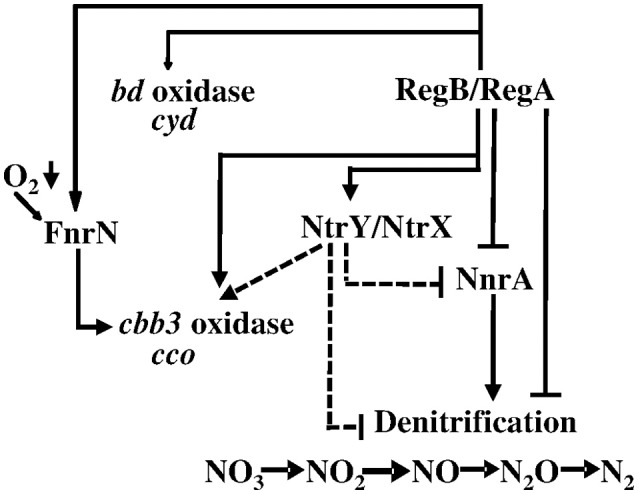
**Regulation of ***B. suis*** respiratory systems by RegA in the “***in vitro*** model of persistence.”** The dashed lines indicate a putative effect of the two-component system NtrY/NtrX on expression of the operons encoding enzymes of the denitrification pathway and the *cbb3*-type cytochrome *c* oxidase, postulated on the basis of previous knowledge of this system in *B. abortus* (Carrica et al., 2013); see the manuscript for further explanations. Positive or negative regulation is indicated.

NtrY/X, a second redox regulatory two-component system (Carrica et al., 2012), contributes with PrrB/A (RegB/A) to the complex network which controls the respiratory systems in *B. abortus* (Carrica et al., 2013). The histidine kinase NtrY is required in combination with PrrB for optimal expression of all *B. abortus* denitrification genes. Positive control of *ntrY* expression by PrrB was confirmed in our present work by the observation of its RegA-dependent induction. However, these results are not consistent with the RegA-dependent repression of the denitrification genes, unless NtrY exerts a negative effect on their expression under the conditions applied. Otherwise, their down-regulation would be independent of NtrYX in *B. suis* in this context, meaning that the consequences of RegA-dependent *ntrY* induction on bacterial physiology still remain to be elucidated. We postulate that regulation of the denitrification pathway in *Brucella* spp. is complex, as several regulators are involved, and species-specific mechanisms may exist. Accordingly, we propose a hierarchical regulation scheme of respiratory systems reflecting conditions at day 3 in the “*in vitro* model of persistence,” placing the two-component system RegB/A in a lead position (Figure 8).

In fact, RegA-dependent regulation of other two-component systems and regulatory genes involved in a broad range of bacterial processes is highly suggestive of master regulation in the adaptation of *B. suis* physiology to an anaerobic environment. Induction of the transcriptional regulator MucR represents an outstanding example of such a major role for RegA, since MucR acts mainly as a repressor of *Brucella* genes involved in very diverse functions (Caswell et al., 2013; Mirabella et al., 2013). In the late exponential growth phase, *mucR* expression was shown to be under negative control of the regulator VjbR (Mirabella et al., 2013). The RegA-dependent induction of MucR in our model was therefore not surprising, as RegA was found to repress *vjbR*.

RegA-dependent repression of the *virB* operon is in line with the assumption that the bacterium does not likely need to activate the T4SS, the central virulence determinant, for establishment of the persistent state. In addition, our model does not confront the bacterium with the acidic conditions necessary for *in vitro* activation of the *virB* operon, mimicking the intracellular signal that triggers its induction for bacterial adaptation to the host cell (Boschiroli et al., 2002). Thus, our study allowed the characterization of RegA as a novel repressor of *virB* in addition to BlxR/BabR (de Jong and Tsolis, 2012). This result suggests that oxygen limitation of the installed replicative niche may be a signal to turn off *virB* expression. Most of the RegA-controlled genes or proteins previously described as virulence factors were detected as repressed by RegA (67% cumulated, *vjbR* and *virB* genes excluded) (Table S4). For example, the negative impact on LPS biosynthesis probably mirrors the global repression of envelope biogenesis, associated with a lack of bacterial proliferation. In fact, the number of the RegA-repressed genes or proteins known as virulence factors is significant (Table S4), because their function is normally required for intracellular replication.

### Role of RegA in adaptation of metabolism

Due to the lack of a carbohydrate source in the medium used in the “*in vitro* model of persistence,” bacteria have to produce pentoses for nucleic acids synthesis, and hexoses for envelope biogenesis during the first multiplicative step. Several main components of the medium such as glycerol with lactate and glutamate can serve as substrates for gluconeogenesis (Figure 6B). The RegA-mediated repression of enzymes belonging to the corresponding pathways further leads to a reduced production of intermediates feeding amino acids biosynthesis pathways, whose numerous genes and proteins were also RegA-repressed. ABC transporters and enzymes specific of the PPC required for production of nucleic acids were also found under the negative control of RegA. Induction of a ribose ABC transporter of high affinity suggests that *B. suis*, unable to produce sufficient ribose via the PPC, may attempt to import this precursor possibly for residual biosynthetic needs, such as DNA repair (see results) and/or specific RNA synthesis for adaptation. The RegA-dependent repression of RNA polymerase subunits and of genes involved in DNA replication and transcription is compatible with their residual activity in the WT strain (GEO accession number GSE87538). During persistence, the PPC may become less central in *B. suis* physiology, as restricted gluconeogenesis provides less PPC substrates, and potential use of the DHAP-pathway produces glycolysis intermediates (Figure 6B). In *B. suis*, glycerol may represent an important carbon source to partially support the energy metabolism needed for bacterial survival under conditions of persistence, despite RegA-dependent down-regulation of the downstream genes and proteins (Figure 6B). Although a glycerol uptake facilitator protein was not identified in *Brucella*, growth of *B. abortus* B19 with glycerol as the main carbon supply suggested that at least this strain uses a common transporter system for erythritol and glycerol (Sangari et al., 1996). The present results strongly suggested that *B. suis* underwent a RegA-controlled slowdown of gluconeogenesis and of amino acids and nucleic acids synthesis, which is consistent with growth arrest of bacteria entering persistence.

Induction of *fumB* highlights the hypothesis that *B. suis* may use fumarate as an electron acceptor under anoxia, itself converted to succinate by succinate dehydrogenase (Al Dahouk et al., 2009) (Figure 6B). Our previous proteome data already suggested that *B. suis* may use the reductive branch of the TCA cycle under anaerobiosis (Al Dahouk et al., 2009). *B. suis* possesses dehydrogenases specific for glycerol-3-P (BR0200, *glpD*), L-lactate and NADH, efficient electron donors for fumarate reduction in *E. coli* (Gennis and Valley, 1996), the latter two were however repressed by RegA (see results). While anaerobiosis becomes established, use of oxygen via a terminal cytochrome *c* oxidase may be shifting to the use of fumarate as an alternative electron acceptor, adapted to low energetic needs. We suggest that decreased expression of *aceA* in the WT strain may be related to increased expression of *fumB*, as a mean to limit succinate accumulation (Figure 6B) and, under our experimental conditions, may also be explained by the lack of fatty acids in the medium.

A functional ICL presented an advantage for persistence *in vitro* in the presence of fatty acids, but not for aerobic growth, leading to the hypothesis that *B. suis* might be able to use them under these specific conditions. Demonstrating a strict need of ICL for both the acute and chronic phases of infection, *B. suis* behaves similarly to other intracellular bacterial species, since alteration of both *M. tuberculosis icl1* and *icl2* genes prevented growth during the first stage of infection (Muñoz-Elías and McKinney, 2005; Blumenthal et al., 2010) and persistence during the chronic phase in mice (McKinney et al., 2000; Blumenthal et al., 2010). At the chronic stage, ICL together with enzymes of fatty acids degradation were induced in lungs (Schnappinger et al., 2003), ICL activity being essential for use of fatty acids as carbon source (Muñoz-Elías and McKinney, 2005). However, *Salmonella enterica* serovar Typhimurium required its unique *aceA* gene to establish persistent, but not acute infection *in vivo* (Fang et al., 2005).

Despite the increased production of ICL in intracellular *B. suis* (Al Dahouk et al., 2008), the *aceA* mutant was not attenuated in the same model of infection, possibly due to less drastic hypoxia in cell cultures than *in vivo* (Atkuri and Herzenberg, 2005). The crucial role of ICL for *B. suis* multiplication in the low-oxygenated environment of host organs gave further indications that fatty acids may act as a possible source of energy for *B. suis*. A dual function of *B. suis* ICL and its participation as a major virulence factor is also conceivable, as *P. aeruginosa* ICL has a positive effect on type III secretion system expression in oxygen-limited cultures (Chung et al., 2016). The enzyme of *P. aeruginosa* has also been identified as a virulence factor in a rat model of chronic lung infections (Lindsey et al., 2008). Surprisingly, the role of ICL seems to markedly differ in *B. suis* and *B. abortus*, as the enzyme is dispensable in the latter for virulence and persistence (Zúñiga-Ripa et al., 2014). These findings can be correlated to the fact that ICL was not regulated during *in vitro* infection with *B. abortus* (Lamontagne et al., 2009), in contrast to *B. suis* (Al Dahouk et al., 2008). Moreover, their respective *cydB* mutants devoid of cytochrome *bd* oxidase with high affinity for oxygen displayed opposite fates in mice (Endley et al., 2001; Jiménez de Bagüés et al., 2007). Important variations in metabolic routes may exist among *Brucella* strains, which may contribute to their different phenotypes at the chronic stage of spleen infection in mice (Bosseray et al., 1982).

The present study has shed light on the central role of RegA in control of metabolic pathways involved in establishment of bacterial persistence. Activation of two-component systems that control regulons involved in the setting-up of a low oxygen-dependent non-replicative state is likely to be a widespread mechanism among aerobic human pathogens. The RoxS/R regulon, the homolog of RegB/A in *P. aeruginosa* (Elsen et al., 2004), includes genes necessary for respiratory function and maintenance of the redox balance, and for sugar and amino acid metabolism (Kawakami et al., 2010). The *M. tuberculosis* DosST/R regulon (Voskuil et al., 2003) is expressed under hypoxia and during *in vitro* dormancy established by bacterial adaptation to the gradual limitation of oxygen obtained with the “Wayne model” (Wayne and Sohaskey, 2001). This model was the most extensively used to study the dormant state of tubercle bacilli and was developed further, resulting in the application of our “*in vitro* model of persistence” to *B. suis*.

Another trait common to these pathogens is the need of ICL for infection. Therefore, our work provides a solid base allowing further studies of the regulation and of the role of selected genes such as *aceA* in adaptation of *B. suis* to the specific environment associated with persistence. The observed patterns of RegA-dependent regulation of several metabolic pathways could be specific of the time point chosen in our “*in vitro* model of persistence,” which is a dynamic model (see results). Expected results should provide a better understanding of the common mechanisms involved in setting-up a persistence state, typical of the chronic phase of infection.

Whereas ICL has been considered as a potential target for drugs active against *M. tuberculosis* (Sharma et al., 2013), clinical isolates of *P. aeruginosa* and other gram-negative pathogens (Fahnoe et al., 2012), this will not be applicable to all *Brucella* species of clinical relevance. Finally, similar to a possible alternative approach for the design of anti-tubercular molecules (Banerjee et al., 2016), targeting of the response regulator RegA for the development of a new therapeutic strategy interfering with the establishment of persistent infection would be of great interest.

## Author contributions

EA, VJ-M, MJdB, IM-A, and SO-B carried out the experiments. VP performed the microarray processing and the associated statistical analysis. VJ-M and EA conceived the study and achieved the interpretation of the data. SK, SAD, and AO contributed to conception of the study. EA and VJ-M were involved in drafting the manuscript and, SK, VJ-M, AO, and SAD revised it. All authors read and approved the final manuscript.

## Funding

This work was supported by funds from the Centre National de la Recherche Scientifique (CNRS), France, the National Institute for Agricultural and Food Research and Technology (INIA) in Spain (Grant RTA 2013-00065-C02-01), and by grant 1329-485 from the Bundesinstitut für Risikobewertung (BfR), Germany. EA was supported by a fellowship from the Lebanese CNRS and IM-A received funds from Infectiopôle Sud.

### Conflict of interest statement

The authors declare that the research was conducted in the absence of any commercial or financial relationships that could be construed as a potential conflict of interest.
