# Supplementary material for: RegA Plays a Key Role in Oxygen-Dependent Establishment of Persistence and in Isocitrate Lyase Activity, a Critical Determinant of In vivo Brucella suis Pathogenicity
Source: Front Cell Infect Microbiol. 2017 May 18;7:186. doi: 10.3389/fcimb.2017.00186 (PMC5435760; doi:10.3389/fcimb.2017.00186)
Supplement: Supplementary file 1 [file Table1.PDF]

S1 Table: RegA-dependent genes in *B. suis* identified with the *in vitro* model of persistence. Up-regulation (white) and down-regulation (grey) in the wild-type strain, as determined by transcriptome analysis.

COG group	Gene ID	Gene Name	Function	Fold[a)] WT/Δ*regA*
**Translation, ribosomal structure and biogenesis (J)**	BR1119	*nifR3*	nitrogen regulation protein Nifr3	2.41
	BR1914	*trmD*	tRNA (guanine-N1)-methyltransferase	2.23
	BR2166	*rbfA*	ribosome-binding factor A	2.17
	BR2167	*truB*	tRNA pseudouridine synthase B	2.72
	BRA0091		acetyltransferase, GNAT family	2.31
	BRA0202	*ileS*	isoleucyl-tRNA synthetase	0.45
	BRA0751		endoribonuclease L-PSP, putative	0.45
**Transcription (K)**	BR0060		transcriptional regulator, LysR family	2.05
	BR0290		transcriptional regulator, TetR family	2.35
	BR0324		transcriptional regulator, LysR family	2.47
	BR0549		transcriptional regulator, LacI family	2.49
	BR0550		transcriptional regulator, AraC family	5.15
	BR0604	*feuP*	DNA-binding response regulator	3.73
	BR0654	*fnrN*	transcriptional regulator, Crp/Fnr family	7.66
	BR0938		ATP-dependent RNA helicase, DEAD/DEAH box family	2.17
	BR1100	*gntR5* *phnF*	transcriptional regulator, GntR family phosphonate metabolism transcriptional regulator	2.23
	BR1187		transcriptional regulator, Cro/CI family	3.48
	BR1319		transcriptional regulator, AraC family	2.66
	BR1451		transcriptional regulator, ArsR family	3.64
	BR1572		transcriptional regulator, MarR family	2.23
	BR1613		transcriptional regulator, Cro/CI family	2.46
	BR1951		transcriptional regulator, LysR family	2.59
	BRA0023		transcriptional regulator, GntR family	2.55
	BRA0302		sugar-binding protein, putative	2.31
	BRA0414		transcriptional regulator, Crp/Fnr family	2.57
	BRA0451		transcriptional regulator, AraC family	2.41
	BRA0480		transcriptional regulator, MarR family	2.1
	BRA0634		transcriptional regulator, AsnC family	2.25
	BRA0709		transcriptional regulator OxyR, putative	2.71
	BRA0813	*gntR1*	transcriptional regulator, GntR family	2.07
	BRA0913		transcriptional regulator, GntR family	2.15

	BRA0984		transcriptional regulator, putative	2.23
	BRA0997		transcriptional regulator, IclR family	2.83
	BRA1015		transcriptional regulator, GntR family	2.57
	BRA1169		transcriptional regulator, IclR family	4.1
	BR0872	*exoR*	exopolysacchride production negative regulator exoR	0.43
	BR1242	*rpoC*	DNA-directed RNA polymerase, beta' subunit	0.37
	BR2169	*pnp*	polyribonucleotide nucleotidyltransferase	0.46
	BRA0098		transcriptional regulator, LysR family	0.44
	BRA0119	*vjbR*	transcriptional regulator, LuxR family	0.21
	BRA0262	*nnrA*	transcriptional regulator, Crp/Fnr family	0.44
	BR1492		cold-shock family protein	0.28
	BR1514		cold-shock family protein	0.35
**DNA replication, recombination and repair (L)**	BR0256		site-specific recombinase, phage integrase family	2.09
	BR0260		site-specific recombinase, resolvase family	2.02
	BR0514		transposase, interruption-C	2.1
	BR1474		site-specific recombinase, phage integrase family	2.28
	BRA0555		IS66 family element, orf4, putative	2.49
	BRA0556		IS66 family element, orf3, putative	3.6
	BRA0557		IS66 family element, orf2, putative	2.4
	BRA0558		IS66 family element, orf1, transposase, putative	3.99
	BRA0791		Micrococcal nuclease homologs	2.04
	BR0001	*dnaA*	chromosomal replication initiator protein DnaA	0.42
	BR0491	*babI*	modification methylase BabI	0.48
	BR1105		DNA-binding protein HU	0.34
**defense mechanisms (V)**	BRA0840	*hsdM*	type I restriction-modification system, M subunit	2.02
	BRA0947		D-aminopeptidase	2.02
	BR1060		HlyD family secretion protein	0.47
	BRA0334		hydrophobe/amphiphile efflux-1 family protein	0.44
	BRA0914		AcrB/AcrD/AcrF multidrug efflux protein	0.4
**Cell division and chromosome partitioning (D)**	BR1432	*ftsW*	cell division protein FtsW	0.42
	BR1895		cell division protein FtsK	0.46
	BRA0530		cell division protein FtsK, putative	0.32
	BR0096	*ccmC*	heme exporter protein CcmC	2.05
	BR0607		cytochrome c-type biogenesis protein, putative	7.83

**Posttranslational modification, protein turnover, chaperones (O)**	BR0608	*ccmE*	cytochrome c-type biogenesis protein CcmE	2.93
	BR0905	*aat*	leucyl/phenylalanyl-tRNA--protein transferase	2.28
	BR2132		Mg chelatase-related protein	3.78
	BR0366		twin-arginine translocation signal domain protein	0.43
	BR0626		glutathione S-transferase family protein	0.36
	BRA0289		protease, putative	0.47
	BRA0290		peptidase, U32 family	0.49
	BRA0295		peptidyl-prolyl cis-trans isomerase, putative	0.33
**Cell envelope biogenesis, outer membrane (M)**	BR0637	*omp2a*	porin Omp2a	2.03
	BR1486		membrane protein, putative	2.46
	BR1508		membrane protein, putative	2.16
	BR1671	*macA*	HlyD family secretion protein	2.53
	BR2158	*int*	apolipoprotein N-acyltransferase	2.35
	BRA0421		glycosyl transferase, group 1 family protein	2.3
	BRA0428		undecaprenyl-phosphate alpha-N-acetylglucosaminyltransferase, putative	2.04
	BRA0590		membrane protein, putative	2.84
	BR0117		penicillin-binding protein, 1A family	0.48
	BR0225		ABC transporter, periplasmic substrate-binding protein, putative	0.47
	BR0518	*wbkB*	WbkB protein	0.31
	BR0521		perosamine synthase, putative	0.35
	BR0522	*gmd*	GDP-mannose 4,6-dehydratase	0.42
	BR0643		transglycosylase SLT domain protein	0.35
	BR0685		organic solvent tolerance, putative	0.4
	BR0888		peptidase, M23/M37 family	0.43
	BR0916		penicillin-binding protein 1A, putative	0.38
	BR0981		glycosyl transferase WboA	0.46
	BR0982	*wbdA*	glycosyl transferase, group 1 family protein	0.4
	BR0990		rare lipoprotein A family protein	0.35
	BR1172		D-alanyl-D-alanine carboxypeptidase	0.24
	BR1204		OmpA family protein	0.48
	BR1271	*ddlA*	D-alanine--D-alanine ligase A	0.43
	BR1284		membrane protein, putative	0.15
	BR1433	*murD*	UDP-N-acetylmuramoylalanine--D-glutamate ligase	0.45
	BR1434	*mraY*	phospho-N-acetylmuramoyl-pentapeptide-transferase	0.37

	BR1435	*murF*	UDP-N-acetylmuramoylalanyl-D-glutamyl-2,6-diaminopimelate--D-alanyl-D-alanyl ligase	0.39
	BR1436	*murE*	UDP-N-acetylmuramoylalanyl-D-glutamate--2,6-diaminopimelate ligase	0.46
	BR1437		penicillin-binding protein	0.44
	BR1468		choloylglycine hydrolase family protein	0.44
	BR1475	*omp28*	immunoreactive 28 kDa outer membrane protein	0.3
	BR1622	*omp31*	outer membrane protein Omp31	0.4
	BR1714		OmpA family protein	0.5
	BRA0135		glycosyl transferase, group 2 family protein	0.44
	BRA0487		glycosyl hydrolase, family 25	0.42
	BRA0921		outer surface protein	0.32
**Cell motility and secretion (N)**	BRA0159		flagellar protein FliL	2.21
	BRA0151	*flgB*	flagellar basal-body rod protein FlgB	0.4
**Inorganic ion transport and metabolism (P)**	BR0498		cobalt-zinc-cadmium resistance protein	2.45
	BR1099	*phnN*	phosphonate metabolism protein PhnN	2.08
	BR1344		iron compound ABC transporter, ATP-binding protein	2.11
	BR1347		iron compound TonB-dependent receptor, putative	2.28
	BRA0089	*modB*	molybdenum ABC transporter, permease protein	2.13
	BRA0116		drug resistance transporter, Bcr/CflA family	2.32
	BRA0468		ABC transporter, ATP-binding protein	2.08
	BRA0469		ABC transporter, periplasmic substrate-binding protein	2.39
	BRA0677		iron compound ABC transporter, permease protein	2.74
	BRA0678		iron compound ABC transporter, ATP-binding protein	2.42
	BR0220		copper-translocating P-type ATPase	0.42
	BR0748	*ppk*	polyphosphate kinase	0.45
	BR1364		lipoprotein, putative	0.42
	BR1365	*cbiM*	cobalamin biosynthesis protein CbiM	0.5
	BR2139	*pstC*	phosphate ABC transporter, permease protein	0.49
	BR2140		phosphate ABC transporter, permease protein	0.43
	BRA0283		membrane protein, putative	0.36
	BRA0703	*sodC*	superoxide dismutase, Cu-Zn	0.46

	BRA1190		tonB dependent receptor domain protein	0.34
**Signal transduction mechanisms (T)**	BR0605	*feuQ*	sensor histidine kinase	3.72
	BR1118	*ntrB*	nitrogen regulation protein NtrB	2.44
	BR1659		sensor histidine kinase	2.05
	BRA0301		cyclic nucleotide-binding protein	2.15
	BR0133	*regB*	sensor histidine kinase	0.41
	BR0577		sensor histidine kinase	0.35
	BR1036	*dksA*	dnaK suppressor protein	0.48
	BRA1055		universal stress protein family	0.29
**Energy production and conversion (C)**	BR0039		cytochrome c, membrane-bound	3.35
	BR0467	*coxB*	cytochrome c oxidase, subunit II	7.91
	BR0468	*coxA*	cytochrome c oxidase, subunit I	2.43
	BR0961	*fumB*	fumarate hydratase, class I	5.06
	BR1462		NADH oxidoreductase, putative	2.31
	BR1541		ubiquinol-cytochrome c reductase, cytochrome c1, putative	2.87
	BR1542	*petB*	ubiquinol-cytochrome c reductase, cytochrome b	4.84
	BR1543		ubiquinol-cytochrome c reductase, iron-sulfur subunit	5.75
	BRA0391		alcohol dehydrogenase, zinc-containing	2.36
	BRA0508	*cydD*	ABC transporter, ATP-binding protein CydD	6.77
	BRA0509	*cydC*	ABC transporter, permease/ATP-binding protein	2.55
	BRA1014		aldehyde dehydrogenase family protein	2.58
	BR0808	*nuoG*	NADH dehydrogenase I, G subunit	0.43
	BR0809	*nuoH*	NADH dehydrogenase I, H subunit	0.33
	BR0810	*nuoI*	NADH dehydrogenase I, I subunit	0.33
	BR0811	*nuoJ*	NADH dehydrogenase I, J subunit	0.41
	BR0812	*nuoK*	NADH dehydrogenase I, K subunit	0.37
	BR0813	*nuoL*	Putative identification NADH dehydrogenase I, L subunit	0.25
	BR0814	*nuoM*	NADH dehydrogenase I, M subunit	0.22
	BR0815	*nuoN*	NADH dehydrogenase I, N subunit	0.25
	BR1017	*maeB*	NADP-dependent malic enzyme	0.47
	BR1126	*lpdA-1*	pyruvate dehydrogenase complex, E3 component, lipoamide dehydrogenase	0.36
	BR1127	*aceF*	pyruvate dehydrogenase complex, E2 component, dihydrolipoamide acetyltransferase	0.32

	BR1128	*pdhB*	pyruvate dehydrogenase complex, E1 component, beta subunit	0.36
	BR1148	*gltA*	citrate synthase	0.3
	BR1614	*aceA*	isocitrate lyase	0.04
	BR1648	*glcB*	malate synthase G	0.5
	BR1728	*gap*	glyceraldehyde 3-phosphate dehydrogenase	0.34
	BR1729	*pgk*	phosphoglycerate kinase	0.38
	BR1781	*pyc*	pyruvate carboxylase	0.45
	BR1922	*sucB*	2-oxoglutarate dehydrogenase, E2 component, dihydrolipoamide succinyltransferase	0.41
	BR1923	*sucA*	2-oxoglutarate dehydrogenase, E1 component	0.41
	BRA0166		CAIB/BAIF family protein	0.45
	BRA0170		cytochrome b561, putative	0.28
	BRA0174	*cycA*	cytochrome c2	0.3
	BRA0246	*norE*	cytochrome c oxidase, subunit III	0.29
	BRA0247	*norF*	norF protein	0.36
	BRA0248	*norC*	nitric-oxide reductase, small subunit	0.14
	BRA0249	*norB*	nitric-oxide reductase, large subunit	0.17
	BRA0260	*nirK*	copper-containing nitrite reductase	0.4
	BRA0261	*nirV*	NirV protein	0.19
	BRA0274	*nosR*	transcriptional regulator NosR, putative	0.45
	BRA0275	*nosZ*	nitrous-oxide reductase	0.38
	BRA0296	*narI*	respiratory nitrate reductase, gamma subunit	0.34
	BRA0297	*narJ*	respiratory nitrate reductase, delta subunit	0.35
	BRA0298	*narH*	respiratory nitrate reductase, beta subunit	0.41
	BRA0299	*narG*	respiratory nitrate reductase, alpha subunit	0.34
	BRA0571	*qor*	quinone oxidoreductase	0.48
	BRA0919		oxidoreductase, molybdopterin-binding, putative	0.45
**carbohydrate transport and metabolism (G)**	BR0235		sugar ABC transporter, periplasmic sugar-binding protein	2.14
	BR0236		sugar ABC transporter, permease protein	2.27
	BRA0265		sugar ABC transporter, periplasmic sugar-binding protein, putative	3.75
	BRA0305		sugar ABC transporter, permease protein	2.31

	BRA0435		epimerase/dehydratase family protein, putative	2.2
	BRA0995	*rbsA-4*	ribose ABC transporter, ATP-binding protein	2.9
	BRA0996	*rbsB-3*	ribose ABC transporter, periplasmic D-ribose-binding protein	2.23
	BRA1156		2,4-dihydroxyhept-2-ene-1,7-dioic acid aldolase, putative	2.22
	BR0111		cyclic beta 1-2 glucan synthetase	0.46
	BR0519	*rfbE*	O-antigen export system ATP-binding protein RfbE	0.44
	BR0520	*rfbD*	O-antigen export system permease protein RfbD	0.31
	BRA0143		phthalate transporter, putative	0.41
	BRA0385	*xfp*	xylulose-5-phosphate/fructose-6-phosphate phosphoketolase	0.3
	BRA0655	*ugpB*	glycerol-3-phosphate ABC transporter, periplasmic glycerol-3-P-binding protein	0.41
	BRA0656	*ugpA*	glycerol-3-phosphate ABC transporter, permease protein	0.22
	BRA0657	*ugpE*	glycerol-3-phosphate ABC transporter, permease protein	0.22
	BRA0658	*ugpC*	glycerol-3-phosphate ABC transporter, ATP-binding protein	0.44
	BRA1180		sugar ABC transporter, periplasmic sugar-binding protein, putative	0.42
**amino acid transport and metabolism (E)**	BR0279		N-carbamyl-L-amino acid amidohydrolase, putative	2.2
	BR0283		pyridine nucleotide-disulphide oxidoreductase family protein	2.2
	BR1314	*mmsB*	3-hydroxyisobutyrate dehydrogenase	2.01
	BR1825		chorismate mutase	2.32
	BR1953		amino acid ABC transporter, periplasmic amino acid-binding protein	2.15
	BR1955		amino acid ABC transporter, permease protein	2.08
	BR1960		L-asparaginase type II, putative	2.2
	BR1965		methionine-gamma-lyase, putative	2.01
	BRA0028		branched-chain amino acid ABC transporter, ATP-binding protein	2.58
	BRA0034		shikimate dehydrogenase family protein	2.34
	BRA0651		branched-chain amino acid ABC transporter, ATP-binding protein	2.2
	BRA0784		peptide ABC transporter, permease protein	2.22
	BRA0948		amino acid ABC transporter, periplasmic amino acid-binding protein	2.09

	BRA0953		branched-chain amino acid ABC transporter, periplasmic amino acid-binding protein, putative	2.2
	BRA1090		peptide ABC transporter, periplasmic peptide-binding protein	2.08
	BRA1161		5-carboxy-2-hydroxymuconate semialdehyde dehydrogenase	2
	BRA1162	*hpcD*	5-carboxymethyl-2-hydroxymuconate delta isomerase	2.14
	BRA1174		branched-chain amino acid ABC transporter, permease protein	2.25
	BRA1175		branched chain amino acid ABC transporter, permease/ATP-binding protein	2.3
	BRA1176		branched-chain amino acid ABC transporter, ATP-binding protein	2.13
	BR0009		ABC transporter, periplasmic substrate-binding protein, putative	0.26
	BR0010		ABC transporter, periplasmic substrate-binding protein, putative	0.34
	BR0617	*pepN*	aminopeptidase N	0.25
	BR0635		histidinol-phosphate aminotransferase, putative	0.47
	BR0739	*metC*	cystathionine beta-lyase	0.49
	BR0765	*glyA*	serine hydroxymethyltransferase	0.49
	BR1358	*ureC-2*	urease, alpha subunit	0.47
	BR1359	*ureE-2*	urease accessory protein UreE, putative	0.38
	BR1361	*ureG-2*	urease accessory protein UreG	0.47
	BR1362	*ureD-2*	urease accessory protein UreD	0.46
	BR1388	*ilvH*	acetolactate synthase, small subunit	0.48
	BR1389	*ilvB*	acetolactate synthase, catalytic subunit	0.49
	BR1920		transporter, LysE family	0.48
	BR2181		cytosol aminopeptidase family protein	0.48
	BRA0056		amino acid permease family protein	0.22
	BRA0081	*pepF*	oligoendopeptidase F	0.29
	BRA0406		dihydrodipicolinate synthase family protein	0.46
	BRA0535		oligopeptide ABC transporter, permease protein	0.2
	BRA0536		oligopeptide ABC transporter, permease protein	0.25
	BRA0537		oligopeptide ABC transporter, periplasmic oligopeptide-binding protein	0.06

	BRA0538		oligopeptide ABC transporter, periplasmic oligopeptide-binding protein	0.11
	BRA0576		peptide ABC transporter, periplasmic peptide-binding protein	0.31
	BRA0774		AzlC family protein	0.42
	BRA0878	*dcp*	peptidyl-dipeptidase Dcp	0.41
	BRA0908		aldehyde dehydrogenase family protein	0.5
**nucleotide transport and metabolism (F)**	BR0278	*dhT*	D-hydantoinase	2.6
	BR0672		MutT/nudix family protein	2.31
	BRA0607		MutT/nudix family protein	2.2
	BR1043		ribonucleotide reductase subunit alpha	0.35
	BRA0006	*iunH*	inosine-uridine preferring nucleoside hydrolase	0.34
**coenzyme metabolism (H)**	BR0655	*hemN-1*	oxygen-independent coproporphyrinogen III oxidase	2
	BR0757	*hemB*	delta-aminolevulinic acid dehydratase	3.64
	BR1299	*cobK*	precorrin-6x reductase	2.19
	BR1723		5-formyltetrahydrofolate cyclo-ligase family protein	2.37
	BR1756		thiamine pyrophosphokinase	2.29
	BRA0201	*ribF*	riboflavin biosynthesis protein RibF	2.08
	BRA1108		glutamate-1-semialdehyde 2,1-aminomutase, putative	3
	BR0960		monooxygenase, FAD-binding	0.27
	BRA0489	*bioA*	adenosylmethionine-8-amino-7-oxononanoate aminotransferase	0.4
	BRA0926		beta alanine-pyruvate transaminase	0.43
	BRA1199	*hemN-2*	oxygen-independent coproporphyrinogen III oxidase	0.22
**Lipid metabolism (I)**	BRA1086		3-hydroxyacyl-CoA dehydrogenase family protein	2.05
	BR0372	*bacA*	bacteroid development protein BacA	0.47
	BR0740		PAP2 family protein	0.4
	BRA0131		PAP2 family protein	0.44
	BRA0488		3-oxoacyl-(acyl-carrier-protein) synthase III, putative	0.44
**secondary metabolites biosynthesis, transport and catabolism (Q)**	BR0097		heme exporter protein D	2.62
	BR0700		oxidoreductase, short chain dehydrogenase/reductase family	2.16
	BRA0012		enterobactin synthetase, component D, putative	2.46
	BRA0388		heme-thiolate monooxygenase, putative	0.34
**Intracellular trafficking, secretion,**	BR1826	*ffh*	signal recognition particle protein	2.32
	BRA0059	*virB11*	type IV secretion system protein VirB11	0.43
	BRA0060	*virB10*	type IV secretion system protein VirB10	0.43

and vesicular transport (U)	BRA0064	*virB6*	type IV secretion system protein VirB6	0.38
	BRA0065	*virB5*	type IV secretion system protein VirB5	0.36
	BRA0066	*virB4*	type IV secretion system protein VirB4	0.4
	BRA0067	*virB3*	type IV secretion system protein VirB3	0.24
	BRA0068	*virB2*	type IV secretion system protein VirB2	0.19
	BRA0069	*virB1*	type IV secretion system protein VirB1	0.23
General function prediction only (R)	BR0129		acetyltransferase, GNAT family	2.19
	BRA0711	*idhA*	myo-inositol 2-dehydrogenase	2.15
	BRA1089		penicillin amidase family protein	2.62
	BR0007		ABC transporter, permease protein	0.36
	BR1197		ThiJ/PfpI family protein	0.49
	BR1948		membrane protein, putative	0.23
	BRA0010		lipoprotein, Bmp family	0.38
	BRA0099		amidase	0.4
	BRA0250		protein phosphatase, putative	0.16
	BRA0534		ABC transporter, ATP-binding protein	0.16
Function unknown (S)	BR0040		conserved hypothetical protein	2.18
	BR0052		hypothetical protein	2.46
	BR0139		lipoprotein, putative	2.23
	BR0189		hypothetical protein	2.32
	BR0242		hypothetical protein	2.05
	BR0258		BRO family protein	2.14
	BR0293		hypothetical protein	2.53
	BR0294		hypothetical protein	2.71
	BR0322		conserved hypothetical protein	2.32
	BR0333		hypothetical protein	2.06
	BR0392		hypothetical protein	2.51
	BR0393		hypothetical protein	2.18
	BR0555		conserved hypothetical protein	2.32
	BR0584		hypothetical protein	2.86
	BR0590		conserved hypothetical protein	2.23
	BR0592		hypothetical protein	2.06
	BR0624		hypothetical protein	3.34
	BR0625		hypothetical protein	2.51
	BR0711		hypothetical protein	2.49
	BR0818		glyoxalase family protein	2.9
	BR0821		hypothetical protein	8.2
	BR0869		conserved hypothetical protein	2.02

	BR0921		hypothetical protein	2.17
	BR0968		hypothetical protein	2.31
	BR0972		conserved hypothetical protein	2.09
	BR0979		hypothetical protein	2.01
	BR1073		conserved hypothetical protein	2.41
	BR1089		conserved hypothetical protein	2.14
	BR1176		hypothetical protein	2.58
	BR1253		hypothetical protein	2.51
	BR1321		conserved hypothetical protein	2.97
	BR1405		hypothetical protein	2.42
	BR1406		conserved hypothetical protein	2.8
	BR1407		conserved hypothetical protein	3
	BR1466		hypothetical protein	2.18
	BR1467		hypothetical protein	2.21
	BR1471		hypothetical protein	3.69
	BR1472		hypothetical protein	2.01
	BR1477		conserved hypothetical protein	2.19
	BR1501		hypothetical protein	2.42
	BR1665		hypothetical protein	2.56
	BR1794		hypothetical protein	3.85
	BR1797		hypothetical protein	2.63
	BR1839		hypothetical protein	2.38
	BR1840		conserved hypothetical protein	2.71
	BR1952		hypothetical protein	3.13
	BR1979		YaiI/YqxD family protein	2.1
	BR2096		hypothetical protein	2.71
	BR2153		conserved hypothetical protein	2.34
	BR2176		conserved hypothetical protein	2.13
	BRA0053		hypothetical protein	2.1
	BRA0264		hypothetical protein	3.55
	BRA0303		hypothetical protein	2.11
	BRA0369		hypothetical protein	3.32
	BRA0413		hypothetical protein	2.58
	BRA0436		conserved hypothetical protein	2.21
	BRA0452		hypothetical protein	2.45
	BRA0507		conserved hypothetical protein	7.43
	BRA0561		conserved hypothetical protein	2.63
	BRA0564		conserved hypothetical protein	9.62

	BRA0688		hypothetical protein	2.26
	BRA0772		hypothetical protein	2.28
	BRA0827		hypothetical protein	2.35
	BRA0830		hypothetical protein	2.64
	BRA0834		hypothetical protein	3.19
	BRA0835		hypothetical protein	3.55
	BRA0836		hypothetical protein	3.12
	BRA0837		hypothetical protein	3.05
	BRA0839		conserved hypothetical protein	2.89
	BRA0863		hypothetical protein	2
	BRA1036		conserved hypothetical protein	2.46
	BRA1141		conserved hypothetical protein	2.94
	BRA1168		conserved hypothetical protein	2.04
	BR0049		conserved hypothetical protein	0.43
	BR0080		conserved hypothetical protein	0.48
	BR0106		conserved hypothetical protein	0.38
	BR0187		conserved hypothetical protein	0.49
	BR0317		hypothetical protein	0.32
	BR0346		conserved hypothetical protein	0.45
	BR0378		conserved hypothetical protein	0.46
	BR0434		conserved hypothetical protein	0.48
	BR0447		hypothetical protein	0.47
	BR0564		lipoprotein, putative	0.37
	BR0580		conserved hypothetical protein	0.43
	BR0644		hypothetical protein	0.41
	BR0686		conserved hypothetical protein	0.49
	BR0738		hypothetical protein	0.34
	BR1024		conserved hypothetical protein	0.36
	BR1125		hypothetical protein	0.46
	BR1130		conserved hypothetical protein	0.28
	BR1163		conserved hypothetical protein	0.24
	BR1182		conserved hypothetical protein	0.42
	BR1191		conserved hypothetical protein	0.46
	BR1205		lipoprotein, putative	0.32
	BR1241		conserved hypothetical protein	0.4
	BR1423		hypothetical protein	0.46
	BR1518		hypothetical protein	0.49
	BR1525		conserved hypothetical protein	0.4

	BR1558		hypothetical membrane protein, putative	0.5
	BR1559		conserved hypothetical protein	0.5
	BR1593		conserved hypothetical protein	0.27
	BR1596		conserved hypothetical protein	0.36
	BR1600		conserved hypothetical protein	0.31
	BR1615		hypothetical protein	0.07
	BR1616		hypothetical protein	0.05
	BR1617		hypothetical protein	0.05
	BR1621		conserved hypothetical protein	0.5
	BR1624		hypothetical protein	0.48
	BR1634		hypothetical protein	0.15
	BR1635		hypothetical protein	0.18
	BR1660		hypothetical protein	0.46
	BR1713		conserved hypothetical protein	0.49
	BR1742		hypothetical protein	0.45
	BR1752		conserved hypothetical protein	0.46
	BR1774		conserved hypothetical protein	0.44
	BR1819		conserved hypothetical protein	0.44
	BR1921		conserved hypothetical protein	0.49
	BR1938		conserved hypothetical protein	0.41
	BR2016		conserved hypothetical protein	0.46
	BR2041		conserved hypothetical protein	0.39
	BRA0035		hypothetical protein	0.38
	BRA0036		hypothetical protein	0.42
	BRA0048		hypothetical protein	0.44
	BRA0120		conserved hypothetical protein	0.5
	BRA0133		hypothetical protein	0.41
	BRA0167		conserved hypothetical protein	0.46
	BRA0184		conserved hypothetical protein	0.21
	BRA0244		conserved hypothetical protein	0.32
	BRA0245		conserved hypothetical protein	0.25
	BRA0252		conserved hypothetical protein	0.12
	BRA0253		conserved hypothetical protein	0.18
	BRA0254		conserved hypothetical protein	0.29
	BRA0255		conserved hypothetical protein	0.29
	BRA0331		hypothetical protein	0.27
	BRA0332		hypothetical protein	0.23
	BRA0333		hypothetical protein	0.49

	BRA0336		conserved hypothetical protein	0.36
	BRA0365		hypothetical protein	0.44
	BRA0434		hypothetical protein	0.35
	BRA0585		membrane protein, putative	0.32
	BRA0586		conserved domain protein	0.5
	BRA0775		conserved hypothetical protein	0.49
	BRA0787		conserved hypothetical protein	0.5
	BRA0797		hypothetical protein	0.46
	BRA0897		hypothetical protein	0.39
	BRA0999		conserved hypothetical protein	0.3
	BRA1019		conserved hypothetical protein	0.49
	BRA1043		hypothetical protein	0.5
	BRA1056		hypothetical protein	0.5
	BRA1061		conserved hypothetical protein	0.44
	BRA1099		hypothetical protein	0.5
	BRA1164		hypothetical protein	0.32
	BRA1191		hypothetical protein	0.37

[a]Fold WT/Δ*regA*: fold change is expressed as the ratio of wild-type/Δ*regA* hybridization levels
