# Supplementary material for: RegA Plays a Key Role in Oxygen-Dependent Establishment of Persistence and in Isocitrate Lyase Activity, a Critical Determinant of In vivo Brucella suis Pathogenicity
Source: Front Cell Infect Microbiol. 2017 May 18;7:186. doi: 10.3389/fcimb.2017.00186 (PMC5435760; doi:10.3389/fcimb.2017.00186)
Supplement: Supplementary file 2 [file Table2.PDF]

S2 Table: *B. suis* proteins regulated by RegA during the establishment of persistence. RegA-controlled proteins in the wild-type strain were identified by 2D-DIGE and ICPL analyses combined with mass spectrometry.

ORF Number	Accession Number	Protein Name	Ratio WT/*regA* 2D-DIGE[1)]	T-Test 2D-DIGE	Ratio WT/*regA* ICPL	T-Test ICPL	Ratio T[2)] WT/*regA*
**Transcription (K)**							
BR0569	gi|23501458|ref|NP_697585.1|	Ros/MucR family transcriptional regulator			1.54	1.26E-02	
BR0651	gi|23501538|ref|NP_697665.1|	DNA-directed RNA polymerase subunit omega			1.85	2.80E-04	
BR1242	gi|23502119|ref|NP_698246.1|	DNA-directed RNA polymerase subunit beta'			0.63	4.53E-01	0.37
BR1243	gi|23502120|ref|NP_698247.1|	DNA-directed RNA polymerase subunit beta	0.55	2.0E-02	0.53	3.64E-03	
BR1492	gi|23502361|ref|NP_698488.1|	cold-shock family protein			0.63	1.77E-02	0.28
BRA0614	gi|23500358|ref|NP_699798.1|	nucleoside diphosphate kinase regulator	1.66	4.8E-03			
**Translation, ribosomal structure and biogenesis (J)**							
BR0790	gi|23501677|ref|NP_697804.1|	30S ribosomal protein S9	2.09	6.5E-04			
BR0830	gi|23501717|ref|NP_697844.1|	30S ribosomal protein S4	0.55	7.9E-04			
BR0877	gi|23501763|ref|NP_697890.1|	arginyl-tRNA synthetase			0.49	5.42E-03	
BR0899	gi|23501785|ref|NP_697912.1|	aspartyl/glutamyl-tRNA amidotransferase subunit B	0.34	2.7E-02			
BR1161	gi|23502039|ref|NP_698166.1|	elongation factor Ts	1.84	3.1E-03			
BR1219	gi|23502096|ref|NP_698223.1|	30S ribosomal protein S8	1.7	1.7E-02			
BR1230	gi|23502107|ref|NP_698234.1|	50S ribosomal protein L2			0.66	1.30E-04	
BR1710	gi|23502568|ref|NP_698695.1|	elongation factor P			1.52	6.93E-05	
BRA0187	gi|23499949|ref|NP_699389.1|	histidyl-tRNA synthetase	0.39	3.7E-04			
BRA0790	gi|23500524|ref|NP_699964.1|	lysyl-tRNA synthetase	0.52	1.2E-02			
**Posttranslational modification, protein turnover, chaperones (O)**							
BR0163	gi|23501074|ref|NP_697201.1|	heat shock protein 20	2.14	2.2E-02			
BR0835	gi|23501722|ref|NP_697849.1|	glutaredoxin-like protein			1.85	1.09E-08	
BR1108	gi|23501986|ref|NP_698113.1|	ATP-dependent protease ATP-binding subunit ClpX			0.64	3.56E-03	
BR1207	gi|23502084|ref|NP_698211.1|	serine protease	0.52	4.4E-03			
BRA0195	gi|23499956|ref|NP_699396.1|	molecular chaperone GroEL	0.26	3.1E-03			
BRA0195	gi|23499956|ref|NP_699396.1|	molecular chaperone GroEL	0.36	2.0E-04			
BRA1044	gi|23500766|ref|NP_700206.1|	glutathione S-transferase			0.48	3.45E-03	
**Cell envelope biogenesis, outer membrane (M)**							
BR0119	gi|23501033|ref|NP_697160.1|	Outer membrane protein			0.56	1.26E-02	
BR0522	gi|23501417|ref|NP_697544.1|	GDP-mannose 4,6-dehydratase			0.52	7.50E-05	0.42
BR0701	gi|23501588|ref|NP_697715.1|	outer-membrane protein Omp25	0.57	1.9E-02	0.38	7.26E-03	
BR0701	gi|23501588|ref|NP_697715.1|	outer-membrane protein Omp25	0.57	1.3E-02			
BR1429	gi|23502300|ref|NP_698427.1|	UDP-N-acetylenolpyruvoylglucosamine reductase	0.59	5.0E-03			
BR1475	gi|23502344|ref|NP_698471.1|	outer-membrane protein Omp28 BP26			0.63	4.76E-05	0.3
BR1622	gi|23502485|ref|NP_698612.1|	Outer membrane protein Omp31-1	0.51	1.2E-03	0.58	1.97E-02	0.4
BRA0423	gi|23500173|ref|NP_699613.1|	outer membrane protein omp31-2			0.37	2.59E-02	
**Carbohydrate transport and metabolism (G)**							
BR0639	gi|23501526|ref|NP_697653.1|	Omp2b porin, sugar permease			0.65	2.78E-02	
BR1138	gi|23502016|ref|NP_698143.1|	triosephosphate isomerase			1.54	1.28E-03	
BR1690	gi|23502548|ref|NP_698675.1|	phosphoglucosamine mutase			0.60	1.86E-03	
BR1727	gi|23502584|ref|NP_698711.1|	transketolase			0.60	1.90E-04	
BRA0858	gi|23500586|ref|NP_700026.1|	predicted erythritol ABC transporter 2, substrate-binding component	0.59	4.4E-03			
BRA1150	gi|23500864|ref|NP_700304.1|	D-xylose ABC transporter periplasmic D-xylose-binding protein	0.55	2.5E-02			

**Amino acid transport and metabolism (E)**							
BR0268	gi|23501175|ref|NP_697302.1|	urease subunit gamma			1.89	1.11E-04	
BR0765	gi|23501652|ref|NP_697779.1|	serine hydroxymethyltransferase			0.52	1.50E-03	0.49
BR1380	gi|23502251|ref|NP_698378.1|	ketol-acid reductoisomerase			0.56	2.93E-05	
BR1488	gi|23502357|ref|NP_698484.1|	carbamoyl phosphate synthase large subunit	0.6	7.4E-03			
BR1488	gi|23502357|ref|NP_698484.1|	carbamoyl phosphate synthase large subunit	0.55	3.3E-03			
BR1685	gi|23502543|ref|NP_698670.1|	D-3-phosphoglycerate dehydrogenase	0.54	1.7E-02			
BR1685	gi|23502543|ref|NP_698670.1|	D-3-phosphoglycerate dehydrogenase	0.52	3.4E-03	0.52	7.63E-07	
BR1685	gi|23502543|ref|NP_698670.1|	D-3-phosphoglycerate dehydrogenase	0.6	2.9E-02			
BR1687		phosphoserine aminotransferase			0.56	1.80E-07	
BR1906	gi|23502757|ref|NP_698884.1|	isopropylmalate isomerase large subunit	0.38	1.1E-04			
BRA0537	gi|23500284|ref|NP_699724.1|	oligopeptide ABC transporter, periplasmic oligopeptide-binding protein	0.26	1.4E-04			0.06
BRA0538	gi|23500285|ref|NP_699725.1|	oligopeptide ABC transporter periplasmic oligopeptide-binding protein	0.26	2.0E-04			0.11
BRA0538	gi|23500285|ref|NP_699725.1|	oligopeptide ABC transporter periplasmic oligopeptide-binding protein	0.35	2.3E-05			
BRA0538	gi|23500285|ref|NP_699725.1|	oligopeptide ABC transporter periplasmic oligopeptide-binding protein	0.24	1.2E-05			
BRA0538	gi|23500285|ref|NP_699725.1|	oligopeptide ABC transporter periplasmic oligopeptide-binding protein	0.17	8.8E-05			
BRA0538	gi|23500285|ref|NP_699725.1|	oligopeptide ABC transporter periplasmic oligopeptide-binding protein	0.20	3.9E-04	0.19	1.29E-04	
BRA0576	gi|23500320|ref|NP_699760.1|	peptide ABC transporter substrate-binding protein	0.59	4.3E-02			0.31
BRA0738	gi|23500475|ref|NP_699915.1|	glycine betaine/L-proline ABC transporter periplasmic glycine betaine-binding protein	1.65	2.3E-02			
BRA0890	gi|23500617|ref|NP_700057.1|	3-isopropylmalate dehydrogenase			0.57	5.58E-03	
**Energy production and conversion (C)**							
BR0500	gi|23501401|ref|NP_697528.1|	pyruvate phosphate dikinase	0.61	1.4E-02			
BR0806	gi|23501693|ref|NP_697820.1|	NADH dehydrogenase subunit E			1.69	1.49E-05	
BR0960	gi|23501843|ref|NP_697970.1|	monooxygenase			0.55	4.02E-05	
BR1069	gi|23501947|ref|NP_698074.1|	nitroreductase	1.9	3.4E-02			
BR1126	gi|23502004|ref|NP_698131.1|	pyruvate dehydrogenase complex, E3 component, dihydrolipoamide dehydrogenase			0.54	1.95E-03	0.36
BR1127	gi|23502005|ref|NP_698132.1	pyruvate dehydrogenase complex, E2 component, dihydrolipoamide acetyltransferase			0.62	4.50E-08	0.32
BR1129	gi|23502007|ref|NP_698134.1|	pyruvate dehydrogenase complex, E1 component alpha subunit			0.53	2.03E-03	
BR1148	gi|23502026|ref|NP_698153.1|	type II citrate synthase	0.55	1.7E-02	0.40	3.24E-03	0.3
BR1543	gi|23502411|ref|NP_698538.1|	ubiquinol-cytochrome c reductase, iron-sulfur subunit	2.76	1.4E-03			5.75
BR1543	gi|23502411|ref|NP_698538.1|	ubiquinol-cytochrome c reductase, iron-sulfur subunit	1.97	4.4E-03			
BR1614	gi|23502477|ref|NP_698604.1|	isocitrate lyase	0.47	1.0E-03	0.43	2.21E-01	0.04
BR1614	gi|23502477|ref|NP_698604.1|	isocitrate lyase	0.26	2.9E-03			
BR1614	gi|23502477|ref|NP_698604.1|	isocitrate lyase	0.22	1.7E-04			
BR1640	gi|23502500|ref|NP_698627.1|	succinate-semialdehyde dehydrogenase	0.20	3.6E-02			
BR1767	gi|23502624|ref|NP_698751.1|	ferredoxin A			2.08	1.74E-07	
BR1799	gi|23502652|ref|NP_698779.1|	ATP synthase F0F1 subunit beta	0.41	4.8E-02			

BR1799	gi|23502652|ref|NP_698779.1|	ATP synthase F0F1 subunit beta	0.46	9.1E-03			
BR1799	gi|23502652|ref|NP_698779.1|	ATP synthase F0F1 subunit beta	0.44	3.6E-02			
BR1923	gi|23502773|ref|NP_698900.1|	2-oxoglutarate dehydrogenase E1			0.43	4.55E-02	0.41
BR2089	gi|23502937|ref|NP_699064.1|	phosphoenolpyruvate carboxykinase	0.60	1.1E-03			
BRA0920	gi|23500647|ref|NP_700087.1|	L-lactate dehydrogenase			0.51	1.23E-03	
**Coenzyme Metabolism (H)**							
BR2160	gi|23503008|ref|NP_699135.1|	S-adenosylmethionine synthetase			0.54	8.21E-03	
**Lipid metabolism (I)**							
BR0907	gi|23501793|ref|NP_697920.1|	acetyl-CoA carboxylase biotin carboxyl carrier protein subunit	1.81	1.5E-03			
BR1778	gi|23502634|ref|NP_698761.1|	4-hydroxy-3-methylbut-2-en-1-yl diphosphate synthase	0.55	7.3E-03			
BR1510	gi|23502379|ref|NP_698506.1|	long-chain acyl-CoA thioester hydrolase			0.54	4.30E-02	
**Inorganic ion transport and metabolism (P)**							
BR2149	gi|23502997|ref|NP_699124.1|	DNA starvation/stationary phase protection protein Dps	1.83	1.2E-02			
BRA0355	gi|23500108|ref|NP_699548.1|	catalase			0.60	8.93E-04	
BRA0565	gi|23500309|ref|NP_699749.1|	bacterioferritin	2.81	3.6E-03			
BRA0565	gi|23500309|ref|NP_699749.1|	bacterioferritin	2.16	2.7E-03			
BRA0565	gi|23500309|ref|NP_699749.1|	bacterioferritin	3.11	7.9E-05	3.23	1.01E-07	
**Intracellular trafficking, secretion, and vesicular transport (U)**							
BR2072	gi|23502920|ref|NP_699047.1|	preprotein translocase subunit SecB	1.88	1.6E-02			
BR0890	gi|23501776|ref|NP_697903.1|	preprotein translocase subunit Yaj subunit			0.47	4.19E-02	
**Signal transduction mechanisms (T)**							
BR1966	gi|23502815|ref|NP_698942.1|	universal stress protein	1.83	4.2E-03			
BR1966	gi|23502815|ref|NP_698942.1|	universal stress protein	1.94	5.5E-04	1.92	1.07E-12	
BR1966	gi|23502815|ref|NP_698942.1|	universal stress protein	1.76	1.6E-03			
BR1966	gi|23502815|ref|NP_698942.1|	universal stress protein	1.82	1.4E-02			
**General function prediction only (R)**							
BR0203	gi|23501112|ref|NP_697239.1|	alcohol dehydrogenase	1.75	1.4E-05	2.86	4.72E-12	
BR0314	gi|23501221|ref|NP_697348.1|	proline iminopeptidase			0.55	1.08E-02	
BR1307	gi|23502183|ref|NP_698310.1|	CobW protein vit B12 biosynthèse			1.89	2.16E-04	
BRA0596	gi|23500340|ref|NP_699780.1|	metal-dependent hydrolase	1.63	3.0E-02			
BRA0635	gi|23500379|ref|NP_699819.1|	twin-arginine translocation pathway signal sequence domain-containing protein	0.31	9.5E-05			
BRA0990	gi|23500714|ref|NP_700154.1|	putative sulfite oxidase subunit YedY			0.45	5.15E-05	
**Function unknown (S)**							
BR0066	gi|23500981|ref|NP_697108.1|	hypothetical protein			2.08	2.36E-03	
BR0121	gi|23501035|ref|NP_697162.1|	hypothetical protein			0.65	1.14E-06	
BR0162	gi|23501073|ref|NP_697200.1|	hypothetical protein			1.72	1.08E-03	
BR0563	gi|23501452|ref|NP_697579.1|	hypothetical protein	0.49	2.2E-03			
BR0721	gi|23501608|ref|NP_697735.1|	oxidoreductase			1.96	2.19E-02	
BR1011	gi|23501891|ref|NP_698018.1|	hypothetical protein			0.60	3.58E-03	
BR1024	gi|23501904|ref|NP_698031.1|	hypothetical protein	0.54	6.8E-03			0.36
BR1163	gi|23502041|ref|NP_698168.1|	hypothetical protein			0.56	7.46E-07	0.24
BR1634	gi|23502495|ref|NP_698622.1|	hypothetical protein			0.54	6.41E-03	0.15
BR1681	gi|23502539|ref|NP_698666.1|	hypothetical protein			2.04	4.47E-04	
BR1822	gi|23502675|ref|NP_698802.1|	hypothetical protein			0.31	5.45E-04	
BR1989	gi|23502837|ref|NP_698964.1|	hypothetical protein	0.47	8.0E-03			
BR2114	gi|23502962|ref|NP_699089.1|	hypothetical protein			0.46	2.07E-04	
BRA0849	gi|23500578|ref|NP_700018.1|	hypothetical protein			0.56	5.19E-04	

[1)] In some cases, 2D-DIGE detected more than one spot for a given protein.
[2)] Ratio T WT/*regA*: ratio of wild-type/**Δ***regA* hybridization levels obtained in the transcriptome study.
