# Supplementary material for: RegA Plays a Key Role in Oxygen-Dependent Establishment of Persistence and in Isocitrate Lyase Activity, a Critical Determinant of In vivo Brucella suis Pathogenicity
Source: Front Cell Infect Microbiol. 2017 May 18;7:186. doi: 10.3389/fcimb.2017.00186 (PMC5435760; doi:10.3389/fcimb.2017.00186)
Supplement: Supplementary file 3 [file Table3.PDF]

**S3 Table: Genes selected for RT-qPCR analysis comparing wild-type and Δ*regA* strains**. Up-regulation (white) and down-regulation (grey) of RegA-dependent genes in the *B. suis* wild-type.

| Gene ID | Gene symbol | Function | WT/Δ*regA* (log2)[a)] | -ΔΔCt[b)] | Operon | RegA motif[c)] |
|---|---|---|---|---|---|---|
| **Translation, ribosomal structure and biogenesis (J)** | | | | | | |
| BR1119 | *nifR3* | nitrogen regulation protein Nifr3 | 1.27 | 1.54 | + | + |
| BR1914 | *trmD* | tRNA (guanine-N1)-methyltransferase | 1.16 | 1.18 | + | + |
| BR2167 | *truB* | tRNA pseudouridine synthase B | 1.44 | 1.44 | + | + |
| **Transcription (K)** | | | | | | |
| BR0550 | | transcriptional regulator, AraC family | 2.36 | 2.43 | | + |
| BR0604 | *feuP* | DNA-binding response regulator | 1.90 | 1.82 | +/- | + |
| BR0654 | *fnrN* | transcriptional regulator, Crp/Fnr family | 2.94 | 4.21 | | + |
| BR1613 | | transcriptional regulator, Cro/CI family | 1.30 | 1.68 | | + |
| **Posttranslational modification, protein turnover, chaperones(O)** | | | | | | |
| BR0607 | | cytochrome *c*-type biogenesis protein, putative | 2.97 | 3.83 | + | + |
| BR0905 | *aat* | leucyl/phenylalanyl-tRNA-protein transferase | 1.19 | 1.24 | +/- | + |
| **Signal transduction mechanisms (T)** | | | | | | |
| BR0605 | *feuQ* | sensor histidine kinase | 1.90 | 2.70 | +/- | + |
| BR1118 | *ntrB* | nitrogen regulation protein NtrB | 1.29 | 1.52 | + | + |
| **Energy production and conversion (C)** | | | | | | |
| BR0467 | *coxB* | cytochrome *c* oxidase, subunit II | 2.98 | 3.66 | + | + |
| BR0961 | *fumB* | fumarate hydratase, class I | 2.34 | 3.69 | | + |
| BR1543 | | ubiquinol-cytochrome *c* reductase, iron-sulfur subunit | 2.52 | 3.83 | + | + |
| BRA0508 | *cydD* | ABC transporter, ATP-binding protein CydD | 2.76 | 4.03 | + | + |
| **carbohydrate transport and metabolism (G)** | | | | | | |
| BRA0265 | | sugar ABC transporter, periplasmic sugar-binding protein, putative | 1.91 | 2.23 | | + |
| BRA0996 | *rbsB-3* | ribose ABC transporter, periplasmic D-ribose-binding protein | 1.16 | 1.43 | + | + |
| **nucleotide transport and metabolism (F)** | | | | | | |
| BR0672 | | MutT/nudix family protein | 1.21 | 1.24 | | + |
| **coenzyme metabolism (H)** | | | | | | |
| BR0757 | *hemB* | delta-aminolevulinic acid dehydratase | 1.86 | 2.46 | | + |
| **Transcription (K)** | | | | | | |
| BR1242 | *rpoC* | DNA-directed RNA polymerase, beta' subunit | -1.44 | -0.82 | | + |
| BRA0119 | *vjbR* | transcriptional regulator, LuxR family | -2.22 | -2.87 | | + |
| BRA0262 | *nnrA* | transcriptional regulator, Crp/Fnr family | -1.20 | -0.82 | | + |
| **DNA replication, recombination and repair (L)** | | | | | | |
| BR0001 | *dnaA* | chromosomal replication initiator protein | -1.24 | -0.96 | | + |
| BR1105 | | DNA-binding protein HU | -1.57 | -1.33 | | + |
| **Cell division and chromosome partitioning (D)** | | | | | | |
| BR1432 | *ftsW* | cell division protein FtsW | -1.25 | -1.17 | + | + |
| BR1895 | | cell division protein FtsK | -1.12 | -0.97 | | + |
| BRA0530 | | cell division protein FtsK, putative | -1.62 | -1.54 | | - |
| **Posttranslational modification, protein turnover, chaperones (O)** | | | | | | |
| BR0626 | | glutathione S-transferase family protein | -1.46 | -1.10 | | + |
| **Cell envelope biogenesis, outer membrane (M)** | | | | | | |
| BR0522 | *gmd* | GDP-mannose 4,6-dehydratase | -1.25 | -0.82 | + | + |
| BR0982 | | glycosyl transferase, group 1 family protein | -1.31 | -0.90 | + | - |
| BR1434 | *mraY* | phospho-N-acetylmuramoyl-pentapeptide-transferase | -1.43 | -3.10 | + | + |
| BR1475 | *omp28* | 28 kDa outer membrane protein | -1.74 | -1.71 | | - |

| | | | | | | |
|---|---|---|---|---|---|---|
| **Cell motility and secretion (N)** | | | | | | |
| BRA0151 | *flgB* | flagellar basal-body rod protein FlgB | -1.32 | -1.35 | + | + |
| **Inorganic ion transport and metabolism (P)** | | | | | | |
| BRA0703 | *sodC* | superoxide dismutase, Cu-Zn | -1.13 | -1.68 | | - |
| **Signal transduction mechanisms (T)** | | | | | | |
| BR0133 | | sensor histidine kinase | -1.28 | -1.53 | | + |
| **Energy production and conversion (C)** | | | | | | |
| BR0809 | *nuoH* | NADH dehydrogenase I, H subunit | -1.61 | -2.56 | + | + |
| BR1017 | *maeB* | NADP-dependent malic enzyme | -1.08 | -0.16 | | ND |
| BR1127 | *aceF* | pyruvate dehydrogenase complex, E2 component, dihydrolipoamid acetyltransferase | -1.66 | -1.49 | + | - |
| BR1148 | *gltA* | citrate synthase | -1.71 | -1.02 | | + |
| BR1614 | *aceA* | isocitrate lyase | -4.78 | -5.44 | + | - |
| BR1648 | *glcB* | malate synthase G | -1.00 | -0.31 | | ND |
| BR1729 | *pgk* | phosphoglycerate kinase | -1.40 | -1.78 | | + |
| BR1781 | *pyc* | pyruvate carboxylase | -1.16 | 0.99 | | ND |
| BR1923 | *sucA* | 2-oxoglutarate dehydrogenase, E1 component | -1.28 | -0.74 | + | + |
| BRA0248 | *norC* | nitric-oxide reductase, small subunit | -2.82 | -2.64 | + | + |
| BRA0260 | *nirK* | copper-containing nitrite reductase | -1.33 | -2.50 | + | + |
| BRA0275 | *nosZ* | nitrous-oxide reductase | -1.41 | -0.93 | + | + |
| BRA0299 | *narG* | respiratory nitrate reductase, alpha subunit | -1.55 | -0.82 | + | + |
| **carbohydrate transport and metabolism (G)** | | | | | | |
| BR0111 | | cyclic beta 1-2 glucan synthetase | -1.11 | -0.49 | | ND |
| BRA0385 | *xfp* | xylulose-5-phosphate/fructose-6-phosphate phosphoketolase | -1.74 | -0.99 | + | + |
| BRA0656 | *ugpA* | glycerol-3-phosphate ABC transporter, permease protein | -2.18 | -1.99 | + | - |
| **Amino acid transport and metabolism (E)** | | | | | | |
| BR0617 | *pepN* | aminopeptidase N | -2.01 | -1.04 | | - |
| BR1358 | *ureC-2* | urease, alpha subunit | -1.09 | -0.58 | + | + |
| BR1359 | *ureE-2* | urease accessory protein UreE, putative | -1.39 | -1.07 | + | + |
| BR1388 | *ilvH* | acetolactate synthase, small subunit | -1.07 | -2.36 | + | + |
| **nucleotide transport and metabolism (F)** | | | | | | |
| BR1043 | | ribonucleotide reductase subunit alpha | -1.52 | 0.95 | | ND |
| **coenzyme metabolism (H)** | | | | | | |
| BRA1199 | *hemN-2* | oxygen-independent coproporphyrinogen III oxidase | -2.17 | -1.71 | | - |
| **Lipid metabolism (I)** | | | | | | |
| BR0372 | *bacA* | bacteroid development protein BacA | -1.09 | -1.02 | | + |
| **Intracellular trafficking, secretion, and vesicular transport (U)** | | | | | | |
| BRA0066 | *virB4* | type IV secretion system protein VirB4 | -1.32 | -0.69 | + | - |
| BRA0068 | *virB2* | type IV secretion system protein VirB2 | -2.42 | -2.62 | + | - |
| BRA0069 | *virB1* | type IV secretion system protein VirB1 | -2.14 | -2.34 | + | - |
| **hypothetical function (S)** | | | | | | |
| BRA0332 | | hypothetical protein | -2.10 | -1.59 | | - |

[a)] WT/Δ*regA* (log2): log2 of the hybridization ratio WT/Δ*regA*.

[b)] -ΔΔCt: ΔΔCt (Gene of interest) = ΔCt (wild-type) - ΔCt (Δ*regA* mutant).

[c)] Presence (+) of putative RegA-binding motifs was searched in DNA sequences 1 kb upstream or 200 bp downstream and upstream the initiation codon of individual genes or of the first gene in operons, according to their induction or repression by RegA.

Genes predicted to be included in an operon according to BioCyc or DOOR were reported in the column "operon". +/- indicates discordance in prediction of operon structure between the two databases.
