# Supplementary material for: RegA Plays a Key Role in Oxygen-Dependent Establishment of Persistence and in Isocitrate Lyase Activity, a Critical Determinant of In vivo Brucella suis Pathogenicity
Source: Front Cell Infect Microbiol. 2017 May 18;7:186. doi: 10.3389/fcimb.2017.00186 (PMC5435760; doi:10.3389/fcimb.2017.00186)
Supplement: Supplementary file 4 [file Table4.PDF]

S4 Table. RegA-dependent genes and proteins essential for virulence of *Brucella* spp. Positive (white) or negative (grey) regulation by RegA during the set-up of persistence.

Gene ID	Protein	Gene name	Function	References
BR2166		*rbfA*	ribosome-binding factor A	Kim *et al*, 2012
BR0569	+	NI[a)]	Ros/MucR family transcriptional regulator	Mirabella *et al*, 2013<br>Caswell *et al*, 2013
BR0604		*feuP*	DNA-binding response regulator	Dorrell *et al*, 1998
BR0605		*feuQ*	sensor histidine kinase	Lestrate *et al*, 2003
BR0654		*fnrN*	transcriptional regulator, Crp/Fnr family	Abdou *et al*, 2013
BR1100		*gntR5*	transcriptional regulator, GntR family	Haine *et al*, 2005
BRA0813		*gntR1*	transcriptional regulator, GntR family	Haine *et al*, 2005
BRA0119		*vjbR*	transcriptional regulator, LuxR family	Delrue *et al*, 2005
BRA0262		*nnrA*	transcriptional regulator, Crp/Fnr family	Haine *et al*, 2006
BRA0947			D-aminopeptidase	Kim *et al*, 2003
BR1671		*macA*	HlyD family secretion protein	Lestrate *et al*, 2003
BR0521			perosamine synthase, putative	Godfroid *et al*, 1998
BR0522	+	*gmd*	GDP-mannose 4,6-dehydratase	Allen *et al*, 1998
BR0701	+	NI	outer-membrane protein Omp25	Edmonds *et al*, 2002
BR0982		*wbdA*	glycosyl transferase, group 1 family protein	Wu *et al*, 2006<br>Köhler *et al*, 2002
BR1475		*omp28*	immunoreactive 28 kDa outer membrane protein	Cloeckaert *et al*, 2004
BRA0135			glycosyl transferase, group 2 family protein	Delrue *et al*, 2004
BRA0703		*sodC*	Cu/Zn super oxide dismutase	Gee *et al*, 2005
BRA0508		*cydD*	ABC transporter, ATP-binding protein CydD	Köhler *et al*, 2002
BRA0509		*cydC*	ABC transporter, permease/ATP-binding protein	Kim *et al*, 2003
BR0500	+	NI	pyruvate phosphate dikinase	Zuniga-Ripa *et al*, 2014
BR1614	+	*aceA*	isocitrate lyase	This work
BR1729		*pgk*	phosphoglycerate kinase	Trant *et al*, 2010
BRA0246		*norE*	cytochrome c oxidase, subunit III	Lestrate *et al*, 2003
BRA0249		*norB*	nitric-oxide reductase, large subunit	Haine *et al*, 2006
BRA0919			oxidoreductase, molybdopterin-binding, putative	Lestrate *et al*, 2003
BRA0995		*rbsA-4*	ribose ABC transporter, ATP-binding protein	Delrue *et al*, 2004
BRA0385		*xfp*	xylulose-5-phosphate/fructose-6-phosphate phosphoketolase	Lestrate *et al*, 2003
BRA0655		*ugpB*	glycerol-3-phosphate ABC transporter, periplasmic glycerol-3-phosphate-binding protein	Castaneda-Roldan *et al*, 2006
BRA0656		*ugpA*	glycerol-3-phosphate ABC transporter, permease protein	Lestrate *et al*, 2003
BR0617		*pepN*	aminopeptidase N	Contreras-Rodriguez *et al*, 2003
BR0765	+	*glyA*	serine hydroxymethyltransferase, GlyA	Köhler *et al*, 2002
BR1380	+	NI	ketol-acid reductoisomerase, IlvC	Köhler *et al*, 2002
BR1389		*ilvB*	acetolactate synthase, catalytic subunit	Köhler *et al*, 2002
BR1488	+	NI	carbamoyl phosphate synthase subunit CarB	Köhler *et al*, 2002
BR1906	+	NI	isopropylmalate isomerase large subunit LeuC	Köhler *et al*, 2002
BR0372		bacA	bacteroid development protein BacA	LeVier *et al*, 2000
BRA0069-64, BRA0060-59		*virB1-B6, virB10-B11*	type IV secretion system proteins VirB1-VirB6 VirB10-VirB11	O'Callaghan *et al*, 1999

[a)]NI: not identified in the present transcriptome analysis.
