# Supplementary material for: RegA Plays a Key Role in Oxygen-Dependent Establishment of Persistence and in Isocitrate Lyase Activity, a Critical Determinant of In vivo Brucella suis Pathogenicity
Source: Front Cell Infect Microbiol. 2017 May 18;7:186. doi: 10.3389/fcimb.2017.00186 (PMC5435760; doi:10.3389/fcimb.2017.00186)
Supplement: Supplementary file 5 [file Table5.PDF]

S5 Table: Selected genes with their respective oligonucleotide sequences for qPCR

Gene	Oligonucleotide sequence		Gene	Oligonucleotide sequence
BR0757R	ACCTGACGGTGAACGACCTCA		BR1119R	CAAGCTGCACCACATGCGTTC
BR0757L	GCACGGTTGATGAGGTTATCC		BR1119L	GTGTTTCCGATCTCCCGTTTC
BR1614R	CGAAATGGGTGCAAACCGCCT		BR1242R	CGGGAAAGCGTGACTTCTACG
BR1614L	AACCTGCCAGCCGGAAAGGTA		BR1242L	CCTGAGAAGATTCTGTCCTGG
BR0672R	AAAGGAGCCGTGGAAGGGTTG		BRA0530R	CGGAGCAGATGGAATGACAGG
BR0672L	CGCCAGATCGATGGTGATGAC		BRA0530L	CCGTGGTGAAGTTCTGCCTGA
BR0467R	CTTGCTGGTCTGGGTGGTTCT		BR1432R	CATGACGAGAGCAACACCAAG
BR0467L	GAAGCTTACCGGGTTATCGAC		BR1432L	GGAATTCTCCTGTCCTTCGCC
BR0607R	GACCAGCTTCGCGAAGTTGAG		BR1895R	GGATCATCAGGAGAAGCGACC
BR0607L	CTGCAACAAACGCCAGCACAC		BR1895L	GGCAGGTCTATATCCTTCTGG
BR1543R	CGTTTATCGACCAGATGCGTCC		BR0626R	CATCTGCCACATCAGCCATTC
BR1543L	CGCATCCGTTGGAAGATTGGC		BR0626L	GGCAAGGGCGATCAGTTTGAAC
BR0961R	GTGTCGGTGGACACGTTCAAG		BRA0275R	CTTGAGGAATTCACGGTCCTG
BR0961L	GCTGCGATATTGGCGTTCTTC		BRA0275L	CAGTGTGGTGAAGTGCGTATTG
BR2167R	CGGTCTCGAAGATCAAGTGGC		BRA0656R	GGCCAGATCAGAAGAGTACGG
BR2167L	GAGTGCTTCCACTTCCTCACG		BRA0656L	CGCATTCGGGCTCAAATCCAC
BR0550R	GATGTCATGTCCTCGGATGAC		BR1781R	GACCGTAACCTGGATGGATCG
BR0550L	CTTGGCGCTGACATTTGTGCC		BR1781L	GCCATATGGGCTGAGGAAGAC
BR0604R	GTCGTTGACAGCGCCTATGAC		BR1358R	CATGGTGGAAGGGTTGCCTG
BR0604L	TGCCACATAGTCATCCGCACC		BR1358L	CCACTTATGGCGACGAACTGC
BR1118R	CCTTTCTTCCCTTCGGAAGCC		BRA0151R	CTTGACGATGCTGGTGTTGAG
BR1118L	GAAGAGAATGACCACCGCTCC		BRA0151L	GATGTGCTGGATAAGACGCAG
BRA0996R	CTGCAAATTGCGTGAAGGGAG		BR0372R	GATTGCCACACTCACCTGAACG
BRA0996L	CGCATCGGTAATCATCAGGTC		BR0372L	CTCATTGGCGCGCATGTGTTC
BR0133R	GTTCGTGGCGCTTTATCTGCA		BR1017R	CGGTTGATCTCATGCGCATCG
BR0133L	CAGCACGTCAAACGCCAGAAC		BR1017L	CCTCGCCATCCATGACGATC
BRA0262R	GAAGCGTCTTGCTTCTACCTG		BR1729R	CACATAGAGATCGCCATTGGC
BRA0262L	CATAGCCAGACCGGGATTCTG		BR1729L	CGCTCTCCAAGGTTCTGGATC
BRA1199 R	CTGGCTGAATTCGATCCCGGA		BR1127R	CCACATCCTCACCTTCTTCGG
BRA1199 L	CATCGGGCTGAATGATGGTCG		BR1127L	GACAAGGCCACGATGGAAGTC
BR1388R	GCAGTCCTCGTCGATAATGAG		BRA0265R	TGTTGCTGCTGCTTGGTCGCA
BR1388 L	CGATGGTGATGCGCGAAAGGT		BRA0265L	GAGGTCAAGGATGCAGTGGTTG
BR1434 R	CATCGGCGTTGATCGTGTTCC		BR1043R	CACGAGGGAACGTCATTCTCC
BR1434L	AGCAGGCATGACGCCAGAATG		BR1043L	GACCAGCGAGATCAAGAATCC
BR0522 R	GCAGTACACGGGATTAAGCGT		BR1492R	CGAGATTGTCGGCAGACGAAC
BR0522 L	ACGAGCTGCATGATTCGAACC		BR1492L	GCTTCATTCAGCCTGATCAGG
BRA0068R	CGCTGCAATCGAGCCTAACCT		BR0111R	TGCCAGGATGCCGTCAAGTTC
BRA0068L	CCGGAATGCCATCTTGTAACC		BR0111L	CTCAAGATCGGCGAGCTTTGG

BR0809 R	CTGCTCGTCGTATTGCTGATC		BRA0069R	CCCATCTTCAGGTTCTTGCAG
BR0809L	ACTTCAACAAGTCGGCGAAGG		BRA0069 L	TGCAATAGGCGTCGTTGGTGG
BR1105R	GGCTGTTGACGTAGTGCTTGC		BRA0385R	CCCTCCAGATAGGTGTTGG
BR1105L	GCGGGAATAGCAACTTCCTTG		BRA0385L	TCCAGCCTGAGCACATCAAGC
BR0001R	GAACTGCTGACCGAGCTTTGG		BRA0332 R	TGCAGATGGTTCTCTTGCTGG
BR0001L	GGCTTTGCTTCGGCATTGTCG		BRA0332 L	CGTGGTTTCTCTGGGAATTCC
BR1728R	AGGTCGTCGCCATCAACGATC		BRA0703 R	AGTTGGCACCGTGGTCATTTC
BR1728L	GCATTCAAGGGCGATATCGAC		BRA0703 L	GTGATGGGTATTACCCGGATC
BR1148R	TCCCACGCATTCACCTACGAC		16S R	ATGTCAAGGGCTGGTAAGGTT
BR1148L	GGTTTCGAGGAAGTCGCCATG		16S L	GGGGAGCAAACAGGATTAGAT
BR1923R	GGACGATCCGAATTCGGTCGA		BRA0248 R	CCCG AAA CGT CTT CTA CGG C
BR1923L	CTTCAGCTTGTCGGTGACATG		BRA0248 L	TCTCCCAGACATGCTTGCCC
BR1648 L	CCGGAGAAATTCTGGAAGGGC		BRA0508 R	CGGTCGAGCAGAAAGCCATTC
BR1648R	GACATTGGTGGTCGAAACCG		BRA 0508 L	GTGCCTTATCTGTCGCGCTTC
BRA0260 R	ATCGTCCTGATGCACGACCAC		BR0756R	CGTATCATGGCCTTCCTCATCGA
BRA0260 L	CGTCCATGCACACACTCAGGT		BR0756L	GTGTGCGCAACATAGAACAGC
BR0617R	CTCCACGGTGACGAATTGAAG		BR1359 R	CCAAGATCGATTGGCTTGTGC
BR0617L	TCGGTCACGATCTGCAAGGTG		BR1359 L	CACTTCCTTGAGATGGACGTG
BRA0119R	CGTAACGCATACCTATCGTGG		BR0617 R	CTCCACGGTGACGAATTGAAG
BRA0119L	GCAATGGCGCTCGTATCATTG		BR0617 L	TCGGTCACGATCTGCAAGGTG
BR1914R	CCCATCATCGACCGAATCCAG		BRA0299 R	GTCTGCATGTCCTTCTACGAC
BR1914L	GCTCGATACAGTGCAGATTCG		BRA0299 L	AGCATGAGGAAACCGGCATTG
BR0905R	CTCATACCAGGCTTCGACGGT		BR1475 R	TCCACAATCATGCTCGTCGGC
BR0905L	CCAGAAAACCATCCGTCAGGG		BR1475L	GCACCGAGAGATTGAGAATGG
BR0605R	GCGAAGGCGATTCAAGCTTGC		BRA0066 R	GAACGGTCACTTGCACCGTTC
BR0605L	GACCGCACATCTCTTCAGCCT		BRA0066 L	ACAGTTCCTCCTTGTCCTGCG

R: right, L: left
