# Supplementary material for: RegA Plays a Key Role in Oxygen-Dependent Establishment of Persistence and in Isocitrate Lyase Activity, a Critical Determinant of In vivo Brucella suis Pathogenicity
Source: Front Cell Infect Microbiol. 2017 May 18;7:186. doi: 10.3389/fcimb.2017.00186 (PMC5435760; doi:10.3389/fcimb.2017.00186)
Supplement: Supplementary file 6 [file Table6.PDF]

S6 Table: Analysis and significance of all proteins detected by ICPL in WT and Δ*regA* strains. Differential expression is indicated as the average ratio Δ*regA* /WT.

MS_run	Accession Number	Protein Name	Δ*regA* /WT ICPL4/ICPL6 ratio	Δ*regA* /WT ICPL10/ICPL6 ratio	Δ*regA* /WT ICPL4/ICPL0 ratio	Δ*regA* /WT ICPL10/ICPL0 ratio	WT/WT ICPL6/ICPL0 ratio	Δ*regA* /Δ*regA* ICPL10/ICPL4 ratio	Δ*regA* /WT Average	STDV % Δ*regA* /WT	T-Test, One-sample, equal	ICPL0/ICPL4 ratio	ICPL6/ICPL10 ratio	ICPL4/ICPL0 SD	ICPL6/ICPL0 SD	ICPL10/ICPL0 SD	ICPL0/ICPL4 SD	ICPL6/ICPL10 SD	ICPL10/ICPL6 SD	ICPL4/ICPL0 CV%	ICPL6/ICPL0 CV%	ICPL10/ICPL0 CV%	ICPL0/ICPL4 CV%	ICPL6/ICPL10 CV%	ICPL10/ICPL6 CV%	multiplet count	sequence count	unique peptides	Mascot peptide significance score sum
MS_run03	gi|161486701|ref|NP_697181.2|	trpS gene product [Brucella sui	1,17	0,94	1,26	1,02	1,08	0,81	1,10	12,31%	1,60E-01	0,79365079	1,058823529	0	0	0	0	0	0	0	0	0	0	0	0	1	3	3	105,26
MS_run01	gi|161486703|ref|NP_700229.2|	upp gene product [Brucella sui	0,97	1,14	0,96	1,13	0,99	1,18	1,05	8,56%	3,70E-01	1,04166667	0,876106195	0	0	0	0	0	0	0	0	0	0	0	0	1	6	6	258,08
MS_run02	gi|163842796|ref|YP_001627200.1|	hypothetical protein BSUIS A0	0,92	1,01	1,08	1,18	1,17	1,09	1,05	9,44%	1,91E-01	0,92592593	0,991525424	0	0	0	0	0	0	0	0	0	0	0	0	1	1	1	24,72
MS_run01	gi|163843413|ref|YP_001627817.1|	type II citrate synthase [Brucel	1,73	1,54	1,57	1,40	0,91	0,89	1,56	11,54%	1,38E-03	0,63694268	0,648044693	0,23445682	0,17020576	0,237970586	0,09856168	0,049434239	0,120470199	15	19	17	15	8	8	5	13	13	618,57
MS_run03	gi|163843644|ref|YP_001628048.1|	acetolactate synthase 3 catalyt	2,09	1,86	1,36	1,21	0,65	0,89	1,63	35,94%	2,72E-02	0,73529412	0,537190083	0	0	0	0	0	0	0	0	0	0	0	0	1	7	7	446,44
MS_run03	gi|163843968|ref|YP_001628372.1|	preprotein translocase subunit	1,47	1,31	1,22	1,09	0,83	0,89	1,27	13,85%	1,38E-02	0,81967213	0,76146789	0	0	0	0	0	0	0	0	0	0	0	0	1	7	7	263,87
MS_run02	gi|23499773|ref|NP_699213.1|	iunH gene product [Brucella su	1,11	1,06	1,07	1,02	0,96	0,95	1,07	3,35%	9,71E-03	0,93457944	0,941176471	0	0	0	0	0	0	0	0	0	0	0	0	1	2	2	99,56
MS_run03	gi|23499777|ref|NP_699217.1|	unnamed protein product [Bru	0,69	1,23	0,62	1,11	0,90	1,79	0,91	26,34%	1,45E-01	1,61290323	0,810810811	0	0	0	0	0	0	0	0	0	0	0	0	1	1	1	44,15
MS_run01	gi|23499794|ref|NP_699234.1|	unnamed protein product [Bru	1,00	0,83	0,99	0,82	0,99	0,83	0,90	11,89%	3,53E-01	1,01010101	1,207317073	0	0	0	0	0	0	0	0	0	0	0	0	1	1	1	23,77
MS_run02	gi|23499794|ref|NP_699234.1|		0,80	0,91	0,88	1,00	1,10	1,14				1,13636364	1,1	0	0	0	0	0	0	0	0	0	0	0	0	1	1	1	30,01
MS_run03	gi|23499844|ref|NP_699284.1|	omp10 gene product [Brucella	1,16	0,93	0,85	0,69	0,73	0,81	0,84	15,92%	3,72E-01	1,51111111	1,077586207	0,56568542	0,08485281	0,148492424	1,00566298	0,109723466	0,094984493	67	12	22	67	10	10	2	3	3	168,54
MS_run03	gi|23499853|ref|NP_699293.1|	modA gene product [Brucella s	0,90	0,70	0,83	0,64	0,92	0,77	0,77	10,42%	2,40E-01	1,20481928	1,4375	0	0	0	0	0	0	0	0	0	0	0	0	1	2	2	93,46
MS_run01	gi|23499864|ref|NP_699304.1|	unnamed protein product [Bru	1,46	1,54	1,02	1,08	0,70	1,06	1,25	29,03%	1,32E-01	0,98039216	0,648148148	0	0	0	0	0	0	0	0	0	0	0	0	1	3	3	135,64
MS_run02	gi|23499864|ref|NP_699304.1|	unnamed protein product [Bru	1,33	1,49	1,28	1,43	0,96	1,12				0,78125	0,671328671	0	0	0	0	0	0	0	0	0	0	0	0	1	3	3	149,08
MS_run03	gi|23499864|ref|NP_699304.1|	unnamed protein product [Bru	0,93	0,63	1,70	1,14	1,82	0,67				0,58823529	1,596491228	0	0	0	0	0	0	0	0	0	0	0	0	1	2	2	98,82
MS_run02	gi|23499876|ref|NP_699316.1|	gnd gene product [Brucella sui	0,66	12,19	0,64	11,82	0,97	18,47	6,33	567,79%	3,33E-01	1,5625	0,082064298	0	0	0	0	0	0	0	0	0	0	0	0	1	4	4	171,81
MS_run01	gi|23499880|ref|NP_699320.1|	unnamed protein product [Bru	0,34	0,41	1,06	1,28	3,14	1,21	0,65	35,93%	4,92E-04	0,94339623	2,453125	0	0	0	0	0	0	0	0	0	0	0	0	1	4	4	188,16
MS_run02	gi|23499880|ref|NP_699320.1|	unnamed protein product [Bru	0,25	0,24	0,93	0,90	3,76	0,97				1,07526882	4,177777778	0	0	0	0	0	0	0	0	0	0	0	0	1	4	4	173,32
MS_run03	gi|23499880|ref|NP_699320.1|	unnamed protein product [Bru	0,30	0,32	0,87	0,92	2,91	1,06				1,14942529	3,163043478	0	0	0	0	0	0	0	0	0	0	0	0	1	3	3	123,78
MS_run01	gi|23499937|ref|NP_699377.1|	unnamed protein product [Bru	0,58	0,50	0,66	0,56	1,13	0,85	0,51	8,03%	1,03E-05	1,51515152	2,017857143	0	0	0	0	0	0	0	0	0	0	0	0	1	1	1	56
MS_run03	gi|23499937|ref|NP_699377.1|	unnamed protein product [Bru	0,44	0,49	0,40	0,45	0,91	1,13				2,5	2,022222222	0	0	0	0	0	0	0	0	0	0	0	0	1	1	1	45,92
MS_run01	gi|23499945|ref|NP_699385.1|	unnamed protein product [Bru	0,88	0,94	0,76	0,84	0,86	1,11	0,80	14,63%	7,65E-03	1,26582278	1,068965517	0,04242641	0,1123981	0,045825757	0,26398937	0,139880603	0,151860122	6	13	5	21	13	16	3	6	6	245,93
MS_run02	gi|23499945|ref|NP_699385.1|	unnamed protein product [Bru	0,70	0,38	0,92	0,83	1,32	0,90				1,08695652	1,356899295	0,16653328	0,60104076	0,177857621	0,16789979	0,402499518	0,325954965	18	46	21	15	30	86	3	6	6	274,96
MS_run03	gi|23499945|ref|NP_699385.1|	unnamed protein product [Bru	0,86	0,94	0,76	0,80	0,88	1,05				1,31578947	1,063157895	0,1719593	0,11388591	0,124485608	0,26599627	0,144773305	0,113224929	23	13	16	20	14	12	6	8	8	423,88
MS_run01	gi|23499956|ref|NP_699396.1|	groEL gene product [Brucella s	0,86	0,80	0,90	0,79	1,04	0,88	0,80	6,50%	2,89E-05	1,11735331	1,255319149	0,12042196	0,12953498	0,099409926	0,15902671	0,254131846	0,151550897	13	12	13	14	20	19	31	42	42	5015,25
MS_run02	gi|23499956|ref|NP_699396.1|	groEL gene product [Brucella s	0,85	0,78	0,89	0,81	1,05	0,91				1,12359551	1,278481013	0,12588619	0,14425267	0,117936625	0,17008568	0,1781279	0,118756719	14	14	15	15	14	15	31	42	42	3926,49
MS_run03	gi|23499956|ref|NP_699396.1|	groEL gene product [Brucella s	0,70	0,69	0,75	0,74	1,07	0,98				1,33333333	1,450704225	0,13004395	0,12264521	0,151497144	0,22839825	0,27527744	0,106284231	17	12	21	17	19	15	32	43	43	4417,35
MS_run01	gi|23499957|ref|NP_699397.1|	groES gene product [Brucella s	0,73	0,69	0,80	0,82	1,11	1,01	0,69	13,22%	1,73E-05	1,24266749	1,445783133	0,10888231	0,07558108	0,108882309	0,17813365	0,152184397	0,086046275	14	7	13	14	11	12	10	10	10	1072,23
MS_run02	gi|23499957|ref|NP_699397.1|	groES gene product [Brucella s	0,69	0,72	0,85	0,88	1,23	1,04				1,17712228	1,390804598	0,1348809	0,15946339	0,180712874	0,15943912	0,202441462	0,102801297	16	13	21	14	15	14	9	9	9	513,33
MS_run03	gi|23499957|ref|NP_699397.1|	groES gene product [Brucella s	0,44	0,52	0,61	0,56	1,40	0,92				1,63934426	1,926243568	0,04947342	0,34100691	0,101101081	0,13287999	0,489594821	0,110122807	8	24	18	8	25	21	13	11	11	1046,79
MS_run01	gi|23499963|ref|NP_699403.1|	ileS gene product [Brucella suis	1,72	1,22	1,60	1,13	0,93	0,71	1,56	28,95%	4,18E-04	0,625	0,82300885	0	0	0	0	0	0	0	0	0	0	0	0	1	5	5	190,46
MS_run02	gi|23499963|ref|NP_699403.1|	ileS gene product [Brucella suis	1,74	1,27	1,77	1,30	1,02	0,73				0,56497175	0,784615385	0	0	0	0	0	0	0	0	0	0	0	0	1	3	3	84,63
MS_run03	gi|23499963|ref|NP_699403.1|	ileS gene product [Brucella suis	1,44	2,01	1,45	2,03	1,01	1,40				0,68965517	0,497536946	0	0	0	0	0	0	0	0	0	0	0	0	1	4	4	160,9
MS_run01	gi|23499972|ref|NP_699412.1|	unnamed protein product [Bru	1,22	0,92	1,20	0,90	0,98	0,75	3,23	310,01%	4,15E-02	0,83333333	1,088888889	0	0	0	0	0	0	0	0	0	0	0	0	1	1	1	42,99
MS_run02	gi|23499972|ref|NP_699412.1|	unnamed protein product [Bru	7,35	1,02	9,12	1,25	1,24	0,14				0,56651945	0,981789202	11,5824091	0,49497475	0,38890873	0,71948026	0,090882213	0,094690647	127	40	31	127	9	9	2	2	2	80,51
MS_run03	gi|23499972|ref|NP_699412.1|	unnamed protein product [Bru	6,33	0,99	7,34	1,13	1,16	0,15				0,49550735	1,039905549	8,83883476	0,18384776	0,113137085	0,59669041	0,266813846	0,255126809	120	16	10	120	26	26	2	2	2	66,28
MS_run03	gi|23500009|ref|NP_699449.1|	unnamed protein product [Bru	3,05	3,02	3,23	3,20	1,06	0,99	3,12	9,22%	7,12E-06	0,30959752	0,33125	0	0	0	0	0	0	0	0	0	0	0	0	1	1	1	43,17
MS_run01	gi|23500077|ref|NP_699517.1|	minD gene product [Brucella su	0,55	0,55	0,78	0,80	1,41	1,03	0,76	13,83%	2,28E-03	1,28205128	1,781954307	0,15885003	0,22291628	0,12832251	0,1953192	0,512267501	0,076348816	20	16	16	15	29	14	4	7	7	499,48
MS_run02	gi|23500077|ref|NP_699517.1|	minD gene product [Brucella su	0,81	0,73	0,70	0,59	0,86	0,84				1,43589744	1,513111888	0,07071068	0,24041631	0,098994949	0,14504754	0,661367357	0,31937706	10	28	17	10	44	44	2	8	8	355,15
MS_run03	gi|23500077|ref|NP_699517.1|	minD gene product [Brucella su	0,87	0,97	0,86	0,96	0,98	1,12				1,17638965	1,036159324	0,09192388	0,05656854	0,16263456	0,12647755	0,117221752	0,109886223	11	6	17	11	11	11	2	7	7	403,75
MS_run01	gi|23500078|ref|NP_699518.1|	minE gene product [Brucella su	1,47	1,02	1,84	1,28	1,25	0,70	1,14	35,98%	4,71E-01	0,54347826	0,9765625	0	0	0	0	0	0	0	0	0	0	0	0	1	3	3	142,48
MS_run03	gi|23500078|ref|NP_699518.1|	minE gene product [Brucella su	0,68	1,00	0,73	1,07	1,07	1,47				1,36986301	1	0	0	0	0	0	0	0	0	0	0	0	0	1	2	2	261,55
MS_run03	gi|23500094|ref|NP_699534.1|		0,57	0,60	0,59	0,62	1,03	1,05	0,60	1,72%	5,28E-06	1,69491525	1,661290323	0	0	0	0	0	0	0	0	0	0	0	0	1	1	1	22,77
MS_run02	gi|23500100|ref|NP_699540.1|	unnamed protein product [Bru	0,74	0,75	0,71	0,72	0,96	1,01	0,76	4,34%	3,80E-06	1,40956919	1,339783282	0,02828427	0,04242641	0,056568542	0,05615301	0,164188881	0,092161197	4	4	8	4	12	12	2	3	3	77,54
MS_run03	gi|23500100|ref|NP_699540.1|	unnamed protein product [Bru	0,73	0,82	0,75	0,84	1,03	1,12				1,33333333	1,226190476	0	0	0	0	0	0	0	0	0	0	0	0	1	2	2	50,46
MS_run01	gi|23500108|ref|NP_699548.1|	katA gene product [Brucella su	1,42	1,39	1,32	1,13	0,93	0,86	1,68	52,57%	8,93E-04	0,75774971	0,719562955	0,51860068	0,36251108	0,265179185	0,18645048	0,099549583	0,221907266	39	39	23	25	14	16	8	16	16	1150,69
MS_run02	gi|23500108|ref|NP_699548.1|	katA gene product [Brucella su	1,83	1,41	1,43	1,18	0,78	0,83				0,6921161	0,744372881	0,18248288	0,23245071	0,282193787	0,23668584	0,290149907	0,623648057	13	30	24	34	39	44	4	14	14	1175,52
MS_run03	gi|23500108|ref|NP_699548.1|	katA gene product [Brucella su	3,04	2,31	1,94	1,74	0,64	0,89				0,51430536	0,439880014	0,45841757	0,08502941	0,416685333	0,16838772	0,114710817	0,701366452	24	13	24	33	26	30	6	14	14	1108,11
MS_run02	gi|23500130|ref|NP_699570.1|	unnamed protein product [Bru	1,32	1,13	1,25	1,10	0,95	0,88	1,20	8,69%	1,10E-02	0,8912656	1,042262148	0,56568542	0,07071068	0,67882251	0,40334077	0,57890939	0,630110709	45	7	62	45	56	56	2	1	1	52,66
MS_run01	gi|23500134|ref|NP_699574.1|	unnamed protein product [Bru	1,07	1,25	1,09	1,08	1,01	1,00	1,05	10,08%	6,64E-02	0,92214856	0,798480084	0,08602325	0,41601683	0,179080987	0,07768437	0,161331091	0,243928801	8	41	17	8	20	19	7	7	7	454,09
MS_run02	gi|23500134|ref|NP_699574.1|	unnamed protein product [Bru	1,12	1,03	1,13	1,07	1,01	0,95				0,88557994	0,905320108	0,04708149	0,21535242	0,078102497	0,03716023	0,220093579	0,196026339	4	21	7	4	24	19	7	7	7	500,2
MS_run03	gi|23500134|ref|NP_699574.1|	unnamed protein product [Bru	0,96	0,94	0,94	0,87	0,98	0,92				1,06382979	1,063506505	0,06683313	0,20287517	0,090055538	0,06836204	0,190498165	0,153764875	7	21	10	6	18	16	8	7	7	558,8
MS_run01	gi|23500173|ref|NP_699613.1|	omp31-2 gene product [Brucel	1,37	1,44	1,11	1,11	0,81	1,00	2,71	211,87%	2,59E-02	0,90097403	0,692307692	0,13936079	0,11186727	0,138844437	0,11110283	0,097548327	0,21370442	13	14	13	12	14	15	9	10	9	599,03
MS_run02	gi|23500173|ref|NP_699613.1|	omp31-2 gene product [Brucel	2,67	1,97	1,68	1,44	0,63	0,86				0,59525919	0,507381507	0,16182295	0,18096961	0,149855486	0,05339341	0,137248704	0,850677141	10	29	10	9	27	43	6	8	7	509,81
MS_run03	gi|23500173|ref|NP_699613.1|	omp31-2 gene product [Brucel	6,84	7,71	2,60	2,59	0,38	0,99				0,38573378	0,132864481	0,46482972	0,07737355	0,332966465	0,06307699	0,045271918	2,241349599	18	20	13	16	34	29	8	8	7	584,81
MS_run01	gi|23500208|ref|NP_699648.1|	unnamed protein product [Bru	1,13	1,00	1,12	0,99	0,99	0,88	1,11	13,50%	3,45E-02	0,89285714	1	0	0	0	0	0	0	0	0	0	0	0	0	1	1	1	27,05
MS_run02	gi|23500208|ref|NP_699648.1|	unnamed protein product [Bru	0,95	0,90	1,15	1,09	1,21	0,95				0,86956522	1,110091743	0	0	0	0	0	0	0	0	0	0	0	0	1	1	1	28,22
MS_run03	gi|23500208|ref|NP_699648.1|	unnamed protein product [Bru	1,37	1,14	1,34	1,12	0,98	0,84				0,74626866	0,875	0	0	0	0	0	0	0	0	0	0	0	0	1	1	1	30,29

MS_run01	gi|23500233|ref|NP_699673.1|	metA gene product [Brucella su	0,63	0,66	0,69	0,73	1,10	1,06	0,68	3,75%	1,64E-04	1,44927536	1,506849315	0	0	0	0	0	0	0	0	0	0	0	0	1	7	7	431,52
MS_run02	gi|23500244|ref|NP_699684.1|	unnamed protein product [Bru	0,74	1,07	0,52	0,75	0,70	1,44	0,85	16,93%	1,48E-01	1,92307692	0,933333333	0	0	0	0	0	0	0	0	0	0	0	0	1	4	4	165,76
MS_run03	gi|23500244|ref|NP_699684.1|	unnamed protein product [Bru	0,99	1,02	0,85	0,88	0,86	1,04				1,17647059	0,977272727	0	0	0	0	0	0	0	0	0	0	0	0	1	4	4	151,31
MS_run01	gi|23500281|ref|NP_699721.1|	unnamed protein product [Bru	1,02	1,16	1,01	1,15	0,99	1,14	1,09	7,06%	3,98E-01	0,99009901	0,860869565	0	0	0	0	0	0	0	0	0	0	0	0	1	1	1	22,22
MS_run03	gi|23500284|ref|NP_699724.1|	unnamed protein product [Bru	11,33	9,83	0,68	0,59	0,06	0,87	5,61	500,25%	1,51E-01	1,47058824	0,101694915	0	0	0	0	0	0	0	0	0	0	0	0	1	5	4	316,96
MS_run01	gi|23500285|ref|NP_699725.1|	unnamed protein product [Bru	4,15	3,81	5,81	5,33	1,40	0,92	5,15	138,73%	1,29E-04	0,17211704	0,262664165	0	0	0	0	0	0	0	0	0	0	0	0	1	3	3	133,7
MS_run03	gi|23500285|ref|NP_699725.1|	unnamed protein product [Bru	7,68	6,54	4,57	3,29	0,60	0,72				0,21881838	0,152968037	2,39945202	0,50056635	2,204137019	0,66542399	0,038782728	1,94456794	53	84	67	304	25	30	6	7	6	317,54
MS_run01	gi|23500309|ref|NP_699749.1|	bfr gene product [Brucella suis	0,36	0,32	0,33	0,29	0,91	0,88	0,31	7,09%	1,01E-07	3,03030303	3,137931034	0	0	0	0	0	0	0	0	0	0	0	0	1	5	5	277,22
MS_run02	gi|23500309|ref|NP_699749.1|	bfr gene product [Brucella suis	0,23	0,30	0,38	0,49	1,64	1,31				2,70562771	3,338054968	0,06363961	0,27577164	0,084852814	0,45916025	0,015248391	0,00136849	17	17	17	17	0	0	2	5	5	341,29
MS_run03	gi|23500309|ref|NP_699749.1|	bfr gene product [Brucella suis	0,27	0,22	0,31	0,25	1,14	0,82				3,27956989	4,569805195	0,00707107	0,10606602	0,042426407	0,07603299	0,351257589	0,016870004	2	9	17	2	8	8	2	6	6	444,45
MS_run01	gi|23500315|ref|NP_699755.1|	qor gene product [Brucella suis	1,16	0,92	1,28	1,01	1,10	0,79	1,36	31,05%	1,57E-02	0,78125	1,089108911	0	0	0	0	0	0	0	0	0	0	0	0	1	5	5	274,35
MS_run03	gi|23500315|ref|NP_699755.1|	qor gene product [Brucella suis	1,83	1,47	1,78	1,43	0,97	0,80				0,60489368	0,681746032	0,65760931	0,35355339	0,530330086	0,2241035	0,005611959	0,012074911	37	36	37	37	1	1	2	5	5	412,7
MS_run01	gi|23500319|ref|NP_699759.1|	unnamed protein product [Bru	1,37	1,44	1,23	1,16	0,90	0,94	1,38	30,97%	1,87E-03	0,81300813	0,692307692	0,16537835	0,08820431	0,2020396	0,14831765	0,158887304	0,24071089	13	10	17	18	23	17	5	6	6	281,84
MS_run02	gi|23500319|ref|NP_699759.1|	unnamed protein product [Bru	2,01	1,85	1,54	1,62	0,77	1,05				0,65210027	0,541941942	0,43792313	0,33809269	0,280903305	0,15349081	0,15103759	0,536395806	28	44	17	24	28	29	6	6	6	260,46
MS_run03	gi|23500319|ref|NP_699759.1|	unnamed protein product [Bru	1,23	1,16	0,98	1,01	0,80	1,03				1,02040816	0,865740741	0,11105554	0,14530756	0,142294129	0,11149696	0,244511509	0,265525878	11	18	14	11	28	23	8	8	8	439,02
MS_run03	gi|23500320|ref|NP_699760.1|	unnamed protein product [Bru	2,13	1,78	1,66	1,39	0,78	0,84	1,74	26,52%	7,78E-03	0,60240964	0,561151079	0	0	0	0	0	0	0	0	0	0	0	0	1	3	3	98,96
MS_run01	gi|23500338|ref|NP_699778.1|	gatA gene product [Brucella su	0,88	1,01	0,95	1,10	1,08	1,15	1,14	14,61%	7,06E-02	1,05274823	0,989139766	0,01414214	0,19091883	0,261629509	0,01567169	0,061981115	0,063474242	1	18	24	1	6	6	2	3	3	192,64
MS_run02	gi|23500338|ref|NP_699778.1|	gatA gene product [Brucella su	1,19	1,19	1,06	1,06	0,89	1,00				0,94339623	0,839622642	0	0	0	0	0	0	0	0	0	0	0	0	1	4	4	323,26
MS_run03	gi|23500338|ref|NP_699778.1|	gatA gene product [Brucella su	1,25	1,27	1,34	1,36	1,07	1,01				0,74626866	0,786764706	0	0	0	0	0	0	0	0	0	0	0	0	1	3	3	217,71
MS_run01	gi|23500339|ref|NP_699779.1|	gatC gene product [Brucella su	1,12	1,30	1,01	1,17	0,90	1,16	1,02	13,46%	2,55E-01	0,99009901	0,769230769	0	0	0	0	0	0	0	0	0	0	0	0	1	2	2	155,46
MS_run02	gi|23500339|ref|NP_699779.1|	gatC gene product [Brucella su	1,12	1,05	0,94	0,88	0,84	0,94				1,06382979	0,954545455	0	0	0	0	0	0	0	0	0	0	0	0	1	2	2	150,13
MS_run03	gi|23500339|ref|NP_699779.1|	gatC gene product [Brucella su	0,91	0,82	0,98	0,89	1,08	0,91				1,02040816	1,213483146	0	0	0	0	0	0	0	0	0	0	0	0	1	2	2	142,47
MS_run01	gi|23500369|ref|NP_699809.1|	unnamed protein product [Bru	1,18	1,59	0,66	0,89	0,56	1,35	0,97	29,63%	2,84E-01	1,51515152	0,629213483	0	0	0	0	0	0	0	0	0	0	0	0	1	1	1	35,02
MS_run03	gi|23500369|ref|NP_699809.1|	unnamed protein product [Bru	1,12	0,86	0,82	0,63	0,73	0,77				1,2195122	1,158730159	0	0	0	0	0	0	0	0	0	0	0	0	1	1	1	49,32
MS_run01	gi|23500377|ref|NP_699817.1|	unnamed protein product [Bru	1,59	1,83	1,91	2,20	1,20	1,15	1,40	50,54%	1,70E-01	0,52356021	0,545454545	0	0	0	0	0	0	0	0	0	0	0	0	1	3	3	100,06
MS_run03	gi|23500377|ref|NP_699817.1|	unnamed protein product [Bru	0,86	0,93	0,91	0,99	1,06	1,09				1,0989011	1,070707071	0	0	0	0	0	0	0	0	0	0	0	0	1	2	2	94,1
MS_run01	gi|23500399|ref|NP_699839.1|	ugpB gene product [Brucella su	0,83	0,85	1,10	1,25	1,33	1,14	1,06	19,17%	3,59E-01	0,90909091	1,189434163	0,29115288	0,25657358	0,268477187	0,17855478	0,212690574	0,148686644	26	19	21	20	18	17	5	9	9	516,49
MS_run02	gi|23500399|ref|NP_699839.1|	ugpB gene product [Brucella su	0,92	0,88	1,05	0,88	1,13	0,84				0,96491228	1,13	0,14200939	0,18903263	0,335062183	0,12830895	0,632450957	0,271261638	14	17	38	13	56	31	5	9	9	459,31
MS_run03	gi|23500399|ref|NP_699839.1|	ugpB gene product [Brucella su	1,48	1,14	1,26	1,07	0,85	0,85				0,79365079	0,888888889	0,26296388	0,38318403	0,159530561	0,1797902	0,235863202	0,342241664	21	45	15	23	27	30	6	9	9	475,83
MS_run01	gi|23500424|ref|NP_699864.1|	fliY gene product [Brucella suis	1,16	1,10	1,10	0,99	0,95	0,90	0,82	19,94%	9,84E-02	0,91754651	0,912621359	0,10874282	0,14023789	0,265581124	0,09085558	0,173115911	0,147310045	10	15	27	10	19	13	5	6	6	377,29
MS_run02	gi|23500424|ref|NP_699864.1|	fliY gene product [Brucella suis	0,74	0,70	0,73	0,78	0,98	1,08				1,23438206	1,420289855	0,38591882	0,21221059	0,134350288	0,19159901	0,236246865	0,151766385	53	22	17	16	17	22	3	5	5	306,08
MS_run03	gi|23500424|ref|NP_699864.1|	fliY gene product [Brucella suis	0,72	0,62	0,65	0,55	0,90	0,84				1,53846154	1,608258929	0,08074652	0,14024978	0,06350853	0,19109415	3,233754623	0,272824063	12	16	12	12	201	44	6	6	6	488,33
MS_run01	gi|23500436|ref|NP_699876.1|	ribH gene product [Brucella sui	0,94	0,94	0,91	0,91	0,97	1,00	1,20	58,22%	3,53E-01	1,12959285	1,095595596	0,21213203	0,0212132	0,240416306	0,26332179	0,266138288	0,228461568	23	2	26	23	24	24	2	4	4	197,8
MS_run02	gi|23500436|ref|NP_699876.1|	ribH gene product [Brucella sui	0,68	0,85	0,62	0,71	0,90	1,15				1,62698413	1,247009569	0,0212132	0,38183766	0,070710678	0,05611959	0,413606718	0,281461307	3	42	10	3	33	33	2	3	3	171,72
MS_run03	gi|23500436|ref|NP_699876.1|	ribH gene product [Brucella sui	1,50	1,73	2,13	2,45	1,42	1,15				0,46948357	0,579591837	0	0	0	0	0	0	0	0	0	0	0	0	1	2	2	107,69
MS_run01	gi|23500437|ref|NP_699877.1|	unnamed protein product [Bru	1,11	1,09	1,07	1,05	0,96	0,98	0,91	17,33%	2,48E-01	0,93457944	0,914285714	0	0	0	0	0	0	0	0	0	0	0	0	1	3	3	178,88
MS_run02	gi|23500437|ref|NP_699877.1|	unnamed protein product [Bru	0,73	0,81	0,84	0,93	1,15	1,11				1,19047619	1,23655914	0	0	0	0	0	0	0	0	0	0	0	0	1	3	3	186,11
MS_run03	gi|23500437|ref|NP_699877.1|	unnamed protein product [Bru	1,00	1,03	0,61	0,63	0,61	1,03				1,63934426	0,968253968	0	0	0	0	0	0	0	0	0	0	0	0	1	3	3	185,42
MS_run01	gi|23500440|ref|NP_699880.1|	unnamed protein product [Bru	1,40	1,05	1,27	1,16	0,91	0,91	1,08	19,32%	9,21E-02	0,78740157	0,950413223	0,21779195	0,14153916	0,143643076	0,16787764	0,09789361	0,130778511	17	16	12	21	10	12	3	5	5	258,77
MS_run02	gi|23500440|ref|NP_699880.1|	unnamed protein product [Bru	1,29	1,05	1,25	1,09	0,97	0,87				0,82323498	0,963900135	0,32958307	0,10816654	0,272763634	0,27122349	0,200399264	0,205136456	26	11	25	33	21	20	4	5	5	270,24
MS_run03	gi|23500440|ref|NP_699880.1|	unnamed protein product [Bru	0,77	0,95	0,85	0,83	1,11	0,98				1,17712228	1,104949209	0,07325754	0,47088215	0,325115364	0,0905961	0,447825756	0,298579394	9	42	39	8	41	32	5	5	5	306,06
MS_run01	gi|23500443|ref|NP_699883.1|	sodC gene product [Brucella su	1,60	1,53	1,54	1,44	0,96	0,94	1,44	12,14%	1,66E-07	0,64935065	0,655629139	0,30556505	0,13202273	0,088881944	0,10934066	0,08523982	0,215526445	20	14	6	17	13	14	5	5	5	243,53
MS_run02	gi|23500443|ref|NP_699883.1|	sodC gene product [Brucella su	1,49	1,39	1,55	1,55	1,04	1,00				0,64725597	0,718537914	0,11338724	0,10271319	0,142641742	0,05362138	0,099375935	0,23134092	7	10	9	8	14	17	6	5	5	244,73
MS_run03	gi|23500443|ref|NP_699883.1|	sodC gene product [Brucella su	1,33	1,21	1,39	1,25	1,05	0,90				0,72181544	0,828571429	0,1463785	0,15984367	0,190341798	0,07878457	0,215146424	0,285393533	11	15	15	11	26	24	6	7	7	290,74
MS_run01	gi|23500447|ref|NP_699887.1|	ahpD gene product [Brucella su	1,09	0,99	1,15	1,08	1,05	0,94	1,08	14,13%	2,01E-02	0,873779	0,986111111	0,14441837	0,29752951	0,121778487	0,11855373	0,215782779	0,227902024	13	28	11	14	22	23	7	9	9	586,71
MS_run02	gi|23500447|ref|NP_699887.1|	ahpD gene product [Brucella su	1,47	1,02	1,12	0,95	0,76	0,85				0,89635854	0,97826087	0,16891813	0,09073772	0,153704261	0,15648791	0,203039758	0,202945037	15	12	16	17	21	20	5	7	7	494,24
MS_run03	gi|23500447|ref|NP_699887.1|	ahpD gene product [Brucella su	1,18	1,02	0,90	1,02	0,76	1,13				1,11111111	0,981481481	0,34467376	0,2592682	0,18239152	0,57723605	0,23477283	0,225302746	38	34	18	52	24	22	5	8	8	472,47
MS_run01	gi|23500448|ref|NP_699888.1|	ahpC gene product [Brucella su	1,25	1,39	1,16	1,30	0,93	1,12	5,32	1326,53%	2,26E-01	0,86711712	0,724644346	0,06363961	0,10606602	0,304055916	0,04777749	0,088236589	0,169289682	6	11	23	6	12	12	2	7	7	589,89
MS_run02	gi|23500448|ref|NP_699888.1|	ahpC gene product [Brucella su	1,06	1,09	1,05	1,07	0,99	1,02				0,9587156	0,924159021	0,06363961	0,11313708	0,028284271	0,05838496	0,081306468	0,095568709	6	11	3	6	9	9	2	7	7	446,74
MS_run03	gi|23500448|ref|NP_699888.1|	ahpC gene product [Brucella su	3,13	49,28	1,10	1,02	0,35	0,93				0,86206897	0,018410688	0,09192388	0,46669048	0,275771645	0,51395887	0,002661214	31,69787426	8	133	27	60	14	64	3	8	8	686,69
MS_run01	gi|23500463|ref|NP_699903.1|	gcvH gene product [Brucella su	0,67	0,94	0,93	1,30	1,38	1,40	0,96	22,29%	4,56E-02	1,07526882	1,061538462	0	0	0	0	0	0	0	0	0	0	0	0	1	3	3	152,93
MS_run01	gi|23500475|ref|NP_699915.1|	unnamed protein product [Bru	0,68	0,71	0,71	0,72	1,05	1,01	0,77	16,17%	2,20E-03	1,4084507	1,366666667	0,09539392	0,15947832	0,199749844	0,18391775	0,047140452	0,246055172	13	15	28	13	3	34	3	6	6	257,42
MS_run02	gi|23500475|ref|NP_699915.1|	unnamed protein product [Bru	0,64	0,57	0,63	0,63	0,98	1,00				1,60503338	1,53125	0,05354126	0,19697716	0,223159136	0,14176996	0,41113606	0,309549056	9	20	36	9	27	54	4	7	7	294,24
MS_run03	gi|23500475|ref|NP_699915.1|	unnamed protein product [Bru	1,03	0,88	1,03	0,99	1,00	0,96				0,97123998	1,037694013	0,10812801	0,30270998	0,172336879	0,11772192	0,260199098	0,134656598	10	30	17	12	25	15	4	8	8	304,57
MS_run01	gi|23500493|ref|NP_699933.1|	unnamed protein product [Bru	0,09	1,08	1,00	7,55	10,94	7,55	1,85	245,85%	7,97E-03	1,00250627	1,096161477	0,07071068	14,1209224	8,633773798	0,0708879	0,617218314	0,610447981	7	129	114	7	56	56	2	4	4	221,84
MS_run02	gi|23500493|ref|NP_699933.1|	unnamed protein product [Bru	0,11	1,43	0,91	7,02	8,03	7,75				1,13836478	0,856661115	0,2192031	10,2742615	7,714534983	0,27572717	0,522525929	0,874737651	24	128	110	24	61	61	2	4	4	201,26
MS_run03	gi|23500493|ref|NP_699933.1|	unnamed protein product [Bru	0,72	0,99	0,56	0,73	0,78	1,29				1,78571429	0,882934703	0,07637626	0,0212132	0,38890873	0,22451515	0,173855924	0,374265729	14	3	54	13	20	38	3	5	5	249,34
MS_run02	gi|23500516|ref|NP_699956.1|	folD gene product [Brucella sui	0,95	0,85	1,04	0,93	1,10	0,89	0,92	7,26%	1,71E-02	0,96153846	1,182795699	0	0	0	0	0	0	0	0	0	0	0	0	1	3	3	106,16
MS_run03	gi|23500516|ref|NP_699956.1|	folD gene product [Brucella sui	0,81	0,87	0,93	1,00	1,15	1,08				1,07526882	1,15	0	0	0	0	0	0	0	0	0	0	0	0	1	4	4	159,24
MS_run02	gi|23500523|ref|NP_699963.1|	unnamed protein product [Bru	0,94	1,14	1,47	1,78	1,56	1,21	1,33	31,94%	4,35E-01	0,68027211	0,876404494	0	0	0	0	0	0	0	0	0	0	0	0	1	6	6	261,29
MS_run01	gi|23500529|ref|NP_699969.1|	unnamed protein product [Bru	0,98	1,07	0,89	0,96	0,91	1,07	0,89	11,84%	3,55E-01	1,16614256	1,064590965	0,24041631	0,03535534	0,431335137	0,31501088	0,51785427	0,518233929	27	4	45	27	49	49	2	2	2	77,68
MS_run02	gi|23500529|ref|NP_699969.1|	unnamed protein product [Bru	0,95	0,75	0,86	0,68	0,91	0,79				1,1627907	1,338235294	0	0	0	0	0	0	0	0	0	0	0	0	1	2	2	78,58
MS_run01	gi|23500578|ref|NP_700018.1|	unnamed protein product [Bru	1,58	1,40	1,61	1,43	1,02	0,89	1,78	35,91%	5,19E-04	0,62111801	0,713286713	0	0	0	0	0	0	0	0	0	0	0	0	1	2	2	124,49
MS_run02	gi|23500578|ref|NP_700018.1|	unnamed protein product [Bru	1,72	2,06	1,87	2,25	1,09	1,20				0,53475936	0,484444444	0	0	0	0	0	0	0	0	0	0	0	0	1	2	2	122,26
MS_run03	gi|23500578|ref|NP_700018.1|	unnamed protein product [Bru	1,63	2,54	1,29	2,01	0,79	1,56				0,7751938	0,393034826	0	0	0	0	0	0	0	0	0	0	0	0	1	2	2	123,88
MS_run01	gi|23500586|ref|NP_700026.1|	rbsB-2 gene product [Brucella :	1,11	1,87	1,21	1,91	1,09	1,58	1,20	41,71%	4,70E-01	0,83465545	0,532407407	0,33995098	0,08082904	0,49802945	0,18025395	0,009049366	0,3964359	28	7	26	22	2	21	4	9	9	564,08
MS_run02	gi|23500586|ref|NP_700026.1|	rbsB-2 gene product [Brucella :	0,84	0,81	1,00	0,88	1,19	0,88				1	1,142857143	0,12583057	0,54169487	0,323316151	0,1209017	0,374378714	0,09581594	13	46	37	12	33	12	3	9	9	592,71
MS_run01	gi|23500589|ref|NP_700029.1|	unnamed protein product [Bru	1,27	1,56	1,18	1,45	0,93	1,23	1,36	14,87%	7,27E-02	0,84745763	0,64137931	0	0	0	0	0	0	0	0	0	0	0	0	1	3	3	124,68
MS_run01	gi|23500597|ref|NP_700037.1|	tpiA gene product [Brucella sui	0,07	2,69	0,02	0,78	0,29	39,00	1,12	105,30%	9,85E-02	50	0,371794872	0	0	0	0	0	0	0	0	0	0	0	0	1	8	8	360,35
MS_run02	gi|23500597|ref|NP_700037.1|	tpiA gene product [Brucella sui	0,95	0,93	0,84	0,82	0,88	0,98				1,19047619	1,073170732	0	0	0	0	0	0	0	0	0	0	0	0	1	8	8	347,24
MS_run03	gi|23500597|ref|NP_700037.1|	tpiA gene product [Brucella sui	2,88	3,00	0,23	0,24	0,08	1,04				4,34782609	0,333333333	0	0	0	0	0	0	0	0	0	0	0	0	1	8	8	402,69
MS_run01	gi|23500598|ref|NP_700038.1|	unnamed protein product [Bru	0,71	0,76	0,82	0,87	1,15	1,07	0,84	12,56%	2,80E-04	1,22814949	1,32183908	0,03535534	0,1266228	0,100166528	0,05327809	0,31428829	0,162943573	4	11	12	4	24	22	3	8	8	664,57
MS_run02	gi|23500598|ref|NP_700038.1|	unnamed protein product [Bru	0,78	0,76	0,98	0,92	1,25	0,94				1,02051442	1,349431818	0,01414214	0,35355339	0,056568542	0,01472679	0,301323912	0,169705627	1	28	6	1	22	22	2	7	7	552,66

MS_run03	gi|23500598|ref|NP_700038.1|	unnamed protein product [Bru	0,75	0,67	1,02	1,08	1,36	1,06				0,98039216	1,315315315	0,16165808	0,32145503	0,182482876	0,1755994	0,474370566	0,132048345	16	24	17	18	36	20	3	8	8	704,74
MS_run01	gi|23500599|ref|NP_700039.1|	fbaA gene product [Brucella su	1,02	1,15	1,03	1,00	1,01	0,97	1,38	36,93%	3,92E-02	0,97316704	0,826530612	0,22246723	0,26663021	0,19956202	0,19926777	0,109626183	0,29568508	22	27	20	20	13	26	4	9	9	508,92
MS_run03	gi|23500599|ref|NP_700039.1|	fbaA gene product [Brucella su	1,78	2,02	1,44	1,63	0,81	1,14				0,69687257	0,4938648	0,00707107	0,00707107	0,014142136	0,00343389	5,323E-05	0,000218243	0	1	1	0	0	0	2	5	5	308,44
MS_run01	gi|23500603|ref|NP_700043.1|	unnamed protein product [Bru	0,43	0,37	0,34	0,30	0,80	0,88	0,75	73,32%	3,53E-01	2,94117647	2,666666667	0	0	0	0	0	0	0	0	0	0	0	0	1	2	2	57,5
MS_run03	gi|23500603|ref|NP_700043.1|	unnamed protein product [Bru	2,57	0,62	1,08	0,26	0,42	0,24				0,92592593	1,615384615	0	0	0	0	0	0	0	0	0	0	0	0	1	2	2	77,17
MS_run01	gi|23500606|ref|NP_700046.1|	dcp gene product [Brucella sui:	1,18	1,31	1,00	1,11	0,85	1,11	1,35	31,02%	5,58E-02	1	0,765765766	0	0	0	0	0	0	0	0	0	0	0	0	1	11	11	537,43
MS_run02	gi|23500606|ref|NP_700046.1|	dcp gene product [Brucella sui:	0,98	1,36	1,40	1,94	1,43	1,39				0,71428571	0,737113402	0	0	0	0	0	0	0	0	0	0	0	0	1	12	12	521,52
MS_run03	gi|23500606|ref|NP_700046.1|	dcp gene product [Brucella sui:	1,73	1,14	1,85	1,22	1,07	0,66				0,54054054	0,87704918	0	0	0	0	0	0	0	0	0	0	0	0	1	9	9	423,54
MS_run01	gi|23500610|ref|NP_700050.1|	leuD gene product [Brucella su	1,35	1,41	1,07	1,11	0,79	1,04	0,92	35,34%	1,47E-01	0,93457944	0,711711712	0	0	0	0	0	0	0	0	0	0	0	0	1	1	1	39,59
MS_run03	gi|23500610|ref|NP_700050.1|	leuD gene product [Brucella su	0,41	0,71	0,47	0,82	1,15	1,74				2,12765957	1,402439024	0	0	0	0	0	0	0	0	0	0	0	0	1	1	1	29,44
MS_run01	gi|23500617|ref|NP_700057.1|	leuB gene product [Brucella su	1,30	1,39	1,12	1,07	0,86	0,96	1,75	70,94%	5,58E-03	0,90341922	0,718052668	0,35998611	0,17988885	0,202821761	0,3246466	0,126094031	0,182237653	32	21	19	36	18	13	6	10	10	629,17
MS_run02	gi|23500617|ref|NP_700057.1|	leuB gene product [Brucella su	1,70	1,40	1,21	1,19	0,71	0,99				0,74626866	0,669491525	0,28825625	0,24863628	0,318710945	0,2410325	0,266150884	0,534375477	24	35	27	32	40	38	7	8	8	586,86
MS_run03	gi|23500617|ref|NP_700057.1|	leuB gene product [Brucella su	2,81	3,23	2,61	2,01	0,93	0,77				0,38314176	0,309565264	0,63614464	0,26901673	0,601764073	0,16426082	0,084606665	0,75551778	24	29	30	43	27	23	6	9	9	563,76
MS_run01	gi|23500620|ref|NP_700060.1|	trx-2 gene product [Brucella su	1,05	1,19	0,96	0,98	0,91	1,02	1,01	17,62%	2,92E-01	1,04211898	0,840848246	0,17629521	0,16195678	0,283319608	0,19053177	0,121064235	0,162811144	18	18	29	18	14	14	7	13	13	539,85
MS_run02	gi|23500620|ref|NP_700060.1|	trx-2 gene product [Brucella su	1,38	1,24	1,01	0,89	0,73	0,88				0,99009901	0,808988764	0,14889594	0,14370108	0,142056327	0,13122177	0,196122768	0,254993172	15	20	16	13	24	21	5	11	11	555,02
MS_run03	gi|23500620|ref|NP_700060.1|	trx-2 gene product [Brucella su	0,77	0,77	0,91	0,95	1,18	1,04				0,999901	1,305019305	0,17088007	0,26495283	0,172506039	0,32323923	0,101357019	0,067980692	19	23	18	32	8	9	4	11	11	474,64
MS_run02	gi|23500634|ref|NP_700074.1|	unnamed protein product [Bru	0,80	0,91	0,88	1,00	1,10	1,14	0,90	7,15%	1,23E-02	1,13636364	1,1	0	0	0	0	0	0	0	0	0	0	0	0	1	1	1	22,14
MS_run01	gi|23500647|ref|NP_700087.1|	lldD gene product [Brucella sui	1,25	1,23	1,09	1,00	0,87	0,91	1,98	70,23%	1,23E-03	0,91743119	0,813084112	0,25567558	0,16303374	0,125266383	0,18541833	0,138663972	0,203538842	23	19	13	20	17	17	5	7	7	359,11
MS_run02	gi|23500647|ref|NP_700087.1|	lldD gene product [Brucella sui	2,85	3,16	1,85	2,05	0,65	1,11				0,56991467	0,316335081	0,5939697	0,09899495	0,22627417	0,18297948	0,013373898	0,133767806	32	15	11	32	4	4	2	4	4	195,26
MS_run03	gi|23500647|ref|NP_700087.1|	lldD gene product [Brucella sui	2,76	2,52	2,03	2,01	0,74	0,99				0,47619048	0,397515528	0,10606602	0,13435029	0,565685425	0,14341407	0,074288319	0,434538316	5	18	28	30	19	17	3	5	5	278,13
MS_run01	gi|23500661|ref|NP_700101.1|	unnamed protein product [Bru	1,44	1,15	1,51	1,21	1,05	0,80	1,33	15,00%	2,79E-02	0,66225166	0,867768595	0	0	0	0	0	0	0	0	0	0	0	0	1	6	6	313,43
MS_run03	gi|23500665|ref|NP_700105.1|	unnamed protein product [Bru	1,50	1,38	1,23	1,13	0,82	0,92	1,31	14,10%	1,25E-02	0,81300813	0,725663717	0	0	0	0	0	0	0	0	0	0	0	0	1	4	4	166,58
MS_run03	gi|23500668|ref|NP_700108.1|	unnamed protein product [Bru	1,02	1,49	0,60	0,88	0,59	1,47	1,00	32,26%	4,70E-01	1,66666667	0,670454545	0	0	0	0	0	0	0	0	0	0	0	0	1	6	6	215,05
MS_run01	gi|23500686|ref|NP_700126.1|	unnamed protein product [Bru	0,95	0,97	0,99	1,07	1,04	1,08	0,99	8,60%	2,80E-01	1,02284528	1,095381181	0,15895492	0,15217862	0,339546266	0,1725412	0,540479413	0,405452037	16	15	32	17	49	42	4	4	4	301,61
MS_run02	gi|23500686|ref|NP_700126.1|	unnamed protein product [Bru	1,15	1,13	1,00	0,94	0,87	0,94				1,00010001	0,882978723	0,01414214	0,09451631	0,235442845	0,01414355	0,191331015	0,187541363	1	11	25	1	22	17	3	5	5	279,95
MS_run03	gi|23500686|ref|NP_700126.1|	unnamed protein product [Bru	0,85	0,92	0,90	0,98	1,06	1,09				1,11331024	1,084085557	0,20331421	0,12498	0,148895937	0,27339925	0,200615862	0,171915293	23	12	15	25	19	19	6	7	7	420,6
MS_run01	gi|23500714|ref|NP_700154.1|	unnamed protein product [Bru	1,73	2,19	1,30	1,64	0,75	1,26	2,23	53,88%	5,15E-05	0,76923077	0,457317073	0	0	0	0	0	0	0	0	0	0	0	0	1	5	5	258,56
MS_run02	gi|23500714|ref|NP_700154.1|	unnamed protein product [Bru	2,87	3,08	2,50	2,68	0,87	1,07				0,4	0,324626866	0	0	0	0	0	0	0	0	0	0	0	0	1	6	6	337,64
MS_run03	gi|23500714|ref|NP_700154.1|	unnamed protein product [Bru	2,24	2,76	1,68	2,07	0,75	1,23				0,5952381	0,362318841	0	0	0	0	0	0	0	0	0	0	0	0	1	5	5	308,54
MS_run01	gi|23500742|ref|NP_700182.1|	unnamed protein product [Bru	2,76	2,13	1,05	0,81	0,38	0,77	1,26	56,78%	6,34E-02	0,95238095	0,469135802	0	0	0	0	0	0	0	0	0	0	0	0	1	3	3	348,11
MS_run02	gi|23500742|ref|NP_700182.1|	unnamed protein product [Bru	1,07	1,06	1,24	1,23	1,16	0,99				0,81541395	0,943315508	0,18384776	0,16970563	0,183847763	0,1208968	0,003025056	0,003399552	15	15	15	15	0	0	2	3	3	321,86
MS_run03	gi|23500742|ref|NP_700182.1|	unnamed protein product [Bru	1,04	1,13	0,78	0,78	0,75	1,00				1,13122172	0,743119266	0,2251666	0,08485281	0,243378991	0,49873078	0,227430511	0,299968845	29	11	31	44	31	27	4	5	5	474,19
MS_run02	gi|23500744|ref|NP_700184.1|	rpmH gene product [Brucella s	1,15	1,25	1,02	1,11	0,89	1,09	1,13	8,14%	1,01E-01	0,98039216	0,801801802	0	0	0	0	0	0	0	0	0	0	0	0	1	1	1	27,72
MS_run01	gi|23500750|ref|NP_700190.1|	unnamed protein product [Bru	1,27	1,23	0,84	0,81	0,66	0,96	1,37	28,95%	8,06E-04	1,19047619	0,814814815	0	0	0	0	0	0	0	0	0	0	0	0	1	4	4	187,99
MS_run02	gi|23500750|ref|NP_700190.1|	unnamed protein product [Bru	1,69	1,59	1,37	1,29	0,81	0,94				0,72992701	0,627906977	0	0	0	0	0	0	0	0	0	0	0	0	1	3	3	127,07
MS_run03	gi|23500750|ref|NP_700190.1|	unnamed protein product [Bru	1,67	1,63	1,57	1,53	0,94	0,97				0,63694268	0,614379085	0	0	0	0	0	0	0	0	0	0	0	0	1	3	3	110,22
MS_run01	gi|23500754|ref|NP_700194.1|	dapE gene product [Brucella su	1,50	1,27	0,94	0,76	0,63	0,81	1,11	49,29%	3,48E-02	1,38012039	0,791657777	0,6363961	0,44547727	0,466690476	0,93436515	0,100023852	0,160882566	68	71	61	68	13	13	2	5	5	278,26
MS_run02	gi|23500754|ref|NP_700194.1|	dapE gene product [Brucella su	1,90	1,74	0,65	0,62	0,34	0,95				2,03598485	0,579292929	0,44547727	0,18384776	0,403050865	1,40617826	0,080710168	0,242866463	69	54	66	69	14	14	2	5	5	293,83
MS_run03	gi|23500754|ref|NP_700194.1|	dapE gene product [Brucella su	1,53	1,47	0,49	0,47	0,32	0,96				2,04081633	0,680851064	0	0	0	0	0	0	0	0	0	0	0	0	1	4	4	341,38
MS_run01	gi|23500766|ref|NP_700206.1|	unnamed protein product [Bru	1,58	1,47	0,87	0,81	0,55	0,93	2,07	92,66%	3,45E-03	1,14942529	0,679012346	0	0	0	0	0	0	0	0	0	0	0	0	1	5	5	229,25
MS_run02	gi|23500766|ref|NP_700206.1|	unnamed protein product [Bru	1,99	1,90	1,77	1,69	0,89	0,95				0,56497175	0,526627219	0	0	0	0	0	0	0	0	0	0	0	0	1	4	4	227,17
MS_run03	gi|23500766|ref|NP_700206.1|	unnamed protein product [Bru	3,99	2,78	3,51	2,45	0,88	0,70				0,28490028	0,359183673	0	0	0	0	0	0	0	0	0	0	0	0	1	4	4	200,76
MS_run01	gi|23500774|ref|NP_700214.1|	gpm gene product [Brucella su	0,91	1,01	0,87	0,96	0,95	1,11	0,91	7,04%	3,02E-03	1,30822747	0,994872914	0,417193	0	0,098994949	0,6309634	0,102591035	0,10420521	48	0	10	48	10	10	2	7	7	423
MS_run02	gi|23500774|ref|NP_700214.1|	gpm gene product [Brucella su	0,92	0,94	0,94	0,96	1,02	1,03				1,07471264	1,063212773	0,09192388	0,01414214	0,042426407	0,10565963	0,032256419	0,028548	10	1	4	10	3	3	2	7	7	510,45
MS_run03	gi|23500774|ref|NP_700214.1|	gpm gene product [Brucella su	0,80	0,75	0,96	0,90	1,20	0,93				1,04347826	1,339473027	0,05656854	0,12727922	0,021213203	0,06148755	0,11046336	0,061777399	6	11	2	6	8	8	2	7	7	437,48
MS_run03	gi|23500788|ref|NP_700228.1|	ubiE gene product [Brucella su	1,13	1,18	0,77	0,80	0,68	1,04	0,97	18,57%	3,01E-01	1,2987013	0,85	0	0	0	0	0	0	0	0	0	0	0	0	1	2	2	69,32
MS_run02	gi|23500840|ref|NP_700280.1|	unnamed protein product [Bru	0,74	0,82	0,69	0,76	0,93	1,10	0,77	4,82%	1,67E-05	1,44927536	1,223684211	0	0	0	0	0	0	0	0	0	0	0	0	1	2	2	58,72
MS_run03	gi|23500840|ref|NP_700280.1|	unnamed protein product [Bru	0,73	0,82	0,75	0,84	1,03	1,12				1,33333333	1,226190476	0	0	0	0	0	0	0	0	0	0	0	0	1	2	2	56,35
MS_run01	gi|23500903|ref|NP_700343.1|	unnamed protein product [Bru	1,22	0,89	1,14	1,06	0,93	0,93	0,92	15,76%	2,97E-01	0,89567551	1,048076923	0,19226718	0,16653328	0,192764969	0,15676121	0,234801417	0,08693546	17	18	18	18	22	10	4	16	16	691,42
MS_run02	gi|23500903|ref|NP_700343.1|	unnamed protein product [Bru	0,71	0,74	0,87	0,90	1,21	1,04				1,11111111	1,344444444	0,04949747	0,42035699	0,365102725	0,29249295	0,139211729	0,066128883	6	35	41	26	10	9	3	15	15	797,53
MS_run03	gi|23500903|ref|NP_700343.1|	unnamed protein product [Bru	1,05	0,88	0,84	0,72	0,80	0,86				1,19115145	1,15208807	0,06344289	0,09643651	0,178978583	0,08949749	0,253627032	0,233273788	8	12	25	8	22	26	4	16	16	724,1
MS_run01	gi|23500906|ref|NP_700346.1|	unnamed protein product [Bru	1,99	1,37	1,35	0,93	0,68	0,69	1,26	36,09%	5,88E-03	0,74074074	0,731182796	0	0	0	0	0	0	0	0	0	0	0	0	1	5	5	263,04
MS_run02	gi|23500906|ref|NP_700346.1|	unnamed protein product [Bru	1,68	1,74	1,16	1,20	0,69	1,03				0,86206897	0,575	0	0	0	0	0	0	0	0	0	0	0	0	1	5	5	303,29
MS_run03	gi|23500906|ref|NP_700346.1|	unnamed protein product [Bru	1,08	0,83	1,03	0,79	0,95	0,77				0,97087379	1,202531646	0	0	0	0	0	0	0	0	0	0	0	0	1	5	5	249,47
MS_run01	gi|23500918|ref|NP_697045.1|	dnaN gene product [Brucella su	1,00	1,02	0,99	0,82	0,99	0,83	0,95	8,01%	8,75E-02	1,01010101	0,982300885	0,1225969	0,2165179	0,149733096	0,13879952	0,203265612	0,208851803	12	22	18	14	21	21	5	12	12	594,75
MS_run02	gi|23500918|ref|NP_697045.1|	dnaN gene product [Brucella su	0,98	0,96	0,95	0,99	0,96	1,05				1,05965463	1,011235955	0,20605016	0,20448542	0,19583156	0,18955855	0,173398952	0,101148936	22	21	20	18	17	11	7	14	14	797,67
MS_run03	gi|23500918|ref|NP_697045.1|	dnaN gene product [Brucella su	0,83	0,86	0,92	1,11	1,11	1,21				1,08695652	1,168375272	0,14993332	0,21275965	0,271256336	0,14716859	0,217862362	0,14193368	16	19	24	14	19	16	5	13	13	743,21
MS_run01	gi|23500926|ref|NP_697053.1|	unnamed protein product [Bru	1,32	1,32	1,47	1,47	1,11	1,00	1,40	7,28%	4,37E-03	0,68027211	0,755102041	0	0	0	0	0	0	0	0	0	0	0	0	1	5	5	269,41
MS_run01	gi|23500930|ref|NP_697057.1|	unnamed protein product [Bru	0,98	0,92	1,09	1,03	1,12	0,94	0,89	9,84%	3,16E-03	0,92868706	1,089285714	0,1995829	0,29713914	0,15026643	0,16050818	0,146858448	0,112701175	18	27	15	17	13	12	5	10	10	717,73
MS_run02	gi|23500930|ref|NP_697057.1|	unnamed protein product [Bru	0,79	0,79	0,91	0,88	1,15	0,97				1,02040816	1,261904762	0,45460606	0,06110101	0,18266545	0,18561328	0,214116453	0,157670231	50	5	21	18	17	20	4	10	10	591,41
MS_run03	gi|23500930|ref|NP_697057.1|	unnamed protein product [Bru	0,78	0,78	0,87	0,85	1,12	0,97				1,06976215	1,294337607	0,11135529	0,21792583	0,182277261	0,24976339	0,209273939	0,142829124	13	19	22	23	16	18	4	10	10	688,89
MS_run02	gi|23500939|ref|NP_697066.1|	unnamed protein product [Bru	0,26	6,32	0,20	4,80	0,76	24,00	2,89	271,66%	1,37E-01	5	0,158333333	0	0	0	0	0	0	0	0	0	0	0	0	1	2	2	106,34
MS_run01	gi|23500942|ref|NP_697069.1|	rpsA gene product [Brucella su	0,88	0,82	0,89	0,91	1,01	1,02	0,92	7,26%	1,88E-02	1,12373737	1,222222222	0,07726381	0,10606078	0,15991475	0,09664986	0,123027448	0,094005342	9	11	18	9	10	11	16	18	18	1117,73
MS_run02	gi|23500942|ref|NP_697069.1|	rpsA gene product [Brucella su	1,00	0,93	1,04	0,98	1,04	0,95				0,96674762	1,080065359	0,14736833	0,21110639	0,149654147	0,13896744	0,272256305	0,250197077	14	20	15	14	25	27	14	18	18	1063,4
MS_run03	gi|23500942|ref|NP_697069.1|	rpsA gene product [Brucella su	0,86	0,80	1,01	0,89	1,18	0,88				0,99009901	1,24928662	0,18933419	0,31753024	0,157201216	0,16191041	0,402751683	0,19688779	19	27	18	16	32	25	15	17	17	1115,65
MS_run01	gi|23500948|ref|NP_697075.1|	unnamed protein product [Bru	0,84	1,11	0,77	1,02	0,92	1,32	0,95	12,34%	1,80E-01	1,2987013	0,901960784	0	0	0	0	0	0	0	0	0	0	0	0	1	1	1	26,81
MS_run03	gi|23500948|ref|NP_697075.1|	unnamed protein product [Bru	1,00	0,82	1,11	0,91	1,11	0,82				0,9009009	1,21978022	0	0	0	0	0	0	0	0	0	0	0	0	1	1	1	23,48
MS_run01	gi|23500969|ref|NP_697096.1|	unnamed protein product [Bru	0,85	0,87	0,90	0,92	1,06	1,02	1,23	25,32%	4,40E-02	1,11111111	1,152173913	0	0	0	0	0	0	0	0	0	0	0	0	1	1	1	47
MS_run02	gi|23500969|ref|NP_697096.1|	unnamed protein product [Bru	1,30	1,23	1,42	1,34	1,09	0,94				0,70422535	0,813432836	0	0	0	0	0	0	0	0	0	0	0	0	1	1	1	46,94
MS_run03	gi|23500969|ref|NP_697096.1|	unnamed protein product [Bru	1,45	1,42	1,52	1,49	1,05	0,98				0,65789474	0,704697987	0	0	0	0	0	0	0	0	0	0	0	0	1	1	1	37,42
MS_run03	gi|23500981|ref|NP_697108.1|	unnamed protein product [Bru	0,58	0,42	0,52	0,42	0,90	0,81	0,48	6,81%	2,36E-03	5,53763441	5,436666667	0,58689863	0,19091883	0,473761543	6,31073794	5,746421108	0,440451286	114	21	114	114	106	106	2	2	2	138,8
MS_run01	gi|23500989|ref|NP_697116.1|	argG gene product [Brucella su	1,13	1,37	0,96	1,03	0,85	1,07	1,15	21,19%	1,72E-02	1,04211898	0,731481481	0,1112954	0,26250714	0,111310377	0,12252847	0,145127923	0,277240132	12	31	11	12	20	20	6	10	10	468

MS_run02	gi|23500989|ref|NP_697116.1|	argG gene product [Brucella su	1,19	1,04	0,89	0,78	0,75	0,88				1,21170865	0,963541667	0,33941125	0,20506097	0,254558441	0,46209837	0,051559869	0,05561514	38	28	33	38	5	5	2	7	7	456,28
MS_run03	gi|23500989|ref|NP_697116.1|	argG gene product [Brucella su	1,44	1,48	1,17	1,33	0,82	1,14				0,85470085	0,676691729	0,06324555	0,11401754	0,192743353	0,0473944	0,191772525	0,735439432	5	14	14	6	28	50	6	10	10	441,54
MS_run01	gi|23500994|ref|NP_697121.1|	unnamed protein product [Bru	1,21	0,95	1,38	1,01	1,15	0,73	1,21	22,52%	3,56E-02	0,72463768	0,910407462	0,13527749	0,24716728	0,04	0,07003046	0,335239504	0,270168457	10	22	4	10	37	28	4	4	4	418,62
MS_run02	gi|23500994|ref|NP_697121.1|	unnamed protein product [Bru	1,47	1,23	1,54	1,29	1,05	0,84				0,64935065	0,813953488	0	0	0	0	0	0	0	0	0	0	0	0	1	2	2	152,79
MS_run03	gi|23500994|ref|NP_697121.1|	unnamed protein product [Bru	0,85	1,50	1,06	0,98	1,25	0,92				0,94339623	0,629860602	0,02516611	0,33867388	0,254558441	0,02265884	0,049544166	0,571095248	2	27	26	2	8	38	3	4	4	356,88
MS_run01	gi|23501007|ref|NP_697134.1|	acnA gene product [Brucella su	0,85	0,85	0,73	0,69	0,86	0,95	0,95	20,59%	4,22E-01	1,37631976	1,179104478	0,13048673	0,17219175	0,153278976	0,23041641	0,358413044	0,19458224	18	20	22	17	30	23	9	18	18	1218,35
MS_run02	gi|23501007|ref|NP_697134.1|	acnA gene product [Brucella su	0,77	0,81	0,91	0,92	1,18	1,01				1,09943216	1,238914067	0,16767315	0,2981191	0,188941496	0,23044066	0,350731588	0,207560757	18	25	21	21	28	26	10	20	20	1203,21
MS_run03	gi|23501007|ref|NP_697134.1|	acnA gene product [Brucella su	1,34	1,20	1,21	1,13	0,90	0,93				0,82644628	0,834738025	0,4426624	0,18406521	0,150764717	0,22798141	0,125786715	0,197003952	37	20	13	28	15	16	6	17	17	978,25
MS_run01	gi|23501030|ref|NP_697157.1|	unnamed protein product [Bru	0,62	0,51	1,29	1,06	2,07	0,82	1,14	39,22%	3,13E-01	0,7751938	1,952830189	0	0	0	0	0	0	0	0	0	0	0	0	1	5	5	382,58
MS_run02	gi|23501030|ref|NP_697157.1|	unnamed protein product [Bru	1,27	1,09	1,76	1,51	1,39	0,86				0,56818182	0,920529801	0	0	0	0	0	0	0	0	0	0	0	0	1	5	5	320,42
MS_run01	gi|23501033|ref|NP_697160.1|	unnamed protein product [Bru	1,06	1,10	1,07	1,18	1,01	1,10	1,79	80,78%	1,26E-02	0,93457944	0,90990991	0,15534907	0,16822604	0,043588989	0,11992268	0,158174176	0,004537722	15	17	4	13	17	0	3	3	2	320,93
MS_run02	gi|23501033|ref|NP_697160.1|	unnamed protein product [Bru	1,63	1,52	1,82	1,48	1,12	0,81				0,54945055	0,656976744	0,20792627	0,2454248	0,151437556	0,07424979	0,136869878	0,380306765	11	22	10	14	21	25	3	3	2	306,57
MS_run03	gi|23501033|ref|NP_697160.1|	unnamed protein product [Bru	4,07	2,31	2,26	1,96	0,55	0,87				0,44247788	0,413333333	0,11930353	0,09192388	0,378197479	0,02475321	0,126320961	0,15351829	5	17	19	6	31	7	3	3	2	246,27
MS_run01	gi|23501035|ref|NP_697162.1|	unnamed protein product [Bru	1,57	1,29	1,49	1,19	0,95	0,80	1,53	20,42%	1,14E-06	0,67114094	0,776470588	0,17797472	0,2006127	0,223073571	0,08876782	0,156723795	0,271977891	12	21	19	13	20	21	12	13	13	689,11
MS_run02	gi|23501035|ref|NP_697162.1|	unnamed protein product [Bru	1,91	1,73	1,50	1,27	0,79	0,84				0,66693344	0,579622132	0,23934434	0,22881579	0,312665956	0,12672693	0,286033933	0,566189685	16	29	25	19	49	33	10	14	14	611,36
MS_run03	gi|23501035|ref|NP_697162.1|	unnamed protein product [Bru	1,68	1,74	1,52	1,50	0,91	0,98				0,65789474	0,575949367	0,26355626	0,16542154	0,152666771	0,1021708	0,188995049	0,458887249	17	18	10	16	33	26	9	14	14	586,26
MS_run01	gi|23501064|ref|NP_697191.1|	ihfB gene product [Brucella sui	1,09	0,98	1,13	1,02	1,04	0,90	1,13	9,98%	2,23E-02	0,88495575	1,019607843	0	0	0	0	0	0	0	0	0	0	0	0	1	2	2	85,22
MS_run03	gi|23501064|ref|NP_697191.1|	ihfB gene product [Brucella sui	1,21	1,31	1,09	1,18	0,90	1,08				0,91743119	0,762711864	0	0	0	0	0	0	0	0	0	0	0	0	1	2	2	88,29
MS_run01	gi|23501071|ref|NP_697198.1|	yfiA gene product [Brucella sui	0,94	0,83	0,95	0,85	1,02	0,89	0,98	13,64%	3,85E-01	1,0583779	1,242334906	0,09899495	0,13435029	0,296984848	0,11028849	0,276005123	0,183354755	10	13	35	10	22	22	2	7	7	406,54
MS_run02	gi|23501071|ref|NP_697198.1|	yfiA gene product [Brucella sui	1,19	1,17	1,15	1,14	0,96	0,99				0,90228526	0,861945813	0,28991378	0,21213203	0,374766594	0,22845846	0,097706134	0,132361329	25	22	33	25	11	11	2	6	6	382,97
MS_run03	gi|23501071|ref|NP_697198.1|	yfiA gene product [Brucella sui	0,83	0,90	0,85	0,92	1,02	1,08				1,17647059	1,108695652	0	0	0	0	0	0	0	0	0	0	0	0	1	4	4	345,99
MS_run01	gi|23501073|ref|NP_697200.1|	unnamed protein product [Bru	0,65	0,74	0,70	0,80	1,08	1,14	0,58	16,50%	1,08E-03	1,42857143	1,35	0	0	0	0	0	0	0	0	0	0	0	0	1	2	2	107,36
MS_run03	gi|23501073|ref|NP_697200.1|	unnamed protein product [Bru	0,41	0,31	0,57	0,43	1,39	0,75				1,75438596	3,23255814	0	0	0	0	0	0	0	0	0	0	0	0	1	2	2	119,67
MS_run01	gi|23501074|ref|NP_697201.1|	unnamed protein product [Bru	1,04	1,01	1,05	0,94	1,01	0,90	1,11	84,02%	2,85E-01	0,83333333	0,985981308	0,48798907	0,38003289	0,429495828	0,22046181	0,142925173	0,13360165	46	38	46	26	14	13	4	4	3	199,69
MS_run02	gi|23501074|ref|NP_697201.1|	unnamed protein product [Bru	0,66	0,54	0,65	0,81	0,98	1,26				0,90785474	0,744361349	0,417193	0,44433471	0,384230833	0,22058202	0,658337902	0,401355252	65	45	47	24	88	74	3	4	3	159,52
MS_run03	gi|23501074|ref|NP_697201.1|	unnamed protein product [Bru	3,85	0,84	1,00	0,97	0,26	0,97				0,99019608	0,526293867	0,42158431	0,22627417	0,415812458	0,01386484	0,598496711	0,306986075	42	87	43	1	114	37	3	4	3	166,24
MS_run01	gi|23501082|ref|NP_697209.1|	grpE gene product [Brucella su	0,87	0,72	0,80	0,60	0,92	0,75	0,85	13,47%	9,05E-02	1,25	1,391304348	0,10016653	0,06658328	0,058594653	0,13695171	0,114897217	0,052225452	13	7	10	11	8	7	3	6	6	297,82
MS_run02	gi|23501082|ref|NP_697209.1|	grpE gene product [Brucella su	1,03	1,00	0,95	0,88	0,92	0,93				1,06323132	1,069791667	0,09192388	0,21213203	0,113137085	0,10342471	0,378596756	0,352909708	10	23	13	10	35	35	2	5	5	223,7
MS_run03	gi|23501082|ref|NP_697209.1|	grpE gene product [Brucella su	0,76	0,67	1,01	0,92	1,34	0,91				1,06036745	1,507166895	0,40705241	0,19754746	0,333716447	0,50446246	0,450248805	0,167531361	40	15	36	48	30	25	4	8	8	363,59
MS_run02	gi|23501095|ref|NP_697222.1|	unnamed protein product [Bru	0,98	1,05	0,94	1,01	0,96	1,07	1,08	21,64%	2,48E-01	1,06382979	0,95049505	0	0	0	0	0	0	0	0	0	0	0	0	1	3	3	115,33
MS_run03	gi|23501095|ref|NP_697222.1|	unnamed protein product [Bru	1,27	0,83	1,57	1,03	1,24	0,66				0,63694268	1,203883495	0	0	0	0	0	0	0	0	0	0	0	0	1	2	2	88,2
MS_run01	gi|23501102|ref|NP_697229.1|	unnamed protein product [Bru	1,42	1,37	1,48	1,42	1,04	0,96	2,87	333,35%	4,67E-01	0,67567568	0,732394366	0	0	0	0	0	0	0	0	0	0	0	0	1	5	5	255,47
MS_run02	gi|23501102|ref|NP_697229.1|	unnamed protein product [Bru	1,99	2,05	1,61	1,66	0,81	1,03				0,62111801	0,487951807	0	0	0	0	0	0	0	0	0	0	0	0	1	6	6	256,85
MS_run03	gi|23501102|ref|NP_697229.1|	unnamed protein product [Bru	0,89	12,24	0,56	7,71	0,63	13,77				1,78571429	0,081712062	0	0	0	0	0	0	0	0	0	0	0	0	1	6	6	332,94
MS_run01	gi|23501112|ref|NP_697239.1|	unnamed protein product [Bru	0,32	0,32	0,33	0,33	1,02	1,00	0,35	7,71%	4,72E-12	3,03030303	3,090909091	0	0	0	0	0	0	0	0	0	0	0	0	1	3	3	254,7
MS_run02	gi|23501112|ref|NP_697239.1|	unnamed protein product [Bru	0,22	0,28	0,27	0,33	1,24	1,24				4,06015038	4,06952381	0,11313708	0,1979899	0,120208153	1,70130955	2,0512831	0,141887171	42	16	36	42	50	50	2	5	5	274,66
MS_run03	gi|23501112|ref|NP_697239.1|	unnamed protein product [Bru	0,40	0,46	0,43	0,49	1,07	1,14				2,3255814	2,183673469	0	0	0	0	0	0	0	0	0	0	0	0	1	3	3	161,37
MS_run01	gi|23501117|ref|NP_697244.1|	unnamed protein product [Bru	0,74	0,99	0,79	1,06	1,07	1,34	0,97	20,76%	5,14E-03	1,26582278	1,009433962	0	0	0	0	0	0	0	0	0	0	0	0	1	9	9	591
MS_run02	gi|23501117|ref|NP_697244.1|	unnamed protein product [Bru	0,72	1,15	0,93	1,44	1,28	1,55				1,08111267	0,942629562	0,00707107	0,16970563	0,38890873	0,00826446	0,37372996	0,456484452	1	13	27	1	40	40	2	9	9	548,3
MS_run03	gi|23501117|ref|NP_697244.1|	unnamed protein product [Bru	0,78	0,79	1,11	1,12	1,42	1,01				0,9009009	1,267857143	0	0	0	0	0	0	0	0	0	0	0	0	1	8	8	593,94
MS_run01	gi|23501134|ref|NP_697261.1|	unnamed protein product [Bru	0,80	0,75	0,79	0,72	0,99	0,91	1,10	35,41%	1,38E-01	1,26582278	1,328571429	0,04932883	0,06506407	0,052915026	0,07234339	0,027893749	0,015292961	6	7	7	6	2	2	3	7	7	274,18
MS_run02	gi|23501134|ref|NP_697261.1|	unnamed protein product [Bru	1,11	0,98	0,94	0,82	0,85	0,87				1,08317887	1,021512386	0,14849242	0,38890873	0,318198052	0,17202551	0,078362551	0,075318391	16	46	39	16	8	8	2	7	7	279,42
MS_run03	gi|23501134|ref|NP_697261.1|	unnamed protein product [Bru	1,62	1,62	1,54	1,54	0,95	1,00				0,64935065	0,616883117	0	0	0	0	0	0	0	0	0	0	0	0	1	6	6	217,72
MS_run01	gi|23501156|ref|NP_697283.1|	infA gene product [Brucella sui	1,12	0,80	1,55	1,11	1,38	0,72	1,61	140,46%	1,76E-01	0,64516129	1,243243243	0	0	0	0	0	0	0	0	0	0	0	0	1	3	3	120,01
MS_run03	gi|23501156|ref|NP_697283.1|	infA gene product [Brucella sui	1,54	5,23	0,87	0,63	0,56	0,73				1,21148459	0,841892234	0,26162951	0,70710678	0,056568542	0,36642788	1,046797087	6,506271829	30	126	9	30	124	124	2	6	6	213,89
MS_run01	gi|23501159|ref|NP_697286.1|	hisD gene product [Brucella su	1,77	1,44	1,33	1,08	0,75	0,81	1,19	22,65%	2,18E-02	0,7518797	0,694444444	0	0	0	0	0	0	0	0	0	0	0	0	1	6	6	317,24
MS_run02	gi|23501159|ref|NP_697286.1|	hisD gene product [Brucella su	1,07	0,94	1,33	1,17	1,24	0,88				0,7518797	1,05982906	0	0	0	0	0	0	0	0	0	0	0	0	1	6	6	247,63
MS_run03	gi|23501159|ref|NP_697286.1|	hisD gene product [Brucella su	1,02	1,09	1,02	1,06	1,00	1,03				1,03291139	0,988349604	0,32526912	0,15556349	0,233345238	0,32938645	0,366057029	0,40233295	32	16	22	32	37	37	2	8	8	403,32
MS_run01	gi|23501175|ref|NP_697302.1|	ureA gene product [Brucella su	0,62	0,57	0,68	0,62	1,09	0,91	0,53	10,21%	1,11E-04	1,47058824	1,758064516	0	0	0	0	0	0	0	0	0	0	0	0	1	3	3	119,17
MS_run02	gi|23501175|ref|NP_697302.1|	ureA gene product [Brucella su	0,50	0,38	0,51	0,39	1,02	0,76				1,96078431	2,615384615	0	0	0	0	0	0	0	0	0	0	0	0	1	2	2	101,78
MS_run01	gi|23501177|ref|NP_697304.1|	ureC gene product [Brucella su	1,40	1,59	1,16	1,32	0,83	1,14	1,37	15,48%	3,98E-02	0,86206897	0,628787879	0	0	0	0	0	0	0	0	0	0	0	0	1	5	5	220,38
MS_run01	gi|23501178|ref|NP_697305.1|	ureE gene product [Brucella su	1,16	1,15	0,93	0,92	0,80	0,99	1,27	55,43%	5,35E-02	1,07526882	0,869565217	0	0	0	0	0	0	0	0	0	0	0	0	1	7	7	317,55
MS_run02	gi|23501178|ref|NP_697305.1|	ureE gene product [Brucella su	2,59	2,35	0,96	0,87	0,37	0,91				1,04166667	0,425287356	0	0	0	0	0	0	0	0	0	0	0	0	1	5	5	207,88
MS_run03	gi|23501178|ref|NP_697305.1|	ureE gene product [Brucella su	1,10	1,32	0,87	1,04	0,79	1,20				1,14942529	0,759615385	0	0	0	0	0	0	0	0	0	0	0	0	1	6	6	327,61
MS_run03	gi|23501180|ref|NP_697307.1|	ureG-1 gene product [Brucella	0,86	0,64	0,81	0,53	0,95	0,65	0,71	13,13%	2,83E-01	1,36455526	2,074690402	0,35355339	0,33234019	0,212132034	0,59560881	1,457449968	0,449516677	44	35	40	44	70	70	2	4	4	283,54
MS_run01	gi|23501193|ref|NP_697320.1|	unnamed protein product [Bru	0,77	1,16	0,70	1,06	0,91	1,51	1,09	34,96%	3,97E-01	1,42857143	0,858490566	0	0	0	0	0	0	0	0	0	0	0	0	1	6	6	401,4
MS_run02	gi|23501193|ref|NP_697320.1|	unnamed protein product [Bru	1,53	1,74	0,81	0,92	0,53	1,14				1,2345679	0,576086957	0	0	0	0	0	0	0	0	0	0	0	0	1	6	6	325,03
MS_run01	gi|23501208|ref|NP_697335.1|	argD gene product [Brucella su	1,11	1,02	1,34	1,24	1,21	0,93	1,18	12,10%	2,32E-01	0,74626866	0,975806452	0	0	0	0	0	0	0	0	0	0	0	0	1	1	1	57,1
MS_run01	gi|23501218|ref|NP_697345.1|	pyrD gene product [Brucella su	0,87	1,40	0,73	1,18	0,84	1,62	1,06	30,94%	1,34E-01	1,36986301	0,711864407	0	0	0	0	0	0	0	0	0	0	0	0	1	1	1	23,5
MS_run03	gi|23501218|ref|NP_697345.1|	pyrD gene product [Brucella su	0,73	1,44	0,72	1,41	0,98	1,96				1,38888889	0,695035461	0	0	0	0	0	0	0	0	0	0	0	0	1	1	1	24,17
MS_run01	gi|23501220|ref|NP_697347.1|	unnamed protein product [Bru	0,95	0,77	0,93	0,75	0,98	0,81	1,21	41,28%	7,71E-02	1,07526882	1,306666667	0	0	0	0	0	0	0	0	0	0	0	0	1	2	2	75,38
MS_run02	gi|23501220|ref|NP_697347.1|	unnamed protein product [Bru	1,88	2,01	1,47	1,57	0,78	1,07				0,68027211	0,496815287	0	0	0	0	0	0	0	0	0	0	0	0	1	2	2	85,08
MS_run03	gi|23501220|ref|NP_697347.1|	unnamed protein product [Bru	1,08	1,28	0,85	1,01	0,79	1,19				1,17647059	0,782178218	0	0	0	0	0	0	0	0	0	0	0	0	1	2	2	89,24
MS_run01	gi|23501221|ref|NP_697348.1|	pip gene product [Brucella suis	2,17	2,10	1,56	1,51	0,72	0,97	1,83	30,00%	1,08E-02	0,64102564	0,476821192	0	0	0	0	0	0	0	0	0	0	0	0	1	9	9	751,82
MS_run01	gi|23501242|ref|NP_697369.1|	unnamed protein product [Bru	0,96	0,98	1,06	1,08	1,10	1,02	1,02	4,96%	2,43E-01	0,94339623	1,018518519	0	0	0	0	0	0	0	0	0	0	0	0	1	1	1	24,65
MS_run01	gi|23501271|ref|NP_697398.1|	unnamed protein product [Bru	0,67	1,15	1,23	1,16	1,84	0,94	1,05	22,26%	1,88E-01	0,85195916	1,420991117	0,38890873	1,89504617	0,318198052	0,26828693	1,249255038	1,008360953	31	103	28	31	88	88	2	3	3	114,74
MS_run01	gi|23501278|ref|NP_697405.1|	unnamed protein product [Bru	1,40	1,04	1,34	1,00	0,96	0,74	1,12	21,67%	1,18E-02	0,78034009	0,96039604	0,3959798	0,03535534	0,021213203	0,2305962	0,056008458	0,06082639	30	4	2	30	6	6	2	3	3	99,8
MS_run03	gi|23501278|ref|NP_697405.1|	unnamed protein product [Bru	1,38	1,06	1,01	0,75	0,73	0,74				0,99487786	0,957805514	0,09899495	0,31112698	0,190918831	0,09751276	0,172166275	0,190750993	10	43	26	10	18	18	2	3	3	116,57
MS_run01	gi|23501288|ref|NP_697415.1|	unnamed protein product [Bru	1,38	1,27	1,49	1,41	1,08	0,95	1,09	22,27%	3,57E-01	0,71669884	0,817028986	0,51618795	0,26162951	0,685893578	0,24912546	0,212644431	0,329718331	35	24	49	35	26	26	2	4	4	143,15
MS_run02	gi|23501288|ref|NP_697415.1|	unnamed protein product [Bru	0,86	0,99	0,85	0,99	0,99	1,16				0,76046635	1,010204082	0,24041631	0,08544004	0,072341781	0,31102206	0,141068639	0,16705306	28	9	7	41	14	17	3	5	5	213,13
MS_run03	gi|23501288|ref|NP_697415.1|	unnamed protein product [Bru	0,99	0,97	0,83	1,07	0,84	1,29				1,05263158	0,625400077	0,16970563	0,15502688	0,400041664	0,39820388	0,225774461	0,429314831	20	18	37	38	36	44	3	4	4	169,48
MS_run01	gi|23501289|ref|NP_697416.1|	atpF gene product [Brucella su	0,96	0,86	0,88	0,79	0,92	0,90	0,87	5,94%	2,55E-01	1,13636364	1,164556962	0	0	0	0	0	0	0	0	0	0	0	0	1	4	4	542,95

MS_run03	gi|23501316|ref|NP_697443.1|	unnamed protein product [Bru	0,80	1,05	0,80	1,05	1,00	1,31	0,93	12,50%	9,24E-02	1,25	0,952380952	0	0	0	0	0	0	0	0	0	0	0	0	1	3	3	193,2
MS_run01	gi|23501321|ref|NP_697448.1|	unnamed protein product [Bru	1,43	1,47	6,33	6,96	4,44	1,10	4,05	260,72%	3,17E-01	0,76640878	0,682128993	7,97616449	5,38815367	8,62670273	0,96571919	0,071317583	0,154114586	126	121	124	126	10	10	2	4	4	178,74
MS_run01	gi|23501323|ref|NP_697450.1|	fabI-1 gene product [Brucella s	0,22	0,32	0,85	1,23	3,82	1,45	0,71	41,33%	9,92E-03	1,17647059	3,105691057	0	0	0	0	0	0	0	0	0	0	0	0	1	4	4	273,08
MS_run02	gi|23501323|ref|NP_697450.1|	fabI-1 gene product [Brucella s	0,17	0,19	1,25	1,40	7,26	1,12				0,8	5,185714286	0	0	0	0	0	0	0	0	0	0	0	0	1	4	4	300,75
MS_run03	gi|23501323|ref|NP_697450.1|	fabI-1 gene product [Brucella s	0,78	0,56	0,85	0,74	1,09	0,87				1,17647059	1,779661017	0,08544004	0,22030282	0,100166528	0,11483088	0,28702323	0,106776796	10	20	14	10	16	19	3	6	6	340,38
MS_run01	gi|23501328|ref|NP_697455.1|	unnamed protein product [Bru	1,23	1,11	1,50	1,36	1,22	0,91	1,33	49,89%	8,02E-02	0,66666667	0,897058824	0	0	0	0	0	0	0	0	0	0	0	0	1	1	1	79,95
MS_run02	gi|23501328|ref|NP_697455.1|	unnamed protein product [Bru	2,05	1,76	2,11	1,81	1,03	0,86				0,47393365	0,569060773	0	0	0	0	0	0	0	0	0	0	0	0	1	2	2	105,15
MS_run03	gi|23501328|ref|NP_697455.1|	unnamed protein product [Bru	0,74	0,74	0,75	0,75	1,01	1,00				1,33333333	1,346666667	0	0	0	0	0	0	0	0	0	0	0	0	1	1	1	46,41
MS_run01	gi|23501332|ref|NP_697459.1|	xseB gene product [Brucella su	0,51	0,16	2,35	0,75	4,60	0,32	0,94	83,84%	1,84E-01	0,42553191	6,133333333	0	0	0	0	0	0	0	0	0	0	0	0	1	1	1	24,24
MS_run01	gi|23501340|ref|NP_697467.1|	unnamed protein product [Bru	1,41	1,22	1,11	0,96	0,79	0,86	1,00	19,14%	1,52E-01	0,9009009	0,822916667	0	0	0	0	0	0	0	0	0	0	0	0	1	1	1	51,52
MS_run02	gi|23501340|ref|NP_697467.1|	unnamed protein product [Bru	1,04	1,17	0,73	0,82	0,70	1,12				1,36986301	0,853658537	0	0	0	0	0	0	0	0	0	0	0	0	1	1	1	55,36
MS_run03	gi|23501340|ref|NP_697467.1|	unnamed protein product [Bru	0,93	1,00	0,78	0,84	0,84	1,08				1,28205128	1	0	0	0	0	0	0	0	0	0	0	0	0	1	1	1	52,45
MS_run01	gi|23501353|ref|NP_697480.1|	rplI gene product [Brucella suis	0,79	0,90	0,72	0,66	0,91	0,91	0,83	9,08%	3,09E-03	1,38888889	1,109064609	0,13982131	0,23983328	0,127279221	0,26421899	0,545641044	0,259757272	19	26	19	19	49	29	5	9	9	615,64
MS_run02	gi|23501353|ref|NP_697480.1|	rplI gene product [Brucella suis	0,91	1,00	0,85	0,81	0,93	0,95				1,18236194	1	0,12065792	0,2033388	0,142653426	0,1734438	0,359587352	0,287755325	14	22	18	15	36	29	6	8	8	580,53
MS_run03	gi|23501353|ref|NP_697480.1|	rplI gene product [Brucella suis	0,78	0,79	0,77	0,91	0,99	1,17				1,30252101	1,284885057	0,30590303	0,13228757	0,248756909	0,41958021	0,332827112	0,269930727	39	13	27	32	26	34	7	9	9	690,95
MS_run01	gi|23501355|ref|NP_697482.1|	rpsR gene product [Brucella su	0,86	0,92	0,68	0,73	0,79	1,07	0,91	22,88%	3,78E-01	1,47058824	1,082191781	0	0	0	0	0	0	0	0	0	0	0	0	1	1	1	47,18
MS_run02	gi|23501355|ref|NP_697482.1|	rpsR gene product [Brucella su	1,33	1,22	1,20	1,10	0,90	0,92				0,83333333	0,818181818	0	0	0	0	0	0	0	0	0	0	0	0	1	1	1	47,18
MS_run03	gi|23501355|ref|NP_697482.1|	rpsR gene product [Brucella su	0,73	0,80	0,66	0,72	0,91	1,09				1,57630762	1,2765625	0,18384776	0,03535534	0,113137085	0,43909186	0,249697082	0,156213228	28	4	16	28	20	20	2	2	2	70,79
MS_run01	gi|23501356|ref|NP_697483.1|	rpsF gene product [Brucella sui	0,92	0,96	0,76	0,80	0,83	1,05	0,85	13,29%	4,47E-03	1,31578947	1,0375	0	0	0	0	0	0	0	0	0	0	0	0	1	4	3	323,23
MS_run02	gi|23501356|ref|NP_697483.1|	rpsF gene product [Brucella sui	0,85	0,98	1,04	1,04	1,22	1,00				0,9672619	1,215029762	0,11313708	0,57275649	0,113137085	0,10522422	0,68290521	0,549348856	11	47	11	11	56	56	2	5	4	226,08
MS_run03	gi|23501356|ref|NP_697483.1|	rpsF gene product [Brucella sui	0,62	0,66	0,77	0,80	1,25	1,03				1,30603303	1,617966752	0,12020815	0,19091883	0,169705627	0,20257525	0,581871117	0,237641057	16	15	21	16	36	36	2	5	4	227,55
MS_run01	gi|23501359|ref|NP_697486.1|	fabG gene product [Brucella su	1,02	0,96	0,98	0,92	0,96	0,94	0,95	20,05%	2,79E-01	1,02040816	1,043478261	0	0	0	0	0	0	0	0	0	0	0	0	1	8	8	395,71
MS_run02	gi|23501359|ref|NP_697486.1|	fabG gene product [Brucella su	1,03	1,20	1,13	1,32	1,10	1,17				0,88495575	0,833333333	0	0	0	0	0	0	0	0	0	0	0	0	1	8	8	467,65
MS_run03	gi|23501359|ref|NP_697486.1|	fabG gene product [Brucella su	0,77	0,62	0,82	0,67	1,07	0,82				1,2195122	1,611940299	0,12858201	0,04932883	0,128970281	0,24514456	0,431557044	0,145124067	16	5	19	20	27	23	3	8	8	499,5
MS_run01	gi|23501360|ref|NP_697487.1|	acpP gene product [Brucella su	0,96	0,91	1,00	0,93	1,04	0,93	0,89	12,16%	1,64E-02	1,01241351	1,103092784	0,15684387	0,26737926	0,097082439	0,14367604	0,221480585	0,229902439	16	26	10	14	20	25	5	4	4	696,69
MS_run02	gi|23501360|ref|NP_697487.1|	acpP gene product [Brucella su	0,90	0,96	1,04	1,03	1,15	0,99				0,97000937	1,049282297	0,08421203	0,12230427	0,16663333	0,07953798	0,158838001	0,13538117	8	11	16	8	15	14	4	4	4	577,61
MS_run03	gi|23501360|ref|NP_697487.1|	acpP gene product [Brucella su	0,72	0,62	0,87	0,79	1,21	0,90				1,15186019	1,623598553	0,06291529	0,33688524	0,073654599	0,08603797	0,311457496	0,134688603	7	28	9	7	19	22	4	4	4	442,6
MS_run01	gi|23501367|ref|NP_697494.1|	unnamed protein product [Bru	0,91	0,87	0,79	0,76	0,87	0,96	0,83	6,35%	7,53E-04	1,22114669	1,154266769	0,07549834	0,03785939	0,1530795	0,52199353	0,177651944	0,159829894	10	4	20	43	15	18	4	3	3	172,64
MS_run02	gi|23501367|ref|NP_697494.1|	unnamed protein product [Bru	0,73	0,89	0,73	0,92	0,99	1,27				1,2345679	1,127906977	0,12020815	0,02828427	0,084852814	0,60200205	0,056965568	0,048944016	17	3	9	49	5	6	3	2	2	129,68
MS_run03	gi|23501367|ref|NP_697494.1|	unnamed protein product [Bru	0,87	0,82	0,81	0,84	0,93	1,04				1,20551924	1,219178082	0,29512709	0,14	0,105039675	0,041081	0,065937426	0,048759222	36	15	13	3	5	6	3	3	3	113,15
MS_run01	gi|23501379|ref|NP_697506.1|	unnamed protein product [Bru	1,02	1,23	1,11	1,19	1,08	1,08	1,01	11,10%	3,20E-01	0,86956522	0,8125	0,06363961	0,22501852	0,262868789	0,18344866	0,072258252	0,103190856	6	21	22	21	9	8	3	2	2	169,57
MS_run02	gi|23501379|ref|NP_697506.1|	unnamed protein product [Bru	0,91	0,84	0,97	0,90	1,07	0,93				1,03798823	1,201636905	0,14605935	0,1931105	0,170587221	0,15942148	0,359520167	0,253364555	15	18	19	15	30	30	4	3	3	221,25
MS_run03	gi|23501379|ref|NP_697506.1|	unnamed protein product [Bru	0,96	1,04	0,96	1,04	1,00	1,08				1,04166667	0,962264151	0,12489996	0,05859465	0,052915026	0,15944568	0,08336423	0,091700065	13	6	5	15	9	9	3	2	2	155,63
MS_run01	gi|23501397|ref|NP_697524.1|	unnamed protein product [Bru	1,01	1,15	1,01	1,16	1,00	1,15	1,14	18,08%	2,64E-01	0,99564097	0,869230769	0,18463929	0,20966243	0,13820275	0,16545971	0,115660225	0,134075856	18	21	12	17	13	12	5	8	8	613,46
MS_run02	gi|23501397|ref|NP_697524.1|	unnamed protein product [Bru	0,86	1,34	1,07	1,55	1,24	1,45				0,82960856	0,751912514	0,21779195	0,0305505	0,305122926	0,25525222	0,143231306	0,225856144	20	2	20	31	19	17	4	9	9	612,08
MS_run03	gi|23501397|ref|NP_697524.1|	unnamed protein product [Bru	0,94	1,07	1,21	1,26	1,29	1,04				0,84965035	0,938596491	0,36390246	0,3361101	0,179698822	0,26187454	0,233600053	0,292620494	30	26	14	31	25	27	5	9	9	620,33
MS_run01	gi|23501417|ref|NP_697544.1|	gmd gene product [Brucella su	1,54	1,58	1,37	1,41	0,89	1,03	1,91	47,68%	7,50E-05	0,72992701	0,6328125	0,30932338	0,22501852	0,223069496	0,17992551	0,074981568	0,19179822	23	25	16	25	12	12	8	13	13	859,65
MS_run02	gi|23501417|ref|NP_697544.1|	gmd gene product [Brucella su	2,15	2,72	1,56	1,55	0,73	0,99				0,64102564	0,367741935	0,1724239	0,25487252	0,136674797	0,08542206	0,09528266	0,675644063	11	35	9	13	26	25	6	13	13	800,94
MS_run03	gi|23501417|ref|NP_697544.1|	gmd gene product [Brucella su	2,83	2,10	2,22	1,92	0,78	0,86				0,45045045	0,478396742	0,47234975	0,28891113	0,220310387	0,13333186	0,146899205	0,662985029	21	37	12	30	31	32	8	13	13	812,21
MS_run01	gi|23501436|ref|NP_697563.1|	xylA gene product [Brucella su	0,69	0,49	1,00	0,70	1,44	0,70	1,27	58,15%	2,32E-01	1	2,057142857	0	0	0	0	0	0	0	0	0	0	0	0	1	7	7	265,14
MS_run03	gi|23501436|ref|NP_697563.1|	xylA gene product [Brucella su	1,82	2,16	1,64	1,65	0,90	1,01				0,7014157	0,70569415	0,82731493	0,32526912	0,90509668	0,35491846	0,584236698	1,78481186	51	36	55	51	83	83	2	11	11	488,23
MS_run01	gi|23501451|ref|NP_697578.1|	unnamed protein product [Bru	1,72	1,18	0,62	0,43	0,36	0,69	0,99	39,08%	6,54E-02	1,61964472	0,851388889	0,05656854	0,01414214	0,035355339	0,14777571	0,104101832	0,144697527	9	4	8	9	12	12	2	2	2	127,53
MS_run02	gi|23501451|ref|NP_697578.1|	unnamed protein product [Bru	1,33	0,96	1,00	0,71	0,75	0,71				1,00505051	1,051073132	0,00707107	0,16970563	0,042426407	0,00714249	0,176214607	0,16177922	1	23	6	1	17	17	2	3	3	181,35
MS_run01	gi|23501455|ref|NP_697582.1|	unnamed protein product [Bru	0,76	0,75	0,87	0,87	1,15	1,00	0,77	9,93%	6,36E-05	1,14942529	1,337837838	0,02645751	0,10214369	0,078102497	0,0363439	0,010239288	0,005781231	3	9	9	3	1	1	3	6	6	445,48
MS_run02	gi|23501455|ref|NP_697582.1|	unnamed protein product [Bru	0,68	0,52	0,77	0,67	1,13	0,87				1,2987013	1,940298507	0,0305505	0,14106736	0,195533458	0,05318107	0,551695797	0,149798281	4	12	29	4	28	29	3	6	6	410,01
MS_run03	gi|23501455|ref|NP_697582.1|	unnamed protein product [Bru	0,84	0,79	0,84	0,84	1,00	1,00				1,05395369	1,264367816	0,26006409	0,06429101	0,107857931	0,19307197	0,140765046	0,079945169	31	6	13	18	11	10	3	6	6	488,02
MS_run01	gi|23501458|ref|NP_697585.1|	unnamed protein product [Bru	0,63	0,55	0,59	0,52	0,94	0,88	0,65	15,85%	1,26E-02	1,69491525	1,807692308	0	0	0	0	0	0	0	0	0	0	0	0	1	3	3	280,29
MS_run03	gi|23501458|ref|NP_697585.1|	unnamed protein product [Bru	0,64	0,50	1,01	0,79	1,57	0,78				0,99009901	1,987341772	0	0	0	0	0	0	0	0	0	0	0	0	1	3	3	324,78
MS_run01	gi|23501490|ref|NP_697617.1|	unnamed protein product [Bru	1,97	1,67	1,26	1,07	0,64	0,85	1,11	48,07%	2,42E-01	0,79365079	0,598130841	0	0	0	0	0	0	0	0	0	0	0	0	1	1	1	59,05
MS_run03	gi|23501490|ref|NP_697617.1|	unnamed protein product [Bru	0,73	0,45	1,08	0,67	1,48	0,62				0,92592593	2,208955224	0	0	0	0	0	0	0	0	0	0	0	0	1	1	1	39,68
MS_run01	gi|23501498|ref|NP_697625.1|	unnamed protein product [Bru	0,85	0,86	0,87	0,84	1,03	0,97	0,73	9,23%	4,73E-06	1,14942529	1,156626506	0,07496666	0,12576433	0,151756384	0,10963258	0,254865238	0,179175498	9	12	18	10	22	21	6	10	10	410,12
MS_run02	gi|23501498|ref|NP_697625.1|	unnamed protein product [Bru	0,64	0,68	0,62	0,62	0,98	1,00				1,6166884	1,487110187	0,10688779	0,19956202	0,076321688	0,25569249	0,285873864	0,14984939	17	20	12	16	19	22	4	6	6	290,47
MS_run03	gi|23501498|ref|NP_697625.1|	unnamed protein product [Bru	0,69	0,70	0,73	0,70	1,06	0,96				1,25964415	1,426017029	0,12529964	0,15821926	0,193132079	0,37729841	0,166390055	0,074814678	17	15	28	30	12	11	4	6	6	290,64
MS_run01	gi|23501508|ref|NP_697635.1|	unnamed protein product [Bru	1,36	1,63	1,02	1,00	0,75	0,98	1,31	18,87%	1,68E-03	0,98039216	0,612903226	0,12503333	0,14422205	0,148492424	0,12374131	0,133785384	0,259868372	12	19	15	13	22	16	3	6	6	425,25
MS_run02	gi|23501508|ref|NP_697635.1|	unnamed protein product [Bru	1,20	1,52	1,14	1,44	0,95	1,26				0,87719298	0,659722222	0	0	0	0	0	0	0	0	0	0	0	0	1	4	4	330,71
MS_run03	gi|23501508|ref|NP_697635.1|	unnamed protein product [Bru	1,38	1,47	1,23	1,30	0,89	1,06				0,81349206	0,688211575	0,04242641	0,08485281	0,084852814	0,02805979	0,110191925	0,235672368	3	10	7	3	16	16	2	5	5	291,68
MS_run01	gi|23501522|ref|NP_697649.1|	unnamed protein product [Bru	1,26	1,15	1,10	1,00	0,87	0,91	1,13	9,52%	2,28E-02	0,90909091	0,87	0	0	0	0	0	0	0	0	0	0	0	0	1	4	4	256,42
MS_run01	gi|23501526|ref|NP_697653.1|	omp2b gene product [Brucella	1,02	1,25	0,85	0,89	0,83	1,05	1,53	76,33%	2,78E-02	1,18347339	0,798076923	0,10647378	0,10260883	0,167715746	0,14106008	0,168460247	0,230770553	13	12	19	12	21	18	9	14	14	1320,13
MS_run02	gi|23501526|ref|NP_697653.1|	omp2b gene product [Brucella	1,43	1,34	1,09	1,02	0,76	0,94				0,91866835	0,749128074	0,23998512	0,14678783	0,130805199	0,2068456	0,153207395	0,241143161	22	19	13	23	20	18	9	11	11	1034,61
MS_run03	gi|23501526|ref|NP_697653.1|	omp2b gene product [Brucella	3,63	2,43	1,73	1,63	0,48	0,94				0,57983193	0,411636829	0,30620955	0,16431677	0,222849511	0,13894893	0,101457954	1,032835547	18	35	14	24	25	43	8	14	14	1242,6
MS_run01	gi|23501533|ref|NP_697660.1|	dapA gene product [Brucella su	0,91	0,96	0,95	1,00	1,04	1,05	1,08	13,56%	2,77E-01	1,05263158	1,04	0	0	0	0	0	0	0	0	0	0	0	0	1	3	3	152,27
MS_run02	gi|23501533|ref|NP_697660.1|	dapA gene product [Brucella su	1,01	1,25	1,02	1,26	1,01	1,24				0,98039216	0,801587302	0	0	0	0	0	0	0	0	0	0	0	0	1	3	3	175,14
MS_run03	gi|23501533|ref|NP_697660.1|	dapA gene product [Brucella su	1,22	1,32	0,99	1,07	0,81	1,08				1,01010101	0,757009346	0	0	0	0	0	0	0	0	0	0	0	0	1	2	2	147,17
MS_run01	gi|23501536|ref|NP_697663.1|	unnamed protein product [Bru	1,06	1,18	0,88	0,98	0,83	1,11	1,23	32,53%	4,25E-02	1,13636364	0,846938776	0	0	0	0	0	0	0	0	0	0	0	0	1	3	3	176,68
MS_run02	gi|23501536|ref|NP_697663.1|	unnamed protein product [Bru	1,86	1,68	1,17	1,06	0,63	0,91				0,85470085	0,594339623	0	0	0	0	0	0	0	0	0	0	0	0	1	3	3	169,53
MS_run03	gi|23501538|ref|NP_697665.1|	rpoZ gene product [Brucella su	0,50	0,58	0,51	0,59	1,02	1,16	0,54	4,00%	2,80E-04	1,96078431	1,728813559	0	0	0	0	0	0	0	0	0	0	0	0	1	2	2	158,83
MS_run01	gi|23501542|ref|NP_697669.1|	hemN-1 gene product [Brucell	0,43	0,50	0,34	0,40	0,80	1,18	0,56	14,37%	2,18E-04	2,94117647	2	0	0	0	0	0	0	0	0	0	0	0	0	1	9	9	398,01
MS_run02	gi|23501542|ref|NP_697669.1|	hemN-1 gene product [Brucell	0,51	0,80	0,48	0,75	0,94	1,56				2,08333333	1,253333333	0	0	0	0	0	0	0	0	0	0	0	0	1	8	8	315,37
MS_run03	gi|23501542|ref|NP_697669.1|	hemN-1 gene product [Brucell	0,74	0,68	0,55	0,50	0,74	0,91				1,81818182	1,48	0	0	0	0	0	0	0	0	0	0	0	0	1	5	5	215,95
MS_run03	gi|23501571|ref|NP_697698.1|	unnamed protein product [Bru	0,79	0,83	0,79	0,83	1,00	1,05	0,81	2,00%	3,78E-04	1,26582278	1,204819277	0	0	0	0	0	0	0	0	0	0	0	0	1	2	2	59,4
MS_run01	gi|23501576|ref|NP_697703.1|	unnamed protein product [Bru	1,03	1,15	1,30	1,45	1,26	1,12	1,31	19,24%	5,44E-03	0,74074074	0,865853659	0,1421267	0,27376998	0,201565303	0,26891618	0,203853365	0,244842546	11	22	14	36	24	21	7	14	14	1118,82
MS_run02	gi|23501576|ref|NP_697703.1|	unnamed protein product [Bru	1,64	1,61	1,40	1,40	0,85	1,00				0,71892393	0,605054046	0,20165978	0,34993809	0,219371396	0,12043123	0,100871974	0,179178237	14	41	16	17	17	11	7	16	16	1055,94
MS_run03	gi|23501576|ref|NP_697703.1|	unnamed protein product [Bru	1,19	1,08	1,37	1,13	1,15	0,82				0,73183761	0,928571429	0,09983319	0,17897858	0,198725103	0,05404317	0,348175731	0,586626765	7	15	18	7	37	54	5	12	12	756,64
MS_run01	gi|23501581|ref|NP_697708.1|	ndk gene product [Brucella sui	0,73	0,74	0,70	0,71	0,96	1,01	0,69	6,62%	2,06E-05	1,42857143	1,350877193	0,090185	0,14294521	0,115902258	0,18145923	0,022522209	0,012696526	13	15	16	13	2	2	3	7	7	367,83
MS_run02	gi|23501581|ref|NP_697708.1|	ndk gene product [Brucella sui	0,72	0,56	0,76	0,59	1,06	0,78				1,31578947	1,771929825	0,16258331	0,07071068	0,115902258	0,25440108	0,311855223	0,130554599	21	7	20	19	18	23	3	6	6	364,45
MS_run03	gi|23501581|ref|NP_697708.1|	ndk gene product [Brucella sui	0,72	0,64	0,76	0,60	1,06	0,79				1,31670132	1,594748541	0,15599145	0,10472185	0,113541476	0,29221362	0,326988602	0,115089143	21	10	19	22	21	18	4	6	6	363,45
MS_run01	gi|23501588|ref|NP_697715.1|	omp25 gene product [Brucella	1,46	1,64	1,30	1,38	0,89	1,07	2,60	147,65%	7,26E-03	0,77221228	0,609756098	0,7391066	0,49141014	0,137805937	0,17676888	0,047239916	0,135363535	57	55	10	23	8	8	11	7	7	755,19
MS_run02	gi|23501588|ref|NP_697715.1|	omp25 gene product [Brucella	2,73	2,19	1,81	1,54	0,66	0,85				0,55402087	0,457255747	0,19449109	0,19652941	0,138544016	0,0580782	0,108754097	0,60478456	11	30	9	10	24	28	9	6	6	673,64

MS_run03	gi|23501588|ref|NP_697715.1|	omp25 gene product [Brucella	5,90	5,37	2,83	3,03	0,48	1,07				0,35335689	0,186046512	0,22662114	0,17164471	0,34494444	0,02822842	0,06327545	2,12704707	8	36	11	8	34	40	9	6	6	574,31
MS_run02	gi|23501608|ref|NP_697735.1|	unnamed protein product [Bru	0,50	0,39	0,65	0,50	1,29	0,77	0,51	9,32%	2,19E-02	1,53846154	2,58	0	0	0	0	0	0	0	0	0	0	0	0	1	1	1	28,35
MS_run01	gi|23501628|ref|NP_697755.1|	unnamed protein product [Bru	0,89	2,27	0,67	1,70	0,75	2,54	1,15	50,68%	3,23E-01	1,49253731	0,441176471	0	0	0	0	0	0	0	0	0	0	0	0	1	8	8	332,88
MS_run03	gi|23501628|ref|NP_697755.1|	unnamed protein product [Bru	0,90	0,90	0,95	0,95	1,06	1,00				1,05263158	1,115789474	0	0	0	0	0	0	0	0	0	0	0	0	1	9	9	375,4
MS_run01	gi|23501634|ref|NP_697761.1|	unnamed protein product [Bru	0,76	0,84	0,67	0,74	0,88	1,11	0,87	24,53%	4,85E-01	1,53508772	1,204164311	0,13435029	0,09192388	0,183847763	0,31013455	0,17494464	0,121937379	20	11	25	20	15	15	2	3	3	135,66
MS_run02	gi|23501634|ref|NP_697761.1|	unnamed protein product [Bru	1,51	1,28	0,81	0,68	0,54	0,84				1,2347561	0,81401288	0,01414214	0,03535534	0,155563492	0,02155813	0,238214743	0,375588581	2	7	23	2	29	29	2	2	2	81,67
MS_run03	gi|23501634|ref|NP_697761.1|	unnamed protein product [Bru	0,84	0,83	0,77	0,76	0,92	0,99				1,2987013	1,210526316	0	0	0	0	0	0	0	0	0	0	0	0	1	2	2	87,55
MS_run01	gi|23501652|ref|NP_697779.1|	glyA gene product [Brucella su	1,54	1,40	1,38	1,45	0,89	1,05	1,91	65,33%	1,50E-03	0,73006265	0,714285714	0,15080735	0,29552214	0,303832661	0,07687478	0,083651422	0,177732179	11	33	21	11	12	13	8	11	11	419,04
MS_run02	gi|23501652|ref|NP_697779.1|	glyA gene product [Brucella su	1,59	2,20	1,64	1,56	1,03	0,95				0,6097561	0,45508982	0,34638267	0,32275378	0,283011021	0,12001915	0,138413365	0,445225862	21	31	18	20	30	20	8	10	10	445,46
MS_run03	gi|23501652|ref|NP_697779.1|	glyA gene product [Brucella su	3,78	2,51	1,96	1,92	0,52	0,98				0,50917289	0,398963731	0,19137659	0,41007113	0,072341781	0,0450959	0,137006256	0,654670482	10	79	4	9	34	26	5	8	8	293,26
MS_run03	gi|23501659|ref|NP_697786.1|	unnamed protein product [Bru	0,87	0,98	0,73	0,82	0,84	1,12	0,85	8,89%	1,58E-01	1,36986301	1,024390244	0	0	0	0	0	0	0	0	0	0	0	0	1	1	1	71,2
MS_run01	gi|23501677|ref|NP_697804.1|	rpsI gene product [Brucella sui:	0,74	0,80	0,83	0,88	1,12	1,06	0,84	13,34%	3,03E-03	1,22635934	1,246770944	0,14863266	0,11458621	0,203666394	0,21974773	0,135715191	0,083065531	18	10	23	18	11	10	5	6	6	409,8
MS_run02	gi|23501677|ref|NP_697804.1|	rpsI gene product [Brucella sui:	1,08	0,80	1,04	1,05	0,96	1,01				0,96153846	1,253101737	0,27573538	0,24772969	0,184300479	0,24233847	0,305387574	0,143017594	27	26	18	25	24	18	5	5	5	405,42
MS_run03	gi|23501677|ref|NP_697804.1|	rpsI gene product [Brucella sui:	0,70	0,77	0,68	0,76	0,97	1,11				1,47058824	1,298701299	0,04658326	0,10457852	0,076941536	0,10642835	0,317867598	0,159229066	7	11	10	7	24	21	7	7	7	457,29
MS_run01	gi|23501678|ref|NP_697805.1|	rplM gene product [Brucella su	0,14	0,74	0,14	0,83	1,00	6,15	0,72	27,46%	3,92E-02	6,66666667	1,280981067	0,0212132	0,12055428	0,39551654	3,67602316	0,107709036	0,359116865	16	12	48	55	8	49	3	6	6	306,42
MS_run02	gi|23501678|ref|NP_697805.1|	rplM gene product [Brucella su	0,93	0,98	0,89	0,96	0,96	1,07				1,13058943	1,030229885	0,09899495	0,14142136	0,28991378	0,12575578	0,164666476	0,157152078	11	15	30	11	16	16	2	5	5	237,16
MS_run03	gi|23501678|ref|NP_697805.1|	rplM gene product [Brucella su	0,80	0,79	0,75	0,74	0,94	0,99				1,3245614	1,27027027	0,3493327	0,13228757	0	0,01240538	0,17876698	0,095112045	47	14	0	1	14	12	3	6	6	296,87
MS_run01	gi|23501690|ref|NP_697817.1|	nuoB gene product [Brucella su	0,98	0,91	0,84	0,78	0,86	0,93	0,88	7,35%	3,99E-01	1,19047619	1,102564103	0	0	0	0	0	0	0	0	0	0	0	0	1	2	2	112,04
MS_run01	gi|23501691|ref|NP_697818.1|	nuoC gene product [Brucella su	0,73	0,66	0,76	0,69	1,04	0,91	0,76	13,13%	6,95E-03	1,31578947	1,507246377	0	0	0	0	0	0	0	0	0	0	0	0	1	6	6	352,46
MS_run02	gi|23501691|ref|NP_697818.1|	nuoC gene product [Brucella su	0,64	0,57	0,76	0,67	1,18	0,88				1,31578947	1,76119403	0	0	0	0	0	0	0	0	0	0	0	0	1	6	6	351,7
MS_run03	gi|23501691|ref|NP_697818.1|	nuoC gene product [Brucella su	1,06	0,95	0,83	0,74	0,78	0,89				1,20481928	1,054054054	0	0	0	0	0	0	0	0	0	0	0	0	1	7	7	495,47
MS_run01	gi|23501692|ref|NP_697819.1|	nuoD gene product [Brucella su	0,80	21,25	1,17	31,02	1,46	26,51	13,56	1304,06%	4,88E-01	0,85470085	0,047066409	0	0	0	0	0	0	0	0	0	0	0	0	1	3	3	194,32
MS_run01	gi|23501693|ref|NP_697820.1|	nuoE gene product [Brucella su	0,60	0,54	0,63	0,57	1,05	0,90	0,59	14,56%	1,49E-05	1,58730159	1,842105263	0	0	0	0	0	0	0	0	0	0	0	0	1	3	3	216,68
MS_run02	gi|23501693|ref|NP_697820.1|	nuoE gene product [Brucella su	0,37	0,45	0,52	0,63	1,39	1,21				1,92307692	2,206349206	0	0	0	0	0	0	0	0	0	0	0	0	1	3	3	203,43
MS_run03	gi|23501693|ref|NP_697820.1|	nuoE gene product [Brucella su	0,51	0,49	0,89	0,86	1,74	0,97				1,12359551	2,023255814	0	0	0	0	0	0	0	0	0	0	0	0	1	2	2	172,12
MS_run01	gi|23501695|ref|NP_697822.1|	nuoG gene product [Brucella su	0,85	0,79	0,87	0,81	1,02	0,93	0,83	3,08%	9,81E-03	1,14942529	1,259259259	0	0	0	0	0	0	0	0	0	0	0	0	1	12	12	821,37
MS_run01	gi|23501717|ref|NP_697844.1|	rpsD gene product [Brucella su	0,96	1,09	1,09	1,19	1,13	1,09	1,37	39,16%	8,99E-02	0,91743119	0,920473621	0,17515707	0,19768662	0,225321992	0,12480821	0,180020496	0,187688431	16	17	19	14	20	17	6	10	10	520,03
MS_run02	gi|23501717|ref|NP_697844.1|	rpsD gene product [Brucella su	0,92	1,07	1,17	1,35	1,26	1,16				0,85876456	0,933333333	0,17289207	0,26975915	0,166523272	0,12840785	0,014815188	0,01740768	15	21	12	15	2	2	6	11	11	545,75
MS_run03	gi|23501717|ref|NP_697844.1|	rpsD gene product [Brucella su	1,68	2,12	1,87	1,87	1,11	1,00				0,36764706	0,506106988	0,70073771	0,05291503	0,789957805	0,24067127	0,215569039	0,744957331	37	5	42	65	43	35	4	8	8	433,88
MS_run01	gi|23501722|ref|NP_697849.1|	unnamed protein product [Bru	0,48	0,49	0,60	0,62	1,26	1,03	0,54	12,23%	1,09E-08	1,66666667	2,032258065	0	0	0	0	0	0	0	0	0	0	0	0	1	2	2	87,39
MS_run02	gi|23501722|ref|NP_697849.1|	unnamed protein product [Bru	0,42	0,64	0,54	0,83	1,26	1,54				2,0173454	1,576417163	0,20506097	0,22627417	0,28991378	0,7732314	0,279698047	0,114350303	38	18	35	38	18	18	2	3	3	129,9
MS_run03	gi|23501722|ref|NP_697849.1|	unnamed protein product [Bru	0,35	0,49	0,41	0,59	1,17	1,43				2,45215311	2,204863424	0,04242641	0,15556349	0,28991378	0,25374645	0,826763758	0,182926356	10	13	50	10	37	37	2	3	3	130,7
MS_run02	gi|23501724|ref|NP_697851.1|	purL gene product [Brucella su	0,68	0,61	1,50	1,34	2,20	0,89	1,03	39,22%	1,91E-01	0,66666667	1,641791045	0	0	0	0	0	0	0	0	0	0	0	0	1	2	2	50,21
MS_run02	gi|23501728|ref|NP_697855.1|	unnamed protein product [Bru	0,70	1,05	1,19	1,79	1,71	1,50	0,92	39,18%	5,68E-02	0,84033613	0,955307263	0	0	0	0	0	0	0	0	0	0	0	0	1	1	1	23
MS_run03	gi|23501728|ref|NP_697855.1|	unnamed protein product [Bru	0,61	0,84	0,50	0,69	0,82	1,38				2	1,188405797	0	0	0	0	0	0	0	0	0	0	0	0	1	1	1	24,72
MS_run01	gi|23501729|ref|NP_697856.1|	purC gene product [Brucella su	2,32	1,09	3,71	1,75	1,60	0,47	2,22	96,43%	1,30E-01	0,26954178	0,914285714	0	0	0	0	0	0	0	0	0	0	0	0	1	2	2	80,81
MS_run01	gi|23501730|ref|NP_697857.1|	unnamed protein product [Bru	1,15	0,90	1,09	0,98	0,95	0,89	0,90	14,70%	1,04E-01	0,91774017	1,105769231	0,11990274	0,11378342	0,262253058	0,10650754	0,151470017	0,144522454	11	12	27	12	14	16	6	8	8	540,68
MS_run02	gi|23501730|ref|NP_697857.1|	unnamed protein product [Bru	0,97	0,95	0,96	0,99	0,99	1,03				1,04166667	1,054347826	0,15571647	0,16091997	0,133345238	0,13558476	0,170407795	0,172877398	16	16	13	13	16	18	7	9	9	654,52
MS_run03	gi|23501730|ref|NP_697857.1|	unnamed protein product [Bru	0,70	0,71	0,71	0,75	1,02	1,06				1,4084507	1,425274725	0,08	0,14064139	0,071274119	0,13613602	0,278703418	0,193707408	11	14	10	10	20	27	6	8	8	572,73
MS_run01	gi|23501736|ref|NP_697863.1|	purB gene product [Brucella su	1,11	0,55	1,66	0,82	1,50	0,49	1,20	39,96%	4,79E-01	0,60240964	1,829268293	0	0	0	0	0	0	0	0	0	0	0	0	1	5	5	241,98
MS_run03	gi|23501736|ref|NP_697863.1|	purB gene product [Brucella su	0,97	1,36	1,31	1,83	1,35	1,40				0,76335878	0,737704918	0	0	0	0	0	0	0	0	0	0	0	0	1	3	3	134,29
MS_run01	gi|23501740|ref|NP_697867.1|	unnamed protein product [Bru	0,89	0,88	0,93	0,91	1,04	0,98	1,28	40,83%	9,97E-02	1,07526882	1,142857143	0	0	0	0	0	0	0	0	0	0	0	0	1	6	6	285,38
MS_run02	gi|23501740|ref|NP_697867.1|	unnamed protein product [Bru	1,81	1,94	1,39	1,49	0,77	1,07				0,71942446	0,516778523	0	0	0	0	0	0	0	0	0	0	0	0	1	6	6	264,97
MS_run01	gi|23501741|ref|NP_697868.1|	unnamed protein product [Bru	1,09	0,96	0,86	0,76	0,79	0,88	0,85	13,22%	4,48E-02	1,0318458	1,039473684	0,27537853	0,18083141	0,04163332	0,18518405	0,188730932	0,191337516	32	23	5	18	18	20	3	7	7	370,27
MS_run02	gi|23501741|ref|NP_697868.1|	unnamed protein product [Bru	0,95	0,83	0,99	0,86	1,04	0,87				1,01010101	1,209302326	0	0	0	0	0	0	0	0	0	0	0	0	1	5	5	337,37
MS_run03	gi|23501741|ref|NP_697868.1|	unnamed protein product [Bru	0,61	0,82	0,65	0,87	1,06	1,35				1,51515152	1,071560994	0,0212132	0,2	0,255799401	0,30858407	0,20764871	0,362556238	3	19	29	20	19	44	3	6	6	274,39
MS_run03	gi|23501754|ref|NP_697881.1|	unnamed protein product [Bru	0,90	1,52	0,75	1,26	0,83	1,68	1,11	30,05%	3,55E-01	1,33333333	0,658730159	0	0	0	0	0	0	0	0	0	0	0	0	1	3	3	99,42
MS_run01	gi|23501760|ref|NP_697887.1|	unnamed protein product [Bru	0,75	0,71	0,84	0,79	1,12	0,95	0,70	7,06%	1,54E-08	1,1965812	1,425047893	0,08485281	0,04242641	0,106066017	0,12087295	0,243491272	0,121677649	10	4	13	10	17	17	2	6	6	360,1
MS_run02	gi|23501760|ref|NP_697887.1|	unnamed protein product [Bru	0,58	0,62	0,68	0,72	1,17	1,06				1,49534781	1,647156863	0,09192388	0,14142136	0,049497475	0,20364174	0,311820226	0,117027239	14	12	7	14	19	19	2	7	7	513,78
MS_run03	gi|23501760|ref|NP_697887.1|	unnamed protein product [Bru	0,64	0,68	0,66	0,71	1,04	1,07				1,52777778	1,494262295	0,08485281	0,00707107	0,134350288	0,19641855	0,274728372	0,125156316	13	1	19	13	18	18	2	6	6	377,74
MS_run01	gi|23501763|ref|NP_697890.1|	argS gene product [Brucella su	1,24	1,28	0,98	1,01	0,79	1,03	2,03	85,62%	5,42E-03	1,02040816	0,782178218	0	0	0	0	0	0	0	0	0	0	0	0	1	3	3	270,96
MS_run02	gi|23501763|ref|NP_697890.1|	argS gene product [Brucella su	1,89	1,88	2,19	2,18	1,16	1,00				0,456621	0,532110092	0	0	0	0	0	0	0	0	0	0	0	0	1	5	5	249,19
MS_run03	gi|23501763|ref|NP_697890.1|	argS gene product [Brucella su	3,48	3,82	2,09	2,29	0,60	1,10				0,4784689	0,262008734	0	0	0	0	0	0	0	0	0	0	0	0	1	3	3	232,16
MS_run01	gi|23501771|ref|NP_697898.1|	serS gene product [Brucella sui	1,12	1,11	1,10	1,09	0,98	0,99	1,06	20,64%	1,48E-01	0,90909091	0,899082569	0	0	0	0	0	0	0	0	0	0	0	0	1	3	3	200,62
MS_run02	gi|23501771|ref|NP_697898.1|	serS gene product [Brucella sui	0,85	0,68	0,94	0,75	1,10	0,80				1,06382979	1,466666667	0	0	0	0	0	0	0	0	0	0	0	0	1	2	2	121,67
MS_run03	gi|23501771|ref|NP_697898.1|	serS gene product [Brucella sui	1,35	1,38	1,15	1,17	0,85	1,02				0,86956522	0,726495726	0	0	0	0	0	0	0	0	0	0	0	0	1	2	2	117,83
MS_run01	gi|23501776|ref|NP_697903.1|	unnamed protein product [Bru	1,16	1,00	1,15	0,99	0,99	0,86	2,12	122,79%	4,19E-02	0,86956522	1	0	0	0	0	0	0	0	0	0	0	0	0	1	4	4	252,28
MS_run02	gi|23501776|ref|NP_697903.1|	unnamed protein product [Bru	1,36	1,69	1,56	1,94	1,15	1,24				0,64102564	0,592783505	0	0	0	0	0	0	0	0	0	0	0	0	1	3	3	239,78
MS_run03	gi|23501776|ref|NP_697903.1|	unnamed protein product [Bru	2,88	2,71	4,67	4,39	1,62	0,94				0,21413276	0,369020501	0	0	0	0	0	0	0	0	0	0	0	0	1	3	3	184,19
MS_run01	gi|23501784|ref|NP_697911.1|	tig gene product [Brucella suis	0,86	0,80	0,88	0,90	1,02	1,02	0,95	9,18%	1,57E-02	1,13636364	1,255555556	0,12988456	0,12275993	0,266214199	0,16881774	0,142400043	0,100679539	15	12	30	15	11	13	6	13	13	688,46
MS_run02	gi|23501784|ref|NP_697911.1|	tig gene product [Brucella suis	0,95	0,88	1,01	1,01	1,06	1,00				0,99702381	1,142857143	0,15283433	0,07182154	0,132790562	0,1447811	0,13187261	0,118551927	15	7	13	15	12	14	5	13	13	720,48
MS_run03	gi|23501784|ref|NP_697911.1|	tig gene product [Brucella suis	0,89	1,06	1,07	1,10	1,21	1,02				0,94123856	0,97042289	0,37917894	0,20141168	0,45163776	0,2484421	0,298477615	0,349101417	35	17	41	26	31	33	6	15	15	727,77
MS_run01	gi|23501785|ref|NP_697912.1|	gatB gene product [Brucella su	0,91	0,96	1,06	1,07	1,16	1,00	1,00	6,44%	1,64E-01	0,94549996	1,018796992	0,12606215	0,54074023	0,462412154	0,10875668	0,268183703	0,092895197	12	47	43	12	26	10	4	6	6	353,26
MS_run01	gi|23501793|ref|NP_697920.1|	accB gene product [Brucella su	1,03	1,18	1,03	1,16	1,00	1,13	1,10	69,82%	3,04E-01	0,97581874	0,857758621	0,0212132	0,13435029	0	0,02019536	0,115819214	0,158864765	2	14	0	2	14	14	2	3	3	254,23
MS_run02	gi|23501793|ref|NP_697920.1|	accB gene product [Brucella su	2,43	1,21	2,50	1,21	1,03	0,48				0,53368984	0,845084375	1,76069589	0,23334524	0,028284271	0,37661944	0,173093093	0,247563377	71	23	2	71	20	20	2	4	4	270,52
MS_run03	gi|23501793|ref|NP_697920.1|	accB gene product [Brucella su	0,36	0,40	0,39	0,37	1,06	0,96				0,81716637	1,201403435	0,44547727	0,27319102	0,353553391	0,86465732	0,376659519	0,401715235	116	26	96	106	31	101	3	4	4	299,38
MS_run01	gi|23501795|ref|NP_697922.1|	unnamed protein product [Bru	1,58	1,50	0,38	0,36	0,24	0,95	1,40	153,12%	1,16E-01	2,63157895	0,666666667	0	0	0	0	0	0	0	0	0	0	0	0	1	3	3	139,86
MS_run02	gi|23501795|ref|NP_697922.1|	unnamed protein product [Bru	1,17	1,39	0,27	0,32	0,23	1,19				3,7037037	0,71875	0	0	0	0	0	0	0	0	0	0	0	0	1	2	2	112,8
MS_run03	gi|23501795|ref|NP_697922.1|	unnamed protein product [Bru	5,38	3,75	0,43	0,30	0,08	0,70				2,3255814	0,266666667	0	0	0	0	0	0	0	0	0	0	0	0	1	3	3	136,59
MS_run02	gi|23501808|ref|NP_697935.1|	bcp gene product [Brucella sui:	1,24	1,13	1,10	1,01	0,89	0,92	1,12	8,09%	1,88E-02	0,90909091	0,881188119	0	0	0	0	0	0	0	0	0	0	0	0	1	1	1	31,94
MS_run01	gi|23501815|ref|NP_697942.1|	unnamed protein product [Bru	1,27	1,17	1,24	1,15	0,98	0,93	1,12	45,78%	1,44E-01	0,80645161	0,852173913	0	0	0	0	0	0	0	0	0	0	0	0	1	3	3	110,35

MS_run03	gi|23501815|ref|NP_697942.1|	unnamed protein product [Bru	0,39	0,42	1,61	1,74	4,10	1,08				0,62111801	2,356321839	0	0	0	0	0	0	0	0	0	0	0	0	1	2	2	72,08
MS_run01	gi|23501816|ref|NP_697943.1|	unnamed protein product [Bru	1,13	1,36	0,90	1,09	0,80	1,21	1,02	13,30%	3,81E-01	1,11111111	0,733944954	0	0	0	0	0	0	0	0	0	0	0	0	1	3	3	124,55
MS_run02	gi|23501816|ref|NP_697943.1|	unnamed protein product [Bru	1,09	1,10	0,94	0,95	0,86	1,01				1,06382979	0,905263158	0	0	0	0	0	0	0	0	0	0	0	0	1	3	3	125,92
MS_run03	gi|23501816|ref|NP_697943.1|	unnamed protein product [Bru	0,87	0,89	0,97	0,99	1,11	1,02				1,03092784	1,121212121	0	0	0	0	0	0	0	0	0	0	0	0	1	3	3	127,81
MS_run01	gi|23501828|ref|NP_697955.1|	unnamed protein product [Bru	0,75	0,78	0,64	0,67	0,85	1,04	0,69	17,37%	3,12E-04	1,58730159	1,275728597	0,11313708	0,12727922	0,077781746	0,28059793	0,042181689	0,025932528	18	15	12	18	3	3	2	3	3	194,51
MS_run02	gi|23501828|ref|NP_697955.1|	unnamed protein product [Bru	0,76	0,92	0,84	1,00	1,10	1,19				1,19764773	1,103345462	0,00707107	0,12020815	0,035355339	0,01014209	0,160017393	0,132842095	1	11	4	1	15	15	2	3	3	161,28
MS_run03	gi|23501828|ref|NP_697955.1|	unnamed protein product [Bru	0,46	0,44	0,53	0,50	1,14	0,94				1,88679245	2,28	0	0	0	0	0	0	0	0	0	0	0	0	1	2	2	147,16
MS_run01	gi|23501829|ref|NP_697956.1|	unnamed protein product [Bru	1,03	0,97	1,10	1,13	1,07	1,03	0,88	14,97%	1,22E-02	0,90909091	1,030612245	0,09073772	0,20428738	0,100166528	0,08437728	0,159605294	0,140600925	8	19	9	9	15	14	3	8	8	425,47
MS_run02	gi|23501829|ref|NP_697956.1|	unnamed protein product [Bru	0,75	0,93	0,80	0,98	1,06	1,23				1,25	1,072992701	0,19857828	0,24269322	0,266645833	0,23630767	0,095703396	0,072825478	25	23	27	19	9	8	3	7	7	314,58
MS_run03	gi|23501829|ref|NP_697956.1|	unnamed protein product [Bru	0,74	0,77	0,70	0,71	0,95	1,01				1,42857143	1,29774758	0,06082763	0,07615773	0,047871355	0,10956511	0,138315541	0,075367032	9	8	7	8	11	10	4	8	8	419,95
MS_run01	gi|23501833|ref|NP_697960.1|	unnamed protein product [Bru	1,24	1,20	0,51	0,49	0,41	0,96	0,77	39,15%	4,05E-01	1,96078431	0,836734694	0	0	0	0	0	0	0	0	0	0	0	0	1	4	4	275,31
MS_run02	gi|23501833|ref|NP_697960.1|	unnamed protein product [Bru	1,08	1,42	0,26	0,34	0,24	1,31				3,84615385	0,705882353	0	0	0	0	0	0	0	0	0	0	0	0	1	3	3	223,97
MS_run03	gi|23501833|ref|NP_697960.1|	unnamed protein product [Bru	0,95	0,91	0,41	0,39	0,43	0,95				2,43902439	1,102564103	0	0	0	0	0	0	0	0	0	0	0	0	1	3	3	156,06
MS_run03	gi|23501834|ref|NP_697961.1|	unnamed protein product [Bru	0,78	1,00	0,49	0,49	0,63	1,00	0,69	21,56%	2,97E-01	2,51035197	1,116111111	0,28991378	0,6151829	0,332340187	1,50058892	0,503617163	0,450104714	60	98	69	60	45	45	2	1	1	50,38
MS_run01	gi|23501838|ref|NP_697965.1|	unnamed protein product [Bru	1,15	0,92	1,18	0,95	1,03	0,80	1,06	14,64%	4,40E-01	0,85241638	1,087669683	0,12727922	0,10606602	0,134350288	0,09194482	0,042394411	0,035862832	11	10	14	11	4	4	2	4	4	184,69
MS_run02	gi|23501838|ref|NP_697965.1|	unnamed protein product [Bru	1,09	1,20	1,09	1,20	1,00	1,10				0,91743119	0,833333333	0	0	0	0	0	0	0	0	0	0	0	0	1	2	2	157,28
MS_run03	gi|23501838|ref|NP_697965.1|	unnamed protein product [Bru	0,78	0,82	1,14	1,20	1,46	1,05				0,87719298	1,216666667	0	0	0	0	0	0	0	0	0	0	0	0	1	2	2	108,13
MS_run01	gi|23501843|ref|NP_697970.1|	unnamed protein product [Bru	1,68	1,25	1,78	1,33	1,06	0,75	1,82	34,72%	4,02E-05	0,56179775	0,796992481	0	0	0	0	0	0	0	0	0	0	0	0	1	4	4	186,11
MS_run02	gi|23501843|ref|NP_697970.1|	unnamed protein product [Bru	2,01	2,21	2,01	2,21	1,00	1,10				0,49751244	0,452488688	0	0	0	0	0	0	0	0	0	0	0	0	1	4	4	153,3
MS_run03	gi|23501843|ref|NP_697970.1|	unnamed protein product [Bru	1,90	2,36	1,39	1,72	0,73	1,24				0,71942446	0,424418605	0	0	0	0	0	0	0	0	0	0	0	0	1	3	3	141,64
MS_run01	gi|23501854|ref|NP_697981.1|	unnamed protein product [Bru	7,63	1,05	6,94	0,96	0,91	0,14	4,15	314,74%	1,28E-01	0,14409222	0,947916667	0	0	0	0	0	0	0	0	0	0	0	0	1	4	3	219,81
MS_run01	gi|23501876|ref|NP_698003.1|	metG gene product [Brucella s	0,77	0,86	0,70	0,78	0,91	1,11	1,18	65,05%	2,68E-01	1,42857143	1,166666667	0	0	0	0	0	0	0	0	0	0	0	0	1	5	5	233,92
MS_run03	gi|23501876|ref|NP_698003.1|	metG gene product [Brucella s	1,95	2,57	0,78	1,03	0,40	1,32				1,28205128	0,388349515	0	0	0	0	0	0	0	0	0	0	0	0	1	6	6	356,43
MS_run01	gi|23501884|ref|NP_698011.1|	glnA gene product [Brucella su	1,38	1,37	1,17	1,21	0,85	1,03	1,17	14,31%	3,29E-03	0,85470085	0,73040293	0,15931401	0,18435322	0,247004273	0,11834505	0,172699007	0,304310554	14	22	20	14	24	22	9	11	11	727,01
MS_run02	gi|23501884|ref|NP_698011.1|	glnA gene product [Brucella su	1,28	1,33	1,14	1,15	0,89	1,01				0,87827427	0,755681818	0,19026298	0,23447814	0,1898157	0,15592018	0,159514563	0,225180064	17	26	16	18	21	17	6	8	8	514,78
MS_run03	gi|23501884|ref|NP_698011.1|	glnA gene product [Brucella su	1,01	1,06	0,94	1,00	0,93	1,06				1,06575964	0,949494949	0,16382918	0,1743918	0,128167023	0,20192111	0,216518918	0,273428227	17	19	13	19	23	26	8	10	10	659,49
MS_run01	gi|23501885|ref|NP_698012.1|	glnB gene product [Brucella su	0,84	0,95	0,85	0,83	1,01	0,98	0,70	14,37%	6,82E-04	1,18347339	1,055045872	0,10230673	0,1578607	0,236199069	0,14293755	0,273233949	0,169079797	12	16	29	12	26	18	6	4	4	319,05
MS_run02	gi|23501885|ref|NP_698012.1|	glnB gene product [Brucella su	0,73	0,63	0,83	0,65	1,14	0,78				1,20481928	1,375	0,21004761	0,16703293	0,214639232	0,28367984	0,364638035	0,292952167	25	15	33	24	27	46	7	6	6	336,89
MS_run03	gi|23501885|ref|NP_698012.1|	glnB gene product [Brucella su	0,55	0,56	0,52	0,53	0,93	1,02				1,94633409	1,780195682	0,08931219	0,08590693	0,110408333	0,3775217	0,369307419	0,09629062	17	9	21	19	21	17	7	7	7	417,5
MS_run01	gi|23501890|ref|NP_698017.1|	rpiA gene product [Brucella sui	1,05	1,03	0,84	0,82	0,80	0,98	0,98	15,89%	8,51E-02	1,19047619	0,975609756	0	0	0	0	0	0	0	0	0	0	0	0	1	4	4	222,79
MS_run03	gi|23501890|ref|NP_698017.1|	rpiA gene product [Brucella sui	1,28	1,13	0,91	0,80	0,71	0,88				1,0989011	0,8875	0	0	0	0	0	0	0	0	0	0	0	0	1	5	5	277,16
MS_run01	gi|23501891|ref|NP_698018.1|	unnamed protein product [Bru	1,82	1,70	1,55	1,65	0,85	1,07	1,66	55,92%	3,58E-03	0,60240964	0,549668874	0,41806812	0,17477877	0,290723236	0,06121776	0,05942855	0,510496624	27	21	18	10	11	30	7	7	7	545,07
MS_run02	gi|23501891|ref|NP_698018.1|	unnamed protein product [Bru	2,71	2,72	1,85	1,70	0,68	0,92				0,54585799	0,367332483	0,23678401	0,30021659	0,218013761	0,07003743	0,067891828	0,39099743	13	44	13	13	18	14	5	6	6	431,86
MS_run03	gi|23501891|ref|NP_698018.1|	unnamed protein product [Bru	1,12	1,08	1,01	1,04	0,90	1,03				0,99165439	0,927835052	0,17454703	0,17790447	0,138094171	0,21738999	0,10109835	0,120959033	17	20	13	22	11	11	6	5	5	622,67
MS_run02	gi|23501904|ref|NP_698031.1|	unnamed protein product [Bru	0,74	3,12	0,90	3,78	1,21	4,20	2,51	170,93%	4,04E-01	1,11111111	0,32010582	0	0	0	0	0	0	0	0	0	0	0	0	1	10	10	533,48
MS_run03	gi|23501904|ref|NP_698031.1|	unnamed protein product [Bru	1,06	5,27	0,87	4,32	0,82	4,97				1,14942529	0,189814815	0	0	0	0	0	0	0	0	0	0	0	0	1	11	11	720,34
MS_run01	gi|23501909|ref|NP_698036.1|	folB gene product [Brucella sui	1,45	0,92	1,09	0,69	0,75	0,63	1,21	34,95%	2,23E-03	0,91743119	1,086956522	0	0	0	0	0	0	0	0	0	0	0	0	1	2	2	92,41
MS_run02	gi|23501909|ref|NP_698036.1|	folB gene product [Brucella sui	1,91	1,41	1,11	0,82	0,58	0,74				0,9009009	0,707317073	0	0	0	0	0	0	0	0	0	0	0	0	1	1	1	41,04
MS_run03	gi|23501909|ref|NP_698036.1|	folB gene product [Brucella sui	1,67	1,38	1,10	0,91	0,66	0,83				0,90909091	0,725274725	0	0	0	0	0	0	0	0	0	0	0	0	1	2	2	62,31
MS_run01	gi|23501924|ref|NP_698051.1|	unnamed protein product [Bru	1,13	1,33	0,85	1,00	0,75	1,18	1,08	17,77%	2,98E-01	1,17647059	0,75	0	0	0	0	0	0	0	0	0	0	0	0	1	2	2	147,09
MS_run01	gi|23501925|ref|NP_698052.1|	unnamed protein product [Bru	1,43	1,68	0,97	1,14	0,68	1,18	1,25	29,09%	2,68E-02	1,03092784	0,596491228	0	0	0	0	0	0	0	0	0	0	0	0	1	8	8	482,41
MS_run02	gi|23501925|ref|NP_698052.1|	unnamed protein product [Bru	1,53	1,45	0,92	0,87	0,60	0,95				1,08695652	0,689655172	0	0	0	0	0	0	0	0	0	0	0	0	1	8	8	589,14
MS_run03	gi|23501926|ref|NP_698053.1|	unnamed protein product [Bru	1,46	1,16	1,34	1,07	0,92	0,80	1,26	15,17%	2,20E-02	0,90418354	0,867550872	0,79195959	0,1979899	0,296984848	0,53438569	0,055756603	0,074234239	59	22	28	59	6	6	2	5	5	266,25
MS_run01	gi|23501931|ref|NP_698058.1|	unnamed protein product [Bru	1,23	1,35	1,00	0,94	0,81	0,94	1,13	16,96%	8,23E-02	1,03745202	0,887626263	0,26870058	0,41012193	0,077781746	0,27876396	0,512473854	0,780537101	27	51	8	27	58	58	2	4	4	211,18
MS_run01	gi|23501933|ref|NP_698060.1|	unnamed protein product [Bru	0,81	0,77	0,70	0,66	0,86	0,94	0,74	5,94%	2,22E-02	1,42857143	1,303030303	0	0	0	0	0	0	0	0	0	0	0	0	1	3	3	176,31
MS_run01	gi|23501936|ref|NP_698063.1|	unnamed protein product [Bru	0,72	0,72	0,73	0,73	1,02	1,00	1,62	94,35%	4,07E-01	1,36986301	1,397260274	0	0	0	0	0	0	0	0	0	0	0	0	1	13	13	686,19
MS_run02	gi|23501936|ref|NP_698063.1|	unnamed protein product [Bru	1,03	1,08	2,76	2,88	2,67	1,04				0,36231884	0,927083333	0	0	0	0	0	0	0	0	0	0	0	0	1	13	13	637,03
MS_run03	gi|23501936|ref|NP_698063.1|	unnamed protein product [Bru	1,37	2,69	1,45	3,27	1,06	2,25				0,70170345	0,450637105	0,26870058	0,52325902	3,090056634	0,13003319	0,266228226	1,588141621	19	49	95	19	59	59	2	12	12	626,6
MS_run01	gi|23501971|ref|NP_698098.1|	unnamed protein product [Bru	0,99	0,94	1,00	1,02	1,01	1,02	0,95	6,43%	6,12E-02	1	1,063157895	0,16777962	0,11326959	0,131751028	0,21434385	0,214779364	0,195063368	17	11	13	21	20	21	5	8	8	511,78
MS_run02	gi|23501971|ref|NP_698098.1|	unnamed protein product [Bru	0,95	0,95	1,05	0,99	1,11	0,94				0,95238095	1,058534323	0,13553597	0,34677082	0,334858179	0,12197425	0,178798957	0,177107345	13	31	34	13	17	19	6	7	7	578,24
MS_run03	gi|23501971|ref|NP_698098.1|	unnamed protein product [Bru	0,90	0,97	0,80	0,89	0,89	1,11				1,25	1,034482759	0,07668116	0,04932883	0,071180522	0,10879512	0,188854384	0,154558075	10	6	8	9	18	16	6	8	8	557,93
MS_run01	gi|23501972|ref|NP_698099.1|	unnamed protein product [Bru	0,35	0,26	1,05	0,78	2,96	0,74	1,09	44,22%	8,70E-02	0,95238095	3,794871795	0	0	0	0	0	0	0	0	0	0	0	0	1	1	1	32,8
MS_run02	gi|23501972|ref|NP_698099.1|	unnamed protein product [Bru	0,84	1,37	1,65	1,56	1,97	0,94				0,60661765	1,320921544	0,07071068	1,76776695	0,134350288	0,02599657	1,25095379	1,299839407	4	90	9	4	95	95	2	2	2	74,15
MS_run03	gi|23501972|ref|NP_698099.1|	unnamed protein product [Bru	1,08	1,28	1,20	1,64	1,11	1,37				0,83333333	0,670005807	0,19078784	0,24041631	0,12858201	0,10560326	0,15624514	0,62222684	16	22	8	13	23	49	3	3	3	119,27
MS_run01	gi|23501980|ref|NP_698107.1|	unnamed protein product [Bru	0,83	0,83	1,00	1,00	1,20	1,00	0,92	10,07%	1,50E-01	1	1,2	0	0	0	0	0	0	0	0	0	0	0	0	1	5	5	210,81
MS_run02	gi|23501980|ref|NP_698107.1|	unnamed protein product [Bru	0,87	0,93	0,84	0,90	0,97	1,07				1,19047619	1,077777778	0	0	0	0	0	0	0	0	0	0	0	0	1	5	5	289,3
MS_run03	gi|23501980|ref|NP_698107.1|	unnamed protein product [Bru	1,14	1,04	0,87	0,79	0,76	0,91				1,14942529	0,962025316	0	0	0	0	0	0	0	0	0	0	0	0	1	6	6	329,06
MS_run01	gi|23501983|ref|NP_698110.1|	unnamed protein product [Bru	0,77	0,71	0,90	0,81	1,17	0,90	0,77	8,54%	3,31E-05	1,11111111	1,417721519	0,17953644	0,06557439	0,036055513	0,18125045	0,093471271	0,044248378	20	6	4	16	7	6	3	4	4	418,43
MS_run02	gi|23501983|ref|NP_698110.1|	unnamed protein product [Bru	0,76	0,78	0,78	0,80	1,03	1,02				1,34050546	1,288170027	0,24748737	0,04242641	0,014142136	0,42262188	0,075804853	0,045761816	32	4	2	32	6	6	2	3	3	259,74
MS_run03	gi|23501983|ref|NP_698110.1|	unnamed protein product [Bru	0,70	0,56	0,88	0,84	1,25	0,95				1,13636364	1,69047619	0,11590226	0,26514147	0,165025251	0,14292038	0,514468621	0,039279284	13	21	20	13	30	7	3	4	4	403,79
MS_run03	gi|23501984|ref|NP_698111.1|	lon gene product [Brucella suis	1,34	1,20	1,55	1,39	1,16	0,90	1,37	12,59%	3,31E-02	0,64516129	0,834532374	0	0	0	0	0	0	0	0	0	0	0	0	1	7	7	386,25
MS_run01	gi|23501986|ref|NP_698113.1|	clpX gene product [Brucella sui	1,15	1,18	1,18	1,12	1,03	0,95	1,56	51,35%	3,56E-03	0,85137085	0,850708502	0,33090514	0,18669589	0,221617431	0,21562198	0,207954215	0,288237225	28	18	20	25	24	25	8	10	10	494,38
MS_run02	gi|23501986|ref|NP_698113.1|	clpX gene product [Brucella sui	1,72	1,49	1,15	1,13	0,67	0,98				0,8698283	0,672268908	0,13614943	0,12502	0,194473649	0,09157335	0,211119056	0,445945449	12	19	17	11	31	30	7	9	9	434,82
MS_run03	gi|23501986|ref|NP_698113.1|	clpX gene product [Brucella sui	2,82	2,13	2,00	1,71	0,71	0,86				0,5	0,468965517	0,34543968	0,14829186	0,391778606	0,09657275	0,053146016	0,223483813	17	21	23	19	11	10	7	9	9	439,39
MS_run01	gi|23501987|ref|NP_698114.1|	clpP gene product [Brucella sui	1,09	1,02	1,07	1,01	0,98	0,95	1,02	7,10%	3,19E-02	0,96205962	0,97972155	0,23334524	0,15556349	0,240416306	0,21079064	0,079185687	0,082767956	22	16	24	22	8	8	2	8	8	290,71
MS_run02	gi|23501987|ref|NP_698114.1|	clpP gene product [Brucella sui	1,11	0,98	1,17	1,00	1,05	0,85				0,85914454	1,069647945	0,04949747	0,19091883	0,120208153	0,03650256	0,321104758	0,293892491	4	18	12	4	30	30	2	8	8	313,38
MS_run03	gi|23501987|ref|NP_698114.1|	clpP gene product [Brucella sui	1,02	0,98	0,94	0,91	0,92	0,97				1,07101947	1,016304348	0,04949747	0,04242641	0,021213203	0,05669814	0,02305783	0,022329688	5	5	2	5	2	2	2	8	8	295,28
MS_run01	gi|23502004|ref|NP_698131.1|	lpdA-1 gene product [Brucella	1,76	1,97	1,32	1,28	0,75	0,97	1,84	61,59%	1,95E-03	0,75757576	0,510467836	0,52430907	0,19478468	0,358757114	0,55357725	0,136755994	0,5038716	40	26	28	73	27	26	10	11	11	565,07
MS_run02	gi|23502004|ref|NP_698131.1|	lpdA-1 gene product [Brucella	1,56	0,92	1,54	1,58	0,99	1,03				0,6333173	0,525107627	0,66515662	0,24131584	0,800645573	0,02267458	0,268816891	0,681570789	43	24	51	4	51	74	3	8	8	460,1
MS_run03	gi|23502004|ref|NP_698131.1|	lpdA-1 gene product [Brucella	3,28	2,53	2,00	2,37	0,61	1,19				0,5	0,4727844	0,39284857	0,59231185	2,346493128	0,10099289	0,246766703	1,587904715	20	97	99	20	52	63	5	11	11	558,82
MS_run01	gi|23502005|ref|NP_698132.1|	aceF gene product [Brucella su	1,88	1,44	1,77	1,44	0,94	0,81	1,62	18,14%	4,50E-08	0,56497175	0,696868916	0,30933172	0,21476454	0,262103442	0,09788656	0,166062862	0,311145918	17	23	18	17	24	22	10	13	13	640,32
MS_run02	gi|23502005|ref|NP_698132.1|	aceF gene product [Brucella su	1,69	1,51	1,48	1,40	0,88	0,95				0,66666667	0,661654135	0,29097537	0,11877349	0,273638042	0,12933527	0,153150379	0,384872319	20	14	20	19	23	25	9	12	12	598,26
MS_run03	gi|23502005|ref|NP_698132.1|	aceF gene product [Brucella su	1,98	1,59	1,74	1,54	0,88	0,88				0,57471264	0,629120879	0,23897931	0,18912864	0,131554805	0,10813447	0,110041367	0,290357998	14	21	9	19	17	18	10	12	12	620,76
MS_run01	gi|23502006|ref|NP_698133.1|	pdhB gene product [Brucella su	1,58	1,39	1,57	1,28	1,00	0,82	1,49	11,25%	6,55E-09	0,63694268	0,717948718	0,19908883	0,14855227	0,182836241	0,092018	0,218590064	0,386184388	13	15	14	14	30	28	11	15	15	678,72
MS_run02	gi|23502006|ref|NP_698133.1|	pdhB gene product [Brucella su	1,64	1,53	1,58	1,43	0,96	0,91				0,63569583	0,652847153	0,23440705	0,1918767	0,214786716	0,10043269	0,12022417	0,265052238	15	20	15	16	18	17	7	13	13	528,39
MS_run03	gi|23502006|ref|NP_698133.1|	pdhB gene product [Brucella su	1,55	1,42	1,56	1,30	1,01	0,83				0,64367057	0,704931973	0,32276755	0,19205189	0,345235829	0,12826841	0,157998549	0,315312154	21	19	27	20	22	22	10	15	15	685,57

MS_run01	gi|23502007|ref|NP_698134.1|	pdhA gene product [Brucella su	1,31	1,47	1,43	1,48	1,09	1,04	1,90	61,46%	2,03E-03	0,7037037	0,68	0,22969182	0,16479786	0,168424068	0,1249709	0,078764703	0,139704932	16	15	11	18	12	9	5	9	8	562,62
MS_run02	gi|23502007|ref|NP_698134.1|	pdhA gene product [Brucella su	1,56	1,53	1,69	1,65	1,09	0,97				0,59423347	0,671139307	0,15556349	0,06363961	0,261629509	0,05469884	0,145428242	0,330628979	9	6	16	9	22	22	2	7	6	604,85
MS_run03	gi|23502007|ref|NP_698134.1|	pdhA gene product [Brucella su	2,88	3,31	2,19	2,34	0,76	1,07				0,45670671	0,30125523	0,24494897	0,14059398	0,375088878	0,05834897	0,00559399	0,79074043	11	18	16	13	2	24	4	8	7	542,3
MS_run01	gi|23502010|ref|NP_698137.1|	eno gene product [Brucella sui	0,92	1,02	1,05	1,06	1,13	1,01	1,07	9,23%	1,07E-01	0,9574858	0,984251969	0,24206519	0,25189283	0,212916259	0,16028224	0,171710584	0,187588113	23	22	20	17	17	18	11	14	14	839,27
MS_run02	gi|23502010|ref|NP_698137.1|	eno gene product [Brucella sui	1,18	1,30	1,09	1,04	0,93	0,95				0,90916605	0,771742039	0,19420166	0,10457852	0,180138835	0,11424106	0,251016917	0,277804243	18	11	17	13	33	21	7	11	11	541,77
MS_run03	gi|23502010|ref|NP_698137.1|	eno gene product [Brucella sui	1,02	1,10	0,98	1,04	0,96	1,06				1,02040816	0,912774725	0,20209359	0,16561034	0,179304211	0,16462025	0,131360283	0,147669296	21	17	17	16	14	13	11	13	13	949,94
MS_run01	gi|23502011|ref|NP_698138.1|	kdsA gene product [Brucella su	1,03	1,12	0,92	0,99	0,89	1,08	0,98	9,28%	3,08E-01	1,1048056	0,913897597	0,13435029	0,03535534	0,16263456	0,16221962	0,1867885	0,228413742	15	4	17	15	20	20	2	7	7	474,23
MS_run02	gi|23502011|ref|NP_698138.1|	kdsA gene product [Brucella su	0,99	0,93	1,08	1,00	1,09	0,93				0,92592593	1,076923077	0,22368132	0,13650397	0,072111026	0,14833361	0,171843135	0,16541221	21	13	7	16	16	18	3	9	9	555,51
MS_run03	gi|23502011|ref|NP_698138.1|	kdsA gene product [Brucella su	1,06	0,83	1,00	0,79	0,94	0,79				1	1,227978832	0	0,18384776	0,304055916	0	0,23839807	0,161132559	0	20	38	0	19	19	2	8	8	533,47
MS_run01	gi|23502012|ref|NP_698139.1|	pyrG gene product [Brucella su	1,32	1,14	1,17	0,90	0,89	0,77	1,32	27,98%	1,40E-02	0,86898396	1,021885522	0,21213203	0,36062446	0,127279221	0,15755499	0,545210279	0,608749823	18	41	14	18	53	53	2	6	6	195,08
MS_run02	gi|23502012|ref|NP_698139.1|	pyrG gene product [Brucella su	1,59	1,86	1,18	1,38	0,74	1,17				0,84745763	0,536231884	0	0	0	0	0	0	0	0	0	0	0	0	1	3	3	104,05
MS_run01	gi|23502016|ref|NP_698143.1|	tpiA gene product [Brucella sui	0,59	0,93	0,51	0,80	0,86	1,57	0,65	19,19%	1,28E-03	1,96078431	1,075	0	0	0	0	0	0	0	0	0	0	0	0	1	4	4	442,94
MS_run02	gi|23502016|ref|NP_698143.1|	tpiA gene product [Brucella sui	0,61	0,39	0,61	0,39	1,00	0,64				1,63934426	2,564102564	0	0	0	0	0	0	0	0	0	0	0	0	1	4	4	310,59
MS_run03	gi|23502016|ref|NP_698143.1|	tpiA gene product [Brucella sui	0,53	0,85	0,62	1,00	1,17	1,61				1,61290323	1,17	0	0	0	0	0	0	0	0	0	0	0	0	1	4	4	365,47
MS_run01	gi|23502017|ref|NP_698144.1|	unnamed protein product [Bru	1,08	0,76	1,63	1,15	1,51	0,71	1,16	31,07%	4,51E-01	0,61349693	1,313043478	0	0	0	0	0	0	0	0	0	0	0	0	1	7	7	479,99
MS_run01	gi|23502025|ref|NP_698152.1|	gltX gene product [Brucella sui	0,65	0,87	0,68	0,91	1,05	1,34	1,10	58,65%	4,73E-01	0,74895294	1,153846154	0,4384062	0,40722639	0,424421174	0,36931914	0,136311766	0,104040619	64	39	47	49	12	12	3	6	6	413,78
MS_run02	gi|23502025|ref|NP_698152.1|	gltX gene product [Brucella sui	1,99	1,70	2,07	1,76	1,04	0,85				0,49061535	0,590180879	0,33234019	0,11313708	0,056568542	0,07895942	0,045313303	0,130478037	16	11	3	16	8	8	2	7	7	429,32
MS_run03	gi|23502025|ref|NP_698152.1|	gltX gene product [Brucella sui	0,43	1,18	0,50	0,54	1,16	1,08				1,21031746	0,838329605	0,19091883	0,47511402	0,120208153	0,53313607	0,008647689	0,380243704	39	41	22	44	1	32	3	5	5	519,93
MS_run01	gi|23502026|ref|NP_698153.1|	gltA gene product [Brucella sui	1,48	1,54	1,41	1,40	0,95	0,99	2,52	120,46%	3,24E-03	0,70921986	0,648044693	0,35749126	0,29928248	0,251455762	0,12545904	0,108774264	0,21335503	25	32	18	18	17	14	5	12	12	611,18
MS_run02	gi|23502026|ref|NP_698153.1|	gltA gene product [Brucella sui	2,58	2,13	1,93	1,77	0,75	0,92				0,53377329	0,476073345	0,5622203	0,16145175	0,518394959	0,18305115	0,099608548	0,563608062	29	22	29	34	21	26	5	12	12	547,88
MS_run03	gi|23502026|ref|NP_698153.1|	gltA gene product [Brucella sui	4,90	4,84	3,04	3,26	0,62	1,07				0,32988413	0,206725682	0,43085187	0,10626225	0,395769293	0,03866049	0,027687257	0,848655429	14	17	12	12	13	18	4	11	11	558,49
MS_run01	gi|23502032|ref|NP_698159.1|	unnamed protein product [Bru	1,09	1,10	0,99	0,89	0,90	0,90	0,92	10,50%	3,68E-01	1,01546392	0,908675799	0,12323419	0,11171392	0,122800651	0,10220629	0,100248956	0,12379501	13	12	14	10	11	11	8	31	31	2044,03
MS_run02	gi|23502032|ref|NP_698159.1|	unnamed protein product [Bru	1,00	0,93	0,93	0,85	0,93	0,91				1,07638889	1,070805107	0,11513191	0,10672617	0,093935135	0,14802038	0,136857196	0,126851027	12	11	11	14	13	14	8	32	32	1975,3
MS_run03	gi|23502032|ref|NP_698159.1|	unnamed protein product [Bru	0,79	0,89	0,81	0,77	1,02	0,96				1,24228395	1,122569444	0,15203383	0,21253151	0,227533357	0,29845847	0,160609168	0,135452983	19	21	30	24	14	15	9	32	32	2155,66
MS_run01	gi|23502037|ref|NP_698164.1|	frr gene product [Brucella suis	0,93	0,83	0,92	0,82	0,99	0,89	0,78	12,23%	5,80E-04	1,08811354	1,209876543	0,18275667	0,17455658	0,089274856	0,25919785	0,292438233	0,226377293	20	18	11	24	24	27	5	7	7	328,18
MS_run02	gi|23502037|ref|NP_698164.1|	frr gene product [Brucella suis	0,86	0,83	0,88	0,85	1,02	0,97				1,15955473	1,2	0,23041629	0,22129166	0,162265215	0,3741973	0,224220663	0,117028235	26	22	19	32	19	14	5	7	7	332,77
MS_run03	gi|23502037|ref|NP_698164.1|	frr gene product [Brucella suis	0,56	0,65	0,59	0,68	1,05	1,15				1,63934426	1,542372881	0,24984995	0,14696938	0,085146932	0,18688258	0,302350721	0,129666636	42	14	13	11	20	20	5	7	7	298,42
MS_run01	gi|23502039|ref|NP_698166.1|	tsf gene product [Brucella suis	0,83	0,77	0,76	0,66	0,92	0,86	0,68	8,71%	2,39E-06	1,31670132	1,306100218	0,13436683	0,11635196	0,124476682	0,25387957	0,236880282	0,130642639	18	13	19	19	18	17	12	14	14	806,29
MS_run02	gi|23502039|ref|NP_698166.1|	tsf gene product [Brucella suis	0,71	0,64	0,73	0,72	1,02	0,99				1,38253242	1,563380282	0,1091444	0,1174734	0,101192885	0,20167638	0,196055597	0,09574129	15	12	14	15	13	15	11	12	12	742,6
MS_run03	gi|23502039|ref|NP_698166.1|	tsf gene product [Brucella suis	0,62	0,50	0,62	0,58	1,00	0,94				1,61290323	2,015644955	0,05174725	0,15362291	0,093222724	0,13473701	0,334026916	0,088881283	8	15	16	8	17	18	10	13	13	706,37
MS_run01	gi|23502040|ref|NP_698167.1|	rpsB gene product [Brucella su	0,78	0,84	0,80	0,86	1,03	1,07	0,77	12,08%	1,98E-04	1,24343528	1,209219858	0,03535534	0,01414214	0,113137085	0,05461127	0,17552296	0,12131734	4	1	13	4	15	15	2	10	10	843,34
MS_run02	gi|23502040|ref|NP_698167.1|	rpsB gene product [Brucella su	0,83	0,81	0,94	0,93	1,13	0,98				1,06976215	1,263513514	0,09899495	0,05656854	0,261629509	0,11266069	0,296220408	0,19079075	11	5	28	11	23	23	2	9	9	782,41
MS_run03	gi|23502040|ref|NP_698167.1|	rpsB gene product [Brucella su	0,63	0,57	0,66	0,59	1,04	0,89				1,51515152	1,754098361	0,01527525	0,15373137	0,030550505	0,03570261	0,185980605	0,073626466	2	15	5	2	11	13	3	9	9	883,03
MS_run01	gi|23502041|ref|NP_698168.1|	unnamed protein product [Bru	2,00	1,70	1,28	1,79	0,64	1,40	1,77	16,81%	7,46E-07	0,76923077	0,583783784	0,02828427	0,05656854	0,387986254	0,14458034	0,145742012	0,015405376	2	9	22	19	25	1	3	2	2	234,28
MS_run02	gi|23502041|ref|NP_698168.1|	unnamed protein product [Bru	1,77	1,87	1,81	1,85	1,02	1,02				0,55255365	0,550363812	0,02828427	0,25455844	0,028284271	0,00863457	0,129184758	0,438574563	2	25	2	2	23	23	2	2	2	125,99
MS_run03	gi|23502041|ref|NP_698168.1|	unnamed protein product [Bru	1,78	1,77	1,69	1,90	0,95	1,12				0,60099573	0,534410703	0,57282924	0,21886069	0,254230866	0,15640656	0,139293586	0,13474079	34	23	13	26	26	8	4	3	3	265,07
MS_run01	gi|23502043|ref|NP_698170.1|	unnamed protein product [Bru	1,38	2,30	1,79	3,12	1,30	1,74	2,26	99,88%	2,00E-01	0,59287228	0,437823356	0,60811183	0,94045202	2,545584412	0,20141489	0,055789837	0,293425263	34	73	82	34	13	13	2	3	3	220,46
MS_run02	gi|23502043|ref|NP_698170.1|	unnamed protein product [Bru	1,10	1,74	2,63	4,17	2,39	1,59				0,38022814	0,573141487	0	0	0	0	0	0	0	0	0	0	0	0	1	2	2	202,15
MS_run03	gi|23502043|ref|NP_698170.1|	unnamed protein product [Bru	1,06	3,15	1,17	3,46	1,10	2,96				0,85470085	0,317919075	0	0	0	0	0	0	0	0	0	0	0	0	1	3	3	256,39
MS_run01	gi|23502046|ref|NP_698173.1|	unnamed protein product [Bru	1,00	0,79	1,09	0,87	1,10	0,79	0,81	15,16%	4,44E-02	0,9351407	1,266448266	0,21213203	0,2192031	0,176776695	0,18199385	0,00540513	0,003370041	19	20	20	19	0	0	2	1	1	53,12
MS_run03	gi|23502046|ref|NP_698173.1|	unnamed protein product [Bru	0,66	0,73	0,64	0,71	0,97	1,11				1,5625	1,366197183	0	0	0	0	0	0	0	0	0	0	0	0	1	1	1	26,08
MS_run01	gi|23502060|ref|NP_698187.1|	unnamed protein product [Bru	1,05	0,83	0,96	0,82	0,91	0,85	0,92	10,71%	1,92E-01	1,04575163	1,147621198	0,15564382	0,11757976	0,119023807	0,20428265	0,282162195	0,111613849	16	13	15	20	25	14	4	5	4	288,65
MS_run02	gi|23502060|ref|NP_698187.1|	unnamed protein product [Bru	1,01	0,83	0,94	0,71	0,93	0,76				1,06382979	1,209876543	0,090185	0,09848858	0,080829038	0,10578	0,163005512	0,101176768	10	11	11	10	13	12	3	2	2	370,73
MS_run03	gi|23502060|ref|NP_698187.1|	unnamed protein product [Bru	1,09	0,92	1,00	0,91	0,92	0,91				1	1,082474227	0,13160547	0,10812801	0,113247517	0,12813571	0,154516016	0,164744936	13	12	13	13	14	18	5	5	5	317,54
MS_run01	gi|23502068|ref|NP_698195.1|	unnamed protein product [Bru	0,71	1,83	0,61	1,41	0,87	2,31	1,28	59,46%	9,96E-02	1,63978495	0,978152493	0,01414214	0,20506097	1,117228714	0,03801649	0,920482992	1,726538505	2	24	79	2	94	94	2	3	2	164,98
MS_run02	gi|23502068|ref|NP_698195.1|	unnamed protein product [Bru	0,73	1,53	0,92	1,83	1,26	1,99				1,10240964	0,853730648	0,12020815	0,1767767	1,039446968	0,1448291	0,583114756	1,043429149	13	14	57	13	68	68	2	3	2	164,82
MS_run03	gi|23502068|ref|NP_698195.1|	unnamed protein product [Bru	0,71	2,24	0,67	2,14	0,95	3,19				1,5828018	0,764355715	0,22627417	0,0212132	1,97282792	0,534548	0,696360231	2,037446455	34	2	92	34	91	91	2	3	2	174,51
MS_run01	gi|23502069|ref|NP_698196.1|	unnamed protein product [Bru	1,28	0,34	1,15	0,31	0,90	0,27	0,77	44,58%	3,45E-01	0,86956522	2,903225806	0	0	0	0	0	0	0	0	0	0	0	0	1	1	1	22,79
MS_run01	gi|23502071|ref|NP_698198.1|	unnamed protein product [Bru	2,47	1,69	1,37	1,33	0,56	0,97	1,34	40,08%	1,43E-02	0,72992701	0,591240876	0,18212633	0,24879041	0,388529738	0,09917042	0,156260257	0,430019511	13	45	29	14	26	25	8	12	12	886,06
MS_run02	gi|23502071|ref|NP_698198.1|	unnamed protein product [Bru	1,19	1,26	1,21	1,06	1,02	0,88				0,82644628	0,794871795	0,14652645	0,11090537	0,230651252	0,10921205	0,225182318	0,316776912	12	11	22	13	28	25	5	10	10	639,21
MS_run03	gi|23502071|ref|NP_698198.1|	unnamed protein product [Bru	1,02	1,55	0,93	1,04	0,91	1,11				1,07526882	0,646017699	0,26782457	0,26381812	0,15340578	0,21418723	0,281604347	0,425952559	29	29	15	20	44	28	6	10	10	735,49
MS_run01	gi|23502076|ref|NP_698203.1|	unnamed protein product [Bru	1,00	0,80	1,13	1,02	1,14	0,90	1,29	30,54%	2,00E-02	0,54514728	1,137544803	2,24448955	0,16743158	0,129967945	0,54152168	0,269102081	0,101898064	199	15	13	99	24	13	4	12	12	828,77
MS_run02	gi|23502076|ref|NP_698203.1|	unnamed protein product [Bru	1,24	1,10	1,28	1,23	1,03	0,96				0,46948935	0,841931074	2,92428339	0,10598742	0,158219257	0,39842654	0,408954757	0,221754886	228	10	13	85	49	20	4	12	12	611,98
MS_run03	gi|23502076|ref|NP_698203.1|	unnamed protein product [Bru	1,67	1,84	1,66	1,55	1,00	0,93				0,54043927	0,490066225	0,34394767	0,15456929	0,38491341	0,29508611	0,075723965	0,448731246	21	16	25	55	15	24	4	12	12	823,37
MS_run01	gi|23502078|ref|NP_698205.1|	alaS gene product [Brucella sui	1,47	0,99	1,44	1,30	0,98	0,90	1,71	117,40%	4,71E-02	0,68506006	0,869230769	0,32393415	0,30664855	0,412593424	0,01327152	0,442970808	0,229040277	22	31	32	2	51	23	3	13	12	591,65
MS_run02	gi|23502078|ref|NP_698205.1|	alaS gene product [Brucella sui	1,79	2,21	1,14	0,93	0,64	0,81				0,90785474	0,744361349	0,27577164	0,48790368	0,16263456	0,22058202	0,658337902	1,951385815	24	77	18	24	88	88	2	11	10	576,18
MS_run03	gi|23502078|ref|NP_698205.1|	alaS gene product [Brucella sui	1,94	5,38	1,01	0,98	0,52	0,97				0,99019608	0,526293867	0,01414214	0,5939697	0,014142136	0,01386484	0,598496711	6,114216933	1	114	1	1	114	114	2	12	11	572,02
MS_run03	gi|23502084|ref|NP_698211.1|	unnamed protein product [Bru	1,79	1,58	1,41	1,17	0,78	0,83	1,49	22,79%	1,33E-02	0,72722567	0,66146789	0,28991378	0,30405592	0,113137085	0,15005889	0,195913989	0,468303175	21	39	10	21	30	30	2	5	5	382,68
MS_run01	gi|23502085|ref|NP_698212.1|	rplQ gene product [Brucella su	0,75	0,74	0,80	0,80	1,06	1,01	0,74	9,00%	6,45E-07	1,25791139	1,351869919	0,14279356	0,08342661	0,029154759	0,23467956	0,096286256	0,053039772	18	8	4	19	7	7	6	6	6	325,44
MS_run02	gi|23502085|ref|NP_698212.1|	rplQ gene product [Brucella su	0,78	0,75	0,87	0,86	1,12	0,99				1,14942529	1,350853965	0,11777096	0,06220932	0,108781126	0,16072998	0,240663663	0,120242757	14	6	13	14	18	16	5	5	5	334,08
MS_run03	gi|23502085|ref|NP_698212.1|	rplQ gene product [Brucella su	0,60	0,62	0,62	0,65	1,04	1,04				1,61290323	1,607843137	0,08820431	0,12806248	0,143805424	0,30909527	0,274097101	0,123735805	14	12	22	19	17	20	7	8	8	490,59
MS_run01	gi|23502086|ref|NP_698213.1|	rpoA gene product [Brucella su	0,73	0,72	0,77	0,72	1,06	0,94	0,82	7,95%	1,67E-05	1,30221546	1,386685893	0,13287839	0,15453155	0,090443352	0,21337142	0,247079191	0,162240636	17	15	13	16	18	22	6	15	15	1345,17
MS_run02	gi|23502086|ref|NP_698213.1|	rpoA gene product [Brucella su	0,79	0,90	0,92	0,97	1,17	1,05				1,08708496	1,114288503	0,07574079	0,19701946	0,081342896	0,07774818	0,177597309	0,140460617	8	17	8	7	16	16	6	15	15	1201,24
MS_run03	gi|23502086|ref|NP_698213.1|	rpoA gene product [Brucella su	0,81	0,81	0,84	0,90	1,03	1,08				1,19799139	1,232480534	0,05683309	0,21484878	0,07	0,08089088	0,083618344	0,061588578	7	21	8	7	7	8	6	15	15	1134,47
MS_run01	gi|23502087|ref|NP_698214.1|	rpsK gene product [Brucella su	0,75	0,81	0,86	0,93	1,15	1,09	1,04	24,38%	7,30E-02	1,17445055	1,238095238	0,07778175	0,06363961	0,127279221	0,10684306	0,101015254	0,066119076	9	6	14	9	8	8	2	4	4	251,94
MS_run02	gi|23502087|ref|NP_698214.1|	rpsK gene product [Brucella su	1,07	1,36	1,19	1,51	1,11	1,27				0,84401709	0,75051551	0,0212132	0,02828427	0,261629509	0,01510912	0,149263306	0,27033903	2	3	17	2	20	20	2	4	4	245,12
MS_run03	gi|23502087|ref|NP_698214.1|	rpsK gene product [Brucella su	0,74	1,09	0,90	1,32	1,21	1,47				1,11111111	0,914709172	0,10066446	0,140119	0,427122153	0,11973348	0,002768315	0,267680798	11	12	32	11	0	25	3	4	4	280,8
MS_run01	gi|23502088|ref|NP_698215.1|	rpsM gene product [Brucella su	0,85	0,93	0,80	0,95	0,95	1,18	0,99	16,47%	3,06E-01	1,19047619	1,07310705	0,04949747	0,07071068	0,028284271	0,53684088	0,084503789	0,084783888	6	7	3	45	8	9	3	5	5	319,78
MS_run02	gi|23502088|ref|NP_698215.1|	rpsM gene product [Brucella su	1,24	1,35	1,02	1,11	0,82	1,09				0,98524558	0,749710744	0,00707107	0,01414214	0,148492424	0,00686378	0,113546155	0,204359434	1	2	13	1	15	15	2	5	5	301,49
MS_run03	gi|23502088|ref|NP_698215.1|	rpsM gene product [Brucella su	0,85	1,04	0,79	0,98	0,94	1,23				1,26190476	0,977678571	0,06363961	0,01414214	0,197989899	0,10101525	0,183090149	0,194964586	8	2	20	8	19	19	2	5	5	327,11
MS_run01	gi|23502089|ref|NP_698216.1|	adk gene product [Brucella sui	1,13	0,80	0,90	0,73	0,80	0,81	0,88	10,46%	2,10E-01	1,09318996	1,264136905	0,09073772	0,20297783	0,042426407	0,48407329	0,216490871	0,162218031	10	25	6	44	17	20	4	6	6	345,33
MS_run02	gi|23502089|ref|NP_698216.1|	adk gene product [Brucella sui	0,95	0,89	0,98	0,92	1,03	0,94				1,02040816	1,119565217	0,03605551	0,12055428	0,06	0,03892638	0,206741608	0,159361857	4	12	7	4	18	18	3	6	6	544,94
MS_run03	gi|23502089|ref|NP_698216.1|	adk gene product [Brucella sui	0,83	0,83	0,78	0,78	0,94	1,00				1,23626374	1,204819277	0,04163332	0,15588457	0,069522179	0,5172803	0,332554671	0,186116719	5	17	9	42	28	22	5	7	7	635,32
MS_run01	gi|23502091|ref|NP_698218.1|	rplO gene product [Brucella su	0,79	0,85	0,79	0,85	1,00	1,08	0,78	12,06%	2,05E-04	1,27393703	1,175257732	0,11975421	0,13119112	0,221258575	0,16708524	0,213181419	0,142235128	15	13	26	13	18	17	9	9	9	541,05
MS_run02	gi|23502091|ref|NP_698218.1|	rplO gene product [Brucella su	0,93	0,75	0,99	0,89	1,06	0,90				1	1,298411122	0,06615638	0,23358082	0,135365467	0,15008969	0,26713346	0,114979011	7	22	15	15	21	15	7	8	8	413,98
MS_run03	gi|23502091|ref|NP_698218.1|	rplO gene product [Brucella su	0,59	0,61	0,67	0,70	1,14	1,04				1,49253731	1,631488631	0,06271629	0,15348336	0,114717043	0,12688402	0,240843487	0,093387791	9	13	17	9	15	15	9	9	9	447,27
MS_run01	gi|23502093|ref|NP_698220.1|	rpsE gene product [Brucella su	1,08	0,92	0,85	0,83	0,79	0,98	0,89	13,16%	2,10E-01	1,1845039	1,084337349	0,15607156	0,16920402	0,237423672	0,20227702	0,174836312	0,170536314	18	21	29	17	16	18	5	6	6	360,53
MS_run02	gi|23502093|ref|NP_698220.1|	rpsE gene product [Brucella su	1,12	0,91	1,02	0,99	0,91	0,97				0,98039216	1,101010101	0,13576941	0,18	0,266520793	0,16760875	0,210039712	0,201834538	13	20	27	17	19	22	3	5	5	264,41
MS_run03	gi|23502093|ref|NP_698220.1|	rpsE gene product [Brucella su	0,76	0,75	0,71	0,76	0,94	1,07				1,4084507	1,328947368	0,13107613	0,14909249	0,13183684	0,28731766	0,140574597	0,086253623	18	16	17	20	11	11	7	7	7	459,25
MS_run01	gi|23502094|ref|NP_698221.1|	rplR gene product [Brucella sui	0,77	1,03	0,80	0,82	1,04	1,03	0,81	15,37%	2,05E-02	1,25	0,971841901	0,07765307	0,225	0,171668285	0,10714703	0,191950652	0,25502606	10	22	21	9	20	25	5	5	5	295,23
MS_run02	gi|23502094|ref|NP_698221.1|	rplR gene product [Brucella sui	0,99	1,01	0,86	0,87	0,87	1,01				1,16294794	0,989528796	0,03095696	0,30446675	0,095350232	0,04038203	0,330188856	0,524903254	4	35	11	3	33	52	5	5	5	376,71
MS_run03	gi|23502094|ref|NP_698221.1|	rplR gene product [Brucella sui	0,68	0,53	0,73	0,58	1,08	0,79				1,36986301	1,879310345	0,16607227	0,14876155	0,168315181	0,383153	0,509365887	0,197254389	23	14	29	28	27	37	5	5	5	341,89
MS_run01	gi|23502095|ref|NP_698222.1|	rplF gene product [Brucella sui	0,76	0,75	0,98	1,06	1,29	1,09	0,90	10,57%	2,19E-02	1,04278075	1,329113924	0,24671171	0,16777962	0,407516871	0,27576004	0,302854628	0,265012325	25	13	38	26	23	35	5	6	6	329,46
MS_run02	gi|23502095|ref|NP_698222.1|	rplF gene product [Brucella sui	1,01	1,04	0,91	0,86	0,90	0,94				1,0989011	0,95890411	0,18520259	0,18520259	0,176776695	0,18158556	0,090743173	0,089600486	20	21	21	17	9	9	3	5	5	223,48
MS_run03	gi|23502095|ref|NP_698222.1|	rplF gene product [Brucella sui	0,86	0,76	0,97	0,87	1,12	0,90				0,98039216	1,310344828	0,22835645	0,14638989	0,17253985	0,20708414	0,198425373	0,12887312	24	13	20	21	15	17	7	8	8	454,49
MS_run01	gi|23502096|ref|NP_698223.1|	rpsH gene product [Brucella su	0,78	0,71	0,85	0,78	1,09	0,92	0,80	6,76%	1,12E-05	1,17647059	1,191489362	0,06110101	0,07767453	0,412906365	0,08981292	0,312791896	0,351358173	7	7	53	8	26	49	4	4	4	143,03
MS_run02	gi|23502096|ref|NP_698223.1|	rpsH gene product [Brucella su	0,71	0,80	0,69	0,78	0,97	1,13				1,44927536	1,243589744	0,07	0,06658328	0,086216781	0,14881148	0,213814501	0,139106796	10	7	11	10	17	17	3	4	4	138,56

MS_run03	gi|23502096|ref|NP_698223.1|	rpsH gene product [Brucella su	0,88	0,86	0,89	0,88	1,02	0,98				1,12487361	1,149425287	0,20566964	0,17748239	0,297825452	0,27859747	0,02625044	0,213184382	23	17	34	25	2	25	4	4	4	128,77
MS_run01	gi|23502097|ref|NP_698224.1|	rpsN gene product [Brucella su	0,93	1,24	0,80	1,07	0,86	1,34	1,05	21,19%	2,68E-01	1,25	0,803738318	0	0	0	0	0	0	0	0	0	0	0	0	1	2	2	95,66
MS_run02	gi|23502097|ref|NP_698224.1|	rpsN gene product [Brucella su	1,07	1,56	0,88	1,28	0,82	1,45				1,13636364	0,640625	0	0	0	0	0	0	0	0	0	0	0	0	1	2	2	79,51
MS_run03	gi|23502097|ref|NP_698224.1|	rpsN gene product [Brucella su	0,83	0,96	0,90	1,05	1,09	1,17				1,11111111	1,038095238	0	0	0	0	0	0	0	0	0	0	0	0	1	2	2	106,96
MS_run01	gi|23502098|ref|NP_698225.1|	rplE gene product [Brucella sui	0,85	1,02	0,79	0,72	0,93	0,91	1,03	19,26%	1,66E-01	1,26602564	0,979381443	0,1631768	0,10807405	0,13485795	0,23942131	0,259849297	0,188644746	21	12	19	19	27	18	6	7	7	396,79
MS_run02	gi|23502098|ref|NP_698225.1|	rplE gene product [Brucella sui	1,03	0,93	1,10	0,98	1,07	0,89				0,90909091	1,085585586	0,1933908	0,26642072	0,219165082	0,24369292	0,361997845	0,337322329	18	25	22	27	33	36	6	8	8	321,28
MS_run03	gi|23502098|ref|NP_698225.1|	rplE gene product [Brucella sui	1,45	1,18	1,11	1,21	0,77	1,09				0,90119347	0,844036697	0,10045729	0,2688711	0,093985815	0,09267708	0,200498487	0,467002205	9	35	8	10	24	39	5	7	7	295,3
MS_run01	gi|23502099|ref|NP_698226.1|	rplX gene product [Brucella sui	0,86	0,70	0,83	0,68	0,97	0,82	0,76	10,19%	1,08E-03	1,20481928	1,428571429	2,3884786	0,15755951	0,095350232	0,70622964	0,242376574	0,137379249	288	16	14	59	17	20	5	3	3	334,77
MS_run02	gi|23502099|ref|NP_698226.1|	rplX gene product [Brucella sui	0,86	0,80	0,93	0,86	1,08	0,92				1,07638889	1,255813953	0,06601767	0,18579559	0,152643375	0,07598789	0,092506064	0,053538652	7	17	18	7	7	7	5	4	4	267,28
MS_run03	gi|23502099|ref|NP_698226.1|	rplX gene product [Brucella sui	0,670	0,62	0,71	0,62	1,06	0,87				1,4084507	1,620726496	0,09291573	0,19841875	0,12239281	0,22976405	0,350731364	0,114428801	13	19	20	16	22	18	5	4	4	298,19
MS_run01	gi|23502100|ref|NP_698227.1|	rplN gene product [Brucella su	0,91	0,74	1,10	1,12	1,21	1,02	0,82	22,47%	4,97E-03	0,90909091	1,360663984	0,15524175	0,1174734	0,351468349	0,12717138	0,172407301	0,094236882	14	10	31	14	13	13	5	5	5	296,44
MS_run02	gi|23502100|ref|NP_698227.1|	rplN gene product [Brucella su	0,78	0,77	0,87	0,87	1,11	1,00				1,14942529	1,297619048	0,10583005	0,1835756	0,345398321	0,12225631	0,197095993	0,135050097	12	17	40	11	15	18	5	4	4	275,82
MS_run03	gi|23502100|ref|NP_698227.1|	rplN gene product [Brucella su	0,35	0,87	0,44	1,03	1,27	2,34				1,60365336	1,116504854	0,36665151	0,29137605	0,29143324	0,91398034	0,142409931	0,206812776	83	23	28	57	13	24	4	3	3	243,76
MS_run01	gi|23502101|ref|NP_698228.1|	rpsQ gene product [Brucella su	0,90	0,71	1,07	0,82	1,19	0,77	0,79	16,04%	2,40E-02	0,9097676	1,245098039	0,24433583	0,30287511	0,148436294	0,03508925	0,382145325	0,134141146	23	25	18	4	31	19	3	3	3	117,69
MS_run02	gi|23502101|ref|NP_698228.1|	rpsQ gene product [Brucella su	1,07	0,73	0,90	0,77	0,84	0,86				1,11111111	1,366666667	0,05291503	0,13316656	0,211266025	0,06097867	0,316493851	0,003742167	6	16	27	5	23	1	3	3	3	124,46
MS_run03	gi|23502101|ref|NP_698228.1|	rpsQ gene product [Brucella su	0,58	0,65	0,58	0,71	0,99	1,23				1,61290323	1,532258065	0,06363961	0,09451631	0,070945989	0,5043589	0,152598176	0,07443926	11	10	10	31	10	11	3	3	3	140,01
MS_run01	gi|23502102|ref|NP_698229.1|	rpmC gene product [Brucella su	0,90	0,81	1,04	0,93	1,15	0,89	0,91	22,81%	3,90E-01	0,96153846	1,23655914	0	0	0	0	0	0	0	0	0	0	0	0	1	1	1	41,99
MS_run02	gi|23502102|ref|NP_698229.1|	rpmC gene product [Brucella su	1,17	1,28	1,03	1,12	0,88	1,08				0,97834347	0,845524297	0,12727922	0,05656854	0,374766594	0,12089592	0,334926281	0,508370248	12	6	34	12	40	40	2	2	2	70,42
MS_run03	gi|23502102|ref|NP_698229.1|	rpmC gene product [Brucella su	0,80	0,54	0,75	0,51	0,94	0,68				1,33333333	1,843137255	0	0	0	0	0	0	0	0	0	0	0	0	1	1	1	44,32
MS_run01	gi|23502103|ref|NP_698230.1|	rplP gene product [Brucella sui	0,97	1,04	0,73	0,78	0,75	1,07	0,88	12,92%	4,29E-01	1,36986301	0,961538462	0	0	0	0	0	0	0	0	0	0	0	0	1	1	1	25,14
MS_run01	gi|23502105|ref|NP_698232.1|	rplV gene product [Brucella sui	0,98	0,94	0,84	0,91	0,86	1,08	0,86	13,65%	5,00E-02	1,19047619	1,076007326	0,21902055	0,18479719	0,220680765	0,24188447	0,273515596	0,304436125	26	21	24	20	25	32	6	6	6	330,54
MS_run02	gi|23502105|ref|NP_698232.1|	rplV gene product [Brucella sui	1,14	0,94	0,88	0,89	0,77	1,01				1,13871636	1,068493151	0,08421203	0,19824228	0,230144882	0,10876838	0,413692809	0,381772693	10	26	26	10	39	41	5	5	5	260,56
MS_run03	gi|23502105|ref|NP_698232.1|	rplV gene product [Brucella sui	0,66	0,69	0,67	0,79	1,02	1,17				1,52654363	1,457142857	0,23304506	0,30582675	0,095568475	0,78765373	0,297901787	0,189376542	35	30	12	52	20	28	6	6	6	369,28
MS_run01	gi|23502106|ref|NP_698233.1|	rpsS gene product [Brucella sui	0,84	0,69	0,94	0,79	1,13	0,84	0,80	15,35%	3,11E-02	1,06382979	1,455696203	0,07094599	0,03535534	0,055075705	0,08490541	0,0726429	0,032016624	8	3	7	8	5	5	3	3	3	116,57
MS_run02	gi|23502106|ref|NP_698233.1|	rpsS gene product [Brucella sui	1,01	0,97	1,00	0,88	0,99	0,88				1	1,027027027	0,04932883	0,12124356	0,135769412	0,05329297	0,065307087	0,056007546	5	12	15	5	6	6	3	3	3	99,12
MS_run03	gi|23502106|ref|NP_698233.1|	rpsS gene product [Brucella sui	0,66	0,63	0,65	0,56	0,99	0,86				1,53846154	0,793918919	0,08736895	0,5057997	0,110151411	0,18677975	0,769217512	0,161392865	13	51	20	12	97	25	3	3	3	108,69
MS_run01	gi|23502107|ref|NP_698234.1|	rplB gene product [Brucella sui	1,29	1,18	1,21	1,14	0,94	0,94	1,52	31,99%	1,30E-04	0,82988981	0,845555171	0,07767453	0,12675436	0,08845903	0,06016181	0,1061511	0,129498199	6	14	8	7	13	11	4	11	11	522,18
MS_run02	gi|23502107|ref|NP_698234.1|	rplB gene product [Brucella sui	1,95	1,41	1,68	1,22	0,86	0,73				0,5982906	0,612233988	0,40219191	0,20934421	0,247857486	0,12577973	0,199295131	0,315883551	24	24	20	21	33	22	4	11	11	716,67
MS_run03	gi|23502107|ref|NP_698234.1|	rplB gene product [Brucella sui	2,02	2,00	1,65	1,54	0,82	0,93				0,60614966	0,520838638	0,20613507	0,1611159	0,183121089	0,09905946	0,141407513	0,557865145	12	20	12	16	27	28	4	10	10	618,55
MS_run01	gi|23502108|ref|NP_698235.1|	rplW gene product [Brucella su	0,72	0,82	0,76	0,88	1,05	1,17	0,76	6,47%	1,38E-07	1,32922535	1,213483146	0,13702798	0,09565563	0,074632433	0,25651519	0,171044359	0,124255387	18	9	8	19	14	15	6	7	7	364,93
MS_run02	gi|23502108|ref|NP_698235.1|	rplW gene product [Brucella su	0,70	0,68	0,83	0,84	1,18	1,01				1,20481928	1,469666903	0,22810816	0,13608821	0,077459667	0,22672534	0,267674907	0,142365533	27	12	9	19	18	21	5	6	6	324,76
MS_run03	gi|23502108|ref|NP_698235.1|	rplW gene product [Brucella su	0,71	0,68	0,78	0,75	1,10	0,96				1,28205128	1,475574713	0,13296616	0,0993311	0,139522997	0,29375023	0,318650714	0,159195598	17	9	19	23	22	23	7	7	7	402,92
MS_run01	gi|23502109|ref|NP_698236.1|	rplD gene product [Brucella sui	0,77	1,45	0,85	1,65	1,11	1,94	1,22	49,67%	3,58E-02	1,17647059	0,64603258	0,11846237	0,05131601	0,492375196	0,21305444	0,06346497	0,473595162	14	5	30	18	10	33	3	8	8	547,16
MS_run02	gi|23502109|ref|NP_698236.1|	rplD gene product [Brucella sui	0,65	1,21	0,90	1,72	1,37	1,92				1,19269723	0,912088802	0,31819805	0,21213203	0,989949494	0,42403792	0,401621985	0,534600791	36	15	58	36	44	44	2	8	8	574,89
MS_run03	gi|23502109|ref|NP_698236.1|	rplD gene product [Brucella sui	0,68	1,33	1,05	2,39	1,55	2,28				1,13378685	0,953065134	0,5939697	0,77781746	2,361736649	0,64136669	0,616347865	0,857956228	57	50	99	57	65	65	2	8	8	626,78
MS_run01	gi|23502110|ref|NP_698237.1|	rplC gene product [Brucella sui	1,05	0,99	0,97	0,94	0,92	0,97	1,01	15,08%	2,48E-01	1,03763441	1,015951595	0,11902381	0,11902381	0,085391256	0,11407307	0,168286329	0,179099083	12	13	9	11	17	18	4	8	8	383,94
MS_run02	gi|23502110|ref|NP_698237.1|	rplC gene product [Brucella sui	1,05	0,80	1,03	0,87	0,98	0,84				0,97087379	1,126436782	0,06027714	0,18903263	0,168027775	0,05577082	0,381395976	0,120755432	6	19	19	6	34	15	3	8	8	362,77
MS_run03	gi|23502110|ref|NP_698237.1|	rplC gene product [Brucella sui	1,01	1,44	0,93	1,07	0,92	1,15				0,92523131	0,608465608	0,25383722	0,16090888	0,250266525	0,33004535	0,115262309	0,40091395	27	17	23	36	19	28	4	9	9	351,41
MS_run01	gi|23502111|ref|NP_698238.1|	rpsJ gene product [Brucella sui	1,14	0,87	0,73	0,72	0,64	0,98	0,91	20,80%	2,33E-01	1,36986301	1,068493151	0,10148892	0,42426407	0,06363961	0,21450356	0,381901141	0,090076068	14	66	9	16	36	10	3	4	4	136,57
MS_run02	gi|23502111|ref|NP_698238.1|	rpsJ gene product [Brucella sui	1,19	1,03	0,84	0,85	0,71	1,01				1,19047619	0,940789474	0,08962886	0,27577164	0,06363961	0,10851738	0,198589097	0,052359966	11	39	8	9	21	5	3	5	5	171,15
MS_run03	gi|23502111|ref|NP_698238.1|	rpsJ gene product [Brucella sui	0,75	0,66	0,80	1,35	1,06	1,69				1,2347561	1,435714286	0,06429101	0,13576941	0,919238816	0,54077758	0,433567505	0,044909025	8	13	68	44	30	7	4	4	4	225,89
MS_run01	gi|23502112|ref|NP_698239.1|	tuf gene product [Brucella suis	1,19	1,08	1,20	1,13	1,01	0,94	1,18	15,86%	3,48E-02	0,83339121	0,92173913	0,12794391	0,1015874	0,238307524	0,08097015	0,187536237	0,261677423	11	10	21	10	20	24	8	14	14	1089,21
MS_run02	gi|23502112|ref|NP_698239.1|	tuf gene product [Brucella suis	1,02	0,98	1,08	1,02	1,06	0,94				0,92592593	1,016782407	0,15832456	0,21507474	0,09486833	0,1296799	0,131843019	0,117244704	15	20	9	14	13	12	8	11	11	855,71
MS_run03	gi|23502112|ref|NP_698239.1|	tuf gene product [Brucella suis	1,21	1,34	1,33	1,56	1,10	1,17				0,7525604	0,745212593	0,15858752	0,17183879	0,33752037	0,10309037	0,174603011	0,264904356	12	16	22	14	23	20	8	15	15	917,66
MS_run01	gi|23502113|ref|NP_698240.1|	fusA gene product [Brucella su	1,09	0,90	1,16	0,97	1,06	0,84	1,12	13,88%	1,10E-01	0,86711712	1,107142857	0,3692515	0,54874749	0,485994317	0,29268602	0,247773303	0,209284074	32	52	50	34	22	23	10	24	24	1476,86
MS_run02	gi|23502113|ref|NP_698240.1|	fusA gene product [Brucella su	0,93	0,95	1,24	1,18	1,33	0,95				0,8083442	1,059357641	0,24956748	0,46106502	0,120968315	0,13577161	0,230418754	0,2332363	20	35	10	17	22	25	12	24	24	1398,33
MS_run03	gi|23502113|ref|NP_698240.1|	fusA gene product [Brucella su	1,27	1,21	1,33	1,17	1,04	0,89				0,75472773	0,838033686	0,19768301	0,16152547	0,293497992	0,09565376	0,306780646	0,38593488	15	16	25	13	37	32	11	25	25	1489,54
MS_run01	gi|23502114|ref|NP_698241.1|	rpsG gene product [Brucella su	0,91	1,09	0,85	1,00	0,93	1,18	0,96	11,71%	1,25E-02	1,18546577	0,949675325	0,04949747	0,07071068	0,169705627	0,0694409	0,231875925	0,265001057	6	8	17	6	24	24	2	9	9	485,6
MS_run02	gi|23502114|ref|NP_698241.1|	rpsG gene product [Brucella su	0,83	0,91	1,11	1,22	1,34	1,10				0,9009009	1,098360656	0	0	0	0	0	0	0	0	0	0	0	0	1	8	8	589,47
MS_run03	gi|23502114|ref|NP_698241.1|	rpsG gene product [Brucella su	0,84	0,91	0,90	0,97	1,08	1,07				1,11607143	1,134753314	0,08485281	0,09192388	0,148492424	0,10522422	0,26987166	0,215681693	9	9	15	9	24	24	2	8	8	467,69
MS_run01	gi|23502115|ref|NP_698242.1|	rpsL gene product [Brucella sui	1,11	0,96	0,94	0,82	0,85	0,87	0,87	22,95%	3,31E-01	1,04211898	1,036585366	0,48538644	0,43935559	0,495075752	0,03070372	0,137009754	0,13600271	52	52	60	3	13	14	3	6	6	235,56
MS_run02	gi|23502115|ref|NP_698242.1|	rpsL gene product [Brucella sui	1,35	1,16	0,66	0,63	0,49	0,95				5,96539162	0,879310345	0,79903066	0,55861436	0,756604256	7,27714629	0,170680947	0,224988521	122	115	121	122	19	19	2	5	5	181,04
MS_run03	gi|23502115|ref|NP_698242.1|	rpsL gene product [Brucella sui	0,81	0,67	0,69	0,62	0,85	0,90				1,38253242	1,5	0,3453018	0,48445846	0,302875112	0,09438878	0,329262055	0,14469881	50	57	49	7	22	22	3	6	6	235,78
MS_run03	gi|23502118|ref|NP_698245.1|	unnamed protein product [Bru	0,72	2,19	0,77	2,34	1,07	3,04	1,50	76,15%	2,86E-01	1,2987013	0,457264957	0	0	0	0,00E+00	0	0	0	0	0	0	0	0	1	2	2	96,04
MS_run01	gi|23502119|ref|NP_698246.1|	rpoC gene product [Brucella su	1,64	1,51	1,44	1,24	0,88	0,86	1,58	66,46%	4,53E-01	0,6993007	0,663865546	1,84635497	0,13011533	0,225514227	0,31888224	0,214612548	0,319809337	128	15	18	46	32	21	6	18	18	1060,01
MS_run02	gi|23502119|ref|NP_698246.1|	rpoC gene product [Brucella su	0,46	0,56	1,66	2,01	3,62	1,21				0,60240964	1,800995025	0	0	0	0	0	0	0	0	0	0	0	0	1	11	11	609,36
MS_run03	gi|23502119|ref|NP_698246.1|	rpoC gene product [Brucella su	1,21	1,94	2,84	2,47	2,35	0,87				0,35149493	0,841248606	0,00707107	2,44658946	0,735391052	8,74E-04	0,7400578	1,705766524	0	104	30	0	88	88	2	15	15	765,1
MS_run01	gi|23502120|ref|NP_698247.1|	rpoB gene product [Brucella su	1,14	1,15	1,07	1,06	0,94	0,99	1,96	76,98%	3,64E-03	0,93490607	0,867768595	0,18078583	0,1832268	0,171999446	0,17548361	0,170094479	0,2327702	17	19	16	19	20	20	17	42	42	2280,44
MS_run02	gi|23502120|ref|NP_698247.1|	rpoB gene product [Brucella su	2,35	3,10	1,51	1,43	0,64	0,95				0,66569356	0,365811966	0,30968802	0,46008695	0,28777885	0,15123627	0,240664267	1,648898511	21	72	20	23	66	53	7	28	27	1601,66
MS_run03	gi|23502120|ref|NP_698247.1|	rpoB gene product [Brucella su	2,78	2,73	2,40	2,84	0,86	1,18				0,41762572	0,369138756	0,2991864	0,24452091	0,517105612	0,04704915	0,061703055	0,559236322	12	28	18	11	17	20	9	27	27	1622,04
MS_run01	gi|23502122|ref|NP_698249.1|	rplL gene product [Brucella sui	0,85	0,78	0,89	0,81	1,05	0,91	0,78	7,71%	2,64E-05	1,12359551	1,285839008	0,1041519	0,12778454	0,072456884	0,15820773	0,239323839	0,116892468	12	12	9	14	19	15	10	9	9	753,01
MS_run02	gi|23502122|ref|NP_698249.1|	rplL gene product [Brucella sui	0,79	0,73	0,87	0,84	1,10	0,97				1,14942529	1,363636364	0,08573214	0,18851466	0,135088448	0,12458431	0,176359381	0,10986246	10	17	16	11	13	15	12	10	10	680,7
MS_run03	gi|23502122|ref|NP_698249.1|	rplL gene product [Brucella sui	0,70	0,64	0,74	0,67	1,06	0,91				1,35135135	1,56199005	0,11010817	0,10769501	0,072972598	0,24115638	0,199746166	0,093779463	15	10	11	18	13	15	10	9	9	579,52
MS_run01	gi|23502123|ref|NP_698250.1|	rplJ gene product [Brucella suis	0,83	0,71	0,84	0,71	1,01	0,84	0,82	9,82%	6,61E-03	1,19047619	1,402777778	0,24953958	0,17248188	0,115181017	0,34996914	0,283575069	0,187387743	30	17	16	29	20	26	5	5	5	406,12
MS_run02	gi|23502123|ref|NP_698250.1|	rplJ gene product [Brucella suis	1,01	0,97	0,91	0,85	0,90	0,93				1,11261372	1,070716292	0,10606602	0,19091883	0,06363961	0,13039835	0,30657846	0,278850106	12	21	8	12	29	29	2	4	4	196,36
MS_run03	gi|23502123|ref|NP_698250.1|	rplJ gene product [Brucella suis	0,72	0,79	0,75	0,74	1,03	0,99				1,38116426	1,267454955	0,22818121	0,14888474	0,17691806	0,38179385	0,17282826	0,094067655	31	14	24	28	14	12	4	6	6	446,72
MS_run01	gi|23502124|ref|NP_698251.1|	rplA gene product [Brucella sui	0,91	0,90	1,05	0,97	1,15	0,93	1,06	12,80%	3,98E-01	0,95801247	1,11185567	0,12301761	0,10372239	0,105023807	0,10775753	0,171808844	0,160268848	12	9	11	11	15	18	5	7	7	296,62
MS_run02	gi|23502124|ref|NP_698251.1|	rplA gene product [Brucella sui	1,09	0,90	1,20	1,07	1,10	0,89				0,83333333	1,048725161	0,19	0,05656854	0,061101009	0,11772259	0,082124733	0,49012294	16	5	6	14	8	54	3	5	5	248,42
MS_run03	gi|23502124|ref|NP_698251.1|	rplA gene product [Brucella sui	1,07	0,98	1,31	1,23	1,23	0,94				0,76353675	0,980952381	0,09742518	0,13228757	0,168720676	0,06480916	0,109150422	0,431494827	7	11	14	8	11	44	4	6	6	327,09
MS_run02	gi|23502125|ref|NP_698252.1|	rplK gene product [Brucella sui	0,83	0,68	0,90	0,73	1,08	0,81	0,78	8,73%	1,12E-01	1,11111111	1,479452055	0	0	0	0	0	0	0	0	0	0	0	0	1	1	1	32,88
MS_run01	gi|23502126|ref|NP_698253.1|	nusG gene product [Brucella su	0,69	0,77	0,77	0,89	1,11	1,15	0,73	8,12%	8,88E-06	1,17566644	1,302631579	0,28589042	0,10066446	0,176776695	0,17399757	0,234021735	0,135680838	37	9	20	15	18	18	3	4	4	159,16
MS_run02	gi|23502126|ref|NP_698253.1|	nusG gene product [Brucella su	0,67	0,80	0,73	0,78	1,09	1,07				1,31578947	1,253333333	0,04242641	0,2510644	0,042426407	0,32008624	0,068500298	0,041786042	6	23	5	24	5	5	3	5	5	176,55
MS_run03	gi|23502126|ref|NP_698253.1|	nusG gene product [Brucella su	0,71	0,58	0,80	0,62	1,13	0,77				1,2562422	1,517632482	0,12355835	0,07505553	0,141539158	0,19102751	0,474304142	0,126689359	15	7	23	15	31	22	4	6	6	208,95
MS_run03	gi|23502137|ref|NP_698264.1|	unnamed protein product [Bru	0,89	0,85	0,84	0,80	0,94	0,95	0,85	3,33%	1,32E-02	1,19047619	1,175	0	0	0	0	0	0	0	0	0	0	0	0	1	3	3	184,5
MS_run03	gi|23502141|ref|NP_698268.1|	unnamed protein product [Bru	1,16	1,25	1,13	1,21	0,97	1,07	1,19	4,45%	1,37E-02	0,88495575	0,801652893	0	0	0	0	0	0	0	0	0	0	0	0	1	1	1	29,28
MS_run01	gi|23502144|ref|NP_698271.1|	gloA gene product [Brucella su	0,18	0,19	4,41	4,60	23,93	1,04	1,58	171,10%	1,14E-01	0,22675737	5,202173913	0	0	0	0	0	0	0	0	0	0	0	0	1	3	3	126,1
MS_run03	gi|23502144|ref|NP_698271.1|	gloA gene product [Brucella su	0,92	0,92	0,70	0,70	0,76	1,00				1,42857143	1,085714286	0	0	0	0	0	0	0	0	0	0	0	0	1	3	3	146,64
MS_run01	gi|23502145|ref|NP_698272.1|	unnamed protein product [Bru	1,05	0,98	0,98	0,92	0,93	0,94	1,41	46,27%	3,52E-02	1,12899491	1,03204893	0,417193	0,09899495	0,240416306	0,48308592	0,162093958	0,154083501	43	11	26	43	16	16	2	4	4	180,69
MS_run02	gi|23502145|ref|NP_698272.1|	unnamed protein product [Bru	2,14	1,99	1,69	1,57	0,79	0,93				0,59171598	0,503184713	0	0	0	0	0	0	0	0	0	0	0	0	1	4	4	198,43
MS_run02	gi|23502147|ref|NP_698274.1|	ddl gene product [Brucella suis	1,07	1,49	15,69	21,95	14,70	1,40	10,05	904,61%	4,16E-01	0,06373486	0,669703872	0	0	0	0	0	0	0	0	0	0	0	0	1	1	1	24,55
MS_run01	gi|23502149|ref|NP_698276.1|	glpX gene product [Brucella su	1,31	1,17	0,94	0,83	0,72	0,89	1,15	21,08%	6,33E-03	1,07843137	0,85908628	0,12020815	0,10606602	0,056568542	0,13864839	0,069239468	0,094122402	13	15	7	13	8	8	2	6	6	410,91

MS_run02	gi|23502149|ref|NP_698276.1|	glpX gene product [Brucella su	1,62	1,08	1,24	0,96	0,77	0,77				0,80645161	0,927536232	0,37634204	0,1767767	0,300499584	0,24498151	0,091199416	0,090621149	30	23	31	30	10	8	3	7	7	399,31
MS_run03	gi|23502149|ref|NP_698276.1|	glpX gene product [Brucella su	1,15	1,39	0,97	1,15	0,84	1,19				1,03092784	0,721518987	0,08185353	0,24062419	0,245831921	0,08174704	0,129352194	0,20870964	8	29	21	8	18	15	3	6	6	413,46
MS_run01	gi|23502150|ref|NP_698277.1|	hom gene product [Brucella su	1,40	1,61	1,22	1,40	0,87	1,15	1,20	24,76%	1,45E-02	0,81967213	0,621428571	0	0	0	0	0	0	0	0	0	0	0	0	1	5	5	490,29
MS_run02	gi|23502150|ref|NP_698277.1|	hom gene product [Brucella su	1,02	1,07	0,91	0,95	0,89	1,04				1,0989011	0,936842105	0	0	0	0	0	0	0	0	0	0	0	0	1	5	5	604,08
MS_run03	gi|23502150|ref|NP_698277.1|	hom gene product [Brucella su	1,53	1,42	0,98	0,91	0,64	0,93				1,02040816	0,703296703	0	0	0	0	0	0	0	0	0	0	0	0	1	4	4	338,73
MS_run03	gi|23502160|ref|NP_698287.1|	unnamed protein product [Bru	0,14	0,12	5,98	5,00	42,41	0,84	2,81	270,26%	1,10E-01	0,16722408	8,482	0	0	0	0	0	0	0	0	0	0	0	0	1	1	1	25,33
MS_run01	gi|23502183|ref|NP_698310.1|	unnamed protein product [Bru	0,76	0,50	1,03	0,68	1,35	0,66	0,53	19,08%	2,16E-04	0,97087379	1,985294118	0	0	0	0	0	0	0	0	0	0	0	0	1	5	5	214,26
MS_run02	gi|23502183|ref|NP_698310.1|	unnamed protein product [Bru	0,43	0,52	0,38	0,46	0,88	1,21				2,63157895	1,913043478	0	0	0	0	0	0	0	0	0	0	0	0	1	4	3	178,56
MS_run03	gi|23502183|ref|NP_698310.1|	unnamed protein product [Bru	0,41	0,39	0,40	0,38	0,97	0,95				2,5	2,552631579	0	0	0	0	0	0	0	0	0	0	0	0	1	4	4	242,65
MS_run01	gi|23502202|ref|NP_698329.1|	unnamed protein product [Bru	0,82	1,07	1,19	1,51	1,46	1,27	1,03	22,98%	2,05E-01	0,84325397	0,945894263	0,09899495	0,55861436	0,339411255	0,07014948	0,15732927	0,178309181	8	38	22	8	17	17	2	7	7	411,59
MS_run02	gi|23502202|ref|NP_698329.1|	unnamed protein product [Bru	1,07	1,43	0,79	0,99	0,74	1,25				1,26907631	0,451685892	0,05656854	0,15044379	0,007071068	0,09087316	0,183772472	0,411088697	7	20	1	7	41	29	3	8	8	408,23
MS_run03	gi|23502202|ref|NP_698329.1|	unnamed protein product [Bru	0,81	0,93	0,87	0,87	1,08	1,00				1,15691489	0,957812303	0,12793227	0,24454039	0,045092498	0,17734658	0,502011106	0,25993351	15	23	5	15	52	28	4	8	8	412,1
MS_run03	gi|23502209|ref|NP_698336.1|	fucU gene product [Brucella su	0,56	0,54	0,79	0,76	1,42	0,96	0,66	11,53%	1,84E-02	1,26582278	1,868421053	0	0	0	0	0	0	0	0	0	0	0	0	1	3	3	80,59
MS_run01	gi|23502236|ref|NP_698363.1|	unnamed protein product [Bru	0,96	1,39	0,98	1,42	1,02	1,45	0,97	27,38%	1,21E-01	1,02040816	0,718309859	0	0	0	0	0	0	0	0	0	0	0	0	1	1	1	30,04
MS_run03	gi|23502236|ref|NP_698363.1|	unnamed protein product [Bru	0,67	0,69	0,80	0,82	1,19	1,03				1,25	1,451219512	0	0	0	0	0	0	0	0	0	0	0	0	1	2	2	64,55
MS_run01	gi|23502244|ref|NP_698371.1|	unnamed protein product [Bru	0,93	1,45	0,78	1,22	0,84	1,56	1,07	19,13%	3,05E-01	1,28205128	0,68852459	0	0	0	0	0	0	0	0	0	0	0	0	1	1	1	37,57
MS_run02	gi|23502244|ref|NP_698371.1|	unnamed protein product [Bru	0,95	1,03	1,06	1,14	1,11	1,08				0,94339623	0,973684211	0	0	0	0	0	0	0	0	0	0	0	0	1	1	1	24,8
MS_run01	gi|23502249|ref|NP_698376.1|	unnamed protein product [Bru	1,57	1,22	1,20	1,03	0,77	0,85	1,36	18,00%	2,87E-05	0,83356488	0,818181818	0,11954776	0,22779742	0,194422221	0,09742991	0,073117607	0,121816222	10	30	19	12	9	10	5	8	8	292,95
MS_run02	gi|23502249|ref|NP_698376.1|	unnamed protein product [Bru	1,33	1,25	1,42	1,22	1,06	0,86				0,62111801	0,8	0,63981768	0,34847286	0,533479147	0,13382104	0,13800378	0,6030565	45	33	44	22	17	48	4	9	9	358,6
MS_run03	gi|23502249|ref|NP_698376.1|	unnamed protein product [Bru	1,64	1,35	1,59	1,51	0,97	0,95				0,62696541	0,726744186	0,40128959	0,39513711	0,304055916	0,00277951	0,216872501	0,03259981	25	41	20	0	30	2	3	7	7	269,02
MS_run01	gi|23502251|ref|NP_698378.1|	ilvC gene product [Brucella sui:	1,41	1,72	1,43	1,28	1,01	0,90	1,78	36,16%	2,93E-05	0,70280134	0,587493086	0,19145931	0,33130334	0,149331845	0,1163217	0,091610243	0,257644504	13	33	12	17	16	15	7	9	9	565,38
MS_run02	gi|23502251|ref|NP_698378.1|	ilvC gene product [Brucella sui:	1,85	1,98	1,59	1,39	0,86	0,87				0,62893082	0,50625	0,08820431	0,19304576	0,188865031	0,03376576	0,099036124	0,424737298	6	22	14	5	20	22	5	8	8	409,52
MS_run03	gi|23502251|ref|NP_698378.1|	ilvC gene product [Brucella sui:	2,15	2,52	2,05	2,04	0,95	1,00				0,4926049	0,397058824	0,27402859	0,14363147	0,418568991	0,0623512	0,140283818	0,800123788	13	15	21	13	35	32	9	12	12	606,96
MS_run01	gi|23502259|ref|NP_698386.1|	ilvH gene product [Brucella sui	0,71	1,01	1,15	1,65	1,63	1,43	1,13	34,09%	1,26E-01	0,86956522	0,987878788	0	0	0	0	0	0	0	0	0	0	0	0	1	2	2	81,05
MS_run01	gi|23502265|ref|NP_698392.1|	unnamed protein product [Bru	1,08	1,19	0,98	1,08	0,91	1,10	1,03	34,23%	1,34E-01	1,02211097	0,841346154	0,05656854	0,09899495	0,056568542	0,05899931	0,047593726	0,067343503	6	11	5	6	6	6	2	6	6	328,73
MS_run02	gi|23502265|ref|NP_698392.1|	unnamed protein product [Bru	1,32	1,50	1,19	1,36	0,90	1,14				0,84570368	0,674769675	0,07778175	0,02828427	0,261629509	0,05551081	0,10941357	0,243504657	7	3	19	7	16	16	2	6	6	341,28
MS_run03	gi|23502265|ref|NP_698392.1|	unnamed protein product [Bru	0,34	0,97	0,36	1,01	1,05	2,85				2,43902439	1,035460993	0,07778175	0,34239354	0,284312035	1,02844003	0,075404846	0,080121807	22	33	28	42	7	8	3	6	6	328,29
MS_run02	gi|23502268|ref|NP_698395.1|	hflK gene product [Brucella sui	1,03	1,21	0,70	0,82	0,68	1,17	0,94	19,41%	4,78E-01	1,42857143	0,829268293	0	0	0	0	0	0	0	0	0	0	0	0	1	4	4	186,57
MS_run01	gi|23502271|ref|NP_698398.1|	unnamed protein product [Bru	0,71	0,65	0,79	0,75	1,11	0,95	0,73	9,10%	5,25E-06	1,26582278	1,541666667	0,05291503	0,06806859	0,045825757	0,07843543	0,145452678	0,068368294	7	6	6	6	9	11	3	6	6	332,9
MS_run02	gi|23502271|ref|NP_698398.1|	unnamed protein product [Bru	0,58	0,58	0,82	0,85	1,42	1,04				1,24185976	1,717647059	0,23832751	3,71629744	0,152752523	0,42322657	8,443100962	0,343430926	29	263	18	34	492	59	5	7	7	413,85
MS_run03	gi|23502271|ref|NP_698398.1|	unnamed protein product [Bru	0,69	0,70	0,83	0,83	1,19	1,01				1,22222222	1,43373494	0,10606602	0,11930353	0,097125349	0,15713484	0,320361822	0,156650821	13	10	12	13	22	22	3	6	6	676,36
MS_run03	gi|23502285|ref|NP_698412.1|	unnamed protein product [Bru	0,75	0,94	1,02	1,28	1,36	1,25	1,00	19,02%	6,94E-02	0,98039216	1,0625	0	0	0	0	0	0	0	0	0	0	0	0	1	1	1	40,13
MS_run01	gi|23502287|ref|NP_698414.1|	unnamed protein product [Bru	1,03	0,92	1,12	1,00	1,09	0,89	1,02	7,23%	3,95E-01	0,89285714	1,09	0	0	0	0	0	0	0	0	0	0	0	0	1	3	3	185,06
MS_run03	gi|23502293|ref|NP_698420.1|	unnamed protein product [Bru	0,83	0,89	0,86	0,93	1,04	1,08	0,88	3,84%	3,40E-03	1,1627907	1,11827957	0	0	0	0	0	0	0	0	0	0	0	0	1	3	3	114,8
MS_run01	gi|23502301|ref|NP_698428.1|	murC gene product [Brucella si	1,01	1,02	1,00	1,05	0,99	1,05	1,08	6,56%	1,49E-02	1	0,947138047	0,14621901	0,21118712	0,162696036	0,14232301	0,349840451	0,191366331	15	21	16	14	37	19	7	15	15	1141,62
MS_run02	gi|23502301|ref|NP_698428.1|	murC gene product [Brucella si	1,10	1,26	1,11	1,09	1,01	0,98				0,9009009	0,794411765	0,24405308	0,19230432	0,233273802	0,1700568	0,113974965	0,181752608	22	19	21	19	14	14	7	15	15	1281,28
MS_run03	gi|23502301|ref|NP_698428.1|	murC gene product [Brucella si	1,09	1,07	1,06	1,11	0,97	1,05				0,94788859	0,923295455	0,14380542	0,16330952	0,140202235	0,14007718	0,327832672	0,078716691	14	17	13	15	36	7	7	15	15	1138,59
MS_run01	gi|23502307|ref|NP_698434.1|	murE gene product [Brucella si	1,15	1,43	1,07	1,33	0,93	1,24	1,10	34,98%	1,27E-01	0,93457944	0,69924812	0	0	0	0	0	0	0	0	0	0	0	0	1	6	6	373,8
MS_run03	gi|23502307|ref|NP_698434.1|	murE gene product [Brucella si	0,35	0,78	1,48	1,18	4,19	0,80				0,72609528	3,458009779	0,55154329	4,76589971	0,056568542	0,27058985	3,873122993	0,868941066	37	114	5	37	112	112	2	12	12	758,93
MS_run01	gi|23502337|ref|NP_698464.1|	unnamed protein product [Bru	0,92	0,90	0,97	0,95	1,05	0,98	0,82	8,82%	6,34E-05	1,03629725	1,114969802	0,14179798	0,25437505	0,217293964	0,18055973	0,087699273	0,066517277	15	24	23	17	8	7	6	8	8	462,38
MS_run02	gi|23502337|ref|NP_698464.1|	unnamed protein product [Bru	0,70	0,74	0,85	0,81	1,22	0,95				1,17663344	1,356693364	0,05	0,28071338	0,071879529	0,06767899	0,319363678	0,210805381	6	23	9	6	24	29	5	8	8	358,07
MS_run03	gi|23502337|ref|NP_698464.1|	unnamed protein product [Bru	0,73	0,74	0,77	0,83	1,05	1,08				1,30724539	1,361834812	0,1109955	0,13831124	0,071902712	0,26411684	0,286845866	0,13210069	15	13	9	20	21	18	6	8	8	462,07
MS_run01	gi|23502344|ref|NP_698471.1|	omp28 gene product [Brucella	1,94	1,72	1,71	1,51	0,88	0,88	1,58	30,18%	4,76E-05	0,59134765	0,593850267	0,25455844	0,02828427	0,268700577	0,08803072	0,124405418	0,360679273	15	3	18	15	21	21	2	6	6	412,55
MS_run02	gi|23502344|ref|NP_698471.1|	omp28 gene product [Brucella	1,95	1,85	1,78	1,70	0,92	0,95				0,56195738	0,539699721	0,04242641	0,07778175	0,134350288	0,01339429	0,003110875	0,010680347	2	9	8	2	1	1	2	5	5	384,72
MS_run03	gi|23502344|ref|NP_698471.1|	omp28 gene product [Brucella	1,33	1,24	1,14	1,05	0,86	0,92				0,87780088	0,813890421	0,04242641	0,1767767	0,091923882	0,03266837	0,097570075	0,148359977	4	21	9	4	12	12	2	6	6	358,27
MS_run01	gi|23502348|ref|NP_698475.1|	rpoD gene product [Brucella su	1,16	0,92	1,28	1,01	1,10	0,79	1,09	13,91%	1,94E-01	0,78125	1,089108911	0	0	0	0	0	0	0	0	0	0	0	0	1	4	4	272,31
MS_run01	gi|23502352|ref|NP_698479.1|	carA gene product [Brucella su	0,89	1,87	0,93	1,49	1,04	1,61	1,01	33,55%	3,28E-01	0,73804521	0,533809338	0,23334524	0,15556349	0,947523087	0,2536901	0,001982629	0,57253352	25	15	64	34	0	31	3	7	7	306,75
MS_run02	gi|23502352|ref|NP_698479.1|	carA gene product [Brucella su	0,99	1,22	0,68	0,84	0,69	1,24				1,47058824	0,821428571	0	0	0	0	0	0	0	0	0	0	0	0	1	5	5	234,68
MS_run03	gi|23502352|ref|NP_698479.1|	carA gene product [Brucella su	0,83	0,94	0,70	0,79	0,84	1,13				1,42857143	1,063291139	0	0	0	0	0	0	0	0	0	0	0	0	1	3	3	142,89
MS_run02	gi|23502361|ref|NP_698488.1|	unnamed protein product [Bru	2,84	2,35	1,76	1,46	0,62	0,83	1,60	65,70%	1,77E-02	0,56818182	0,424657534	0	0	0	0	0	0	0	0	0	0	0	0	1	1	1	34,34
MS_run03	gi|23502361|ref|NP_698488.1|	unnamed protein product [Bru	1,49	0,97	1,16	0,76	0,78	0,66				0,86206897	1,026315789	0	0	0	0	0	0	0	0	0	0	0	0	1	1	1	40,49
MS_run01	gi|23502364|ref|NP_698491.1|	aspC gene product [Brucella su	1,15	1,16	1,48	1,12	1,30	0,75	1,29	16,66%	1,23E-03	0,68288421	0,859813084	5,64563696	1,57575379	3,448011021	0,36149695	0,467886397	4,217743682	380	122	308	53	54	363	6	8	8	474,63
MS_run02	gi|23502364|ref|NP_698491.1|	aspC gene product [Brucella su	1,31	1,22	1,26	1,07	0,96	0,85				0,79783853	0,817307692	0,2310844	0,34789845	0,284903492	0,13578124	0,105164232	0,17135279	18	36	27	17	13	14	6	8	8	383,2
MS_run03	gi|23502364|ref|NP_698491.1|	aspC gene product [Brucella su	1,52	1,15	1,56	1,46	1,02	0,94				0,65231983	0,706349206	0,41912607	0,15567059	0,376153781	0,13933483	0,282308648	0,506749005	27	15	26	21	40	44	4	6	6	285,69
MS_run02	gi|23502368|ref|NP_698495.1|	trxB gene product [Brucella sui	0,84	1,00	0,16	0,19	0,19	1,19	0,55	37,73%	3,84E-01	6,25	1	0	0	0	0	0	0	0	0	0	0	0	0	1	3	3	160,75
MS_run01	gi|23502373|ref|NP_698500.1|	greA gene product [Brucella su	0,58	0,57	0,61	0,62	1,06	1,02	0,71	10,16%	4,19E-05	1,66122004	1,770999116	0,12055428	0,17339742	0,142711831	0,3384915	0,214957849	0,071907646	20	16	23	20	12	13	4	6	6	333,96
MS_run02	gi|23502373|ref|NP_698500.1|	greA gene product [Brucella su	0,90	0,84	0,78	0,68	0,87	0,87				1,20282339	1,189473684	0,19553346	0,31659648	0,195533458	0,11204515	0,211103467	0,192142463	25	36	29	9	18	23	3	5	5	299,33
MS_run03	gi|23502373|ref|NP_698500.1|	greA gene product [Brucella su	0,72	0,81	0,71	0,73	0,98	1,04				1,33333333	1,234208777	0,19939492	0,25073226	0,139522997	0,22707609	0,178606513	0,101135841	28	26	19	17	14	12	4	6	6	304,14
MS_run01	gi|23502379|ref|NP_698506.1|	unnamed protein product [Bru	1,16	0,86	0,96	0,67	0,83	0,69	1,85	106,70%	4,30E-02	1,13851992	1,277054795	0,3959798	0,23334524	0,091923882	0,46961551	0,527424168	0,353553391	41	28	14	41	41	41	2	3	3	181,68
MS_run03	gi|23502379|ref|NP_698506.1|	unnamed protein product [Bru	3,83	2,91	2,49	1,89	0,65	0,76				0,40160643	0,343915344	0	0	0	0	0	0	0	0	0	0	0	0	1	2	2	136
MS_run01	gi|23502382|ref|NP_698509.1|	unnamed protein product [Bru	0,87	0,90	1,00	1,04	1,15	1,04	0,80	31,78%	6,66E-03	1	1,105769231	0	0	0	0	0	0	0	0	0	0	0	0	1	1	1	38,28
MS_run02	gi|23502382|ref|NP_698509.1|	unnamed protein product [Bru	0,37	0,34	1,06	0,98	2,86	0,92				0,94339623	2,918367347	0	0	0	0	0	0	0	0	0	0	0	0	1	1	1	42,09
MS_run03	gi|23502382|ref|NP_698509.1|	unnamed protein product [Bru	0,37	0,36	1,15	1,13	3,10	0,98				0,86956522	2,743362832	0	0	0	0	0	0	0	0	0	0	0	0	1	1	1	41,32
MS_run01	gi|23502389|ref|NP_698516.1|	unnamed protein product [Bru	1,60	1,24	1,28	0,99	0,80	0,77	1,18	19,88%	3,04E-03	0,78125	0,808080808	0	0	0	0	0	0	0	0	0	0	0	0	1	7	7	593,04
MS_run02	gi|23502389|ref|NP_698516.1|	unnamed protein product [Bru	1,25	1,20	0,97	0,88	0,77	0,91				1,03651987	0,864847046	0,0212132	0,29698485	0,120208153	0,0227854	0,220597923	0,304849607	2	39	14	2	26	26	2	8	8	702,36
MS_run03	gi|23502389|ref|NP_698516.1|	unnamed protein product [Bru	1,21	1,44	0,99	1,18	0,82	1,19				1,01010101	0,694915254	0	0	0	0	0	0	0	0	0	0	0	0	1	7	7	705,07
MS_run01	gi|23502394|ref|NP_698521.1|	unnamed protein product [Bru	1,34	0,88	0,99	0,65	0,74	0,66	1,40	48,91%	8,59E-03	1,01010101	1,138461538	0	0	0	0	0	0	0	0	0	0	0	0	1	2	2	109,49
MS_run02	gi|23502394|ref|NP_698521.1|	unnamed protein product [Bru	2,52	1,91	1,64	1,24	0,65	0,76				0,6097561	0,524193548	0	0	0	0	0	0	0	0	0	0	0	0	1	3	3	168,7
MS_run03	gi|23502394|ref|NP_698521.1|	unnamed protein product [Bru	1,38	1,79	1,08	1,37	0,78	1,27				0,933646	0,55976912	0,09192388	0,36769553	0,537401154	0,07983662	0,048813836	0,156379384	9	47	39	9	9	9	2	4	4	283,42
MS_run01	gi|23502403|ref|NP_698530.1|	unnamed protein product [Bru	0,81	0,86	0,98	0,97	1,21	0,99	0,84	9,10%	9,77E-05	1,02211097	1,161616162	0,16083117	0,29835661	0,08043631	0,19338637	0,146957141	0,106274749	16	25	8	19	13	12	7	7	7	380,09
MS_run02	gi|23502403|ref|NP_698530.1|	unnamed protein product [Bru	0,82	0,83	0,80	0,80	0,97	1,00				1,25019534	1,204778157	0,09183318	0,1596611	0,175404295	0,16118128	0,275703397	0,195703263	11	16	22	13	23	24	5	4	4	290,5
MS_run03	gi|23502403|ref|NP_698530.1|	unnamed protein product [Bru	0,67	0,69	0,85	0,93	1,27	1,09				1,17647059	1,442732367	0,32012498	0,49261547	0,387969071	0,29404096	0,111246281	0,054305337	38	39	42	25	8	8	6	5	5	416,21

MS_run01	gi|23502420|ref|NP_698547.1|	moxR gene product [Brucella s	1,15	0,97	0,98	0,82	0,85	0,83	1,16	18,43%	1,02E-03	1,02564103	1,05754631	0,09899495	0,07071068	0,106066017	0,10360539	0,224393133	0,205257385	10	8	13	10	21	21	2	6	6	266,35
MS_run02	gi|23502420|ref|NP_698547.1|	moxR gene product [Brucella s	1,35	1,36	1,19	1,15	0,88	0,97				0,8533775	0,757642252	0,1767767	0,23334524	0,042426407	0,1273057	0,174957565	0,313141817	15	27	4	15	23	23	2	6	6	261,93
MS_run03	gi|23502420|ref|NP_698547.1|	moxR gene product [Brucella s	1,49	1,25	1,23	1,03	0,82	0,84				0,85034014	0,862051282	0,34648232	0	0,38890873	0,24051251	0,327082214	0,474278939	28	0	38	28	38	38	2	6	6	218,86
MS_run01	gi|23502443|ref|NP_698570.1|	unnamed protein product [Bru	1,00	1,12	1,23	1,38	1,23	1,12	1,35	27,85%	4,79E-02	0,81797543	0,906989247	0,07778175	0,0212132	0,247487373	0,0519376	0,17867752	0,221501058	6	2	18	6	20	20	2	3	3	174,81
MS_run02	gi|23502443|ref|NP_698570.1|	unnamed protein product [Bru	1,75	1,73	1,66	1,64	0,95	0,99				0,60240964	0,579268293	0	0	0	0	0	0	0	0	0	0	0	0	1	2	2	220,15
MS_run03	gi|23502443|ref|NP_698570.1|	unnamed protein product [Bru	0,95	1,05	1,28	1,42	1,35	1,11				0,83191961	1,100747133	0,43133514	0,00707107	0,74246212	0,28144013	0,572573839	0,546493085	34	1	52	34	52	52	2	3	3	202,16
MS_run01	gi|23502457|ref|NP_698584.1|	unnamed protein product [Bru	0,83	0,72	1,06	0,92	1,27	0,87	0,92	13,22%	4,25E-03	0,94339623	1,380434783	0	0	0	0	0	0	0	0	0	0	0	0	1	2	2	139,58
MS_run02	gi|23502457|ref|NP_698584.1|	unnamed protein product [Bru	0,91	1,06	0,92	1,07	1,01	1,16				1,08695652	0,943925234	0	0	0	0	0	0	0	0	0	0	0	0	1	2	2	120,5
MS_run03	gi|23502457|ref|NP_698584.1|	unnamed protein product [Bru	0,68	0,83	0,91	1,11	1,34	1,22				1,0989011	1,207207207	0	0	0	0	0	0	0	0	0	0	0	0	1	2	2	112,73
MS_run01	gi|23502461|ref|NP_698588.1|	unnamed protein product [Bru	0,68	0,45	0,64	0,42	0,94	0,66	1,05	51,57%	2,93E-01	1,5625	2,238095238	0	0	0	0	0	0	0	0	0	0	0	0	1	2	2	121,16
MS_run03	gi|23502461|ref|NP_698588.1|	unnamed protein product [Bru	1,59	1,66	1,46	1,53	0,92	1,05				0,68493151	0,60130719	0	0	0	0	0	0	0	0	0	0	0	0	1	3	3	165,48
MS_run01	gi|23502462|ref|NP_698589.1|	ctrA gene product [Brucella sui	0,79	0,90	1,37	1,56	1,73	1,14	1,02	28,04%	5,52E-02	0,72992701	1,108974359	0	0	0	0	0	0	0	0	0	0	0	0	1	4	4	133,18
MS_run03	gi|23502462|ref|NP_698589.1|	ctrA gene product [Brucella sui	0,71	0,96	0,81	1,09	1,14	1,35				1,2345679	1,04587156	0	0	0	0	0	0	0	0	0	0	0	0	1	4	4	140,33
MS_run01	gi|23502464|ref|NP_698591.1|	unnamed protein product [Bru	1,25	1,18	1,26	1,19	1,01	0,94	1,30	9,75%	4,55E-05	0,79365079	0,848739496	0	0	0	0	0	0	0	0	0	0	0	0	1	3	3	127,99
MS_run03	gi|23502464|ref|NP_698591.1|	unnamed protein product [Bru	1,47	1,37	1,40	1,30	0,95	0,93				0,71428571	0,730769231	0	0	0	0	0	0	0	0	0	0	0	0	1	3	3	142,5
MS_run03	gi|23502465|ref|NP_698592.1|	unnamed protein product [Bru	0,46	0,50	1,40	1,54	3,05	1,10	0,98	49,68%	1,05E-01	0,71428571	1,980519481	0	0	0	0	0	0	0	0	0	0	0	0	1	1	1	33,81
MS_run01	gi|23502475|ref|NP_698602.1|	unnamed protein product [Bru	1,33	1,11	1,16	1,20	0,87	1,03	1,17	9,83%	1,43E-02	0,82473527	0,820754717	0,24583192	0,26851443	0,285832119	0,05279782	0,299240245	0,160269477	21	31	24	6	36	15	3	3	3	142,99
MS_run02	gi|23502475|ref|NP_698602.1|	unnamed protein product [Bru	1,09	1,01	1,32	1,22	1,21	0,92				0,75757576	0,879549438	0,02081666	0,00707107	0,229419557	0,01175044	0,158750904	0,51215439	2	1	19	2	18	51	3	3	3	132,38
MS_run03	gi|23502475|ref|NP_698602.1|	unnamed protein product [Bru	1,06	1,09	1,19	1,26	1,12	1,06				0,84033613	0,913978495	0,27221315	0,07071068	0,028284271	0,15039514	0,0295126	0,03741876	23	6	2	18	3	3	3	2	2	173,66
MS_run01	gi|23502477|ref|NP_698604.1|	aceA gene product [Brucella su	1,95	2,04	2,52	2,63	1,29	1,04	2,31	96,82%	2,21E-01	0,3968254	0,490494297	0	0	0	0	0	0	0	0	0	0	0	0	1	4	4	192,77
MS_run03	gi|23502477|ref|NP_698604.1|	aceA gene product [Brucella su	0,94	3,39	1,09	3,93	1,16	3,61				0,91743119	0,295165394	0	0	0	0	0	0	0	0	0	0	0	0	1	6	6	273,36
MS_run01	gi|23502485|ref|NP_698612.1|	omp31-1 gene product [Brucel	1,02	1,03	0,85	0,88	0,83	1,04	1,71	91,04%	1,97E-02	1,18347339	0,973333333	0,0975412	0,12944249	0,109674844	0,12966164	0,152791948	0,170833845	12	16	13	11	16	17	9	9	8	507,12
MS_run02	gi|23502485|ref|NP_698612.1|	omp31-1 gene product [Brucel	1,79	1,39	1,42	1,06	0,79	0,74				0,70217798	0,717171717	0,20736011	0,22996894	0,105807552	0,11731932	0,237385057	0,42804205	15	29	10	17	33	31	9	9	8	539,65
MS_run03	gi|23502485|ref|NP_698612.1|	omp31-1 gene product [Brucel	3,83	3,22	2,03	1,97	0,53	0,97				0,47566322	0,277721723	0,39890326	0,15266521	0,396152328	0,07498544	0,107041739	1,278951879	20	29	20	16	39	40	7	7	6	429,33
MS_run01	gi|23502495|ref|NP_698622.1|	unnamed protein product [Bru	1,27	1,06	1,38	1,15	1,09	0,83	1,86	72,09%	6,41E-03	0,72463768	0,947826087	0	0	0	0	0	0	0	0	0	0	0	0	1	1	1	26,46
MS_run02	gi|23502495|ref|NP_698622.1|	unnamed protein product [Bru	2,89	3,51	2,11	2,45	0,73	1,16				0,51413255	0,305607067	0,834386	0,18384776	0,304055916	0,20331043	0,113198119	1,301295735	40	25	12	40	37	37	2	2	2	81,59
MS_run03	gi|23502495|ref|NP_698622.1|	unnamed protein product [Bru	1,61	1,81	1,44	1,62	0,89	1,13				0,70509041	0,554755945	0,2192031	0,12727922	0,367695526	0,10770593	0,04734695	0,154409032	15	14	23	15	9	9	2	2	2	65,08
MS_run01	gi|23502520|ref|NP_698647.1|	unnamed protein product [Bru	0,81	0,81	0,73	0,74	0,91	1,01	0,83	9,20%	1,04E-02	1,37218045	1,194444444	0,07187953	0,07234178	0,142945211	0,14917663	0,144729551	0,208010886	10	8	19	11	12	26	4	7	7	557,79
MS_run02	gi|23502520|ref|NP_698647.1|	unnamed protein product [Bru	0,92	0,88	1,01	0,91	1,10	0,90				0,99019608	1,132653061	0,08655441	0,09460444	0,045	0,07459407	0,143536417	0,114610214	9	9	5	8	13	13	5	8	8	678,74
MS_run03	gi|23502520|ref|NP_698647.1|	unnamed protein product [Bru	0,91	0,70	0,84	0,74	0,92	0,88				1,19047619	1,4375	0,09539392	0,11150486	0,064291005	0,1143371	0,172584148	0,102165892	11	12	9	10	12	15	3	6	6	420,48
MS_run01	gi|23502525|ref|NP_698652.1|	exbB gene product [Brucella su	1,88	1,42	1,35	1,02	0,72	0,76	1,42	30,49%	3,12E-02	0,74074074	0,705882353	0	0	0	0	0	0	0	0	0	0	0	0	1	1	1	36,34
MS_run03	gi|23502533|ref|NP_698660.1|	dut gene product [Brucella suis	0,83	0,88	0,60	0,63	0,72	1,05	0,73	12,10%	1,80E-01	1,66666667	1,142857143	0	0	0	0	0	0	0	0	0	0	0	0	1	3	3	209,79
MS_run01	gi|23502539|ref|NP_698666.1|	unnamed protein product [Bru	0,53	0,53	0,46	0,46	0,87	1,00	0,49	3,44%	4,47E-04	2,17391304	1,891304348	0	0	0	0	0	0	0	0	0	0	0	0	1	6	6	200,6
MS_run01	gi|23502541|ref|NP_698668.1|	purA gene product [Brucella su	1,12	1,12	1,19	1,23	1,06	1,03	1,19	20,97%	6,28E-03	0,88246199	0,903508772	0,36769553	0,45254834	0,685893578	0,27267002	0,136459203	0,169090752	31	43	56	31	15	15	2	10	10	711,68
MS_run02	gi|23502541|ref|NP_698668.1|	purA gene product [Brucella su	1,20	1,16	1,19	1,15	0,99	0,97				0,84033613	0,860869565	0	0	0	0	0	0	0	0	0	0	0	0	1	9	9	577,94
MS_run03	gi|23502541|ref|NP_698668.1|	purA gene product [Brucella su	1,77	1,11	1,24	0,78	0,70	0,63				0,80645161	0,897435897	0	0	0	0	0	0	0	0	0	0	0	0	1	10	10	542,69
MS_run01	gi|23502543|ref|NP_698670.1|	serA-1 gene product [Brucella :	1,36	2,13	1,89	1,95	1,39	1,03	1,91	25,63%	7,63E-07	0,52910053	0,471441236	0,28384855	0,21344398	0,594289492	0,08295333	0,135962671	0,478602476	15	15	30	16	29	22	5	12	12	704,67
MS_run02	gi|23502543|ref|NP_698670.1|	serA-1 gene product [Brucella :	2,29	1,79	1,99	1,62	0,87	0,81				0,50251256	0,56	0,52519838	0,04242641	0,37434387	0,13979066	0,032326441	0,094509757	26	5	23	28	6	5	3	12	12	838,44
MS_run03	gi|23502543|ref|NP_698670.1|	serA-1 gene product [Brucella :	2,23	1,82	2,16	1,76	0,97	0,81				0,46296296	0,55	0,37865552	0,26705805	0,290206823	0,0853647	0,106665157	0,449811746	18	28	16	18	19	25	5	13	13	981,16
MS_run01	gi|23502545|ref|NP_698672.1|	serC gene product [Brucella sui	1,56	1,29	1,71	1,72	1,10	1,00	1,78	21,14%	1,80E-07	0,59463783	0,777027027	0,33767588	0,26361272	0,226476636	0,11058928	0,137780637	0,310960181	20	24	13	19	18	24	5	11	11	801,6
MS_run02	gi|23502545|ref|NP_698672.1|	serC gene product [Brucella sui	1,80	1,76	1,82	1,80	1,01	0,99				0,54945055	0,544973545	0,24583192	0,36963946	0,127279221	0,08865377	0,212971053	0,10032583	14	37	7	16	39	6	3	10	10	715,41
MS_run03	gi|23502545|ref|NP_698672.1|	serC gene product [Brucella sui	2,03	1,77	2,14	1,99	1,06	0,93				0,46929825	0,500861192	0,49812984	0,32887434	0,283945417	0,09729603	0,148095266	0,311527313	23	31	14	21	30	18	4	10	10	821,49
MS_run01	gi|23502548|ref|NP_698675.1|	glmM gene product [Brucella s	1,11	1,36	1,51	1,63	1,35	1,08	1,68	39,70%	1,86E-03	0,68105711	0,911383937	0,33234019	0,48083261	0,480832611	0,15039379	0,563837871	0,839464047	22	36	29	22	62	62	2	5	5	211,03
MS_run02	gi|23502548|ref|NP_698675.1|	glmM gene product [Brucella s	1,32	1,66	1,36	1,38	1,03	1,02				0,73801743	0,84947188	0,00707107	0,52325902	0,438406204	0,00385134	0,649038232	1,270190298	1	51	32	1	76	76	2	4	4	186,31
MS_run03	gi|23502548|ref|NP_698675.1|	glmM gene product [Brucella s	2,17	2,23	2,25	2,17	1,04	0,97				0,47442942	0,472929293	0,78488853	0,38890873	0,113137085	0,16586824	0,154563543	0,730047268	35	38	5	35	33	33	2	4	4	162,48
MS_run01	gi|23502553|ref|NP_698680.1|	unnamed protein product [Bru	1,82	2,12	1,22	1,42	0,67	1,16	1,71	54,85%	1,35E-03	0,81967213	0,471830986	0	0	0	0	0	0	0	0	0	0	0	0	1	6	6	341,52
MS_run02	gi|23502553|ref|NP_698680.1|	unnamed protein product [Bru	2,75	2,82	1,40	1,44	0,51	1,03				0,71428571	0,354166667	0	0	0	0	0	0	0	0	0	0	0	0	1	7	7	439,58
MS_run03	gi|23502553|ref|NP_698680.1|	unnamed protein product [Bru	1,68	1,50	1,24	1,11	0,74	0,90				0,80645161	0,666666667	0	0	0	0	0	0	0	0	0	0	0	0	1	6	6	373,62
MS_run01	gi|23502555|ref|NP_698682.1|	tolB gene product [Brucella sui	1,59	1,74	1,44	1,66	0,91	1,15	1,46	17,96%	6,25E-06	0,70997427	0,574824301	0,25592968	0,14399074	0,268452355	0,13011077	0,096186048	0,2948178	18	16	16	18	17	17	4	11	11	638,62
MS_run02	gi|23502555|ref|NP_698682.1|	tolB gene product [Brucella sui	1,73	1,51	1,46	1,23	0,85	0,84				0,68260181	0,661533627	0,24554361	0,10012492	0,278373251	0,09371066	0,061841994	0,162664067	17	12	23	14	9	11	4	10	10	591,49
MS_run03	gi|23502555|ref|NP_698682.1|	tolB gene product [Brucella sui	1,43	1,31	1,23	1,24	0,86	1,01				0,78244391	0,765765766	0,24758837	0,13576941	0,193132079	0,04322433	0,1524836	0,306682235	20	16	16	6	20	23	3	7	7	435,57
MS_run01	gi|23502568|ref|NP_698695.1|	efp gene product [Brucella suis	0,72	0,64	0,84	0,74	1,16	0,88	0,66	13,10%	6,93E-05	1,19318182	1,587583149	0,05656854	0,01414214	0,113137085	0,08035304	0,261833331	0,10531713	7	1	15	7	16	16	2	5	5	196
MS_run02	gi|23502568|ref|NP_698695.1|	efp gene product [Brucella suis	0,66	0,60	0,89	0,80	1,34	0,90				1,1308459	1,698363024	0,03535534	0,10606602	0,183847763	0,04517677	0,257717782	0,090388354	4	8	23	4	15	15	2	7	7	223,07
MS_run03	gi|23502568|ref|NP_698695.1|	efp gene product [Brucella suis	0,52	0,49	0,54	0,50	1,05	0,91				1,97463768	2,083333333	0,20506097	0,34648232	0,06363961	0,74297452	0,432120811	0,101749377	38	33	13	38	21	21	2	7	7	224,8
MS_run01	gi|23502574|ref|NP_698701.1|	rpmE gene product [Brucella su	0,75	0,74	0,83	0,82	1,11	0,99	0,74	13,58%	1,12E-03	1,20481928	1,353658537	0	0	0	0	0	0	0	0	0	0	0	0	1	2	2	106,98
MS_run02	gi|23502574|ref|NP_698701.1|	rpmE gene product [Brucella su	0,78	0,64	1,02	0,84	1,31	0,82				0,98039216	1,55952381	0	0	0	0	0	0	0	0	0	0	0	0	1	2	2	96,47
MS_run03	gi|23502574|ref|NP_698701.1|	rpmE gene product [Brucella su	0,61	0,48	0,75	0,59	1,23	0,79				1,33333333	2,084745763	0	0	0	0	0	0	0	0	0	0	0	0	1	2	2	91,4
MS_run01	gi|23502575|ref|NP_698702.1|	unnamed protein product [Bru	0,76	0,86	0,80	0,89	1,06	1,11	1,06	54,97%	1,91E-01	1,25	1,158333333	0,15620499	0,14445299	0,200748599	0,18695829	0,043173095	0,033986855	20	14	23	15	4	4	5	6	6	237,72
MS_run02	gi|23502575|ref|NP_698702.1|	unnamed protein product [Bru	0,76	2,47	0,75	2,08	0,98	2,79				1,34282624	0,967883705	0,0212132	0,25455844	1,979898987	0,03823577	1,043687697	2,661403626	3	26	95	3	108	108	2	3	3	127,35
MS_run03	gi|23502575|ref|NP_698702.1|	unnamed protein product [Bru	0,79	0,93	0,79	0,89	1,00	1,13				1,27091377	1,070707071	0,11786291	0,11030261	0,157860698	0,20328702	0,244476551	0,153563967	15	11	18	16	23	16	5	6	6	281,72
MS_run01	gi|23502584|ref|NP_698711.1|	tkt gene product [Brucella suis	1,38	1,26	1,28	1,17	0,93	0,91	1,67	39,10%	1,90E-04	0,78125	0,794871795	0,18875911	0,11937336	0,121119775	0,11150044	0,179734141	0,259539088	15	13	10	14	23	21	5	13	13	693,58
MS_run02	gi|23502584|ref|NP_698711.1|	tkt gene product [Brucella suis	1,81	1,76	1,54	1,30	0,85	0,84				0,63717532	0,543918919	0,1474223	0,23599082	0,185022521	0,27802436	0,231217779	0,26864077	10	28	14	44	43	15	4	9	9	651,23
MS_run03	gi|23502584|ref|NP_698711.1|	tkt gene product [Brucella suis	2,24	2,36	1,93	2,01	0,86	1,04				0,51813472	0,423076923	0,29482198	0,15498387	0,337490741	0,07087986	0,092462988	0,614167966	15	18	17	14	22	26	5	11	11	647,01
MS_run01	gi|23502585|ref|NP_698712.1|	gap gene product [Brucella sui:	0,96	0,91	0,94	0,87	0,98	0,93	0,95	5,67%	4,48E-01	1,06382979	1,094890055	0,15142655	0,1547848	0,07436397	0,16954753	0,076903667	0,075101877	16	16	9	16	7	8	6	12	12	565,21
MS_run02	gi|23502585|ref|NP_698712.1|	gap gene product [Brucella sui:	1,06	1,03	0,92	0,96	0,87	1,04				1,08695652	0,966292135	0,12601587	0,22255336	0,132664992	0,13416909	0,143384361	0,142535815	14	26	14	12	15	14	5	10	10	489,32
MS_run03	gi|23502585|ref|NP_698712.1|	gap gene product [Brucella sui:	1,02	0,90	0,98	0,90	0,97	0,91				1,01010101	1,18126428	0,15088627	0,13841363	0,289251909	0,0204158	0,536313085	0,429757763	15	14	32	2	45	48	4	10	10	495,41
MS_run02	gi|23502591|ref|NP_698718.1|	unnamed protein product [Bru	0,88	0,81	0,80	0,74	0,91	0,93	0,81	4,94%	3,19E-02	1,25	1,22972973	0	0	0	0	0	0	0	0	0	0	0	0	1	2	2	49,64
MS_run01	gi|23502594|ref|NP_698721.1|	unnamed protein product [Bru	0,75	0,75	0,65	0,63	0,87	0,97	0,72	7,83%	3,44E-04	1,54174573	1,433057638	0,04242641	0,13435029	0,141421356	0,10063189	0,53494483	0,279992093	7	16	22	7	37	37	2	3	3	178,53
MS_run02	gi|23502594|ref|NP_698721.1|	unnamed protein product [Bru	0,69	0,62	0,84	0,75	1,21	0,89				1,19047619	1,613333333	0	0	0	0	0	0	0	0	0	0	0	0	1	3	3	138,98
MS_run03	gi|23502594|ref|NP_698721.1|	unnamed protein product [Bru	0,85	0,79	0,74	0,62	0,86	0,84				1,44230769	1,280669599	0,22664216	0,11135529	0,088881944	0,43696496	0,29830503	0,164888954	31	13	14	30	23	21	4	6	6	252,07
MS_run01	gi|23502605|ref|NP_698732.1|	pyk gene product [Brucella sui:	1,51	1,57	1,09	1,27	0,72	1,17	1,11	24,05%	7,94E-02	0,91743119	0,637795276	0,27428695	0,12727922	0,211974841	0,17464224	0,104395808	0,204439389	25	18	17	19	16	13	3	12	12	863,32
MS_run02	gi|23502605|ref|NP_698732.1|	pyk gene product [Brucella sui:	1,22	1,08	1,11	0,98	0,91	0,88				0,81809751	0,927845874	0,23515952	0,24378953	0,204205779	0,37054358	0,247465097	0,261790585	21	27	21	45	27	24	4	12	12	796,34
MS_run03	gi|23502605|ref|NP_698732.1|	pyk gene product [Brucella sui:	0,91	0,72	1,05	0,85	1,16	0,81				1,03102907	1,469842724	0,41012193	0,12727922	0,367695526	0,40271203	0,486088674	0,238010821	39	11	43	39	33	33	2	11	11	600,44
MS_run01	gi|23502609|ref|NP_698736.1|	unnamed protein product [Bru	0,94	1,04	1,15	1,27	1,22	1,10	1,08	20,57%	2,08E-01	0,86956522	0,960629921	0	0	0	0	0	0	0	0	0	0	0	0	1	2	2	94,04
MS_run02	gi|23502609|ref|NP_698736.1|	unnamed protein product [Bru	0,85	0,75	1,39	1,22	1,63	0,88				0,71942446	1,336065574	0	0	0	0	0	0	0	0	0	0	0	0	1	3	3	120,72
MS_run01	gi|23502624|ref|NP_698751.1|	fdxA gene product [Brucella su	0,43	0,43	0,42	0,42	0,98	1,00	0,48	7,72%	1,74E-07	2,38095238	2,333333333	0	0	0	0	0	0	0	0	0	0	0	0	1	2	2	53,86
MS_run02	gi|23502624|ref|NP_698751.1|	fdxA gene product [Brucella su	0,54	0,54	0,56	0,55	1,04	0,99				1,83306056	1,881578947	0,12727922	0,18384776	0,021213203	0,41662593	0,403174919	0,116556063	23	18	4	23	21	21	2	4	4	130,59
MS_run03	gi|23502624|ref|NP_698751.1|	fdxA gene product [Brucella su	0,43	0,35	0,62	0,51	1,45	0,81				1,61290323	2,856719368	0	0,2192031	0,06363961	0	0,074064544	0,009078648	0	15	13	0	3	3	2	3	3	113,3
MS_run01	gi|23502630|ref|NP_698757.1|	unnamed protein product [Bru	0,97	0,96	1,12	1,07	1,16	0,96	1,01	17,67%	3,32E-01	0,96436603	1,196455505	0,417193	0,16263456	0,410121933	0,36083117	0,610586172	0,490392044	37	14	38	37	51	51	2	4	4	215,39
MS_run02	gi|23502630|ref|NP_698757.1|	unnamed protein product [Bru	0,76	1,30	0,66	0,82	0,87	1,25				1,35135135	0,689971584	0,12020815	0,33234019	0,195192213	0,40549736	0,11076276	0,538238403	18	38	24	30	16	41	3	4	4	262,92

MS_run03	gi|23502630|ref|NP_698757.1|	unnamed protein product [Bru	1,10	1,13	1,08	1,15	0,99	1,06				1,04166667	0,908668731	0,50911688	0,26162951	0,544472222	0,49104638	0,203594213	0,252926656	47	27	48	47	22	22	2	4	4	222,89
MS_run01	gi|23502631|ref|NP_698758.1|	rpmF gene product [Brucella su	0,83	0,78	0,91	0,85	1,09	0,93	1,18	35,08%	2,08E-01	1,0989011	1,282352941	0	0	0	0	0	0	0	0	0	0	0	0	1	1	1	24,1
MS_run02	gi|23502631|ref|NP_698758.1|	rpmF gene product [Brucella su	1,49	1,69	1,36	1,54	0,91	1,13				0,73529412	0,590909091	0	0	0	0	0	0	0	0	0	0	0	0	1	1	1	23,88
MS_run01	gi|23502634|ref|NP_698761.1|	ispG gene product [Brucella su	1,13	1,23	1,02	1,05	0,91	1,02	1,20	11,57%	5,43E-05	0,98039216	0,811881188	0,1762101	0,12070478	0,156838179	0,16909985	0,081274014	0,111256312	17	13	15	17	10	9	9	17	17	1790,49
MS_run02	gi|23502634|ref|NP_698761.1|	ispG gene product [Brucella su	1,30	1,31	1,14	1,08	0,88	0,95				0,87719298	0,765189048	0,12895182	0,15006665	0,103716647	0,09651409	0,120277601	0,184079318	11	17	10	11	16	14	7	16	16	1194,93
MS_run03	gi|23502634|ref|NP_698761.1|	ispG gene product [Brucella su	1,43	1,25	1,27	1,21	0,89	0,96				0,79052618	0,802168313	0,1857033	0,10370804	0,221665772	0,11248131	0,147005821	0,277826755	15	12	18	14	18	22	8	17	17	1516,81
MS_run01	gi|23502652|ref|NP_698779.1|	atpD gene product [Brucella su	0,96	0,82	0,89	0,78	0,93	0,88	0,86	5,63%	4,05E-03	1,12359551	1,222141001	0,16555691	0,26157304	0,194305289	0,20852964	0,265536393	0,2004744	19	28	25	19	22	24	13	20	20	1478,27
MS_run02	gi|23502652|ref|NP_698779.1|	atpD gene product [Brucella su	0,86	0,76	0,89	0,79	1,04	0,89				1,12359551	1,309606848	0,17994708	0,16845375	0,155924661	0,20552784	0,180073839	0,122394772	20	16	20	18	14	16	8	18	18	1327,74
MS_run03	gi|23502652|ref|NP_698779.1|	atpD gene product [Brucella su	0,90	0,86	0,91	0,87	1,01	0,96				1,1097561	1,158415842	0,19366638	0,15560366	0,261579452	0,20741399	0,194341561	0,147731159	21	15	30	19	17	17	8	18	18	1319,27
MS_run01	gi|23502653|ref|NP_698780.1|	atpG gene product [Brucella su	1,28	1,75	0,77	1,05	0,60	1,36	1,06	46,33%	1,32E-01	1,2987013	0,571428571	0	0	0	0	0	0	0	0	0	0	0	0	1	5	5	272,52
MS_run03	gi|23502653|ref|NP_698780.1|	atpG gene product [Brucella su	0,38	0,45	1,30	1,52	3,41	1,17				0,76923077	2,243421053	0	0	0	0	0	0	0	0	0	0	0	0	1	5	5	289,82
MS_run01	gi|23502654|ref|NP_698781.1|	atpA gene product [Brucella su	0,98	1,02	0,93	0,89	0,95	0,96	0,94	8,92%	1,92E-01	1,07526882	0,97752809	0,18836618	0,23451433	0,178426168	0,22605152	0,213499384	0,199760356	20	25	20	21	22	20	16	20	20	1386,72
MS_run02	gi|23502654|ref|NP_698781.1|	atpA gene product [Brucella su	0,98	0,98	0,89	0,88	0,91	0,98				1,12359551	1,025	0,14062361	0,23825257	0,161549417	0,15590222	0,232488184	0,181853751	16	26	18	14	23	19	11	17	17	1178
MS_run03	gi|23502654|ref|NP_698781.1|	atpA gene product [Brucella su	1,14	0,96	0,92	0,76	0,80	0,83				1,09318996	1,044776119	0,11710038	0,17458795	0,099618319	0,15543161	0,247423698	0,201328815	13	22	13	14	24	21	11	19	19	1163,1
MS_run01	gi|23502655|ref|NP_698782.1|	atpH gene product [Brucella su	1,24	0,86	0,92	0,67	0,74	0,72	1,48	52,62%	1,29E-02	1,08695652	0,787847447	0,260832	0,1942507	0,021213203	0,26498159	0,234757623	0,260489014	28	26	3	24	30	30	3	7	7	282,05
MS_run02	gi|23502655|ref|NP_698782.1|	atpH gene product [Brucella su	1,60	1,31	1,45	1,56	0,91	1,08				0,55555556	0,865548354	0,72972027	0,19091883	0,554075807	0,24654484	0,390795791	0,737058286	50	21	36	44	45	56	4	7	7	436,63
MS_run03	gi|23502655|ref|NP_698782.1|	atpH gene product [Brucella su	1,78	2,72	1,85	1,81	1,04	0,98				0,79801038	0,924355878	1,47785317	0,67175144	1,258650071	0,63920985	1,01391825	2,978444812	80	65	70	80	110	110	2	8	8	479,4
MS_run01	gi|23502658|ref|NP_698785.1|	unnamed protein product [Bru	0,93	0,85	0,99	0,90	1,07	0,90	0,90	7,34%	6,06E-02	1,01010101	1,183035714	0,15628119	0,17220731	0,130322402	0,13899236	0,210302478	0,139656094	16	16	15	14	18	17	8	10	10	629,09
MS_run02	gi|23502658|ref|NP_698785.1|	unnamed protein product [Bru	0,90	0,84	0,92	0,79	1,02	0,86				1,08695652	1,189189189	0,08734987	0,20327322	0,182071415	0,10374318	0,19252167	0,121919046	9	20	23	10	16	14	5	9	9	485,88
MS_run03	gi|23502658|ref|NP_698785.1|	unnamed protein product [Bru	0,97	0,77	1,02	0,91	1,05	0,89				0,98039216	1,3	0,16688319	0,16440803	0,103585713	0,16776631	0,193650646	0,114859934	16	16	11	17	15	15	5	9	9	486,58
MS_run03	gi|23502675|ref|NP_698802.1|	unnamed protein product [Bru	2,91	3,47	2,97	3,54	1,02	1,19	3,22	28,40%	5,45E-04	0,33670034	0,288135593	0	0	0	0	0	0	0	0	0	0	0	0	1	1	1	35,74
MS_run01	gi|23502677|ref|NP_698804.1|	rpsP gene product [Brucella su	0,94	0,94	0,78	0,82	0,83	1,05	0,90	23,97%	4,89E-01	1,28289474	1,06097561	0,02828427	0,05656854	0,098657657	0,04652018	0,086733148	0,069420358	4	7	12	4	8	7	3	4	4	254,05
MS_run02	gi|23502677|ref|NP_698804.1|	rpsP gene product [Brucella su	1,32	1,35	1,02	1,04	0,77	1,02				0,98039216	0,740384615	0,07767453	0,12342339	0,02081666	0,06959016	0,131574452	0,217551392	8	16	2	7	18	16	3	4	4	181,47
MS_run03	gi|23502677|ref|NP_698804.1|	rpsP gene product [Brucella su	0,73	0,65	0,68	0,58	0,92	0,85				1,5	1,545454545	0,10606602	0,06806859	0,120208153	0,23570226	0,213985734	0,083756013	16	7	21	16	14	13	3	4	4	256,22
MS_run01	gi|23502690|ref|NP_698817.1|	unnamed protein product [Bru	0,86	0,82	1,19	1,18	1,38	1,00	1,04	15,73%	4,33E-01	0,87260677	1,126626627	0,30405592	0,37421028	0,102469508	0,22389979	0,32328512	0,109023279	26	27	9	26	29	13	4	8	8	448,88
MS_run02	gi|23502690|ref|NP_698817.1|	unnamed protein product [Bru	1,21	1,09	1,17	1,06	0,97	0,91				0,85470085	0,91509434	0	0	0	0	0	0	0	0	0	0	0	0	1	6	6	248,65
MS_run03	gi|23502690|ref|NP_698817.1|	unnamed protein product [Bru	1,12	0,69	1,05	0,99	0,94	0,94				0,95238095	0,960939112	0,2330236	0,21213203	0,049497475	0,17790944	0,263650857	0,252979139	22	23	5	19	27	37	3	8	8	356,72
MS_run01	gi|23502703|ref|NP_698830.1|	rplU gene product [Brucella su	1,08	1,02	1,23	1,10	1,13	0,89	1,05	11,75%	2,67E-01	0,75757576	0,985436893	0,32968419	0,19857828	0,227229693	0,09078093	0,063069327	0,064643656	27	18	21	12	6	6	4	4	4	169,56
MS_run02	gi|23502703|ref|NP_698830.1|	rplU gene product [Brucella su	1,14	1,02	1,16	1,16	1,02	1,00				0,86206897	0,982300885	0,11135529	0,13435029	0,257358375	0,0888341	0,163885662	0,186940177	10	13	22	10	17	18	3	3	3	125,62
MS_run03	gi|23502703|ref|NP_698830.1|	rplU gene product [Brucella su	0,88	0,90	1,06	0,84	1,21	0,79				0,95024653	1,111940299	0,32653484	0,26620794	0,066583281	0,27896019	0,005812625	0,159500618	31	22	8	29	1	18	4	3	3	172
MS_run01	gi|23502717|ref|NP_698844.1|	clpB gene product [Brucella su	1,06	1,10	1,01	1,06	0,95	1,05	1,04	6,71%	4,89E-02	0,9950495	0,91	0,19093047	0,18271269	0,257209383	0,20784842	0,225721023	0,26113864	19	19	24	21	25	24	10	27	27	1486,2
MS_run02	gi|23502717|ref|NP_698844.1|	clpB gene product [Brucella su	1,12	1,14	1,06	1,08	0,95	1,01				0,94415249	0,879699248	0,21033562	0,16204056	0,266993847	0,24296673	0,234424035	0,334719718	20	17	25	26	27	29	9	25	25	1234,95
MS_run03	gi|23502717|ref|NP_698844.1|	clpB gene product [Brucella su	1,01	0,88	1,02	0,97	1,01	0,96				0,98639456	1,132173493	0,08066391	0,17862571	0,179814719	0,08705927	0,258512136	0,253979653	8	18	19	9	23	29	8	24	24	1258,45
MS_run01	gi|23502718|ref|NP_698845.1|	unnamed protein product [Bru	2,40	0,83	2,14	0,71	0,89	0,33	1,24	61,22%	5,29E-02	0,95628415	1,338906089	2,15667568	0,16970563	0,197989899	0,96599287	0,61238804	0,381512158	101	19	28	101	46	46	2	3	3	171,22
MS_run02	gi|23502718|ref|NP_698845.1|	unnamed protein product [Bru	1,71	1,94	1,06	1,20	0,62	1,13				0,94339623	0,516666667	0	0	0	0	0	0	0	0	0	0	0	0	1	3	3	145,11
MS_run03	gi|23502718|ref|NP_698845.1|	unnamed protein product [Bru	0,90	0,54	0,94	0,55	1,04	0,58				0,91743119	1,722222222	0,21213203	0,07767453	0,007071068	0,51516733	0,484784365	0,050477455	23	7	1	56	28	9	3	5	5	225,13
MS_run01	gi|23502724|ref|NP_698851.1|	unnamed protein product [Bru	0,93	0,92	0,92	0,89	0,98	0,97	0,95	16,64%	2,58E-01	1,04166667	1,053763441	0,06363961	0,02516611	0,056568542	0,27915995	0,545912744	0,044945775	7	3	6	27	52	5	3	7	7	491,48
MS_run02	gi|23502724|ref|NP_698851.1|	unnamed protein product [Bru	0,96	1,02	0,80	0,82	0,83	1,03				1,25	0,947916667	0,14364308	0,26857649	0,111355287	0,18448139	0,248043247	0,04737138	18	32	14	15	26	5	3	9	9	523,45
MS_run03	gi|23502724|ref|NP_698851.1|	unnamed protein product [Bru	1,33	0,79	1,25	0,80	0,94	0,64				0,86805556	1,258689724	0,64137223	0,16822604	1,841991676	0,38316984	0,544190017	0,004827637	51	18	230	44	43	1	4	10	10	647,86
MS_run01	gi|23502729|ref|NP_698856.1|	grxC gene product [Brucella su	1,14	1,13	1,23	1,08	1,08	0,88	1,07	16,82%	4,15E-01	0,79410096	0,877808136	0,29022979	0,13012814	0,35907288	0,02673877	0,010518589	0,39807473	24	12	33	3	1	35	3	3	3	109,25
MS_run02	gi|23502729|ref|NP_698856.1|	grxC gene product [Brucella su	0,93	1,03	1,28	1,42	1,38	1,11				0,78577592	0,975090854	0,07778175	0,13435029	0,205060967	0,04793649	0,046362392	0,048816524	6	10	14	6	5	5	2	2	2	66,68
MS_run03	gi|23502729|ref|NP_698856.1|	grxC gene product [Brucella su	0,86	0,90	0,89	0,93	1,03	1,04				1,12359551	1,107526882	0	0	0	0	0	0	0	0	0	0	0	0	1	1	1	36,03
MS_run01	gi|23502737|ref|NP_698864.1|	unnamed protein product [Bru	1,26	0,99	1,54	1,60	1,22	1,04	1,08	31,24%	9,57E-02	0,62588014	0,959168956	0,306159	0,55154329	0,645523044	0,03319231	0,066582651	0,210308909	20	45	40	5	7	21	3	9	9	484,89
MS_run02	gi|23502737|ref|NP_698864.1|	unnamed protein product [Bru	1,52	1,08	0,96	0,68	0,63	0,71				1,04166667	0,926470588	0	0	0	0	0	0	0	0	0	0	0	0	1	8	8	537,86
MS_run03	gi|23502737|ref|NP_698864.1|	unnamed protein product [Bru	0,98	0,82	0,85	0,70	0,87	0,82				1,21428571	1,238702322	0,21213203	0,20506097	0,035355339	0,30304576	0,232037735	0,153926022	25	24	5	25	19	19	2	8	8	422,63
MS_run01	gi|23502752|ref|NP_698879.1|	sdhB gene product [Brucella su	0,55	0,75	0,56	0,76	1,01	1,36	0,80	15,55%	2,11E-03	1,78571429	1,328947368	0	0	0	0	0	0	0	0	0	0	0	0	1	6	6	378,14
MS_run02	gi|23502752|ref|NP_698879.1|	sdhB gene product [Brucella su	0,95	1,03	0,94	1,02	0,99	1,09				1,06382979	0,970588235	0	0	0	0	0	0	0	0	0	0	0	0	1	6	6	449,27
MS_run03	gi|23502752|ref|NP_698879.1|	sdhB gene product [Brucella su	0,75	0,87	0,70	0,72	0,93	1,02				1,38888889	1,144385027	0,02828427	0,28284271	0,106066017	0,53689047	0,166215187	0,101714494	4	30	15	39	15	12	3	7	7	390,84
MS_run01	gi|23502753|ref|NP_698880.1|	sdhA gene product [Brucella su	1,03	1,18	0,85	0,95	0,82	1,12	1,21	45,36%	1,03E-01	1,18546577	0,892532394	0,04949747	0,12727922	0,16263456	0,0694409	0,288291887	0,381814244	6	16	17	6	32	32	2	3	3	204,5
MS_run02	gi|23502753|ref|NP_698880.1|	sdhA gene product [Brucella su	0,83	0,81	0,94	0,91	1,13	0,97				1,06382979	1,241758242	0	0	0	0	0	0	0	0	0	0	0	0	1	4	4	224,84
MS_run03	gi|23502753|ref|NP_698880.1|	sdhA gene product [Brucella su	2,18	2,09	1,39	1,32	0,64	0,95				0,73117939	0,481687715	0,2192031	0,06363961	0,028284271	0,11572331	0,058533179	0,254149546	16	10	2	16	12	12	2	5	5	228,36
MS_run01	gi|23502754|ref|NP_698881.1|	unnamed protein product [Bru	2,55	1,11	1,68	0,73	0,66	0,43	1,52	68,42%	9,00E-02	0,5952381	0,904109589	0	0	0	0	0	0	0	0	0	0	0	0	1	1	1	33,32
MS_run01	gi|23502757|ref|NP_698884.1|	leuC gene product [Brucella su	1,41	0,98	0,86	0,91	0,61	1,06	1,12	20,11%	4,95E-02	1,1627907	0,942028986	0,14	0,05656854	0,17691806	0,19530138	0,252238299	0,122081684	16	9	19	17	27	13	3	9	9	471,99
MS_run02	gi|23502757|ref|NP_698884.1|	leuC gene product [Brucella su	1,38	1,53	1,07	1,18	0,78	1,10				0,94456215	0,671865825	0,15556349	0,04949747	0,190918831	0,13732653	0,151293032	0,343879924	15	6	16	15	23	23	2	7	7	356,49
MS_run03	gi|23502757|ref|NP_698884.1|	leuC gene product [Brucella su	0,99	1,03	1,07	1,10	1,08	1,03				0,9464984	0,981818182	0,13435029	0,15556349	0	0,11940125	0,141421356	0,148245552	13	14	0	13	14	14	2	7	7	374,01
MS_run01	gi|23502758|ref|NP_698885.1|	rplS gene product [Brucella sui	0,90	1,05	0,96	1,09	1,07	1,14	0,93	10,75%	1,04E-01	1,01588284	0,958115183	0,09848858	0,02828427	0,196977156	0,25309571	0,180501966	0,1511132	10	3	18	25	19	14	4	5	5	218,36
MS_run02	gi|23502758|ref|NP_698885.1|	rplS gene product [Brucella sui	0,98	0,82	1,10	0,98	1,12	0,89				0,90909091	1,224489796	0,04932883	0,05686241	0,13	0,03821981	0,144046878	0,112844726	4	5	13	4	12	14	3	5	5	223,63
MS_run03	gi|23502758|ref|NP_698885.1|	rplS gene product [Brucella sui	0,91	0,82	0,83	0,76	0,91	0,92				1,20481928	1,216216216	0,08326664	0,11269428	0,028284271	0,13639918	0,128739218	0,077392424	10	12	4	11	11	9	3	5	5	219,2
MS_run01	gi|23502768|ref|NP_698895.1|	lpdA-2 gene product [Brucella	1,00	1,29	1,12	1,44	1,11	1,30	1,21	24,19%	8,55E-02	0,89904854	0,771812081	0,24662725	0,2433105	0,236836793	0,15201112	0,034750953	0,358917792	22	22	16	17	5	28	4	14	14	873,96
MS_run02	gi|23502768|ref|NP_698895.1|	lpdA-2 gene product [Brucella	0,84	1,16	0,97	0,91	1,16	0,94				0,92529998	0,782857143	0,19313208	0,2510644	0,516268664	0,32518949	0,100367929	0,274189195	20	22	57	35	13	24	4	12	12	795,54
MS_run03	gi|23502768|ref|NP_698895.1|	lpdA-2 gene product [Brucella	1,62	1,50	1,42	1,30	0,88	0,92				0,70742926	0,686230316	0,06363961	0,10606602	0,16263456	0,03181662	0,168085546	0,367974647	4	12	13	4	24	24	2	11	11	607,25
MS_run01	gi|23502769|ref|NP_698896.1|	unnamed protein product [Bru	0,67	1,00	0,70	1,05	1,05	1,50	1,05	29,27%	2,44E-01	1,42857143	1	0	0	0	0	0	0	0	0	0	0	0	0	1	1	1	36,61
MS_run02	gi|23502769|ref|NP_698896.1|	unnamed protein product [Bru	1,15	0,92	1,62	1,30	1,41	0,80				0,61728395	1,084615385	0	0	0	0	0	0	0	0	0	0	0	0	1	1	1	26,03
MS_run01	gi|23502772|ref|NP_698899.1|	sucB gene product [Brucella su	1,16	1,08	1,10	1,06	0,94	0,97	1,04	9,95%	4,53E-01	0,91326105	0,925284611	0,17017638	0,26713649	0,191733943	0,13383973	0,192067786	0,228908963	16	28	18	15	21	21	8	8	8	600,32
MS_run02	gi|23502772|ref|NP_698899.1|	sucB gene product [Brucella su	1,05	1,04	1,14	1,11	1,09	0,97				0,87719298	0,964925625	0,09502631	0,23082461	0,069856997	0,0688955	0,196042632	0,202551987	8	21	6	8	20	20	6	8	8	428,68
MS_run03	gi|23502772|ref|NP_698899.1|	sucB gene product [Brucella su	0,77	0,94	1,04	1,01	1,35	0,97				0,96153846	0,991777293	0,26668333	0,272816	0,137295302	0,19680057	0,298792194	0,282251398	26	20	14	20	30	30	7	8	8	443,06
MS_run01	gi|23502773|ref|NP_698900.1|	sucA gene product [Brucella su	1,70	1,63	1,46	1,42	0,86	0,97	2,31	150,03%	4,55E-02	0,68493151	0,613793103	0,11590226	0,03	0,130766968	0,062006	0,066071717	0,157261743	8	3	9	9	11	10	3	12	12	585,1
MS_run02	gi|23502773|ref|NP_698900.1|	sucA gene product [Brucella su	0,80	1,41	0,67	1,21	0,84	1,81				1,89909297	0,714660494	0,4384062	0,31112698	0,579827561	1,24264797	0,085332948	0,168276781	65	37	48	65	12	12	2	9	9	413,91
MS_run03	gi|23502773|ref|NP_698900.1|	sucA gene product [Brucella su	3,75	4,46	4,26	4,98	1,14	1,17				0,23802074	0,226263437	0,70710678	0,27577164	0,509116882	0,03950847	0,032244399	0,636294296	17	24	10	17	14	14	2	8	8	421,27
MS_run01	gi|23502775|ref|NP_698902.1|	sucD gene product [Brucella su	1,28	1,25	1,16	1,09	0,91	0,94	1,32	15,19%	1,87E-05	0,86206897	0,800505051	0,07021396	0,17944358	0,143596657	0,05124336	0,20279528	0,240040862	6	20	13	6	25	19	6	7	7	369,32
MS_run02	gi|23502775|ref|NP_698902.1|	sucD gene product [Brucella su	1,57	1,32	1,17	1,24	0,74	1,06				0,85914454	0,756756757	0,08693868	0,06831301	0,181383571	0,06641531	0,259293236	0,452905581	7	9	15	8	34	34	5	8	8	396,87
MS_run03	gi|23502775|ref|NP_698902.1|	sucD gene product [Brucella su	1,61	1,36	1,42	1,34	0,89	0,94				0,7024549	0,736590485	0,17935068	0,08981462	0,21	0,09074924	0,132832732	0,180936273	13	10	16	13	18	13	5	6	6	284,75
MS_run01	gi|23502776|ref|NP_698903.1|	sucC gene product [Brucella su	1,08	1,04	0,98	0,88	0,91	0,90	1,04	11,21%	8,06E-02	1,02040816	0,962025316	0,13616835	0,14982414	0,163067192	0,14043984	0,194490995	0,193797944	14	16	19	14	20	19	14	15	15	891,83
MS_run02	gi|23502776|ref|NP_698903.1|	sucC gene product [Brucella su	0,91	1,04	0,94	0,96	1,03	1,02				1,06382979	0,961832061	0,23295803	0,26528287	0,207002415	0,26869566	0,14975284	0,193451155	25	26	22	25	16	19	9	12	12	695,99
MS_run03	gi|23502776|ref|NP_698903.1|	sucC gene product [Brucella su	1,23	1,25	1,07	1,12	0,87	1,05				0,93898783	0,8	0,49933289	0,20170257	0,427007862	0,28683434	0,253910518	0,598284881	47	23	38	31	32	48	10	12	12	749,13
MS_run01	gi|23502777|ref|NP_698904.1|	mdh gene product [Brucella su	1,19	1,01	1,06	0,94	0,89	0,89	1,07	8,65%	7,62E-04	0,94788859	0,988636364	0,15107393	0,15705239	0,103077641	0,1545147	0,137759028	0,150658107	14	18	11	16	14	15	13	14	14	1206,54
MS_run02	gi|23502777|ref|NP_698904.1|	mdh gene product [Brucella su	1,25	1,07	1,17	1,05	0,94	0,89				0,85107924	0,933236621	0,11904608	0,14893562	0,098206132	0,08515023	0,148323552	0,154651959	10	16	9	10	16	14	14	14	14	1214,52
MS_run03	gi|23502777|ref|NP_698904.1|	mdh gene product [Brucella su	1,08	0,99	1,06	1,00	0,99	0,94				0,94339623	1,009708738	0,13261702	0,19469634	0,121589179	0,14304403	0,113642003	0,09144007	13	20	12	15	11	9	13	14	14	1263,45
MS_run01	gi|23502793|ref|NP_698920.1|	unnamed protein product [Bru	1,46	1,47	1,15	1,21	0,79	1,05	1,19	20,54%	9,76E-02	0,8698283	0,679451797	0,24094951	0,17866169	0,274633956	0,16813161	0,086836532	0,1662482	21	23	23	19	13	11	7	9	9	463,16
MS_run02	gi|23502793|ref|NP_698920.1|	unnamed protein product [Bru	1,10	1,42	1,23	1,38	1,12	1,12				0,81306187	0,728729604	0,19113695	0,30423401	0,243909136	0,14939131	0,279152471	0,462873086	16	27	18	18	38	33	5	7	7	372,03
MS_run03	gi|23502793|ref|NP_698920.1|	unnamed protein product [Bru	0,83	1,16	0,85	1,07	1,02	1,26				1,17647059	0,861331756	0,26994444	0,25518621	0,214569336	0,34497576	0,228428877	0,236823712	32	25	20	29	27	20	6	8	8	434,11
MS_run01	gi|23502795|ref|NP_698922.1|	secA gene product [Brucella su	1,20	1,29	1,30	1,39	1,08	1,07	1,30	6,60%	9,15E-03	0,76923077	0,776978417	0	0	0	0	0	0	0	0	0	0	0	0	1	7	7	353,94
MS_run01	gi|23502815|ref|NP_698942.1|	unnamed protein product [Bru	0,55	0,43	0,57	0,51	1,04	0,89	0,52	4,37%	1,07E-12	1,76799007	2,318181818	0,41705315	0,0212132	0,149749791	0,59265395	0,52858487	0,13672272	73	2	29	34	23	32	5	6	6	415,28
MS_run02	gi|23502815|ref|NP_698942.1|	unnamed protein product [Bru	0,46	0,49	0,50	0,53	1,08	1,06				2	2,037735849	0,06027714	0,08144528	0,049328829	0,23401557	0,042566391	0,009896549	12	8	9	12	2	2	3	5	5	338,94
MS_run03	gi|23502815|ref|NP_698942.1|	unnamed protein product [Bru	0,51	0,59	0,54	0,56	1,07	1,04				1,06382979	1,745798319	0,29687259	0,10606602	0,091651514	0,7291133	0,419572707	0,130996935	55	10	16	69	24	22	4	5	5	385,83
MS_run01	gi|23502817|ref|NP_698944.1|	hbd gene product [Brucella sui	1,24	0,96	1,41	1,10	1,14	0,78	1,14	21,10%	5,03E-02	0,70921986	1,036363636	0	0	0	0	0	0	0	0	0	0	0	0	1	7	7	379,43

MS_run02	gi|23502817|ref|NP_698944.1|	hbd gene product [Brucella sui	1,38	1,37	1,31	1,30	0,95	0,99				0,76335878	0,730769231	0	0	0	0	0	0	0	0	0	0	0	0	1	7	7	380,29
MS_run03	gi|23502817|ref|NP_698944.1|	hbd gene product [Brucella sui	0,95	1,09	0,76	0,87	0,80	1,14				1,31578947	0,91954023	0	0	0	0	0	0	0	0	0	0	0	0	1	7	7	349,65
MS_run01	gi|23502818|ref|NP_698945.1|	etfA gene product [Brucella sui	1,08	1,13	0,96	0,99	0,89	1,03	1,02	7,43%	1,91E-02	1,04166667	0,881188119	0,05029911	0,11495341	0,061023025	0,05538488	0,07586094	0,100489477	5	13	6	5	9	9	7	11	11	814,42
MS_run02	gi|23502818|ref|NP_698945.1|	etfA gene product [Brucella sui	1,07	1,00	1,04	1,00	0,97	0,96				0,96153846	1	0,21210959	0,18924663	0,125777581	0,15956646	0,213153611	0,2751801	20	20	13	17	21	28	7	11	11	741,22
MS_run03	gi|23502818|ref|NP_698945.1|	etfA gene product [Brucella sui	1,17	0,93	0,98	0,92	0,84	0,94				1,02040816	1,076086957	0,11760102	0,25986535	0,286583321	0,13841098	0,304179495	0,380647603	12	31	31	14	28	41	5	9	9	585,59
MS_run01	gi|23502819|ref|NP_698946.1|	etfB gene product [Brucella sui	1,17	1,00	1,09	1,01	0,94	0,93	1,00	10,22%	6,53E-02	0,91743119	1,023529412	0,178577	0,10509784	0,183492355	0,15856437	0,154902537	0,230507975	16	11	18	17	15	23	14	12	12	1122,52
MS_run02	gi|23502819|ref|NP_698946.1|	etfB gene product [Brucella sui	1,19	0,94	1,02	0,94	0,86	0,92				0,98124098	1,069163861	0,11730724	0,19491287	0,13355285	0,1123558	0,15503843	0,161587896	12	23	14	11	15	17	15	12	12	1097,99
MS_run03	gi|23502819|ref|NP_698946.1|	etfB gene product [Brucella sui	0,95	0,85	0,98	0,85	1,03	0,87				1,02588384	1,172413793	0,07837942	0,16259962	0,121363466	0,07879384	0,225812444	0,151399679	8	16	14	8	19	18	14	12	12	1310,24
MS_run01	gi|23502829|ref|NP_698956.1|	argH gene product [Brucella su	1,23	1,52	1,36	1,67	1,11	1,23	1,44	16,84%	7,19E-02	0,80492424	0,659967542	0,56568542	0,3959798	0,558614357	0,33480435	0,016305942	0,037448405	42	36	33	42	2	2	2	13	13	848,45
MS_run03	gi|23502836|ref|NP_698963.1|	tyrC gene product [Brucella sui	1,38	1,50	0,99	1,08	0,72	1,09	1,24	20,85%	9,79E-02	1,01010101	0,666666667	0	0	0	0	0	0	0	0	0	0	0	0	1	3	3	111,45
MS_run01	gi|23502841|ref|NP_698968.1|	ppa gene product [Brucella sui	0,99	0,76	0,78	0,78	0,79	0,99	0,73	15,43%	4,06E-02	1,27808531	1,315062876	0,17269916	0,17097758	0,10214369	0,21950257	0,202419779	0,159582341	22	22	13	17	15	21	4	3	3	190,97
MS_run02	gi|23502841|ref|NP_698968.1|	ppa gene product [Brucella sui	0,89	0,55	1,00	0,61	1,12	0,61				1,00160256	1,837365591	0,05656854	0,04242641	0,014142136	0,0566592	0,112148656	0,03328225	6	4	2	6	6	6	2	1	1	107,33
MS_run03	gi|23502841|ref|NP_698968.1|	ppa gene product [Brucella sui	0,68	0,60	0,61	0,57	0,90	0,93				1,63934426	1,68	0,18229098	0,18156725	0,162147053	0,42118595	0,342141625	0,144653199	30	20	29	26	20	24	5	3	3	229,85
MS_run01	gi|23502854|ref|NP_698981.1|	unnamed protein product [Bru	0,81	0,91	0,97	1,02	1,20	1,05	0,94	5,37%	3,51E-03	1,03136629	1,101860254	0,14384598	0,3468429	0,34208917	0,15713818	0,193668707	0,130193168	15	29	34	15	18	14	4	6	6	292,07
MS_run02	gi|23502854|ref|NP_698981.1|	unnamed protein product [Bru	0,99	0,94	0,99	0,98	0,99	0,99				1,02689741	1,065513627	0,15979153	0,1734695	0,236361305	0,16607975	0,133098657	0,111941777	16	18	24	16	12	12	4	6	6	345,58
MS_run03	gi|23502854|ref|NP_698981.1|	unnamed protein product [Bru	0,91	0,96	0,91	0,93	1,00	1,02				1,0989011	1,0375	0,24110164	0,36687873	0,051961524	0,2617739	0,257949866	0,253852439	26	37	6	24	25	26	5	7	7	351,6
MS_run01	gi|23502863|ref|NP_698990.1|	rpmB gene product [Brucella s	0,66	0,85	0,56	0,65	0,85	1,16	0,85	18,70%	2,05E-01	1,58730159	1,132144544	0,09899495	0,20502032	0,075055535	0,54673141	0,286848467	0,122236263	18	24	12	34	25	14	4	5	5	357,99
MS_run02	gi|23502863|ref|NP_698990.1|	rpmB gene product [Brucella s	1,26	0,72	0,84	0,80	0,67	0,95				1,19047619	0,815441176	0,20033306	0,33234019	0,065574385	0,28675387	0,437782287	0,23691099	24	50	8	24	54	33	3	4	4	322,06
MS_run03	gi|23502863|ref|NP_698990.1|	rpmB gene product [Brucella s	0,96	0,89	1,08	0,89	1,12	0,82				0,94413847	1,12844249	0,36551334	0,40169433	0,064291005	0,27547484	0,13100889	0,095782053	34	36	7	29	12	11	4	6	6	467,08
MS_run01	gi|23502870|ref|NP_698997.1|	unnamed protein product [Bru	0,87	0,73	0,98	0,82	1,13	0,84	0,75	13,37%	3,90E-04	1,02040816	1,37804878	0	0	0	0	0	0	0	0	0	0	0	0	1	9	9	518,97
MS_run02	gi|23502870|ref|NP_698997.1|	unnamed protein product [Bru	0,56	0,54	0,64	0,62	1,14	0,97				1,5625	1,838709677	0	0	0	0	0	0	0	0	0	0	0	0	1	9	9	525,96
MS_run03	gi|23502870|ref|NP_698997.1|	unnamed protein product [Bru	0,76	0,72	0,90	0,86	1,19	0,96				1,11111111	1,38372093	0	0	0	0	0	0	0	0	0	0	0	0	1	9	9	453,42
MS_run01	gi|23502880|ref|NP_699007.1|	accA gene product [Brucella su	0,99	2,29	1,18	2,72	1,19	2,31	1,86	65,75%	2,57E-01	0,84745763	0,4375	0	0	0	0	0	0	0	0	0	0	0	0	1	7	7	475,92
MS_run03	gi|23502880|ref|NP_699007.1|	accA gene product [Brucella su	1,37	2,52	1,34	2,47	0,98	1,84				0,74626866	0,396761134	0	0	0	0	0	0	0	0	0	0	0	0	1	8	8	644,76
MS_run01	gi|23502901|ref|NP_699028.1|	unnamed protein product [Bru	0,97	1,25	0,98	1,26	1,01	1,29	0,85	25,08%	1,06E-02	1,02040816	0,801587302	0	0	0	0	0	0	0	0	0	0	0	0	1	3	3	131,47
MS_run02	gi|23502901|ref|NP_699028.1|	unnamed protein product [Bru	0,74	0,92	0,81	1,01	1,10	1,25				1,2345679	1,089108911	0	0	0	0	0	0	0	0	0	0	0	0	1	3	3	132,25
MS_run03	gi|23502901|ref|NP_699028.1|	unnamed protein product [Bru	0,53	0,58	0,48	0,65	0,91	1,35				0,83333333	1,711805556	0,33941125	0,12727922	0,098994949	0,7856742	0,334390499	0,002954645	71	14	15	94	20	1	3	3	3	198,51
MS_run01	gi|23502906|ref|NP_699033.1|	parB gene product [Brucella su	0,81	0,94	0,91	1,06	1,13	1,16	0,93	8,38%	6,90E-04	1,0989011	1,066037736	0	0	0	0	0	0	0	0	0	0	0	0	1	3	3	143,48
MS_run03	gi|23502906|ref|NP_699033.1|	parB gene product [Brucella su	0,83	0,95	0,91	1,04	1,10	1,14				1,0989011	1,057692308	0	0	0	0	0	0	0	0	0	0	0	0	1	3	3	138,66
MS_run01	gi|23502912|ref|NP_699039.1|	rho gene product [Brucella suis	1,41	1,39	0,90	0,89	0,64	0,99	1,42	48,85%	2,91E-03	1,11111111	0,719101124	0	0	0	0	0	0	0	0	0	0	0	0	1	6	6	297,74
MS_run02	gi|23502912|ref|NP_699039.1|	rho gene product [Brucella suis	2,28	1,17	1,64	0,84	0,72	0,51				0,6097561	0,857142857	0	0	0	0	0	0	0	0	0	0	0	0	1	5	5	277,4
MS_run03	gi|23502912|ref|NP_699039.1|	rho gene product [Brucella suis	2,19	2,04	1,18	1,10	0,54	0,93				0,84745763	0,490909091	0	0	0	0	0	0	0	0	0	0	0	0	1	6	6	259,49
MS_run01	gi|23502920|ref|NP_699047.1|	secB gene product [Brucella su	0,82	0,62	0,83	0,60	1,01	0,72	0,81	14,59%	4,21E-02	1,20481928	1,53030303	0,22810816	0,17058722	0,084852814	0,29992175	0,328401562	0,047028841	27	17	14	25	21	8	3	6	6	411,83
MS_run02	gi|23502920|ref|NP_699047.1|	secB gene product [Brucella su	0,94	0,78	1,15	0,96	1,23	0,83				0,90259791	1,307295174	0,31112698	0,04949747	0,205060967	0,24419354	0,228877212	0,136007467	27	4	21	27	18	18	2	5	5	430,43
MS_run03	gi|23502920|ref|NP_699047.1|	secB gene product [Brucella su	0,77	0,70	0,80	0,73	1,04	0,91				1,25313283	1,44375	0,05656854	0,00707107	0,106066017	0,08860987	0,220970869	0,107267412	7	1	15	7	15	15	2	7	7	484,53
MS_run02	gi|23502927|ref|NP_699054.1|	hslU gene product [Brucella su	1,12	0,68	0,85	0,52	0,76	0,61	0,98	30,43%	8,05E-02	1,17647059	1,461538462	0	0	0	0	0	0	0	0	0	0	0	0	1	3	3	195,27
MS_run03	gi|23502927|ref|NP_699054.1|	hslU gene product [Brucella su	1,52	1,30	1,00	0,86	0,66	0,86				1	0,76744186	0	0	0	0	0	0	0	0	0	0	0	0	1	3	3	154,43
MS_run01	gi|23502929|ref|NP_699056.1|	hisB gene product [Brucella sui	1,24	1,29	0,98	1,02	0,79	1,04	1,14	37,80%	4,80E-01	1,02040816	0,774509804	0	0	0	0	0	0	0	0	0	0	0	0	1	2	2	80,59
MS_run02	gi|23502929|ref|NP_699056.1|	hisB gene product [Brucella sui	0,93	0,62	1,28	2,25	1,38	1,76				0,78125	0,728888889	0,18036999	1,0323759	0,696562991	0,1086047	0,2954135	0,629944783	14	75	31	14	41	101	3	3	3	129,16
MS_run03	gi|23502929|ref|NP_699056.1|	hisB gene product [Brucella sui	1,06	0,93	1,13	0,99	1,07	0,88				0,88495575	1,080808081	0	0	0	0	0	0	0	0	0	0	0	0	1	1	1	40,43
MS_run01	gi|23502937|ref|NP_699064.1|	pckA gene product [Brucella su	1,13	1,31	1,35	1,38	1,19	1,02	1,32	33,71%	3,35E-02	0,74074074	0,761157092	0,24833445	0,01	0,225905585	0,16203827	0,141569074	0,2187245	18	1	16	22	19	17	5	17	17	1021,5
MS_run02	gi|23502937|ref|NP_699064.1|	pckA gene product [Brucella su	1,04	0,68	1,06	1,17	1,02	1,10				0,64935065	0,778194028	0,28284271	0,20506097	0,236431808	0,20407122	0,301593718	0,463100452	27	20	20	31	39	68	3	14	14	810,17
MS_run03	gi|23502937|ref|NP_699064.1|	pckA gene product [Brucella su	2,08	1,36	1,60	1,63	0,77	1,02				0,62561095	0,823781838	0,25237208	0,13316656	0,539405537	0,10226345	0,50521898	0,78138646	16	17	33	16	61	57	4	13	13	798,47
MS_run01	gi|23502938|ref|NP_699065.1|	unnamed protein product [Bru	0,88	0,85	0,88	0,85	1,00	0,96	0,89	9,26%	1,49E-03	1,13636364	1,12195122	0,05567764	0,11313708	0,035355339	0,06945066	0,280087883	0,060633552	6	11	4	6	25	7	3	7	7	388,7
MS_run02	gi|23502938|ref|NP_699065.1|	unnamed protein product [Bru	0,91	0,91	1,05	1,06	1,16	1,01				0,95272661	1,105757878	0,02828427	0,06363961	0,212132034	0,02566398	0,161251941	0,133299184	3	6	20	3	15	15	2	7	7	405,25
MS_run03	gi|23502938|ref|NP_699065.1|	unnamed protein product [Bru	0,77	0,73	0,90	0,94	1,17	1,04				1,15186615	1,367560574	0,21832697	0,21794495	0,321597575	0,26445221	0,287197472	0,069207807	24	19	34	23	21	10	4	8	8	464,58
MS_run01	gi|23502953|ref|NP_699080.1|	trx-1 gene product [Brucella su	0,89	0,42	0,79	0,37	0,89	0,47	0,73	18,85%	3,19E-02	1,26582278	2,405405405	0	0	0	0	0	0	0	0	0	0	0	0	1	2	2	119,46
MS_run02	gi|23502953|ref|NP_699080.1|	trx-1 gene product [Brucella su	0,53	0,85	0,61	0,83	1,16	1,35				1,63934426	0,952054795	0,13051181	0,27856777	0,504975247	0,34227287	0,329351933	0,331509099	21	24	61	21	35	39	4	4	4	196,44
MS_run03	gi|23502953|ref|NP_699080.1|	trx-1 gene product [Brucella su	0,92	0,90	0,83	0,84	0,90	1,01				1,12359551	1,106481481	0,28663275	0,06244998	0,36373067	0,21175674	0,170896784	0,308093538	35	7	43	19	15	34	4	4	4	221,57
MS_run01	gi|23502955|ref|NP_699082.1|	accD gene product [Brucella su	0,83	0,97	0,99	1,16	1,20	1,17	0,95	26,44%	3,93E-02	1,01010101	1,034482759	0	0	0	0	0	0	0	0	0	0	0	0	1	4	4	245,82
MS_run02	gi|23502955|ref|NP_699082.1|	accD gene product [Brucella su	0,45	0,40	1,19	1,08	2,67	0,91				0,84033613	2,472222222	0	0	0	0	0	0	0	0	0	0	0	0	1	3	3	164,19
MS_run03	gi|23502955|ref|NP_699082.1|	accD gene product [Brucella su	0,99	0,93	1,26	1,18	1,27	0,94				0,79365079	1,076271186	0	0	0	0	0	0	0	0	0	0	0	0	1	4	4	232,14
MS_run01	gi|23502962|ref|NP_699089.1|	unnamed protein product [Bru	1,64	1,47	1,56	1,40	0,95	0,89	2,18	67,86%	2,07E-04	0,66918325	0,680476877	0,45254834	0,08485281	0,091923882	0,19412678	0,015986192	0,034533305	29	9	7	29	2	2	2	3	3	188,22
MS_run02	gi|23502962|ref|NP_699089.1|	unnamed protein product [Bru	3,04	1,83	3,31	2,00	1,09	0,60				0,3021148	0,545	0	0	0	0	0	0	0	0	0	0	0	0	1	2	2	148,39
MS_run03	gi|23502962|ref|NP_699089.1|	unnamed protein product [Bru	3,11	2,00	2,94	1,85	0,95	0,63				0,3407244	0,542318982	0,0212132	0,09192388	0,551543289	0,00246264	0,211370961	0,777753813	1	10	30	1	39	39	2	3	3	172,54
MS_run02	gi|23502965|ref|NP_699092.1|		0,79	0,66	1,20	0,99	1,51	0,83	0,77	20,98%	8,23E-02	0,83333333	1,525252525	0	0	0	0	0	0	0	0	0	0	0	0	1	1	1	28,45
MS_run03	gi|23502965|ref|NP_699092.1|	infC gene product [Brucella sui	0,73	0,72	0,55	0,54	0,75	0,98				1,81818182	1,388888889	0	0	0	0	0	0	0	0	0	0	0	0	1	1	1	28,65
MS_run01	gi|23502967|ref|NP_699094.1|	rpmI gene product [Brucella su	0,57	0,66	0,74	0,85	1,29	1,15	0,76	13,42%	1,10E-03	1,35135135	1,517647059	0	0	0	0	0	0	0	0	0	0	0	0	1	1	1	42,27
MS_run02	gi|23502967|ref|NP_699094.1|	rpmI gene product [Brucella su	1,03	0,85	0,91	0,75	0,88	0,82				1,0989011	1,173333333	0	0	0	0	0	0	0	0	0	0	0	0	1	1	1	48,08
MS_run03	gi|23502967|ref|NP_699094.1|	rpmI gene product [Brucella su	0,62	0,82	0,58	0,77	0,94	1,33				1,72413793	1,220779221	0	0	0	0	0	0	0	0	0	0	0	0	1	1	1	45,58
MS_run01	gi|23502968|ref|NP_699095.1|	rplT gene product [Brucella sui	0,88	0,84	0,88	0,87	1,00	0,99	0,84	10,90%	8,35E-03	1,1365104	1,166666667	0,08848729	0,18062853	0,143422453	0,09912612	0,315230373	0,325067482	10	18	16	9	27	39	7	8	8	532,25
MS_run02	gi|23502968|ref|NP_699095.1|	rplT gene product [Brucella sui	1,02	0,91	0,96	0,92	0,94	0,96				1,04575163	1,096774194	0,08539126	0,1183216	0,104243305	0,09115887	0,194264177	0,155425533	9	13	11	9	18	17	5	7	7	331,36
MS_run03	gi|23502968|ref|NP_699095.1|	rplT gene product [Brucella sui	0,74	0,63	0,75	0,70	1,01	0,93				1,33357041	1,433870968	0,09591663	0,18953577	0,154148969	0,14606669	0,391632992	0,167802754	13	19	22	11	27	26	7	8	8	420,36
MS_run01	gi|23502970|ref|NP_699097.1|	pheS gene product [Brucella su	1,09	0,79	1,11	0,81	1,02	0,73	0,88	13,20%	1,62E-01	0,9009009	1,259259259	0	0	0	0	0	0	0	0	0	0	0	0	1	3	3	109,53
MS_run02	gi|23502970|ref|NP_699097.1|	pheS gene product [Brucella su	0,88	0,87	0,98	0,98	1,12	1,00				1,02040816	1,142857143	0	0	0	0	0	0	0	0	0	0	0	0	1	3	3	132,44
MS_run03	gi|23502970|ref|NP_699097.1|	pheS gene product [Brucella su	0,83	0,67	0,87	0,70	1,05	0,80				1,14942529	1,5	0	0	0	0	0	0	0	0	0	0	0	0	1	4	4	181,56
MS_run03	gi|23502971|ref|NP_699098.1|	pheT gene product [Brucella su	1,97	0,59	2,47	0,83	1,25	0,34	1,46	77,84%	2,05E-01	7,2457378	4,884199134	3,38704148	0,26870058	1,004091629	9,95603022	5,584919144	0,676161594	137	21	121	137	114	114	2	2	2	78,73
MS_run01	gi|23502973|ref|NP_699100.1|	dnaK gene product [Brucella su	0,94	0,93	0,94	0,88	0,99	0,94	0,88	3,63%	6,09E-06	1,0695493	1,077477477	0,12964025	0,10013627	0,141467971	0,17240802	0,189569301	0,142683531	14	10	16	16	18	15	16	19	19	1970,22
MS_run02	gi|23502973|ref|NP_699100.1|	dnaK gene product [Brucella su	0,85	0,90	0,87	0,86	1,03	0,99				1,14957717	1,115789474	0,12386821	0,16927624	0,166074589	0,19638724	0,149390062	0,106893851	14	17	19	17	13	12	13	19	19	1395,35
MS_run03	gi|23502973|ref|NP_699100.1|	dnaK gene product [Brucella su	0,84	0,83	0,87	0,86	1,03	0,98				1,17647059	1,206521739	0,11748509	0,18300522	0,16018609	0,21419509	0,255896424	0,151603453	14	18	19	18	21	18	18	20	20	1774,66
MS_run02	gi|23502990|ref|NP_699117.1|	phoU gene product [Brucella s	1,21	1,06	1,23	1,08	1,02	0,88	1,14	7,51%	3,38E-02	0,81300813	0,944444444	0	0	0	0	0	0	0	0	0	0	0	0	1	4	4	207,25
MS_run03	gi|23502996|ref|NP_699123.1|	pepS gene product [Brucella su	0,50	0,82	0,46	0,75	0,92	1,63	0,63	15,37%	3,25E-02	2,17391304	1,226666667	0	0	0	0	0	0	0	0	0	0	0	0	1	4	4	150,69
MS_run01	gi|23502997|ref|NP_699124.1|	unnamed protein product [Bru	0,91	0,93	0,82	0,84	0,91	1,02	0,82	6,82%	2,25E-03	1,2195122	1,078651685	0,05674504	0,17566066	0,159314006	0,07893147	0,178116145	0,142176411	7	19	19	6	17	15	7	8	8	768,14
MS_run02	gi|23502997|ref|NP_699124.1|	unnamed protein product [Bru	0,86	0,76	0,79	0,71	0,92	0,90				1,26907631	1,321777003	0,10144785	0,18700267	0,153850577	0,18359605	0,074343785	0,039647767	13	20	22	14	6	5	5	7	7	572,4
MS_run03	gi|23502997|ref|NP_699124.1|	unnamed protein product [Bru	0,88	0,76	0,83	0,72	0,94	0,87				1,20481928	1,323943662	0,05787918	0,18986838	0,063482281	0,07741248	0,154374429	0,096556882	7	20	9	6	12	13	5	7	7	527,05
MS_run01	gi|23503007|ref|NP_699134.1|	unnamed protein product [Bru	0,90	0,95	1,11	1,18	1,24	1,06	1,02	15,30%	3,95E-02	0,9009009	1,050847458	0	0	0	0	0	0	0	0	0	0	0	0	1	3	3	241,23
MS_run03	gi|23503007|ref|NP_699134.1|	unnamed protein product [Bru	0,79	0,90	1,10	1,26	1,40	1,15				0,90909091	1,111111111	0	0	0	0	0	0	0	0	0	0	0	0	1	3	3	191,33
MS_run01	gi|23503008|ref|NP_699135.1|	metK gene product [Brucella su	1,55	1,58	1,55	1,58	1,00	1,02	1,86	46,57%	8,21E-03	0,64516129	0,632911392	0	0	0	0	0	0	0	0	0	0	0	0	1	5	5	251,94
MS_run03	gi|23503008|ref|NP_699135.1|	metK gene product [Brucella su	1,95	1,51	2,91	2,25	1,49	0,77				0,34364261	0,662222222	0	0	0	0	0	0	0	0	0	0	0	0	1	3	3	177,78

MS_run01	gi|23503011|ref|NP_699138.1|	nusA gene product [Brucella su	0,94	0,82	0,92	0,74	0,97	0,81	0,93	9,20%	1,71E-01	1,09371265	1,220779221	0,09612492	0,20007499	0,114309521	0,10668716	0,246119269	0,133801386	11	21	15	10	20	16	8	12	12	694,85
MS_run02	gi|23503011|ref|NP_699138.1|	nusA gene product [Brucella su	0,89	1,08	1,02	1,04	1,14	1,02				0,98039216	0,949415205	0,13740451	0,23252957	0,165770926	0,14844724	0,164901788	0,187299711	13	20	16	15	17	17	6	12	12	634,17
MS_run03	gi|23503011|ref|NP_699138.1|	nusA gene product [Brucella su	0,98	0,85	0,95	0,92	0,97	0,96				1,05263158	1,193478261	0,19748418	0,17637082	0,114847725	0,2059425	0,223268707	0,154458756	21	18	13	20	19	18	7	14	14	674,7
MS_run01	gi|23503013|ref|NP_699140.1|	infB gene product [Brucella sui	0,86	0,84	1,07	0,96	1,24	0,90	1,27	52,61%	3,09E-01	1,0538757	1,204059829	0,50911688	1,22329473	0,848528137	0,50144478	0,210016758	0,147100787	48	98	88	48	17	17	2	7	7	313,11
MS_run02	gi|23503013|ref|NP_699140.1|	infB gene product [Brucella sui	0,44	0,50	1,50	1,70	3,39	1,13				0,66666667	1,994117647	0	0	0	0	0	0	0	0	0	0	0	0	1	8	8	367,14
MS_run03	gi|23503013|ref|NP_699140.1|	infB gene product [Brucella sui	1,82	2,01	1,66	1,83	0,91	1,10				0,60240964	0,49726776	0	0	0	0	0	0	0	0	0	0	0	0	1	7	7	345,37
MS_run01	gi|23503015|ref|NP_699142.1|	rpsO gene product [Brucella su	0,71	0,67	0,84	0,81	1,19	0,96	0,80	17,50%	2,19E-02	1,11111111	1,49382716	0,08485281	0,17349352	0,036055513	0,66521931	0,259697461	0,155598236	10	15	4	60	17	23	3	4	4	219,76
MS_run02	gi|23503015|ref|NP_699142.1|	rpsO gene product [Brucella su	1,21	1,00	0,96	0,89	0,80	0,92				0,91145833	0,751879699	0,24440404	0,34278273	0,049497475	0,52314545	0,414363861	0,607602232	25	43	6	57	55	61	4	5	5	288,77
MS_run03	gi|23503015|ref|NP_699142.1|	rpsO gene product [Brucella su	0,62	0,63	0,66	0,66	1,06	1,00				1,55075188	1,597898869	0,14764823	0,11165423	0,084063468	0,37471869	0,063112143	0,023065537	22	11	13	24	4	4	4	5	5	300,71
MS_run01	gi|23503016|ref|NP_699143.1|	pnp gene product [Brucella sui	1,11	1,05	1,12	1,04	1,01	0,93	1,20	19,75%	7,06E-03	0,89285714	0,948065713	0,16155494	0,11187294	0,230074867	0,12206413	0,103249947	0,127067201	14	11	22	14	11	12	10	17	17	676,33
MS_run02	gi|23503016|ref|NP_699143.1|	pnp gene product [Brucella sui	1,07	1,03	1,09	1,02	1,02	0,94				0,92183517	0,968253968	0,11880356	0,12980755	0,136982793	0,10468391	0,170700415	0,233147961	11	13	13	11	18	23	10	17	17	704,75
MS_run03	gi|23503016|ref|NP_699143.1|	pnp gene product [Brucella sui	1,53	1,51	1,48	1,39	0,97	0,94				0,67567568	0,662068966	0,27838822	0,18583787	0,352881	0,14267789	0,115798578	0,246762621	19	19	25	21	17	16	12	18	18	829,35
MS_run03	gi|23503018|ref|NP_699145.1|	fabI-2 gene product [Brucella s	0,63	0,48	0,96	0,75	1,53	0,78	0,70	17,51%	8,82E-02	1,14626866	2,184983466	0,41012193	0,15556349	0,311126984	0,48969783	0,698991765	0,154307655	43	10	41	43	32	32	2	8	8	414,82
MS_run01	gi|23503020|ref|NP_699147.1|	fabA gene product [Brucella su	0,91	0,89	1,00	0,98	1,10	0,98	1,06	15,68%	1,44E-01	1	1,12244898	0	0	0	0	0	0	0	0	0	0	0	0	1	3	3	157,93
MS_run02	gi|23503020|ref|NP_699147.1|	fabA gene product [Brucella su	1,38	1,41	1,02	1,04	0,74	1,02				0,98039216	0,711538462	0	0	0	0	0	0	0	0	0	0	0	0	1	3	3	187,11
MS_run03	gi|23503020|ref|NP_699147.1|	fabA gene product [Brucella su	1,02	0,97	1,10	1,05	1,08	0,95				0,90909091	1,028571429	0	0	0	0	0	0	0	0	0	0	0	0	1	3	3	167,29
MS_run01	gi|23503022|ref|NP_699149.1|	unnamed protein product [Bru	0,93	0,99	1,11	1,17	1,20	1,05	1,05	9,60%	2,31E-01	0,90207233	1,020131531	0,05656854	0,14849242	0,028284271	0,045972	1,02E-01	0,098755421	5	12	2	5	10	10	2	5	5	187,66
MS_run01	gi|23503028|ref|NP_699155.1|	unnamed protein product [Bru	2,10	1,38	2,32	1,53	1,11	0,66	1,22	52,27%	1,27E-01	0,43959591	0,722173932	0,43133514	0,20506097	0,282842712	0,08190633	5,22E-04	0,001001735	19	19	18	19	0	0	2	7	7	349,92
MS_run02	gi|23503028|ref|NP_699155.1|	unnamed protein product [Bru	1,15	1,25	1,02	1,11	0,89	1,09				0,98039216	0,801801802	0	0	0	0	0	0	0	0	0	0	0	0	1	6	6	198,05
MS_run03	gi|23503028|ref|NP_699155.1|	unnamed protein product [Bru	0,72	0,67	0,71	0,66	0,99	0,93				1,4084507	1,5	0	0	0	0	0	0	0	0	0	0	0	0	1	5	5	165
MS_run01	gi|23503031|ref|NP_699158.1|	unnamed protein product [Bru	1,49	2,42	1,46	2,33	0,98	1,59	1,79	45,93%	6,67E-02	1,04885057	0,754626623	1,21622366	0,04242641	2,156675683	0,8737239	7,18E-01	2,305558692	83	4	93	83	95	95	2	5	5	161,3
MS_run03	gi|23503031|ref|NP_699158.1|	unnamed protein product [Bru	1,19	2,01	1,27	2,14	1,07	1,69				0,8604336	0,539521	0,52325902	0,01414214	0,806101731	0,35451153	0,209836891	0,779868118	41	1	38	41	39	39	2	5	5	204,72
MS_run01	gi|23503032|ref|NP_699159.1|	rpsT gene product [Brucella sui	0,80	0,72	0,79	0,79	1,00	0,99	0,79	10,26%	7,51E-05	1,27240717	1,383007288	0,18518009	0,06130525	0,088881944	0,32967648	1,80E-01	0,100722406	23	6	11	26	13	14	4	4	4	146,62
MS_run02	gi|23503032|ref|NP_699159.1|	rpsT gene product [Brucella sui	0,84	0,98	0,85	0,97	1,01	1,14				1,12015019	1,020618557	0,42099089	0,09865766	0,120968315	0,07964907	0,045536015	0,040710286	50	10	12	7	4	4	3	3	3	106,27
MS_run03	gi|23503032|ref|NP_699159.1|	rpsT gene product [Brucella sui	0,68	0,67	0,70	0,68	1,04	0,97				1,42886303	1,489135206	0,04031129	0,04272002	0,118462371	0,07650957	0,139555936	0,07370741	6	4	17	5	9	11	4	4	4	153,58
