# Supplementary material for: RegA Plays a Key Role in Oxygen-Dependent Establishment of Persistence and in Isocitrate Lyase Activity, a Critical Determinant of In vivo Brucella suis Pathogenicity
Source: Front Cell Infect Microbiol. 2017 May 18;7:186. doi: 10.3389/fcimb.2017.00186 (PMC5435760; doi:10.3389/fcimb.2017.00186)
Supplement: Supplementary file 7 [file Table7.PDF]

S7 Table: Peptide Mass Fingerprinting results of RegA-controlled proteins identified by 2D-DIGE.
The database queries are included.

TOPLAB Gesellschaft für angewandte Biotechnologie mbH

AlDahouk_6214

Analysis Information

**Report Type**	Protein-Peptide Summary by Spot	**Analysis Type**	Combined (MS+MS/MS)
**Sample Set Name**	Div_120507	**Database**	Bruc_suis_DEC2011
**Analysis Name**	AlDahouk_6214	**Creation Date**	05/30/2012 10:00:47
**Reported By**	08/17/2012 17:57:09 - admin	**Last Modified**	05/30/2012 13:29:11

**MS Acq. : Proc. Methods**	(Unspecified) : (Unspecified)
**Interpretation Method**	(Unspecified)

**Gel Idx/Pos**	200/CAL 8	**Instr./Gel Origin**	AK043/Div_120507	**Process Status**	Analysis Succeeded
**Plate [#] Name**	[1] 1300017700	**Instrument Sample Name**		**Spectra**	12

Rank	Protein Name	Accession No.	Pep. Count	Protein Score	Protein Score C. I. %	Total Ion Score	Best Ion Score	Best Ion C. I. %	Total Ion C. I. %	Confirmed	Sample Name	Customer sample Name

No Confirmed Protein Found

**Gel Idx/Pos**	176/H3	**Instr./Gel Origin**	AK043/Div_120507	**Process Status**	Analysis Succeeded
**Plate [#] Name**	[1] 1300017700	**Instrument Sample Name**		**Spectra**	4

Rank	Protein Name	Accession No.	Pep. Count	Protein Score	Protein Score C. I. %	Total Ion Score	Best Ion Score	Best Ion C. I. %	Total Ion C. I. %	Confirmed	Sample Name	Customer sample Name
1	hypothetical protein BSUIS_A1239 [Brucella suis ATCC 23445]	gi|163843456|ref|YP_001627860.1|	10	171	100	87	87	100	100	.T.	AlDahouk_6214_Z19769-1_T1	1499
2	unnamed protein product [Brucella suis 1330]	gi|23502068|ref|NP_698195.1|	8	143	100	87	87	100	100	.T.	AlDahouk_6214_Z19769-1_T1	1499

**Gel Idx/Pos**	177/H4	**Instr./Gel Origin**	AK043/Div_120507	**Process Status**	Analysis Succeeded
**Plate [#] Name**	[1] 1300017700	**Instrument Sample Name**		**Spectra**	4

Rank	Protein Name	Accession No.	Pep. Count	Protein Score	Protein Score C. I. %	Total Ion Score	Best Ion Score	Best Ion C. I. %	Total Ion C. I. %	Confirmed	Sample Name	Customer sample Name
1	bacterioferritin [Brucella suis ATCC 23445]	gi|163844717|ref|YP_001622372.1|	6	161	100	127	83	100	100	.T.	AlDahouk_6214_Z19769-3_T1	1467

Gel Idx/Pos	178/H5	Instr./Gel Origin	AK043/Div_120507	Process Status	Analysis Succeeded
Plate [#] Name	[1] 1300017700	Instrument Sample Name		Spectra	4

Rank	Protein	Name	Accession No.	Pep. Count	Protein Score	Protein Score C. I. %	Total Ion Score	Best Ion Score	Best Ion C. I. %	Total Ion C. I. %	Confirmed	Sample Name	Customer sample Name
1	bacterioferritin [Brucella suis ATCC 23445]		gi|163844717|ref|YP_001622372.1|	14	459	100	336	134	100	100	.T.	AlDahouk_6214_Z19769-5_T1	2081

Gel Idx/Pos	179/H6	Instr./Gel Origin	AK043/Div_120507	Process Status	Analysis Succeeded
Plate [#] Name	[1] 1300017700	Instrument Sample Name		Spectra	4

Rank	Protein	Name	Accession No.	Pep. Count	Protein Score	Protein Score C. I. %	Total Ion Score	Best Ion Score	Best Ion C. I. %	Total Ion C. I. %	Confirmed	Sample Name	Customer sample Name
1	grxC gene product [Brucella suis 1330]		gi|23502729|ref|NP_698856.1|	8	384	100	299	146	100	100	.T.	AlDahouk_6214_Z19769-6_T1	1983

Gel Idx/Pos	180/H7	Instr./Gel Origin	AK043/Div_120507	Process Status	Analysis Succeeded
Plate [#] Name	[1] 1300017700	Instrument Sample Name		Spectra	4

Rank	Protein	Name	Accession No.	Pep. Count	Protein Score	Protein Score C. I. %	Total Ion Score	Best Ion Score	Best Ion C. I. %	Total Ion C. I. %	Confirmed	Sample Name	Customer sample Name
1	etfB gene product [Brucella suis 1330]		gi|23502819|ref|NP_698946.1|	22	582	100	387	199	100	100	.T.	AlDahouk_6214_Z19769-7_T1	1082
2	unnamed protein product [Brucella suis 1330]		gi|23500019|ref|NP_699459.1|	16	109	100					.T.	AlDahouk_6214_Z19769-7_T1	1082
3	hypothetical protein BSUIS_B0267 [Brucella suis ATCC 23445]		gi|163844446|ref|YP_001622101.1|	15	93	100					.T.	AlDahouk_6214_Z19769-7_T1	1082

Gel Idx/Pos	181/H8	Instr./Gel Origin	AK043/Div_120507	Process Status	Analysis Succeeded
Plate [#] Name	[1] 1300017700	Instrument Sample Name		Spectra	4

Rank	Protein	Name	Accession No.	Pep. Count	Protein Score	Protein Score C. I. %	Total Ion Score	Best Ion Score	Best Ion C. I. %	Total Ion C. I. %	Confirmed	Sample Name	Customer sample Name
1	unnamed protein product [Brucella suis 1330]		gi|23501191|ref|NP_697318.1|	9	189	100	121	121	100	100	.T.	AlDahouk_6214_Z19769-8_T1	1731
2	unnamed protein product [Brucella suis 1330]		gi|23502043|ref|NP_698170.1|	6	138	100	96	96	100	100	.T.	AlDahouk_6214_Z19769-8_T1	1731
3	endoribonuclease L-PSP [Brucella suis ATCC 23445]		gi|163843429|ref|Y	5	130	100	96	96	100	100	.T.	AlDahouk_6	1731

Rank	Protein Name	Accession No.	Pep. Count	Protein Score	Protein Score C. I. %	Total Ion Score	Best Ion Score	Best Ion C. I. %	Total Ion C. I. %	Confirmed	Sample Name	Customer sample Name
		P_001627833.1|									214_Z19769-8_T1	
4	homoprotocatechuate degradation operon regulator, HpaR [Brucella suis ATCC 23445]	gi|163845479|ref|YP_001623134.1|	9	103	100	60	60	100	100	.T.	AlDahouk_6214_Z19769-8_T1	1731
5	dut gene product [Brucella suis 1330]	gi|23502533|ref|NP_698660.1|	8	60	98.572					.T.	AlDahouk_6214_Z19769-8_T1	1731

**Gel Idx/Pos**	182/H9	**Instr./Gel Origin**	AK043/Div_120507	**Process Status**	Analysis Succeeded
**Plate [#] Name**	[1] 1300017700	**Instrument Sample Name**		**Spectra**	4

Rank	Protein Name	Accession No.	Pep. Count	Protein Score	Protein Score C. I. %	Total Ion Score	Best Ion Score	Best Ion C. I. %	Total Ion C. I. %	Confirmed	Sample Name	Customer sample Name
1	50S ribosomal protein L33 [Brucella suis ATCC 23445]	gi|163844761|ref|YP_001622416.1|	6	222	100	182	125	100	100	.T.	AlDahouk_6214_Z19769-9_T1	1933

**Gel Idx/Pos**	183/H10	**Instr./Gel Origin**	AK043/Div_120507	**Process Status**	Analysis Succeeded
**Plate [#] Name**	[1] 1300017700	**Instrument Sample Name**		**Spectra**	4

Rank	Protein Name	Accession No.	Pep. Count	Protein Score	Protein Score C. I. %	Total Ion Score	Best Ion Score	Best Ion C. I. %	Total Ion C. I. %	Confirmed	Sample Name	Customer sample Name
1	chaperonin GroEL [Brucella suis ATCC 23445]	gi|163844383|ref|YP_001622038.1|	21	262	100	148	84	100	100	.T.	AlDahouk_6214_Z19769-10_T1	384
2	katA gene product [Brucella suis 1330]	gi|23500108|ref|NP_699548.1|	14	68	99.768					.T.	AlDahouk_6214_Z19769-10_T1	384
3	hypothetical protein BSUIS_B0360 [Brucella suis ATCC 23445]	gi|163844531|ref|YP_001622186.1|	13	60	98.667					.T.	AlDahouk_6214_Z19769-10_T1	384

**Gel Idx/Pos**	184/H11	**Instr./Gel Origin**	AK043/Div_120507	**Process Status**	Analysis Succeeded
**Plate [#] Name**	[1] 1300017700	**Instrument Sample Name**		**Spectra**	4

Rank	Protein Name	Accession No.	Pep. Count	Protein Score	Protein Score C. I. %	Total Ion Score	Best Ion Score	Best Ion C. I. %	Total Ion C. I. %	Confirmed	Sample Name	Customer sample Name
1	pyk gene product [Brucella suis 1330]	gi|23502605|ref|NP_698732.1|	19	242	100	119	61	100	100	.T.	AlDahouk_6214_Z19769-11_T1	389
2	pyruvate kinase [Brucella suis ATCC 23445]	gi|163845326|ref|YP_001622981.1|	17	220	100	119	61	100	100	.T.	AlDahouk_6214_Z19769-11_T1	389
3	chaperonin GroEL [Brucella suis ATCC 23445]	gi|163844383|ref|YP_001622038.1|	16	122	100	64	64	100	100	.T.	AlDahouk_6214_Z19769-11_T1	389

Gel Idx/Pos	185/H12	Instr./Gel Origin	AK043/Div_120507	Process Status	Analysis Succeeded
Plate [#] Name	[1] 1300017700	Instrument Sample Name		Spectra	4

Rank	Protein	Name	Accession No.	Pep. Count	Protein Score	Protein Score C. I. %	Total Ion Score	Best Ion Score	Best Ion C. I. %	Total Ion C. I. %	Confirmed	Sample Name	Customer sample Name
1	pckA gene product [Brucella suis 1330]		gi|23502937|ref|NP_699064.1|	34	573	100	279	110	100	100	.T.	AlDahouk_6214_Z19769-12_T1	2062
2	chaperonin GroEL [Brucella suis ATCC 23445]		gi|163844383|ref|YP_001622038.1|	20	107	100					.T.	AlDahouk_6214_Z19769-12_T1	2062
3	unnamed protein product [Brucella suis 1330]		gi|23501035|ref|NP_697162.1|	13	71	99.889					.T.	AlDahouk_6214_Z19769-12_T1	2062

Gel Idx/Pos	186/H13	Instr./Gel Origin	AK043/Div_120507	Process Status	Analysis Succeeded
Plate [#] Name	[1] 1300017700	Instrument Sample Name		Spectra	4

Rank	Protein	Name	Accession No.	Pep. Count	Protein Score	Protein Score C. I. %	Total Ion Score	Best Ion Score	Best Ion C. I. %	Total Ion C. I. %	Confirmed	Sample Name	Customer sample Name
1	trx-1 gene product [Brucella suis 1330]		gi|23502953|ref|NP_699080.1|	3	95	100	77	77	100	100	.T.	AlDahouk_6214_Z19769-13_T1	2082

Gel Idx/Pos	187/H14	Instr./Gel Origin	AK043/Div_120507	Process Status	Analysis Succeeded
Plate [#] Name	[1] 1300017700	Instrument Sample Name		Spectra	4

Rank	Protein	Name	Accession No.	Pep. Count	Protein Score	Protein Score C. I. %	Total Ion Score	Best Ion Score	Best Ion C. I. %	Total Ion C. I. %	Confirmed	Sample Name	Customer sample Name
1	trx-1 gene product [Brucella suis 1330]		gi|23502953|ref|NP_699080.1|	3	69	99.84	51	51	99.996	99.996	.T.	AlDahouk_6214_Z19769-14_T1	1936
2	aspartyl/glutamyl-tRNA amidotransferase subunit C [Brucella suis ATCC 23445]		gi|163844747|ref|YP_001622402.1|	4	52	92.505	26	26	98.32	98.32	.T.	AlDahouk_6214_Z19769-14_T1	1936

Gel Idx/Pos	188/H15	Instr./Gel Origin	AK043/Div_120507	Process Status	Analysis Succeeded
Plate [#] Name	[1] 1300017700	Instrument Sample Name		Spectra	4

Rank	Protein	Name	Accession No.	Pep. Count	Protein Score	Protein Score C. I. %	Total Ion Score	Best Ion Score	Best Ion C. I. %	Total Ion C. I. %	Confirmed	Sample Name	Customer sample Name
1	unnamed protein product [Brucella suis 1330]		gi|23501035|ref|NP_697162.1|	23	277	100	100	100	100	100	.T.	AlDahouk_6214_Z19769-15_T1	418

Rank	Protein Name	Accession No.	Pep. Count	Protein Score	Protein Score C. I. %	Total Ion Score	Best Ion Score	Best Ion C. I. %	Total Ion C. I. %	Confirmed	Sample Name	Customer sample Name
2	chaperonin GroEL [Brucella suis ATCC 23445]	gi|163844383|ref|YP_001622038.1|	23	273	100	134	134	100	100	.T.	AlDahouk_6214_Z19769-15_T1	418

Gel Idx/Pos	189/H16	Instr./Gel Origin	AK043/Div_120507	Process Status	Analysis Succeeded
Plate [#] Name	[1] 1300017700	Instrument Sample Name		Spectra	4

Rank	Protein Name	Accession No.	Pep. Count	Protein Score	Protein Score C. I. %	Total Ion Score	Best Ion Score	Best Ion C. I. %	Total Ion C. I. %	Confirmed	Sample Name	Customer sample Name
1	pckA gene product [Brucella suis 1330]	gi|23502937|ref|NP_699064.1|	22	395	100	249	97	100	100	.T.	AlDahouk_6214_Z19769-16_T1	374
2	chaperonin GroEL [Brucella suis ATCC 23445]	gi|163844383|ref|YP_001622038.1|	19	84	99.995					.T.	AlDahouk_6214_Z19769-16_T1	374
3	unnamed protein product [Brucella suis 1330]	gi|23501913|ref|NP_698040.1|	16	75	99.955					.T.	AlDahouk_6214_Z19769-16_T1	374

Gel Idx/Pos	190/H17	Instr./Gel Origin	AK043/Div_120507	Process Status	Analysis Succeeded
Plate [#] Name	[1] 1300017700	Instrument Sample Name		Spectra	4

Rank	Protein Name	Accession No.	Pep. Count	Protein Score	Protein Score C. I. %	Total Ion Score	Best Ion Score	Best Ion C. I. %	Total Ion C. I. %	Confirmed	Sample Name	Customer sample Name
1	chaperonin GroEL [Brucella suis ATCC 23445]	gi|163844383|ref|YP_001622038.1|	24	433	100	279	153	100	100	.T.	AlDahouk_6214_Z19769-17_T1	380
2	unnamed protein product [Brucella suis 1330]	gi|23501576|ref|NP_697703.1|	17	216	100	115	115	100	100	.T.	AlDahouk_6214_Z19769-17_T1	380
3	pckA gene product [Brucella suis 1330]	gi|23502937|ref|NP_699064.1|	17	92	100					.T.	AlDahouk_6214_Z19769-17_T1	380
4	unnamed protein product [Brucella suis 1330]	gi|23501035|ref|NP_697162.1|	12	58	97.684					.T.	AlDahouk_6214_Z19769-17_T1	380

Gel Idx/Pos	191/H18	Instr./Gel Origin	AK043/Div_120507	Process Status	Analysis Succeeded
Plate [#] Name	[1] 1300017700	Instrument Sample Name		Spectra	4

Rank	Protein Name	Accession No.	Pep. Count	Protein Score	Protein Score C. I. %	Total Ion Score	Best Ion Score	Best Ion C. I. %	Total Ion C. I. %	Confirmed	Sample Name	Customer sample Name
1	pckA gene product [Brucella suis 1330]	gi|23502937|ref|NP_699064.1|	26	386	100	196	106	100	100	.T.	AlDahouk_6214_Z19769-18_T1	426
2	chaperonin GroEL [Brucella suis ATCC 23445]	gi|163844383|ref|YP_001622038.1|	20	234	100	129	129	100	100	.T.	AlDahouk_6214_Z19769-	426

3	unnamed protein product [Brucella suis 1330]	gi|23501035|ref|NP_697162.1|	14	75	99.959					.T.	AlDahouk_6214_Z19769-18_T1	426

Gel Idx/Pos	192/H19	Instr./Gel Origin	AK043/Div_120507	Process Status	Analysis Succeeded
Plate [#] Name	[1] 1300017700	Instrument Sample Name		Spectra	4

Rank	Protein Name	Accession No.	Pep. Count	Protein Score	Protein Score C. I. %	Total Ion Score	Best Ion Score	Best Ion C. I. %	Total Ion C. I. %	Confirmed	Sample Name	Customer sample Name
1	unnamed protein product [Brucella suis 1330]	gi|23501748|ref|NP_697875.1|	12	169	100	78	44	99.972	100	.T.	AlDahouk_6214_Z19769-19_T1	1105
2	unnamed protein product [Brucella suis 1330]	gi|23502071|ref|NP_698198.1|	13	143	100	64	64	100	100	.T.	AlDahouk_6214_Z19769-19_T1	1105
3	TRAP transporter solute receptor TAXI family protein [Brucella suis ATCC 23445]	gi|163843459|ref|YP_001627863.1|	12	133	100	64	64	100	100	.T.	AlDahouk_6214_Z19769-19_T1	1105
4	rplC gene product [Brucella suis 1330]	gi|23502110|ref|NP_698237.1|	11	59	98.505					.T.	AlDahouk_6214_Z19769-19_T1	1105
5	50S ribosomal protein L3 [Brucella suis ATCC 23445]	gi|163843499|ref|YP_001627903.1|	11	58	98.029					.T.	AlDahouk_6214_Z19769-19_T1	1105

Gel Idx/Pos	193/H20	Instr./Gel Origin	AK043/Div_120507	Process Status	Analysis Succeeded
Plate [#] Name	[1] 1300017700	Instrument Sample Name		Spectra	4

Rank	Protein Name	Accession No.	Pep. Count	Protein Score	Protein Score C. I. %	Total Ion Score	Best Ion Score	Best Ion C. I. %	Total Ion C. I. %	Confirmed	Sample Name	Customer sample Name
1	chaperonin GroEL [Brucella suis ATCC 23445]	gi|163844383|ref|YP_001622038.1|	24	392	100	255	99	100	100	.T.	AlDahouk_6214_Z19769-20_T1	421

Gel Idx/Pos	194/H21	Instr./Gel Origin	AK043/Div_120507	Process Status	Analysis Succeeded
Plate [#] Name	[1] 1300017700	Instrument Sample Name		Spectra	4

Rank	Protein Name	Accession No.	Pep. Count	Protein Score	Protein Score C. I. %	Total Ion Score	Best Ion Score	Best Ion C. I. %	Total Ion C. I. %	Confirmed	Sample Name	Customer sample Name
1	katA gene product [Brucella suis 1330]	gi|23500108|ref|NP_699548.1|	29	453	100	214	78	100	100	.T.	AlDahouk_6214_Z19769-21_T1	377
2	hypothetical protein BSUIS_B0360 [Brucella suis ATCC 23445]	gi|163844531|ref|YP_001622186.1|	28	441	100	214	78	100	100	.T.	AlDahouk_6214_Z19769-21_T1	377

Rank	Protein Name	Accession No.	Pep. Count	Protein Score	Protein Score C. I. %	Total Ion Score	Best Ion Score	Best Ion C. I. %	Total Ion C. I. %	Confirmed	Sample Name	Customer sample Name
3	chaperonin GroEL [Brucella suis ATCC 23445]	gi|163844383|ref|YP_001622038.1|	17	67	99.722					.T.	AlDahouk_6214_Z19769-21_T1	377

Gel Idx/Pos	195/H22	Instr./Gel Origin	AK043/Div_120507	Process Status	Analysis Succeeded
Plate [#] Name	[1] 1300017700	Instrument Sample Name		Spectra	4

Rank	Protein Name	Accession No.	Pep. Count	Protein Score	Protein Score C. I. %	Total Ion Score	Best Ion Score	Best Ion C. I. %	Total Ion C. I. %	Confirmed	Sample Name	Customer sample Name
1	chaperonin GroEL [Brucella suis ATCC 23445]	gi|163844383|ref|YP_001622038.1|	33	686	100	420	164	100	100	.T.	AlDahouk_6214_Z19769-22_T1	519
2	unnamed protein product [Brucella suis 1330]	gi|23500285|ref|NP_699725.1|	17	96	100					.T.	AlDahouk_6214_Z19769-22_T1	519
3	unnamed protein product [Brucella suis 1330]	gi|23501912|ref|NP_698039.1|	12	68	99.798					.T.	AlDahouk_6214_Z19769-22_T1	519
4	unnamed protein product [Brucella suis 1330]	gi|23501498|ref|NP_697625.1|	13	61	98.941					.T.	AlDahouk_6214_Z19769-22_T1	519
5	hypothetical protein BSUIS_B0534 [Brucella suis ATCC 23445]	gi|163844697|ref|YP_001622352.1|	13	60	98.839					.T.	AlDahouk_6214_Z19769-22_T1	519
6	glcD gene product [Brucella suis 1330]	gi|23499942|ref|NP_699382.1|	13	60	98.756					.T.	AlDahouk_6214_Z19769-22_T1	519
7	hypothetical protein BSUIS_A1077 [Brucella suis ATCC 23445]	gi|163843302|ref|YP_001627706.1|	11	59	98.47					.T.	AlDahouk_6214_Z19769-22_T1	519

Gel Idx/Pos	196/H23	Instr./Gel Origin	AK043/Div_120507	Process Status	Analysis Succeeded
Plate [#] Name	[1] 1300017700	Instrument Sample Name		Spectra	4

Rank	Protein Name	Accession No.	Pep. Count	Protein Score	Protein Score C. I. %	Total Ion Score	Best Ion Score	Best Ion C. I. %	Total Ion C. I. %	Confirmed	Sample Name	Customer sample Name
1	chaperonin GroEL [Brucella suis ATCC 23445]	gi|163844383|ref|YP_001622038.1|	37	762	100	440	158	100	100	.T.	AlDahouk_6214_Z19769-23_T1	417
2	Random sequence, was unnamed protein product [Brucella suis 1330]	###RND###gi|23501509|ref|NP_697636.1|	12	60	98.539					.T.	AlDahouk_6214_Z19769-23_T1	417

Gel Idx/Pos<br>Plate [#] Name	201/I1<br>[1] 1300017700	Instr./Gel Origin<br>Instrument Sample Name	AK043/Div_120507	Process Status<br>Spectra	Analysis Succeeded<br>4

Rank	Protein	Name	Accession No.	Pep. Count	Protein Score	Protein Score C. I. %	Total Ion Score	Best Ion Score	Best Ion C. I. %	Total Ion C. I. %	Confirmed	Sample Name	Customer sample Name
1	chaperonin GroEL [Brucella suis ATCC 23445]		gi|163844383|ref|YP_001622038.1|	28	588	100	390	137	100	100	.T.	AlDahouk_6214_Z19769-24_T1	435
2	pckA gene product [Brucella suis 1330]		gi|23502937|ref|NP_699064.1|	16	80	99.985					.T.	AlDahouk_6214_Z19769-24_T1	435

Gel Idx/Pos<br>Plate [#] Name	202/I2<br>[1] 1300017700	Instr./Gel Origin<br>Instrument Sample Name	AK043/Div_120507	Process Status<br>Spectra	Analysis Succeeded<br>4

Rank	Protein	Name	Accession No.	Pep. Count	Protein Score	Protein Score C. I. %	Total Ion Score	Best Ion Score	Best Ion C. I. %	Total Ion C. I. %	Confirmed	Sample Name	Customer sample Name
1	chaperonin GroEL [Brucella suis ATCC 23445]		gi|163844383|ref|YP_001622038.1|	35	695	100	397	140	100	100	.T.	AlDahouk_6214_Z19769-25_T1	425

Gel Idx/Pos<br>Plate [#] Name	203/I3<br>[1] 1300017700	Instr./Gel Origin<br>Instrument Sample Name	AK043/Div_120507	Process Status<br>Spectra	Analysis Succeeded<br>4

Rank	Protein	Name	Accession No.	Pep. Count	Protein Score	Protein Score C. I. %	Total Ion Score	Best Ion Score	Best Ion C. I. %	Total Ion C. I. %	Confirmed	Sample Name	Customer sample Name
1	chaperonin GroEL [Brucella suis ATCC 23445]		gi|163844383|ref|YP_001622038.1|	34	692	100	411	143	100	100	.T.	AlDahouk_6214_Z19769-26_T1	420
2	unnamed protein product [Brucella suis 1330]		gi|23501498|ref|NP_697625.1|	15	79	99.982					.T.	AlDahouk_6214_Z19769-26_T1	420
3	unnamed protein product [Brucella suis 1330]		gi|23501912|ref|NP_698039.1|	11	60	98.698					.T.	AlDahouk_6214_Z19769-26_T1	420

Gel Idx/Pos<br>Plate [#] Name	204/I4<br>[1] 1300017700	Instr./Gel Origin<br>Instrument Sample Name	AK043/Div_120507	Process Status<br>Spectra	Analysis Succeeded<br>4

Rank	Protein	Name	Accession No.	Pep. Count	Protein Score	Protein Score C. I. %	Total Ion Score	Best Ion Score	Best Ion C. I. %	Total Ion C. I. %	Confirmed	Sample Name	Customer sample Name
1	unnamed protein product [Brucella suis 1330]		gi|23500285|ref|NP_699725.1|	24	467	100	296	136	100	100	.T.	AlDahouk_6214_Z19769-27_T1	494
2	hypothetical protein BSUIS_B0534 [Brucella suis ATCC		gi|163844697|ref|Y	20	420	100	296	136	100	100	.T.	AlDahouk_6	494

Rank	Protein Name	Accession No.	Pep. Count	Protein Score	Protein Score C. I. %	Total Ion Score	Best Ion Score	Best Ion C. I. %	Total Ion C. I. %	Confirmed	Sample Name	Customer sample Name
	23445]	P_001622352.1|									214_Z19769-27_T1	
3	chaperonin GroEL [Brucella suis ATCC 23445]	gi|163844383|ref|YP_001622038.1|	25	151	100					.T.	AlDahouk_6214_Z19769-27_T1	494

**Gel Idx/Pos**	205/I5	**Instr./Gel Origin**	AK043/Div_120507	**Process Status**	Analysis Succeeded
**Plate [#] Name**	[1] 1300017700	**Instrument Sample Name**		**Spectra**	4

Rank	Protein Name	Accession No.	Pep. Count	Protein Score	Protein Score C. I. %	Total Ion Score	Best Ion Score	Best Ion C. I. %	Total Ion C. I. %	Confirmed	Sample Name	Customer sample Name
1	trkA gene product [Brucella suis 1330]	gi|23501992|ref|NP_698119.1|	27	414	100	199	93	100	100	.T.	AlDahouk_6214_Z19769-28_T1	482
2	hypothetical protein BSUIS_B0533 [Brucella suis ATCC 23445]	gi|163844696|ref|YP_001622351.1|	13	58	97.839					.T.	AlDahouk_6214_Z19769-28_T1	482

**Gel Idx/Pos**	206/I6	**Instr./Gel Origin**	AK043/Div_120507	**Process Status**	Analysis Succeeded
**Plate [#] Name**	[1] 1300017700	**Instrument Sample Name**		**Spectra**	4

Rank	Protein Name	Accession No.	Pep. Count	Protein Score	Protein Score C. I. %	Total Ion Score	Best Ion Score	Best Ion C. I. %	Total Ion C. I. %	Confirmed	Sample Name	Customer sample Name
1	unnamed protein product [Brucella suis 1330]	gi|23502737|ref|NP_698864.1|	22	324	100	159	159	100	100	.T.	AlDahouk_6214_Z19769-29_T1	495
2	gatB gene product [Brucella suis 1330]	gi|23501785|ref|NP_697912.1|	17	212	100	122	79	100	100	.T.	AlDahouk_6214_Z19769-29_T1	495
3	aspartyl/glutamyl-tRNA amidotransferase subunit B [Brucella suis ATCC 23445]	gi|163843171|ref|YP_001627575.1|	17	212	100	122	79	100	100	.T.	AlDahouk_6214_Z19769-29_T1	495

**Gel Idx/Pos**	207/I7	**Instr./Gel Origin**	AK043/Div_120507	**Process Status**	Analysis Succeeded
**Plate [#] Name**	[1] 1300017700	**Instrument Sample Name**		**Spectra**	4

Rank	Protein Name	Accession No.	Pep. Count	Protein Score	Protein Score C. I. %	Total Ion Score	Best Ion Score	Best Ion C. I. %	Total Ion C. I. %	Confirmed	Sample Name	Customer sample Name
1	unnamed protein product [Brucella suis 1330]	gi|23502737|ref|NP_698864.1|	22	472	100	306	144	100	100	.T.	AlDahouk_6214_Z19769-30_T1	2070
2	gatB gene product [Brucella suis 1330]	gi|23501785|ref|NP_697912.1|	19	168	100	58	58	100	100	.T.	AlDahouk_6214_Z19769-30_T1	2070
3	aspartyl/glutamyl-tRNA amidotransferase subunit B [Brucella suis ATCC 23445]	gi|163843171|ref|YP_001627575.1|	19	167	100	58	58	100	100	.T.	AlDahouk_6214_Z19769-30_T1	2070

4	hisS gene product [Brucella suis 1330]	gi|23499949|ref|NP_699389.1|	20	134	100	22	22	96.579	96.579	.T.	AlDahouk_6214_Z19769-30_T1	2070
5	histidyl-tRNA synthetase [Brucella suis ATCC 23445]	gi|163844376|ref|YP_001622031.1|	20	130	100	22	22	96.579	96.579	.T.	AlDahouk_6214_Z19769-30_T1	2070
6	hypothetical protein BSUIS_B0533 [Brucella suis ATCC 23445]	gi|163844696|ref|YP_001622351.1|	15	76	99.968					.T.	AlDahouk_6214_Z19769-30_T1	2070

Gel Idx/Pos	208/I8	Instr./Gel Origin	AK043/Div_120507	Process Status	Analysis Succeeded
Plate [#] Name	[1] 1300017700	Instrument Sample Name		Spectra	4

Rank	Protein Name	Accession No.	Pep. Count	Protein Score	Protein Score C. I. %	Total Ion Score	Best Ion Score	Best Ion C. I. %	Total Ion C. I. %	Confirmed	Sample Name	Customer sample Name
1	unnamed protein product [Brucella suis 1330]	gi|23502737|ref|NP_698864.1|	13	243	100	172	94	100	100	.T.	AlDahouk_6214_Z19769-50_T1	488
2	gatB gene product [Brucella suis 1330]	gi|23501785|ref|NP_697912.1|	17	184	100	96	96	100	100	.T.	AlDahouk_6214_Z19769-50_T1	488
3	aspartyl/glutamyl-tRNA amidotransferase subunit B [Brucella suis ATCC 23445]	gi|163843171|ref|YP_001627575.1|	17	183	100	96	96	100	100	.T.	AlDahouk_6214_Z19769-50_T1	488
4	trkA gene product [Brucella suis 1330]	gi|23501992|ref|NP_698119.1|	22	152	100					.T.	AlDahouk_6214_Z19769-50_T1	488

Gel Idx/Pos	209/I9	Instr./Gel Origin	AK043/Div_120507	Process Status	Analysis Succeeded
Plate [#] Name	[1] 1300017700	Instrument Sample Name		Spectra	4

Rank	Protein Name	Accession No.	Pep. Count	Protein Score	Protein Score C. I. %	Total Ion Score	Best Ion Score	Best Ion C. I. %	Total Ion C. I. %	Confirmed	Sample Name	Customer sample Name
1	metG gene product [Brucella suis 1330]	gi|23501876|ref|NP_698003.1|	30	435	100	193	76	100	100	.T.	AlDahouk_6214_Z19769-52_T1	554
2	methionyl-tRNA synthetase [Brucella suis ATCC 23445]	gi|163843264|ref|YP_001627668.1|	29	420	100	193	76	100	100	.T.	AlDahouk_6214_Z19769-52_T1	554
3	DEAD/DEAH box helicase [Brucella suis ATCC 23445]	gi|163843319|ref|YP_001627723.1|	17	63	99.362					.T.	AlDahouk_6214_Z19769-52_T1	554
4	unnamed protein product [Brucella suis 1330]	gi|23501498|ref|NP_697625.1|	13	60	98.698					.T.	AlDahouk_6214_Z19769-52_T1	554
5	unnamed protein product [Brucella suis 1330]	gi|23501930|ref|NP_698057.1|	16	58	97.983					.T.	AlDahouk_6214_Z19769-52_T1	554

Gel Idx/Pos	210/I10	Instr./Gel Origin	AK043/Div_120507	Process Status	Analysis Succeeded
Plate [#] Name	[1] 1300017700	Instrument Sample Name		Spectra	4

Rank	Protein	Name	Accession No.	Pep. Count	Protein Score	Protein Score C. I. %	Total Ion Score	Best Ion Score	Best Ion C. I. %	Total Ion C. I. %	Confirmed	Sample Name	Customer sample Name
1	secB gene product [Brucella suis 1330]		gi|23502920|ref|NP_699047.1|	6	229	100	189	86	100	100	.T.	AlDahouk_6214_Z19769-55_T1	560

Gel Idx/Pos	211/I11	Instr./Gel Origin	AK043/Div_120507	Process Status	Analysis Succeeded
Plate [#] Name	[1] 1300017700	Instrument Sample Name		Spectra	4

Rank	Protein	Name	Accession No.	Pep. Count	Protein Score	Protein Score C. I. %	Total Ion Score	Best Ion Score	Best Ion C. I. %	Total Ion C. I. %	Confirmed	Sample Name	Customer sample Name
1	chaperonin GroEL [Brucella suis ATCC 23445]		gi|163844383|ref|YP_001622038.1|	25	364	100	206	128	100	100	.T.	AlDahouk_6214_Z19769-59_T1	1416
2	hslU gene product [Brucella suis 1330]		gi|23502927|ref|NP_699054.1|	24	211	100	76	76	100	100	.T.	AlDahouk_6214_Z19769-59_T1	1416
3	pyk gene product [Brucella suis 1330]		gi|23502605|ref|NP_698732.1|	15	78	99.981					.T.	AlDahouk_6214_Z19769-59_T1	1416
4	pyruvate kinase [Brucella suis ATCC 23445]		gi|163845326|ref|YP_001622981.1|	14	68	99.807					.T.	AlDahouk_6214_Z19769-59_T1	1416

Gel Idx/Pos	213/I13	Instr./Gel Origin	AK043/Div_120507	Process Status	Analysis Succeeded
Plate [#] Name	[1] 1300017700	Instrument Sample Name		Spectra	4

Rank	Protein	Name	Accession No.	Pep. Count	Protein Score	Protein Score C. I. %	Total Ion Score	Best Ion Score	Best Ion C. I. %	Total Ion C. I. %	Confirmed	Sample Name	Customer sample Name
1	chaperonin GroEL [Brucella suis ATCC 23445]		gi|163844383|ref|YP_001622038.1|	29	568	100	364	128	100	100	.T.	AlDahouk_6214_Z19769-72_T1	420

AlDahouk_6214

TOPLAB Gesellschaft für angewandte Biotechnologie mbH

Analysis Information

**Report Type**	Protein-Peptide Summary by Spot	**Analysis Type**	Combined (MS+MS/MS)
**Sample Set Name**	Div_120507	**Database**	Bruc_suis_DEC2011
**Analysis Name**	AlDahouk_6214	**Creation Date**	05/30/2012 10:00:47
**Reported By**	08/17/2012 17:55:06 - admin	**Last Modified**	05/30/2012 13:29:11

**MS Acq. : Proc. Methods**	(Unspecified) : (Unspecified)
**Interpretation Method**	(Unspecified)

**Gel Idx/Pos**<br>**Plate [#] Name**	200/CAL 8<br>[1] 1300017700	**Instr./Gel Origin**<br>**Instrument Sample Name**	AK043/Div_120507	**Process Status**<br>**Spectra**	Analysis Succeeded<br>12

Rank	Protein	Name	Accession No.	Pep. Count	Protein Score	Protein Score C. I. %	Total Ion Score	Best Ion Score	Best Ion C. I. %	Total Ion C. I. %	Confirmed	Sample Name	Customer sample Name

No Confirmed Protein Found

**Gel Idx/Pos**<br>**Plate [#] Name**	176/H3<br>[1] 1300017700	**Instr./Gel Origin**<br>**Instrument Sample Name**	AK043/Div_120507	**Process Status**<br>**Spectra**	Analysis Succeeded<br>4

Rank	Protein Name	Accession No.	Pep. Count	Protein Score	Protein Score C. I. %	Total Ion Score	Best Ion Score	Best Ion C. I. %	Total Ion C. I. %	Confirmed	Sample Name	Customer sample Name
1	hypothetical protein BSUIS_A1239 [Brucella suis ATCC 23445]	gi|163843456|ref|YP_001627860.1|	10	171	100	87	87	100	100	.T.	AlDahouk_6214_Z19769-1_T1	1499

Peptide Information

Calc. Mass	Obsrv. Mass	± da	± ppm	Start Seq.	End Seq.	Sequence	Ion Score	C. I. %	Modification	Rank	Result Type
757.4566	757.4601	0.0035	5	2	7	EIVQIR					Mascot
787.442	787.4982	0.0562	71	44	49	DEVIRR					Mascot
794.4406	794.5091	0.0685	86	8	15	ISSVGGFK					Mascot
834.3839	834.3791	-0.0048	-6	33	39	IENDTDK					Mascot
888.4971	888.5604	0.0633	71	1	7	MEIVQIR					Mascot
904.492	904.555	0.063	70	1	7	MEIVQIR			Oxidation (M)[1]		Mascot
1016.5119	1016.5838	0.0719	71	81	89	AGDRAELER					Mascot
1232.6117	1232.6953	0.0836	68	33	42	IENDTDKELR					Mascot

2	unnamed protein product [Brucella suis 1330]	gi|23502068|ref|NP_698195.1|	8	143	100	87	87	100	100	.T.	AlDahouk_6214_Z19769-1_T1	1499

Peptide Information

Calc. Mass	Obsrv. Mass	± da	± ppm	Start Seq.	End Seq.	Sequence	Ion Score	C. I. %	Modification	Rank	Result Type
787.442	787.4982	0.0562	71	67	72	DEVIRR					Mascot
794.4406	794.5091	0.0685	86	31	38	ISSVGGFK					Mascot
834.3839	834.3791	-0.0048	-6	56	62	IENDTDK					Mascot
1016.5119	1016.5838	0.0719	71	104	112	AGDRAELER					Mascot
1232.6117	1232.6953	0.0836	68	56	65	IENDTDKELR					Mascot
1601.7516	1601.8594	0.1078	67	39	51	LYMVEFVTEGEER	87	100			Mascot
1601.7516	1601.8594	0.1078	67	39	51	LYMVEFVTEGEER					Mascot
1617.7465	1617.8573	0.1108	68	39	51	LYMVEFVTEGEER			Oxidation (M)[3]		Mascot
2778.2678	2778.427	0.1592	57	77	102	LGDAMGMACAECGIEPDSLLTRPSAR			Carbamidomethyl (C)[9,12]		Mascot
3044.4583	3044.6365	0.1782	59	113	142	QLDEGLEDTFPASDPVSVTSSAIPASADPK					Mascot

Gel Idx/Pos	177/H4	Instr./Gel Origin	AK043/Div_120507	Process Status	Analysis Succeeded
Plate [#] Name	[1] 1300017700	Instrument Sample Name		Spectra	4

Rank	Protein Name	Accession No.	Pep. Count	Protein Score	Protein Score C. I. %	Total Ion Score	Best Ion Score	Best Ion C. I. %	Total Ion C. I. %	Confirmed	Sample Name	Customer sample Name
1	bacterioferritin [Brucella suis ATCC 23445]	gi|163844717|ref|YP_001622372.1|	6	161	100	127	83	100	100	.T.	AlDahouk_6214_Z19769-3_T1	1467

Peptide Information

Calc. Mass	Obsrv. Mass	± da	± ppm	Start Seq.	End Seq.	Sequence	Ion Score	C. I. %	Modification	Rank	Result Type
705.3599	705.4011	0.0412	58	1	6	MKGEPK			Oxidation (M)[1]		Mascot
840.421	840.4622	0.0412	49	99	105	ASYKESR					Mascot
1137.5687	1137.6415	0.0728	64	31	39	LLNDWGYTR	44	99.98			Mascot
1137.5687	1137.6415	0.0728	64	31	39	LLNDWGYTR					Mascot
1607.7911	1607.9	0.1089	68	85	98	EVLEADLKGEYDAR					Mascot
1944.0953	1944.2172	0.1219	63	62	78	IIFLEGFPNLQTVSPLR	83	100			Mascot
1944.0953	1944.2172	0.1219	63	62	78	IIFLEGFPNLQTVSPLR					Mascot
2449.2664	2449.4094	0.143	58	11	30	LNEALFLELGAVNQYWLHYR					Mascot

Gel Idx/Pos	178/H5	Instr./Gel Origin	AK043/Div_120507	Process Status	Analysis Succeeded
Plate [#] Name	[1] 1300017700	Instrument Sample Name		Spectra	4

Rank	Protein Name	Accession No.	Pep. Count	Protein Score	Protein Score C. I. %	Total Ion Score	Best Ion Score	Best Ion C. I. %	Total Ion C. I. %	Confirmed	Sample Name	Customer sample Name
1	bacterioferritin [Brucella suis ATCC 23445]	gi|163844717|ref|YP_001622372.1|	14	459	100	336	134	100	100	.T.	AlDahouk_6214_Z19769-5_T1	2081

Peptide Information

Calc. Mass	Obsrv. Mass	± da	± ppm	Start Seq.	End Seq.	Sequence	Ion Score	C. I. %	Modification	Rank	Result Type
705.3599	705.4054	0.0455	65	1	6	MKGEPK			Oxidation (M)[1]		Mascot
710.3104	710.3513	0.0409	58	93	98	GEYDAR					Mascot
781.409	781.4592	0.0502	64	111	117	LGDYVSK					Mascot
840.421	840.4784	0.0574	68	99	105	ASYKESR					Mascot
1137.5687	1137.6523	0.0836	73	31	39	LLNDWGYTR	76	100			Mascot
1137.5687	1137.6523	0.0836	73	31	39	LLNDWGYTR					Mascot
1348.6016	1348.7012	0.0996	74	149	161	YGQLNAAPADEAE					Mascot
1426.6882	1426.7925	0.1043	73	106	117	EICDKLGDYVSK			Carbamidomethyl (C)[3]		Mascot
1454.6217	1454.7281	0.1064	73	46	57	EESIEEMHHADK					Mascot
1607.7911	1607.9071	0.116	72	85	98	EVLEADLKGEYDAR	126	100			Mascot
1607.7911	1607.9071	0.116	72	85	98	EVLEADLKGEYDAR					Mascot
1739.7654	1739.8929	0.1275	73	44	57	EREESIEEMHHADK					Mascot
1944.0953	1944.2312	0.1359	70	62	78	IIFLEGFPNLQTVSPLR	134	100			Mascot
1944.0953	1944.2312	0.1359	70	62	78	IIFLEGFPNLQTVSPLR					Mascot
1951.9178	1952.0581	0.1403	72	46	61	EESIEEMHHADKLIDR					Mascot
1967.9127	1968.0961	0.1834	93	46	61	EESIEEMHHADKLIDR			Oxidation (M)[7]		Mascot
2449.2664	2449.4336	0.1672	68	11	30	LNEALFLELGAVNQYWLHYR					Mascot
3015.5198	3015.7368	0.217	72	118	143	QLFDELLADEEGHIDFLETQLDLLAK					Mascot

Gel Idx/Pos	179/H6	Instr./Gel Origin	AK043/Div_120507	Process Status	Analysis Succeeded
Plate [#] Name	[1] 1300017700	Instrument Sample Name		Spectra	4

Rank	Protein Name	Accession No.	Pep. Count	Protein Score	Protein Score C. I. %	Total Ion Score	Best Ion Score	Best Ion C. I. %	Total Ion C. I. %	Confirmed	Sample Name	Customer sample Name
1	grxC gene product [Brucella suis 1330]	gi|23502729|ref|N	8	384	100	299	146	100	100	.T.	AlDahouk_6	1983

P_698856.1| 214_Z19769-6_T1

Peptide Information

Calc. Mass	Obsrv. Mass	± da	± ppm	Start Seq.	End Seq.	Sequence	Ion Score	C. I. %	Modification	Rank	Result Type
742.4933	742.4419	-0.0514	-69	18	24	AKALLAR					Mascot
763.3403	763.393	0.0527	69	42	47	AEMQER					Mascot
779.3352	779.3886	0.0534	69	42	47	AEMQER			Oxidation (M)[3]		Mascot
1719.8184	1719.9432	0.1248	73	26	41	GAEFNEIDASATPELR	106	100			Mascot
1719.8184	1719.9432	0.1248	73	26	41	GAEFNEIDASATPELR					Mascot
1847.9133	1848.0493	0.136	74	25	41	KGAEFNEIDASATPELR	146	100			Mascot
1847.9133	1848.0493	0.136	74	25	41	KGAEFNEIDASATPELR					Mascot
1939.9517	1940.0924	0.1407	73	2	17	VDVIIYTRPGCPYCAR	46	99.989	Carbamidomethyl (C)[11,14]		Mascot
1939.9517	1940.0924	0.1407	73	2	17	VDVIIYTRPGCPYCAR			Carbamidomethyl (C)[11,14]		Mascot
2070.9922	2071.157	0.1648	80	1	17	MVDVIIYTRPGCPYCAR			Carbamidomethyl (C)[12,15]		Mascot
2086.9871	2087.1567	0.1696	81	1	17	MVDVIIYTRPGCPYCAR			Carbamidomethyl (C)[12,15], Oxidation (M)[1]		Mascot
2981.3984	2981.5955	0.1971	66	51	77	NTFPQIFIGSVHVGGCDD LYALEDEGK			Carbamidomethyl (C)[16]		Mascot
3650.8047	3651.0615	0.2568	70	51	83	NTFPQIFIGSVHVGGCDD LYALEDEGKLDSLLK			Carbamidomethyl (C)[16]		Mascot

Gel Idx/Pos	180/H7	Instr./Gel Origin	AK043/Div_120507	Process Status	Analysis Succeeded
Plate [#] Name	[1] 1300017700	Instrument Sample Name		Spectra	4

Rank	Protein Name	Accession No.	Pep. Count	Protein Score	Protein Score C. I. %	Total Ion Score	Best Ion Score	Best Ion C. I. %	Total Ion C. I. %	Confirmed	Sample Name	Customer sample Name
1	etfB gene product [Brucella suis 1330]	gi|23502819|ref|NP_698946.1|	22	582	100	387	199	100	100	.T.	AlDahouk_6214_Z19769-7_T1	1082

Peptide Information

Calc. Mass	Obsrv. Mass	± da	± ppm	Start Seq.	End Seq.	Sequence	Ion Score	C. I. %	Modification	Rank	Result Type
715.4348	715.4719	0.0371	52	242	248	LKADGVL					Mascot
745.3475	745.3899	0.0424	57	220	226	TEEPGGR					Mascot
836.4512	836.5075	0.0563	67	10	16	VVDYNVK					Mascot
857.509	857.5101	0.0011	1	196	202	KKPLDEK					Mascot
873.4424	873.5031	0.0607	69	220	227	TEEPGGRK					Mascot
889.4625	889.5258	0.0633	71	146	154	VELGEGSAK					Mascot

905.4509	905.5118	0.0609	67	74	82	TALAMGADR					Mascot
921.4458	921.5093	0.0635	69	74	82	TALAMGADR			Oxidation (M)[5]		Mascot
992.5523	992.6199	0.0676	68	9	16	RVVDYNVK					Mascot
1046.5728	1046.6471	0.0743	71	232	241	VGSVSELVEK					Mascot
1085.595	1085.6704	0.0754	69	217	226	VLKTEEPGGR					Mascot
1096.6725	1096.7599	0.0874	80	170	179	LPAIVTVDLR	82	100			Mascot
1096.6725	1096.7599	0.0874	80	170	179	LPAIVTVDLR					Mascot
1216.5957	1216.6938	0.0981	81	203	214	SPADFGADIAPR	106	100			Mascot
1216.5957	1216.6938	0.0981	81	203	214	SPADFGADIAPR					Mascot
1258.6526	1258.743	0.0904	72	88	99	TDETVEPLGVAK					Mascot
1273.6635	1273.7533	0.0898	71	158	169	EVDGGLQTIDVK					Mascot
1372.743	1372.8413	0.0983	72	19	32	VKGDGSGVELANVK					Mascot
1600.8944	1601.0146	0.1202	75	103	117	GVVEAEKPDLVFLGK					Mascot
1851.8616	1852.0038	0.1422	77	33	48	MSMNPFDEIAVEEAIR					Mascot
1867.8564	1868.0045	0.1481	79	33	48	MSMNPFDEIAVEEAIR			Oxidation (M)[1]		Mascot
1883.8513	1884.0006	0.1493	79	33	48	MSMNPFDEIAVEEAIR			Oxidation (M)[1,3]		Mascot
1926.9919	1927.0741	0.0822	43	197	214	KPLDEKSPADFGADIAPR					Mascot
1968.076	1968.2255	0.1495	76	55	73	VTEIVAVSVGPAQAQETLR	199	100			Mascot
1968.076	1968.2255	0.1495	76	55	73	VTEIVAVSVGPAQAQETLR					Mascot
2351.3181	2351.4839	0.1658	71	158	179	EVDGGLQTIDVKLPAIVTVDLR					Mascot
2353.2722	2353.4536	0.1814	77	51	73	EAGKVTEIVAVSVGPAQAQETLR					Mascot
3040.4316	3040.667	0.2354	77	118	145	QAIDDDSNQTGQMLSALLNWSQATFASK					Mascot
3056.4265	3056.6782	0.2517	82	118	145	QAIDDDSNQTGQMLSALLNWSQATFASK			Oxidation (M)[13]		Mascot

2 unnamed protein product [Brucella suis 1330] gi|23500019|ref|NP_699459.1| 16 109 100 .T. AlDahouk_6214_Z19769-7_T1 1082

Peptide Information

Calc. Mass	Obsrv. Mass	± da	± ppm	Start Seq.	End Seq.	Sequence	Ion Score	C. I. %	Modification	Rank	Result Type
704.3573	704.4064	0.0491	70	169	174	EETGIR					Mascot
759.4471	759.4213	-0.0258	-34	161	167	LSRQAGK					Mascot
765.473	765.5233	0.0503	66	154	160	VAHAVLR					Mascot
847.4672	847.5273	0.0601	71	175	181	IDFPISR					Mascot
855.4319	855.4928	0.0609	71	135	141	LEEAHTR					Mascot

Calc. Mass	Obsrv. Mass	± da	± ppm	Start Seq.	End Seq.	Sequence	Ion Score	C. I. %	Modification	Rank	Result Type
896.4836	896.5391	0.0555	62	26	33	LVSYATSR					Mascot
921.574	921.5093	-0.0647	-70	153	160	RVAHAVLR					Mascot
990.5255	990.5917	0.0662	67	198	205	ILSNWETK					Mascot
1011.533	1011.605	0.072	71	134	141	RLEEAHTR					Mascot
1046.5775	1046.6471	0.0696	67	2	10	AMIDRGLVR			Oxidation (M)[2]		Mascot
1056.6411	1056.715	0.0739	70	215	224	LLVLDSAGLR					Mascot
1212.7423	1212.8325	0.0902	74	215	225	LLVLDSAGLRR					Mascot
1346.6674	1346.7654	0.098	73	75	86	MVHPGDLFGFAR					Mascot
1362.6624	1362.7615	0.0991	73	75	86	MVHPGDLFGFAR			Oxidation (M)[1]		Mascot
1566.8599	1566.976	0.1161	74	61	74	VNQVTPDGQQIIVR					Mascot
1759.8644	1759.9973	0.1329	76	182	197	QDIAEMTGTTLHTVSR					Mascot
1775.8593	1775.9883	0.129	73	182	197	QDIAEMTGTTLHTVSR			Oxidation (M)[6]		Mascot
1808.0388	1808.1708	0.132	73	59	74	LKVNQVTPDGQQIIVR					Mascot

3 hypothetical protein BSUIS_B0267 [Brucella suis ATCC 23445] gi|163844446|ref|YP_001622101.1| 15 93 100 .T. AlDahouk_6214_Z19769-7_T1 1082

Peptide Information

Calc. Mass	Obsrv. Mass	± da	± ppm	Start Seq.	End Seq.	Sequence	Ion Score	C. I. %	Modification	Rank	Result Type
704.3573	704.4064	0.0491	70	169	174	EETGIR					Mascot
759.4471	759.4213	-0.0258	-34	161	167	LSRQAGK					Mascot
765.473	765.5233	0.0503	66	154	160	VAHAVLR					Mascot
847.4672	847.5273	0.0601	71	175	181	IDFPISR					Mascot
855.4319	855.4928	0.0609	71	135	141	LEEAHTR					Mascot
896.4836	896.5391	0.0555	62	26	33	LVSYATSR					Mascot
921.574	921.5093	-0.0647	-70	153	160	RVAHAVLR					Mascot
990.5255	990.5917	0.0662	67	198	205	ILSNWETK					Mascot
1011.533	1011.605	0.072	71	134	141	RLEEAHTR					Mascot
1046.5775	1046.6471	0.0696	67	2	10	AMIDRGLVR			Oxidation (M)[2]		Mascot
1056.6411	1056.715	0.0739	70	215	224	LLVLDSAGLR					Mascot
1212.7423	1212.8325	0.0902	74	215	225	LLVLDSAGLRR					Mascot
1346.6674	1346.7654	0.098	73	75	86	MVHPGDLFGFAR					Mascot
1362.6624	1362.7615	0.0991	73	75	86	MVHPGDLFGFAR			Oxidation (M)[1]		Mascot
1566.8599	1566.976	0.1161	74	61	74	VNQVTPDGQQIIVR					Mascot
1808.0388	1808.1708	0.132	73	59	74	LKVNQVTPDGQQIIVR					Mascot

Gel Idx/Pos	181/H8	Instr./Gel Origin	AK043/Div_120507	Process Status	Analysis Succeeded
Plate [#] Name	[1] 1300017700	Instrument Sample Name		Spectra	4

Rank	Protein	Name	Accession No.	Pep. Count	Protein Score	Protein Score C. I. %	Total Ion Score	Best Ion Score	Best Ion C. I. %	Total Ion C. I. %	Confirmed	Sample Name	Customer sample Name
1	unnamed protein product [Brucella suis 1330]		gi|23501191|ref|NP_697318.1|	9	189	100	121	121	100	100	.T.	AlDahouk_6214_Z19769-8_T1	1731

Peptide Information

Calc. Mass	Obsrv. Mass	± da	± ppm	Start Seq.	End Seq.	Sequence	Ion Score	C. I. %	Modification	Rank	Result Type
715.446	715.4821	0.0361	50	124	130	LLGSVAR					Mascot
730.4206	730.463	0.0424	58	117	123	VAQLGSR					Mascot
763.4712	763.52	0.0488	64	50	56	VSFGILK					Mascot
847.452	847.5163	0.0643	76	39	45	ISETEIR					Mascot
950.4247	950.4994	0.0747	79	1	8	MDLTGEER					Mascot
966.4196	966.4939	0.0743	77	1	8	MDLTGEER			Oxidation (M)[1]		Mascot
1182.6154	1182.7048	0.0894	76	136	146	FFTNIAEAAAK					Mascot
1247.5508	1247.6444	0.0936	75	28	38	ACIPGCDGIER			Carbamidomethyl (C)[2,6]		Mascot
1615.8326	1615.9525	0.1199	74	14	27	QAVWDALNDIETLK					Mascot
1659.8701	1659.9939	0.1238	75	96	110	LHEDGSATVLSYVIR	121	100			Mascot
1659.8701	1659.9939	0.1238	75	96	110	LHEDGSATVLSYVIR					Mascot

Rank	Protein	Name	Accession No.	Pep. Count	Protein Score	Protein Score C. I. %	Total Ion Score	Best Ion Score	Best Ion C. I. %	Total Ion C. I. %	Confirmed	Sample Name	Customer sample Name
2	unnamed protein product [Brucella suis 1330]		gi|23502043|ref|NP_698170.1|	6	138	100	96	96	100	100	.T.	AlDahouk_6214_Z19769-8_T1	1731

Peptide Information

Calc. Mass	Obsrv. Mass	± da	± ppm	Start Seq.	End Seq.	Sequence	Ion Score	C. I. %	Modification	Rank	Result Type
844.4523	844.5172	0.0649	77	81	88	AALGELDR					Mascot
848.4108	848.4822	0.0714	84	2	8	TDTIENR					Mascot
937.5829	937.6542	0.0713	76	49	57	LVHTGLIGK					Mascot
1158.63	1158.7158	0.0858	74	70	80	ACAINVLAQAK			Carbamidomethyl (C)[2]		Mascot
1201.6172	1201.7091	0.0919	76	58	69	ELTVADGQAAAR	96	100			Mascot
1201.6172	1201.7091	0.0919	76	58	69	ELTVADGQAAAR					Mascot
3365.8103	3366.0498	0.2395	71	95	128	ITVFVASAPGFVEQHLVANGASDLLVAVLGDAGK					Mascot

3	endoribonuclease L-PSP [Brucella suis ATCC 23445]	gi|163843429|ref|YP_001627833.1|	5	130	100	96	96	100	100	.T.	AlDahouk_6 1731 214_Z19769-8_T1

Peptide Information

Calc. Mass	Obsrv. Mass	± da	± ppm	Start Seq.	End Seq.	Sequence	Ion Score	C. I. %	Modification	Rank	Result Type
848.4108	848.4822	0.0714	84	2	8	TDTIENR					Mascot
937.5829	937.6542	0.0713	76	49	57	LVHTGLIGK					Mascot
1158.63	1158.7158	0.0858	74	70	80	ACAINVLAQAK			Carbamidomethyl (C)[2]		Mascot
1201.6172	1201.7091	0.0919	76	58	69	ELTVADGQAAAR	96	100			Mascot
1201.6172	1201.7091	0.0919	76	58	69	ELTVADGQAAAR					Mascot
3365.8103	3366.0498	0.2395	71	95	128	ITVFVASAPGFVEQHLVANGASDLLVAVLGDAGK					Mascot

4	homoprotocatechuate degradation operon regulator, HpaR [Brucella suis ATCC 23445]	gi|163845479|ref|YP_001623134.1|	9	103	100	60	60	100	100	.T.	AlDahouk_6 1731 214_Z19769-8_T1

Peptide Information

Calc. Mass	Obsrv. Mass	± da	± ppm	Start Seq.	End Seq.	Sequence	Ion Score	C. I. %	Modification	Rank	Result Type
778.3876	778.4331	0.0455	58	1	6	MQDKTR					Mascot
787.3944	787.4623	0.0679	86	127	133	EVAPESR					Mascot
840.4938	840.5554	0.0616	73	19	25	QDVLLPR					Mascot
868.5614	868.6255	0.0641	74	30	37	SVPIALLR					Mascot
1088.6462	1088.7269	0.0807	74	81	90	AFILAPSLTR	60	100			Mascot
1088.6462	1088.7269	0.0807	74	81	90	AFILAPSLTR					Mascot
1097.6426	1097.7258	0.0832	76	17	25	TRQDVLLPR					Mascot
1194.6188	1194.709	0.0902	76	134	142	LIYQMIEER					Mascot
1210.6136	1210.7068	0.0932	77	134	142	LIYQMIEER			Oxidation (M)[5]		Mascot
1449.9403	1450.0488	0.1085	75	113	126	VLLQITPAGLAIIK					Mascot
1762.8528	1762.9832	0.1304	74	65	80	ILAETDIVDASEMAER					Mascot
1778.8477	1778.9834	0.1357	76	65	80	ILAETDIVDASEMAER			Oxidation (M)[13]		Mascot

5	dut gene product [Brucella suis 1330]	gi|23502533|ref|NP_698660.1|	8	60	98.572					.T.	AlDahouk_6 1731 214_Z19769-8_T1

Peptide Information

Calc. Mass	Obsrv. Mass	± da	± ppm	Start Seq.	End Seq.	Sequence	Ion Score	C. I. %	Modification	Rank	Result Type

731.3682	731.437	0.0688	94	39	45	AAVAEDR					Mascot
895.5723	895.6351	0.0628	70	46	53	QIVLLPGR					Mascot
1051.6735	1051.761	0.0875	83	46	54	QIVLLPGRR					Mascot
1344.7482	1344.8416	0.0934	69	2	15	TAASSSAPTLGIIR					Mascot
1381.8202	1381.9213	0.1011	73	122	134	IAQAVFAPVIQPK					Mascot
1389.7373	1389.8397	0.1024	74	104	115	VLLINLGDDDFR					Mascot
1836.8796	1837.0094	0.1298	71	21	38	GLDLPAYETAGSAGMDLR					Mascot
1852.8745	1853.0165	0.142	77	21	38	GLDLPAYETAGSAGMDLR			Oxidation (M)[15]		Mascot
2492.4236	2492.5845	0.1609	65	55	76	TLVPTGLILEIPQGYEVQIRPR					Mascot

Gel Idx/Pos	182/H9	Instr./Gel Origin	AK043/Div_120507	Process Status	Analysis Succeeded
Plate [#] Name	[1] 1300017700	Instrument Sample Name		Spectra	4

Rank	Protein Name	Accession No.	Pep. Count	Protein Score	Protein Score C. I. %	Total Ion Score	Best Ion Score	Best Ion C. I. %	Total Ion C. I. %	Confirmed	Sample Name	Customer sample Name
1	50S ribosomal protein L33 [Brucella suis ATCC 23445]	gi|163844761|ref|YP_001622416.1|	6	222	100	182	125	100	100	.T.	AlDahouk_6214_Z19769-9_T1	1933

Peptide Information

Calc. Mass	Obsrv. Mass	± da	± ppm	Start Seq.	End Seq.	Sequence	Ion Score	C. I. %	Modification	Rank	Result Type
734.3831	734.4283	0.0452	62	39	44	YDPIAR					Mascot
787.446	787.5044	0.0584	74	45	50	KHVEFK					Mascot
963.5258	963.6006	0.0748	78	37	44	TKYDPIAR	57	100			Mascot
963.5258	963.6006	0.0748	78	37	44	TKYDPIAR					Mascot
1017.5363	1017.5767	0.0404	40	46	53	HVEFKETK					Mascot
1562.8101	1562.9413	0.1312	84	11	24	LLSTADTGFFYVTK	125	100			Mascot
1562.8101	1562.9413	0.1312	84	11	24	LLSTADTGFFYVTK					Mascot
1690.905	1691.0424	0.1374	81	11	25	LLSTADTGFFYVTKK					Mascot

Gel Idx/Pos	183/H10	Instr./Gel Origin	AK043/Div_120507	Process Status	Analysis Succeeded
Plate [#] Name	[1] 1300017700	Instrument Sample Name		Spectra	4

Rank	Protein Name	Accession No.	Pep. Count	Protein Score	Protein Score C. I. %	Total Ion Score	Best Ion Score	Best Ion C. I. %	Total Ion C. I. %	Confirmed	Sample Name	Customer sample Name
1	chaperonin GroEL [Brucella suis ATCC 23445]	gi|163844383|ref|Y	21	262	100	148	84	100	100	.T.	AlDahouk_6	384

P_001622038.1| 214_Z19769-10_T1

Peptide Information

Calc. Mass	Obsrv. Mass	± da	± ppm	Start Seq.	End Seq.	Sequence	Ion Score	C. I. %	Modification	Rank	Result Type
707.3947	707.4001	0.0054	8	8	13	FGRTAR					Mascot
719.347	719.3906	0.0436	61	278	284	APGFGDR					Mascot
721.3991	721.4317	0.0326	45	5	10	DVKFGR					Mascot
726.4257	726.4708	0.0451	62	445	451	AIQAPAR					Mascot
762.4178	762.4662	0.0484	63	1	7	MAAKDVK					Mascot
855.541	855.6097	0.0687	80	372	380	LAGGVAVIR					Mascot
882.5268	882.6051	0.0783	89	444	451	RAIQAPAR					Mascot
1233.6508	1233.7443	0.0935	76	309	319	LESVTLDMLGR					Mascot
1245.6685	1245.7208	0.0523	42	381	392	VGGATEVEVKEK					Mascot
1249.6456	1249.7411	0.0955	76	309	319	LESVTLDMLGR			Oxidation (M)[8]		Mascot
1344.7004	1344.8033	0.1029	77	169	181	VGNEGVITVEEAK					Mascot
1455.755	1455.8683	0.1133	78	430	443	GVNADQEAGINIVR	64	100			Mascot
1455.755	1455.8683	0.1133	78	430	443	GVNADQEAGINIVR					Mascot
1484.65	1484.7812	0.1312	88	351	362	QQIEETTSDYDR					Mascot
1514.7526	1514.8726	0.12	79	198	210	GYLSPYFVTNPEK					Mascot
1582.905	1583.0101	0.1051	66	119	133	GIDLAVNEVVAELLK					Mascot
1583.8751	1583.9998	0.1247	79	405	421	AAVEEGIVAGGGTALLR	84	100			Mascot
1583.8751	1583.9998	0.1247	79	405	421	AAVEEGIVAGGGTALLR					Mascot
1741.7875	1741.9265	0.139	80	351	364	QQIEETTSDYDREK					Mascot
1759.8934	1760.0315	0.1381	78	211	225	MVADLEDAYILLHEK					Mascot
1775.8884	1776.0339	0.1455	82	211	225	MVADLEDAYILLHEK			Oxidation (M)[1]		Mascot
1853.8585	1854.0007	0.1422	77	182	197	TAETELEVVEGMQFDR					Mascot
1869.8535	1870.0081	0.1546	83	182	197	TAETELEVVEGMQFDR			Oxidation (M)[12]		Mascot
1903.9834	1904.1605	0.1771	93	211	226	MVADLEDAYILLHEKK			Oxidation (M)[1]		Mascot
2038.9572	2039.1107	0.1535	75	59	75	EVELEDKFENMGAQMLR					Mascot
2419.1946	2419.3921	0.1975	82	81	105	TNDTAGDGTTTATVLGQAIVQEGAK					Mascot

2	katA gene product [Brucella suis 1330]	gi|23500108|ref|NP_699548.1|	14	68	99.768	.T.	AlDahouk_6214_Z19769-10_T1	384

Peptide Information

Calc. Mass	Obsrv. Mass	± da	± ppm	Start Seq.	End Seq.	Sequence	Ion Score	C. I. %	Modification	Rank	Result Type
729.4042	729.4608	0.0566	78	459	464	QLGHFK					Mascot
754.3954	754.4499	0.0545	72	41	46	LSHQNR					Mascot
973.5464	973.6202	0.0738	76	450	458	GVPGFIVER					Mascot
1079.5269	1079.6106	0.0837	78	334	342	IFSYADAHR					Mascot
1121.5421	1121.6338	0.0917	82	359	366	CLVHHYHR			Carbamidomethyl (C)[1]		Mascot
1164.5281	1164.6193	0.0912	78	417	426	IGNDDYSQPR					Mascot
1237.6576	1237.7538	0.0962	78	427	437	ALFNLFDAAQK					Mascot
1283.6742	1283.7748	0.1006	78	465	475	LIHPEYEAGVR					Mascot
1328.6263	1328.735	0.1087	82	401	412	EPPLCISGNADR			Carbamidomethyl (C)[5]		Mascot
1381.7621	1381.8689	0.1068	77	79	91	VLQPGAQTPMLAR					Mascot
1510.7397	1510.8606	0.1209	80	223	235	HWTNAEAEQVIGR					Mascot
1596.889	1597.0121	0.1231	77	77	91	AKVLQPGAQTPMLAR			Oxidation (M)[12]		Mascot
1667.8387	1667.9659	0.1272	76	58	73	GWGAYGTLTITGDISR					Mascot
2503.2041	2503.3767	0.1726	69	115	135	FYTQEGNWDLVGNNTPVFFVR					Mascot

3 hypothetical protein BSUIS_B0360 [Brucella suis ATCC 23445] gi|163844531|ref|YP_001622186.1| 13 60 98.667 .T. AlDahouk_6214_Z19769-10_T1 384

Peptide Information

Calc. Mass	Obsrv. Mass	± da	± ppm	Start Seq.	End Seq.	Sequence	Ion Score	C. I. %	Modification	Rank	Result Type
729.4042	729.4608	0.0566	78	459	464	QLGHFK					Mascot
754.3954	754.4499	0.0545	72	41	46	LSHQNR					Mascot
973.5464	973.6202	0.0738	76	450	458	GVPGFIVER					Mascot
1079.5269	1079.6106	0.0837	78	334	342	IFSYADAHR					Mascot
1164.5281	1164.6193	0.0912	78	417	426	IGNDDYSQPR					Mascot
1237.6576	1237.7538	0.0962	78	427	437	ALFNLFDAAQK					Mascot
1283.6742	1283.7748	0.1006	78	465	475	LIHPEYEAGVR					Mascot
1328.6263	1328.735	0.1087	82	401	412	EPPLCISGNADR			Carbamidomethyl (C)[5]		Mascot
1381.7621	1381.8689	0.1068	77	79	91	VLQPGAQTPMLAR					Mascot
1510.7397	1510.8606	0.1209	80	223	235	HWTNAEAEQVIGR					Mascot
1596.889	1597.0121	0.1231	77	77	91	AKVLQPGAQTPMLAR			Oxidation (M)[12]		Mascot
1667.8387	1667.9659	0.1272	76	58	73	GWGAYGTLTITGDISR					Mascot
2503.2041	2503.3767	0.1726	69	115	135	FYTQEGNWDLVGNNTPV					Mascot

FFVR

Gel Idx/Pos	184/H11	Instr./Gel Origin	AK043/Div_120507	Process Status	Analysis Succeeded
Plate [#] Name	[1] 1300017700	Instrument Sample Name		Spectra	4

Rank	Protein	Name	Accession No.	Pep. Count	Protein Score	Protein Score C. I. %	Total Ion Score	Best Ion Score	Best Ion C. I. %	Total Ion C. I. %	Confirmed	Sample Name	Customer sample Name
1	pyk gene product [Brucella suis 1330]		gi|23502605|ref|NP_698732.1|	19	242	100	119	61	100	100	.T.	AlDahouk_6214_Z19769-11_T1	389

Peptide Information

Calc. Mass	Obsrv. Mass	± da	± ppm	Start Seq.	End Seq.	Sequence	Ion Score	C. I. %	Modification	Rank	Result Type
802.4669	802.4631	-0.0038	-5	363	369	QIAETLK					Mascot
907.4883	907.545	0.0567	62	467	475	IAYIGADGK					Mascot
1041.6051	1041.6852	0.0801	77	218	226	IEKPQAVTR					Mascot
1124.5735	1124.6617	0.0882	78	26	35	LFEAGADVFR					Mascot
1252.6685	1252.7665	0.098	78	25	35	KLFEAGADVFR					Mascot
1393.6958	1393.7943	0.0985	71	335	346	EPTYSTIIDAQR					Mascot
1439.6519	1439.7733	0.1214	84	36	47	INMSHADHDLMR					Mascot
1452.842	1452.957	0.115	79	393	406	TPIIALSPVVDTAR					Mascot
1570.7893	1570.9152	0.1259	80	347	362	AMPEPTGADAISLAAR					Mascot
1596.8955	1597.0209	0.1254	79	9	24	ILATLGPASGEEAVIR					Mascot
1610.8749	1611.0013	0.1264	78	370	385	LSAIVTYTASGTTGLR					Mascot
1676.9694	1677.0994	0.13	78	59	74	ELGRPIGILADLQGPK	58	100			Mascot
1676.9694	1677.0994	0.13	78	59	74	ELGRPIGILADLQGPK					Mascot
1687.8934	1688.0247	0.1313	78	227	241	LDEIIELSDALMVAR					Mascot
1703.8884	1704.0225	0.1341	79	227	241	LDEIIELSDALMVAR			Oxidation (M)[12]		Mascot
1724.9905	1725.116	0.1255	73	9	25	ILATLGPASGEEAVIRK					Mascot
1763.9537	1764.0923	0.1386	79	242	258	GDLGVEVPLENVPGIQK					Mascot
1915.047	1915.1512	0.1042	54	448	466	VIITAGVPFGTPGATNMLR					Mascot
1931.0419	1931.1768	0.1349	70	448	466	VIITAGVPFGTPGATNMLR			Oxidation (M)[17]		Mascot
1940.0388	1940.1887	0.1499	77	107	123	VFLPHPEILEAVEPGHR	61	100			Mascot
1940.0388	1940.1887	0.1499	77	107	123	VFLPHPEILEAVEPGHR					Mascot
2219.1304	2219.3042	0.1738	78	328	346	IAEQVEREPTYSTIIDAQR					Mascot
2404.1626	2404.3511	0.1885	78	85	106	VDLVPGQTFTLDNNEALGDETR					Mascot

2	pyruvate kinase [Brucella suis ATCC 23445]	gi|163845326|ref|YP_001622981.1|	17	220	100	119	61	100	100	.T.	AlDahouk_6214_Z19769-11_T1	389

Peptide Information

Calc. Mass	Obsrv. Mass	± da	± ppm	Start Seq.	End Seq.	Sequence	Ion Score	C. I. %	Modification	Rank	Result Type
802.4669	802.4631	-0.0038	-5	363	369	QIAETLK					Mascot
907.4883	907.545	0.0567	62	467	475	IAYIGADGK					Mascot
1041.6051	1041.6852	0.0801	77	218	226	IEKPQAVTR					Mascot
1124.5735	1124.6617	0.0882	78	26	35	LFEAGADVFR					Mascot
1252.6685	1252.7665	0.098	78	25	35	KLFEAGADVFR					Mascot
1439.6519	1439.7733	0.1214	84	36	47	INMSHADHDLMR					Mascot
1452.842	1452.957	0.115	79	393	406	TPIIALSPVVDTAR					Mascot
1570.7893	1570.9152	0.1259	80	347	362	AMPEPTGADAISLAAR					Mascot
1596.8955	1597.0209	0.1254	79	9	24	ILATLGPASGEEAVIR					Mascot
1610.8749	1611.0013	0.1264	78	370	385	LSAIVTYTASGTTGLR					Mascot
1676.9694	1677.0994	0.13	78	59	74	ELGRPIGILADLQGPK	58	100			Mascot
1676.9694	1677.0994	0.13	78	59	74	ELGRPIGILADLQGPK					Mascot
1687.8934	1688.0247	0.1313	78	227	241	LDEIIELSDALMVAR					Mascot
1703.8884	1704.0225	0.1341	79	227	241	LDEIIELSDALMVAR			Oxidation (M)[12]		Mascot
1724.9905	1725.116	0.1255	73	9	25	ILATLGPASGEEAVIRK					Mascot
1763.9537	1764.0923	0.1386	79	242	258	GDLGVEVPLENVPGIQK					Mascot
1915.047	1915.1512	0.1042	54	448	466	VIITAGVPFGTPGATNMLR					Mascot
1931.0419	1931.1768	0.1349	70	448	466	VIITAGVPFGTPGATNMLR			Oxidation (M)[17]		Mascot
1940.0388	1940.1887	0.1499	77	107	123	VFLPHPEILEAVEPGHR	61	100			Mascot
1940.0388	1940.1887	0.1499	77	107	123	VFLPHPEILEAVEPGHR					Mascot
2404.1626	2404.3511	0.1885	78	85	106	VDLVPGQTFTLDNNEALGDETR					Mascot

3	chaperonin GroEL [Brucella suis ATCC 23445]	gi|163844383|ref|YP_001622038.1|	16	122	100	64	64	100	100	.T.	AlDahouk_6214_Z19769-11_T1	389

Peptide Information

Calc. Mass	Obsrv. Mass	± da	± ppm	Start Seq.	End Seq.	Sequence	Ion Score	C. I. %	Modification	Rank	Result Type
707.3947	707.3965	0.0018	3	8	13	FGRTAR					Mascot
719.347	719.3899	0.0429	60	278	284	APGFGDR					Mascot

Calc. Mass	Obsrv. Mass	± da	± ppm	Start Seq.	End Seq.	Sequence	Ion Score	C. I. %	Modification	Rank	Result Type
721.3991	721.4202	0.0211	29	5	10	DVKFGR					Mascot
726.4257	726.4435	0.0178	25	445	451	AIQAPAR					Mascot
762.4178	762.4429	0.0251	33	1	7	MAAKDVK					Mascot
802.4417	802.4631	0.0214	27	363	368	EKLQER					Mascot
855.541	855.606	0.065	76	372	380	LAGGVAVIR					Mascot
882.5268	882.6119	0.0851	96	444	451	RAIQAPAR					Mascot
1233.6508	1233.7408	0.09	73	309	319	LESVTLDMLGR					Mascot
1245.6685	1245.7379	0.0694	56	381	392	VGGATEVEVKEK					Mascot
1455.755	1455.8679	0.1129	78	430	443	GVNADQEAGINIVR					Mascot
1514.7526	1514.8785	0.1259	83	198	210	GYLSPYFVTNPEK					Mascot
1583.8751	1584.0018	0.1267	80	405	421	AAVEEGIVAGGGTALLR	64	100			Mascot
1583.8751	1584.0018	0.1267	80	405	421	AAVEEGIVAGGGTALLR					Mascot
1741.7875	1741.927	0.1395	80	351	364	QQIEETTSDYDREK					Mascot
1853.8585	1854.0057	0.1472	79	182	197	TAETELEVVEGMQFDR					Mascot
2038.9572	2039.1173	0.1601	79	59	75	EVELEDKFENMGAQMLR					Mascot

**Gel Idx/Pos**	185/H12	**Instr./Gel Origin**	AK043/Div_120507	**Process Status**	Analysis Succeeded
**Plate [#] Name**	[1] 1300017700	**Instrument Sample Name**		**Spectra**	4

Rank	Protein	Name	Accession No.	Pep. Count	Protein Score	Protein Score C. I. %	Total Ion Score	Best Ion Score	Best Ion C. I. %	Total Ion C. I. %	Confirmed	Sample Name	Customer sample Name
1	pckA gene product [Brucella suis 1330]		gi|23502937|ref|NP_699064.1|	34	573	100	279	110	100	100	.T.	AlDahouk_6214_Z19769-12_T1	2062

Peptide Information

Calc. Mass	Obsrv. Mass	± da	± ppm	Start Seq.	End Seq.	Sequence	Ion Score	C. I. %	Modification	Rank	Result Type
707.3359	707.3897	0.0538	76	490	495	STWADK					Mascot
713.394	713.4348	0.0408	57	526	532	DAAPAIR					Mascot
756.3747	756.4249	0.0502	66	55	61	TGQHTGR					Mascot
763.446	763.4974	0.0514	67	65	70	DKFVVR					Mascot
838.5145	838.5748	0.0603	72	415	421	KLIAEHK					Mascot
865.4413	865.5035	0.0622	72	496	503	VAYDAQAK					Mascot
923.4832	923.5493	0.0661	72	33	39	LYEETIR					Mascot
961.4949	961.5608	0.0659	69	243	251	TTLSADPTR					Mascot
1079.5844	1079.6602	0.0758	70	33	40	LYEETIRR					Mascot

1185.6011	1185.6868	0.0857	72	405	414	HPSEYGNLLR	68	100		Mascot
1185.6011	1185.6868	0.0857	72	405	414	HPSEYGNLLR				Mascot
1217.6736	1217.7628	0.0892	73	175	185	TETVIAVDLTR				Mascot
1226.5801	1226.674	0.0939	77	516	525	FESHVDHEVK				Mascot
1371.7227	1371.8237	0.101	74	41	54	GEAELSAQGALVAR				Mascot
1436.7532	1436.8677	0.1145	80	20	32	ELSAVFYNLGPAR	102	100		Mascot
1436.8148	1436.8677	0.0529	37	202	214	SVFTALNYILPAK				Mascot
1472.7454	1472.8593	0.1139	77	504	515	KLVDMFVSNFEK			Oxidation (M)[5]	Mascot
1495.725	1495.8405	0.1155	77	323	335	CAYPLDFIPNASK			Carbamidomethyl (C)[1]	Mascot
1527.8237	1527.9408	0.1171	77	40	54	RGEAELSAQGALVAR				Mascot
1553.7594	1553.8844	0.125	80	490	503	STWADKVAYDAQAK				Mascot
1566.856	1566.9716	0.1156	74	187	201	IVLIGGTSYAGEMKK				Mascot
1582.8508	1583.0027	0.1519	96	187	201	IVLIGGTSYAGEMKK			Oxidation (M)[13]	Mascot
1634.8689	1634.9884	0.1195	73	126	138	VITEYAWHSLFIR	110	100		Mascot
1634.8689	1634.9884	0.1195	73	126	138	VITEYAWHSLFIR				Mascot
1678.8282	1678.9553	0.1271	76	280	294	LSAEAEPEIYATTQR				Mascot
1704.8439	1704.9858	0.1419	83	106	121	ELFVQDLIGGADADNK				Mascot
1745.9181	1746.0482	0.1301	75	449	465	ALLAAALDGSLNNAEFR				Mascot
1898.9469	1899.0935	0.1466	77	362	378	LTPAQAMYHFLSGYTAK				Mascot
1914.9419	1915.0717	0.1298	68	362	378	LTPAQAMYHFLSGYTAK			Oxidation (M)[7]	Mascot
1918.0177	1918.1179	0.1002	52	344	361	NIIMLTADAFGVMPPIAK			Oxidation (M)[4]	Mascot
1920.9563	1921.1057	0.1494	78	516	532	FESHVDHEVKDAAPAIR				Mascot
2039.9644	2040.1188	0.1544	76	422	440	VDCWLVNTGWTGGAYG VGK			Carbamidomethyl (C)[3]	Mascot
2159.1091	2159.2717	0.1626	75	106	125	ELFVQDLIGGADADNKIN AR				Mascot
2201.9995	2202.1597	0.1602	73	385	404	GVTEPEATFSTCFGAPF MPR			Carbamidomethyl (C)[12]	Mascot
2217.9944	2218.1719	0.1775	80	385	404	GVTEPEATFSTCFGAPF MPR			Carbamidomethyl (C)[12], Oxidation (M)[18]	Mascot
2554.3186	2554.5215	0.2029	79	466	489	IDPNFGFAVPVEVPGVES SILDPR				Mascot
2795.2153	2795.4282	0.2129	76	252	276	TLIGDDEHGWGEHGVFN FEGGCYAK			Carbamidomethyl (C)[22]	Mascot
3045.6328	3045.8308	0.198	65	139	165	NLLIRPSQEALASYVPEM TIIDLPSFK				Mascot
3180.5081	3180.7688	0.2607	82	295	322	FGTVLENVVLDENRQPD FDDGSLTENTR				Mascot

2	chaperonin GroEL [Brucella suis ATCC 23445]	gi|163844383|ref|YP_001622038.1|	20	107	100	.T.	AlDahouk_6214_Z19769-12_T1	2062

Peptide Information

Calc. Mass	Obsrv. Mass	± da	± ppm	Start Seq.	End Seq.	Sequence	Ion Score	C. I. %	Modification	Rank	Result Type
707.3947	707.3897	-0.005	-7	8	13	FGRTAR					Mascot
719.347	719.3868	0.0398	55	278	284	APGFGDR					Mascot
726.4257	726.4643	0.0386	53	445	451	AIQAPAR					Mascot
855.541	855.603	0.062	72	372	380	LAGGVAVIR					Mascot
875.4482	875.5123	0.0641	73	278	285	APGFGDRR					Mascot
882.5268	882.5948	0.068	77	444	451	RAIQAPAR					Mascot
1233.6508	1233.7372	0.0864	70	309	319	LESVTLDMLGR					Mascot
1245.6685	1245.718	0.0495	40	381	392	VGGATEVEVKEK					Mascot
1249.6456	1249.7391	0.0935	75	309	319	LESVTLDMLGR			Oxidation (M)[8]		Mascot
1344.7004	1344.7865	0.0861	64	169	181	VGNEGVITVEEAK					Mascot
1373.7028	1373.8206	0.1178	86	106	118	AVAAGMNPMDLKR					Mascot
1389.6978	1389.8053	0.1075	77	106	118	AVAAGMNPMDLKR			Oxidation (M)[6]		Mascot
1405.6926	1405.8079	0.1153	82	106	118	AVAAGMNPMDLKR			Oxidation (M)[6,9]		Mascot
1455.755	1455.8617	0.1067	73	430	443	GVNADQEAGINIVR					Mascot
1514.7526	1514.8693	0.1167	77	198	210	GYLSPYFVTNPEK					Mascot
1582.905	1583.0027	0.0977	62	119	133	GIDLAVNEVVAELLK					Mascot
1583.8751	1583.9952	0.1201	76	405	421	AAVEEGIVAGGGTALLR					Mascot
1711	1711.1268	0.1268	74	119	134	GIDLAVNEVVAELLKK					Mascot
1741.7875	1741.9231	0.1356	78	351	364	QQIEETTSDYDREK					Mascot
1759.8934	1760.0338	0.1404	80	211	225	MVADLEDAYILLHEK					Mascot
1775.8884	1776.0276	0.1392	78	211	225	MVADLEDAYILLHEK			Oxidation (M)[1]		Mascot
1853.8585	1854.0009	0.1424	77	182	197	TAETELEVVEGMQFDR					Mascot
1869.8535	1870.0138	0.1603	86	182	197	TAETELEVVEGMQFDR			Oxidation (M)[12]		Mascot
2038.9572	2039.1121	0.1549	76	59	75	EVELEDKFENMGAQMLR					Mascot
2054.9521	2055.1262	0.1741	85	59	75	EVELEDKFENMGAQMLR			Oxidation (M)[11]		Mascot
2070.947	2071.1201	0.1731	84	59	75	EVELEDKFENMGAQMLR			Oxidation (M)[11,15]		Mascot
2419.1946	2419.3916	0.197	81	81	105	TNDTAGDGTTTATVLGQ AIVQEGAK					Mascot

3	unnamed protein product [Brucella suis 1330]	gi|23501035|ref|NP_697162.1|	13	71	99.889	.T.	AlDahouk_6214_Z19769-12_T1	2062

Peptide Information

Calc. Mass	Obsrv. Mass	± da	± ppm	Start Seq.	End Seq.	Sequence	Ion Score	C. I. %	Modification	Rank	Result Type

Calc. Mass	Obsrv. Mass	± da	± ppm	Start Seq.	End Seq.	Sequence	Ion Score	C. I. %	Modification	Rank	Result Type
775.4824	775.535	0.0526	68	24	29	LFNLLR					Mascot
834.4104	834.4352	0.0248	30	275	281	SWESLGR					Mascot
951.4894	951.5582	0.0688	72	56	64	SIGFDASVR					Mascot
1037.5051	1037.5841	0.079	76	447	454	YELNSFHK					Mascot
1173.615	1173.7017	0.0867	74	46	55	AAEWLVEDLK					Mascot
1786.9585	1787.0964	0.1379	77	282	299	TAESFLGPIGLSIPAGEK					Mascot
1930.8633	1931.0555	0.1922	100	130	146	GTSDDKGQLMTFVEACR			Carbamidomethyl (C)[16], Oxidation (M)[10]		Mascot
2066.0818	2066.2322	0.1504	73	225	244	DLHSGFFGGAAANPIHIL TK					Mascot
2193.1326	2193.3064	0.1738	79	256	274	ITIPDFYEGVEETPTQILK					Mascot
2479.2839	2479.4651	0.1812	73	221	244	AADRDLHSGFFGGAAAN PIHILTK					Mascot
2561.2375	2561.4214	0.1839	72	187	210	ADVALVCDTAMWDAETP AISVGLR			Carbamidomethyl (C)[7]		Mascot
2649.3406	2649.5251	0.1845	70	159	182	VTLLFEGEEESGSPSLKP FLEANR					Mascot
2865.478	2865.6606	0.1826	64	302	328	SVLELTWARPTAEVNGII GGYTGEGFK					Mascot

Gel Idx/Pos	186/H13	Instr./Gel Origin	AK043/Div_120507	Process Status	Analysis Succeeded
Plate [#] Name	[1] 1300017700	Instrument Sample Name		Spectra	4

Rank	Protein Name	Accession No.	Pep. Count	Protein Score	Protein Score C. I. %	Total Ion Score	Best Ion Score	Best Ion C. I. %	Total Ion C. I. %	Confirmed	Sample Name	Customer sample Name
1	trx-1 gene product [Brucella suis 1330]	gi|23502953|ref|NP_699080.1|	3	95	100	77	77	100	100	.T.	AlDahouk_6214_Z19769-13_T1	2082

Peptide Information

Calc. Mass	Obsrv. Mass	± da	± ppm	Start Seq.	End Seq.	Sequence	Ion Score	C. I. %	Modification	Rank	Result Type
745.4243	745.4666	0.0423	57	98	103	LADWIK					Mascot
1065.6013	1065.6664	0.0651	61	73	81	SIPTLLMFK			Oxidation (M)[7]		Mascot
1771.8973	1772.0375	0.1402	79	57	72	VNIDENPELAAQFGVR	77	100			Mascot
1771.8973	1772.0375	0.1402	79	57	72	VNIDENPELAAQFGVR					Mascot

Gel Idx/Pos	187/H14	Instr./Gel Origin	AK043/Div_120507	Process Status	Analysis Succeeded
Plate [#] Name	[1] 1300017700	Instrument Sample Name		Spectra	4

Rank	Protein Name	Accession No.	Pep. Count	Protein Score	Protein Score C. I. %	Total Ion Score	Best Ion Score	Best Ion C. I. %	Total Ion C. I. %	Confirmed	Sample Name	Customer sample Name
1	trx-1 gene product [Brucella suis 1330]	gi|23502953|ref|N	3	69	99.84	51	51	99.996	99.996	.T.	AlDahouk_6	1936

		P_699080.1										214_Z19769-14_T1	

Peptide Information

Calc. Mass	Obsrv. Mass	± da	± ppm	Start Seq.	End Seq.	Sequence	Ion Score	C. I. %	Modification	Rank	Result Type
745.4243	745.4351	0.0108	14	98	103	LADWIK					Mascot
1065.6013	1065.6335	0.0322	30	73	81	SIPTLLMFK			Oxidation (M)[7]		Mascot
1771.8973	1772.0354	0.1381	78	57	72	VNIDENPELAAQFGVR	51	99.996			Mascot
1771.8973	1772.0354	0.1381	78	57	72	VNIDENPELAAQFGVR					Mascot

2	aspartyl/glutamyl-tRNA amidotransferase subunit C [Brucella suis ATCC 23445]	gi|163844747|ref|YP_001622402.1|	4	52	92.505	26	26	98.32	98.32	.T.	AlDahouk_6214_Z19769-14_T1	1936

Peptide Information

Calc. Mass	Obsrv. Mass	± da	± ppm	Start Seq.	End Seq.	Sequence	Ion Score	C. I. %	Modification	Rank	Result Type
822.5056	822.5286	0.023	28	10	16	RVAHLAR					Mascot
1004.5734	1004.6529	0.0795	79	2	10	SVDISTVKR					Mascot
1104.5167	1104.6041	0.0874	79	17	26	IAVSEDDAER	26	98.32			Mascot
1104.5167	1104.6041	0.0874	79	17	26	IAVSEDDAER					Mascot
2601.3557	2601.575	0.2193	84	67	92	VTDGGIAAAVVANAPVTEDNFFVVPK					Mascot

Gel Idx/Pos	188/H15	Instr./Gel Origin	AK043/Div_120507	Process Status	Analysis Succeeded
Plate [#] Name	[1] 1300017700	Instrument Sample Name		Spectra	4

Rank	Protein Name	Accession No.	Pep. Count	Protein Score	Protein Score C. I. %	Total Ion Score	Best Ion Score	Best Ion C. I. %	Total Ion C. I. %	Confirmed	Sample Name	Customer sample Name
1	unnamed protein product [Brucella suis 1330]	gi|23501035|ref|NP_697162.1|	23	277	100	100	100	100	100	.T.	AlDahouk_6214_Z19769-15_T1	418

Peptide Information

Calc. Mass	Obsrv. Mass	± da	± ppm	Start Seq.	End Seq.	Sequence	Ion Score	C. I. %	Modification	Rank	Result Type
763.4196	763.4885	0.0689	90	2	8	STLSLDK					Mascot
775.4824	775.5366	0.0542	70	24	29	LFNLLR					Mascot
791.4522	791.4865	0.0343	43	352	357	IREAFR					Mascot
834.4104	834.4697	0.0593	71	275	281	SWESLGR					Mascot

951.4894	951.5596	0.0702	74	56	64	SIGFDASVR					Mascot
1037.5051	1037.582	0.0769	74	447	454	YELNSFHK					Mascot
1056.6299	1056.7056	0.0757	72	211	220	GLVGEEIVIK					Mascot
1063.6259	1063.7013	0.0754	71	343	351	LVHKQDPVK					Mascot
1173.615	1173.7014	0.0864	74	46	55	AAEWLVEDLK					Mascot
1239.6328	1239.7281	0.0953	77	245	255	ILADLHDETGR	100	100			Mascot
1239.6328	1239.7281	0.0953	77	245	255	ILADLHDETGR					Mascot
1250.6852	1250.7726	0.0874	70	9	19	VLNHLDANLNK					Mascot
1301.71	1301.8059	0.0959	74	45	55	KAAEWLVEDLK					Mascot
1786.9585	1787.0988	0.1403	79	282	299	TAESFLGPIGLSIPAGEK					Mascot
1914.8684	1915.0175	0.1491	78	130	146	GTSDDKGQLMTFVEACR			Carbamidomethyl (C)[16]		Mascot
1930.8633	1931.0193	0.156	81	130	146	GTSDDKGQLMTFVEACR			Carbamidomethyl (C)[16], Oxidation (M)[10]		Mascot
2066.0818	2066.2402	0.1584	77	225	244	DLHSGFFGGAAANPIHIL TK					Mascot
2193.1326	2193.3035	0.1709	78	256	274	ITIPDFYEGVEETPTQILK					Mascot
2479.2839	2479.4692	0.1853	75	221	244	AADRDLHSGFFGGAAAN PIHILTK					Mascot
2561.2375	2561.4263	0.1888	74	187	210	ADVALVCDTAMWDAETP AISVGLR			Carbamidomethyl (C)[7]		Mascot
2577.2324	2577.428	0.1956	76	187	210	ADVALVCDTAMWDAETP AISVGLR			Carbamidomethyl (C)[7], Oxidation (M)[11]		Mascot
2649.3406	2649.5349	0.1943	73	159	182	VTLLFEGEEESGSPSLKP FLEANR					Mascot
2865.478	2865.6873	0.2093	73	302	328	SVLELTWARPTAEVNGII GGYTGEGFK					Mascot
3104.5146	3104.7566	0.242	78	364	392	VPADCSVEFHPHGGSPAI QLPYDSPLVSK			Carbamidomethyl (C)[5]		Mascot
3147.6208	3147.8413	0.2205	70	159	186	VTLLFEGEEESGSPSLKP FLEANRQELK					Mascot
3389.6582	3389.8909	0.2327	69	362	392	ERVPADCSVEFHPHGGS PAIQLPYDSPLVSK			Carbamidomethyl (C)[7]		Mascot

2	chaperonin GroEL [Brucella suis ATCC 23445]	gi|163844383|ref|YP_001622038.1|	23	273	100	134	134	100	100	.T.	AlDahouk_6214_Z19769-15_T1	418

Peptide Information

Calc. Mass	Obsrv. Mass	± da	± ppm	Start Seq.	End Seq.	Sequence	Ion Score	C. I. %	Modification	Rank	Result Type
707.3947	707.39	-0.0047	-7	8	13	FGRTAR					Mascot
719.347	719.387	0.04	56	278	284	APGFGDR					Mascot
726.4257	726.4641	0.0384	53	445	451	AIQAPAR					Mascot
855.541	855.6058	0.0648	76	372	380	LAGGVAVIR					Mascot
875.4482	875.5114	0.0632	72	278	285	APGFGDRR					Mascot

882.5268	882.5914	0.0646	73	444	451	RAIQAPAR				Mascot
1000.5673	1000.6361	0.0688	69	19	28	GVDILADAVK				Mascot
1233.6508	1233.739	0.0882	71	309	319	LESVTLDMLGR				Mascot
1245.6685	1245.7211	0.0526	42	381	392	VGGATEVEVKEK				Mascot
1249.6456	1249.7372	0.0916	73	309	319	LESVTLDMLGR			Oxidation (M)[8]	Mascot
1344.7004	1344.8	0.0996	74	169	181	VGNEGVITVEEAK				Mascot
1373.7028	1373.8198	0.117	85	106	118	AVAAGMNPMDLKR				Mascot
1389.6978	1389.8059	0.1081	78	106	118	AVAAGMNPMDLKR			Oxidation (M)[6]	Mascot
1405.6926	1405.803	0.1104	79	106	118	AVAAGMNPMDLKR			Oxidation (M)[6,9]	Mascot
1455.755	1455.865	0.11	76	430	443	GVNADQEAGINIVR				Mascot
1514.7526	1514.8699	0.1173	77	198	210	GYLSPYFVTNPEK				Mascot
1582.905	1583.0243	0.1193	75	119	133	GIDLAVNEVVAELLK				Mascot
1583.8751	1583.9979	0.1228	78	405	421	AAVEEGIVAGGGTALLR	134	100		Mascot
1583.8751	1583.9979	0.1228	78	405	421	AAVEEGIVAGGGTALLR				Mascot
1616.849	1616.9716	0.1226	76	452	467	QITTNAGEEASVIVGK				Mascot
1711	1711.1301	0.1301	76	119	134	GIDLAVNEVVAELLKK				Mascot
1741.7875	1741.9241	0.1366	78	351	364	QQIEETTSDYDREK				Mascot
1759.8934	1760.0309	0.1375	78	211	225	MVADLEDAYILLHEK				Mascot
1775.8884	1776.0291	0.1407	79	211	225	MVADLEDAYILLHEK			Oxidation (M)[1]	Mascot
1853.8585	1854.0005	0.142	77	182	197	TAETELEVVEGMQFDR				Mascot
1869.8535	1870.0054	0.1519	81	182	197	TAETELEVVEGMQFDR			Oxidation (M)[12]	Mascot
2038.9572	2039.1104	0.1532	75	59	75	EVELEDKFENMGAQMLR				Mascot
2054.9521	2055.1221	0.17	83	59	75	EVELEDKFENMGAQMLR			Oxidation (M)[11]	Mascot
2070.947	2071.127	0.18	87	59	75	EVELEDKFENMGAQMLR			Oxidation (M)[11,15]	Mascot
2419.1946	2419.3945	0.1999	83	81	105	TNDTAGDGTTTATVLGQ AIVQEGAK				Mascot
2445.2466	2445.438	0.1914	78	137	160	KINTSEEVAQVGTISANG EAEIGK				Mascot

**Gel Idx/Pos**	189/H16	**Instr./Gel Origin**	AK043/Div_120507	**Process Status**	Analysis Succeeded
**Plate [#] Name**	[1] 1300017700	**Instrument Sample Name**		**Spectra**	4

**Rank**	**Protein**	**Name**	**Accession No.**	**Pep. Count**	**Protein Score**	**Protein Score C. I. %**	**Total Ion Score**	**Best Ion Score**	**Best Ion C. I. %**	**Total Ion C. I. %**	**Confirmed**	**Sample Name**	**Customer sample Name**
1	pckA gene product [Brucella suis 1330]		gi|23502937|ref|NP_699064.1|	22	395	100	249	97	100	100	.T.	AlDahouk_6214_Z19769-16_T1	374

Peptide Information

**Deing_6268, Wollny_6172,
AlDahouk_6214\Div_120507\AlDahouk_6214**

Calc. Mass	Obsrv. Mass	± da	± ppm	Start Seq.	End Seq.	Sequence	Ion Score	C. I. %	Modification	Rank	Result Type
710.4195	710.4171	-0.0024	-3	416	421	LIAEHK					Mascot
713.394	713.4277	0.0337	47	526	532	DAAPAIR					Mascot
756.3747	756.4259	0.0512	68	55	61	TGQHTGR					Mascot
758.4155	758.4453	0.0298	39	336	343	SGKGGQPK					Mascot
763.446	763.4966	0.0506	66	65	70	DKFVVR					Mascot
923.4832	923.5527	0.0695	75	33	39	LYEETIR					Mascot
1185.6011	1185.6934	0.0923	78	405	414	HPSEYGNLLR	65	100			Mascot
1185.6011	1185.6934	0.0923	78	405	414	HPSEYGNLLR					Mascot
1217.6736	1217.7617	0.0881	72	175	185	TETVIAVDLTR					Mascot
1226.5801	1226.6753	0.0952	78	516	525	FESHVDHEVK					Mascot
1436.7532	1436.8728	0.1196	83	20	32	ELSAVFYNLGPAR	88	100			Mascot
1436.8148	1436.8728	0.058	40	202	214	SVFTALNYILPAK					Mascot
1495.725	1495.8464	0.1214	81	323	335	CAYPLDFIPNASK			Carbamidomethyl (C)[1]		Mascot
1527.8237	1527.9458	0.1221	80	40	54	RGEAELSAQGALVAR					Mascot
1634.8689	1634.9955	0.1266	77	126	138	VITEYAWHSLFIR	97	100			Mascot
1634.8689	1634.9955	0.1266	77	126	138	VITEYAWHSLFIR					Mascot
1678.8282	1678.9604	0.1322	79	280	294	LSAEAEPEIYATTQR					Mascot
1745.9181	1746.0508	0.1327	76	449	465	ALLAAALDGSLNNAEFR					Mascot
1898.9469	1899.0896	0.1427	75	362	378	LTPAQAMYHFLSGYTAK					Mascot
1914.9419	1915.0532	0.1113	58	362	378	LTPAQAMYHFLSGYTAK			Oxidation (M)[7]		Mascot
1920.9563	1921.1045	0.1482	77	516	532	FESHVDHEVKDAAPAIR					Mascot
2159.1091	2159.2715	0.1624	75	106	125	ELFVQDLIGGADADNKIN AR					Mascot
2201.9995	2202.1658	0.1663	76	385	404	GVTEPEATFSTCFGAPF MPR			Carbamidomethyl (C)[12]		Mascot
2217.9944	2218.1709	0.1765	80	385	404	GVTEPEATFSTCFGAPF MPR			Carbamidomethyl (C)[12], Oxidation (M)[18]		Mascot
2554.3186	2554.5098	0.1912	75	466	489	IDPNFGFAVPVEVPGVES SILDPR					Mascot
2795.2153	2795.4167	0.2014	72	252	276	TLIGDDEHGWGEHGVFN FEGGCYAK			Carbamidomethyl (C)[22]		Mascot

2 chaperonin GroEL [Brucella suis ATCC 23445] gi|163844383|ref|YP_001622038.1| 19 84 99.995 .T. AlDahouk_6214_Z19769-16_T1 374

Peptide Information

Calc. Mass	Obsrv. Mass	± da	± ppm	Start Seq.	End Seq.	Sequence	Ion Score	C. I. %	Modification	Rank	Result Type

707.3947	707.4076	0.0129	18	8	13	FGRTAR					Mascot
719.347	719.3865	0.0395	55	278	284	APGFGDR					Mascot
721.3991	721.4199	0.0208	29	5	10	DVKFGR					Mascot
726.4257	726.4658	0.0401	55	445	451	AIQAPAR					Mascot
802.4417	802.4821	0.0404	50	363	368	EKLQER					Mascot
855.541	855.6052	0.0642	75	372	380	LAGGVAVIR					Mascot
875.4482	875.5119	0.0637	73	278	285	APGFGDRR					Mascot
882.5268	882.5971	0.0703	80	444	451	RAIQAPAR					Mascot
974.4901	974.531	0.0409	42	396	404	VDDALNATR					Mascot
1233.6508	1233.7413	0.0905	73	309	319	LESVTLDMLGR					Mascot
1245.6685	1245.7183	0.0498	40	381	392	VGGATEVEVKEK					Mascot
1455.755	1455.8627	0.1077	74	430	443	GVNADQEAGINIVR					Mascot
1514.7526	1514.8754	0.1228	81	198	210	GYLSPYFVTNPEK					Mascot
1582.905	1583.0135	0.1085	69	119	133	GIDLAVNEVVAELLK					Mascot
1583.8751	1583.9972	0.1221	77	405	421	AAVEEGIVAGGGTALLR					Mascot
1741.7875	1741.929	0.1415	81	351	364	QQIEETTSDYDREK					Mascot
1759.8934	1760.0389	0.1455	83	211	225	MVADLEDAYILLHEK					Mascot
1853.8585	1854.0038	0.1453	78	182	197	TAETELEVVEGMQFDR					Mascot
1869.8535	1870.0173	0.1638	88	182	197	TAETELEVVEGMQFDR			Oxidation (M)[12]		Mascot
2038.9572	2039.125	0.1678	82	59	75	EVELEDKFENMGAQMLR					Mascot

3 unnamed protein product [Brucella suis 1330] gi|23501913|ref|NP_698040.1| 16 75 99.955 .T. AlDahouk_6214_Z19769-16_T1 374

Peptide Information

Calc. Mass	Obsrv. Mass	± da	± ppm	Start Seq.	End Seq.	Sequence	Ion Score	C. I. %	Modification	Rank	Result Type
715.4712	715.4319	-0.0393	-55	334	340	ILVAATK					Mascot
731.3934	731.423	0.0296	40	183	189	TVAPSEK					Mascot
746.3791	746.4489	0.0698	94	208	214	AGKADER					Mascot
802.4781	802.4821	0.004	5	32	39	LGVTGLSR					Mascot
829.4202	829.4842	0.064	77	261	266	RYEAYK					Mascot
855.5522	855.6052	0.053	62	352	358	LQAIVRR					Mascot
974.44	974.531	0.091	93	201	207	CFTDYLR			Carbamidomethyl (C)[1]		Mascot
1032.5836	1032.6602	0.0766	74	476	484	ALQFLIGDR					Mascot
1074.632	1074.6719	0.0399	37	449	456	FVRFRPPR					Mascot

1156.6514	1156.7366	0.0852	74	267	275	TYVVKPFFR	Mascot
1353.6044	1353.7128	0.1084	80	341	351	ADHLHHESHDR	Mascot
1393.7798	1393.8665	0.0867	62	460	472	TAEGVTLSLPHIR	Mascot
1496.738	1496.8519	0.1139	76	113	125	LTIEYETASAWGR	Mascot
1719.9653	1720.0848	0.1195	69	43	58	TVFISALVHNLVHGGR	Mascot
1749.8442	1750.011	0.1668	95	86	99	FQYEEHLSALIDER	Mascot
2125.9937	2126.1594	0.1657	78	153	171	TYAQFSADSFALANEPTH R	Mascot

Gel Idx/Pos	190/H17	Instr./Gel Origin	AK043/Div_120507	Process Status	Analysis Succeeded
Plate [#] Name	[1] 1300017700	Instrument Sample Name		Spectra	4

Rank	Protein	Name	Accession No.	Pep. Count	Protein Score	Protein Score C. I. %	Total Ion Score	Best Ion Score	Best Ion C. I. %	Total Ion C. I. %	Confirmed	Sample Name	Customer sample Name
1	chaperonin GroEL [Brucella suis ATCC 23445]		gi|163844383|ref|YP_001622038.1|	24	433	100	279	153	100	100	.T.	AlDahouk_6214_Z19769-17_T1	380

Peptide Information

Calc. Mass	Obsrv. Mass	± da	± ppm	Start Seq.	End Seq.	Sequence	Ion Score	C. I. %	Modification	Rank	Result Type
707.3947	707.3848	-0.0099	-14	8	13	FGRTAR					Mascot
719.347	719.381	0.034	47	278	284	APGFGDR					Mascot
726.4257	726.4581	0.0324	45	445	451	AIQAPAR					Mascot
855.541	855.5991	0.0581	68	372	380	LAGGVAVIR					Mascot
875.4482	875.5052	0.057	65	278	285	APGFGDRR					Mascot
882.5268	882.5853	0.0585	66	444	451	RAIQAPAR					Mascot
974.4901	974.5486	0.0585	60	396	404	VDDALNATR					Mascot
1233.6508	1233.7323	0.0815	66	309	319	LESVTLDMLGR					Mascot
1245.6685	1245.7092	0.0407	33	381	392	VGGATEVEVKEK					Mascot
1249.6456	1249.7455	0.0999	80	309	319	LESVTLDMLGR			Oxidation (M)[8]		Mascot
1373.7028	1373.8003	0.0975	71	106	118	AVAAGMNPMDLKR					Mascot
1389.6978	1389.7983	0.1005	72	106	118	AVAAGMNPMDLKR			Oxidation (M)[6]		Mascot
1455.755	1455.8525	0.0975	67	430	443	GVNADQEAGINIVR	126	100			Mascot
1455.755	1455.8525	0.0975	67	430	443	GVNADQEAGINIVR					Mascot
1514.7526	1514.8638	0.1112	73	198	210	GYLSPYFVTNPEK					Mascot
1582.905	1583.0154	0.1104	70	119	133	GIDLAVNEVVAELLK					Mascot
1583.8751	1583.9865	0.1114	70	405	421	AAVEEGIVAGGGTALLR	153	100			Mascot

1583.8751	1583.9865	0.1114	70	405	421	AAVEEGIVAGGGTALLR				Mascot
1711	1711.1198	0.1198	70	119	134	GIDLAVNEVVAELLKK				Mascot
1739.0061	1739.1141	0.108	62	118	133	RGIDLAVNEVVAELLK				Mascot
1741.7875	1741.9216	0.1341	77	351	364	QQIEETTSDYDREK				Mascot
1759.8934	1760.0256	0.1322	75	211	225	MVADLEDAYILLHEK				Mascot
1775.8884	1776.021	0.1326	75	211	225	MVADLEDAYILLHEK			Oxidation (M)[1]	Mascot
1853.8585	1853.9875	0.129	70	182	197	TAETELEVVEGMQFDR				Mascot
1869.8535	1870.0095	0.156	83	182	197	TAETELEVVEGMQFDR			Oxidation (M)[12]	Mascot
2038.9572	2039.106	0.1488	73	59	75	EVELEDKFENMGAQMLR				Mascot
2054.9521	2055.1174	0.1653	80	59	75	EVELEDKFENMGAQMLR			Oxidation (M)[11]	Mascot
2070.947	2071.1111	0.1641	79	59	75	EVELEDKFENMGAQMLR			Oxidation (M)[11,15]	Mascot
2286.2261	2286.3872	0.1611	70	287	308	AMLEDIAILTGGQVISEDL GIK				Mascot
2419.1946	2419.3889	0.1943	80	81	105	TNDTAGDGTTTATVLGQ AIVQEGAK				Mascot
2445.2466	2445.4326	0.186	76	137	160	KINTSEEVAQVGTISANG EAEIGK				Mascot
3329.6423	3329.917	0.2747	83	468	498	ILENTSETFGYNTANGEY GDLISLGIVDPVK				Mascot

2	unnamed protein product [Brucella suis 1330]	gi|23501576|ref|NP_697703.1|	17	216	100	115	115	100	100	.T.	AlDahouk_6214_Z19769-17_T1	380

Peptide Information

Calc. Mass	Obsrv. Mass	± da	± ppm	Start Seq.	End Seq.	Sequence	Ion Score	C. I. %	Modification	Rank	Result Type
815.4046	815.451	0.0464	57	134	139	SYKFDR					Mascot
855.5661	855.5991	0.033	39	18	26	GVAVVLVAK					Mascot
960.4785	960.5453	0.0668	70	85	92	LGNDDWLK					Mascot
1169.6135	1169.6918	0.0783	67	243	252	LVVMEWHGAK					Mascot
1185.6085	1185.6808	0.0723	61	243	252	LVVMEWHGAK			Oxidation (M)[4]		Mascot
1249.6787	1249.7455	0.0668	53	47	59	IADISGFTGALGK					Mascot
1255.6681	1255.7546	0.0865	69	438	449	YGGSITAAQFLK					Mascot
1343.6737	1343.7656	0.0919	68	157	168	ITICVADTHSAR			Carbamidomethyl (C)[4]		Mascot
1390.7073	1390.802	0.0947	68	93	106	IGGAAFSQIGNAER	115	100			Mascot
1390.7073	1390.802	0.0947	68	93	106	IGGAAFSQIGNAER					Mascot
1500.7805	1500.8833	0.1028	68	350	362	LVLADALHYTNDR					Mascot
1650.8486	1650.962	0.1134	69	4	17	RPSISFSEFEVPQK					Mascot
1675.865	1675.9812	0.1162	69	170	185	TFEVAEAVADGVIQAR					Mascot
1779.0057	1779.1147	0.109	61	225	242	LGMGALLGVAQGSVRPP					Mascot

						R					
1795.0006	1795.1274	0.1268	71	225	242	LGMGALLGVAQGSVRPPR			Oxidation (M)[3]		Mascot
1803.96	1804.0795	0.1195	66	169	185	KTFEVAEAVADGVIQAR					Mascot
1806.8949	1807.0497	0.1548	86	285	303	GDMGGAAAVTGLMRALAGR			Oxidation (M)[3,13]		Mascot
1865.9756	1866.1028	0.1272	68	2	17	SKRPSISFSEFEVPQK					Mascot
2653.3718	2653.5588	0.187	70	186	209	NLVNEPANILGPVEFAEEAEKLEK					Mascot
2740.48	2740.6558	0.1758	64	107	133	VTLTLALPETTIAGDEAADVALGMVLR					Mascot

3	pckA gene product [Brucella suis 1330]	gi|23502937|ref|NP_699064.1|	17	92	100	.T.	AlDahouk_6214_Z19769-17_T1	380

Peptide Information

Calc. Mass	Obsrv. Mass	± da	± ppm	Start Seq.	End Seq.	Sequence	Ion Score	C. I. %	Modification	Rank	Result Type
707.3359	707.3848	0.0489	69	490	495	STWADK					Mascot
713.394	713.3989	0.0049	7	526	532	DAAPAIR					Mascot
756.3747	756.418	0.0433	57	55	61	TGQHTGR					Mascot
763.446	763.4883	0.0423	55	65	70	DKFVVR					Mascot
923.4832	923.5383	0.0551	60	33	39	LYEETIR					Mascot
1185.6011	1185.6808	0.0797	67	405	414	HPSEYGNLLR					Mascot
1217.6736	1217.7416	0.068	56	175	185	TETVIAVDLTR					Mascot
1436.8148	1436.8572	0.0424	30	202	214	SVFTALNYILPAK					Mascot
1527.8237	1527.9318	0.1081	71	40	54	RGEAELSAQGALVAR					Mascot
1634.8689	1634.9762	0.1073	66	126	138	VITEYAWHSLFIR					Mascot
1678.8282	1678.9562	0.128	76	280	294	LSAEAEPEIYATTQR					Mascot
1745.9181	1746.0325	0.1144	66	449	465	ALLAAALDGSLNNAEFR					Mascot
1914.9419	1915.0223	0.0804	42	362	378	LTPAQAMYHFLSGYTAK			Oxidation (M)[7]		Mascot
1920.9563	1921.0887	0.1324	69	516	532	FESHVDHEVKDAAPAIR					Mascot
2159.1091	2159.2507	0.1416	66	106	125	ELFVQDLIGGADADNKINAR					Mascot
2201.9995	2202.1411	0.1416	64	385	404	GVTEPEATFSTCFGAPFMPR			Carbamidomethyl (C)[12]		Mascot
2554.3186	2554.4973	0.1787	70	466	489	IDPNFGFAVPVEVPGVESSILDPR					Mascot

4	unnamed protein product [Brucella suis 1330]	gi|23501035|ref|NP_697162.1|	12	58	97.684	.T.	AlDahouk_6214_Z19769-17_T1	380

Peptide Information

Calc. Mass	Obsrv. Mass	± da	± ppm	Start	End	Sequence	Ion	C. I. %	Modification	Rank	Result Type

				Seq.	Seq.		Score			
763.4196	763.4883	0.0687	90	2	8	STLSLDK				Mascot
775.4824	775.5278	0.0454	59	24	29	LFNLLR				Mascot
834.4104	834.4622	0.0518	62	275	281	SWESLGR				Mascot
951.4894	951.5519	0.0625	66	56	64	SIGFDASVR				Mascot
960.5359	960.5453	0.0094	10	329	338	TVIAAEASAK				Mascot
1239.6328	1239.7183	0.0855	69	245	255	ILADLHDETGR				Mascot
1301.71	1301.7806	0.0706	54	45	55	KAAEWLVEDLK				Mascot
1786.9585	1787.0823	0.1238	69	282	299	TAESFLGPIGLSIPAGEK				Mascot
1914.8684	1915.0223	0.1539	80	130	146	GTSDDKGQLMTFVEACR			Carbamidomethyl (C)[16]	Mascot
2066.0818	2066.2109	0.1291	62	225	244	DLHSGFFGGAAANPIHIL TK				Mascot
2479.2839	2479.457	0.1731	70	221	244	AADRDLHSGFFGGAAAN PIHILTK				Mascot
2649.3406	2649.512	0.1714	65	159	182	VTLLFEGEEESGSPSLKP FLEANR				Mascot

Gel Idx/Pos	191/H18	Instr./Gel Origin	AK043/Div_120507	Process Status	Analysis Succeeded
Plate [#] Name	[1] 1300017700	Instrument Sample Name		Spectra	4

Rank	Protein	Name	Accession No.	Pep. Count	Protein Score	Protein Score C. I. %	Total Ion Score	Best Ion Score	Best Ion C. I. %	Total Ion C. I. %	Confirmed	Sample Name	Customer sample Name
1	pckA gene product [Brucella suis 1330]		gi|23502937|ref|NP_699064.1|	26	386	100	196	106	100	100	.T.	AlDahouk_6214_Z19769-18_T1	426

Peptide Information

Calc. Mass	Obsrv. Mass	± da	± ppm	Start Seq.	End Seq.	Sequence	Ion Score	C. I. %	Modification	Rank	Result Type
707.3359	707.4028	0.0669	95	490	495	STWADK					Mascot
710.4195	710.4265	0.007	10	416	421	LIAEHK					Mascot
713.394	713.424	0.03	42	526	532	DAAPAIR					Mascot
756.3747	756.4221	0.0474	63	55	61	TGQHTGR					Mascot
763.446	763.4937	0.0477	62	65	70	DKFVVR					Mascot
865.4413	865.5026	0.0613	71	496	503	VAYDAQAK					Mascot
923.4832	923.5459	0.0627	68	33	39	LYEETIR					Mascot
1185.6011	1185.6849	0.0838	71	405	414	HPSEYGNLLR					Mascot
1217.6736	1217.7592	0.0856	70	175	185	TETVIAVDLTR					Mascot
1226.5801	1226.6692	0.0891	73	516	525	FESHVDHEVK					Mascot

Calc. Mass	Obsrv. Mass	± da	± ppm	Start Seq.	End Seq.	Sequence	Ion Score	C. I. %	Modification	Rank	Result Type
1344.6504	1344.7805	0.1301	97	505	515	LVDMFVSNFEK			Oxidation (M)[4]		Mascot
1436.7532	1436.8638	0.1106	77	20	32	ELSAVFYNLGPAR	91	100			Mascot
1436.8148	1436.8638	0.049	34	202	214	SVFTALNYILPAK					Mascot
1495.725	1495.8331	0.1081	72	323	335	CAYPLDFIPNASK			Carbamidomethyl (C)[1]		Mascot
1527.8237	1527.9373	0.1136	74	40	54	RGEAELSAQGALVAR					Mascot
1604.8279	1604.9518	0.1239	77	295	308	FGTVLENVVLDENR					Mascot
1634.8689	1634.984	0.1151	70	126	138	VITEYAWHSLFIR	106	100			Mascot
1634.8689	1634.984	0.1151	70	126	138	VITEYAWHSLFIR					Mascot
1678.8282	1678.9537	0.1255	75	280	294	LSAEAEPEIYATTQR					Mascot
1745.9181	1746.0425	0.1244	71	449	465	ALLAAALDGSLNNAEFR					Mascot
1898.9469	1899.0784	0.1315	69	362	378	LTPAQAMYHFLSGYTAK					Mascot
1914.9419	1915.0559	0.114	60	362	378	LTPAQAMYHFLSGYTAK			Oxidation (M)[7]		Mascot
1920.9563	1921.0986	0.1423	74	516	532	FESHVDHEVKDAAPAIR					Mascot
2159.1091	2159.2642	0.1551	72	106	125	ELFVQDLIGGADADNKIN AR					Mascot
2201.9995	2202.1606	0.1611	73	385	404	GVTEPEATFSTCFGAPF MPR			Carbamidomethyl (C)[12]		Mascot
2217.9944	2218.1641	0.1697	77	385	404	GVTEPEATFSTCFGAPF MPR			Carbamidomethyl (C)[12], Oxidation (M)[18]		Mascot
2554.3186	2554.5068	0.1882	74	466	489	IDPNFGFAVPVEVPGVES SILDPR					Mascot
2795.2153	2795.4216	0.2063	74	252	276	TLIGDDEHGWGEHGVFN FEGGCYAK			Carbamidomethyl (C)[22]		Mascot
3180.5081	3180.7366	0.2285	72	295	322	FGTVLENVVLDENRQPD FDDGSLTENTR					Mascot

2	chaperonin GroEL [Brucella suis ATCC 23445]	gi|163844383|ref|YP_001622038.1|	20	234	100	129	129	100	100	.T.	AlDahouk_6214_Z19769-18_T1	426

Peptide Information

Calc. Mass	Obsrv. Mass	± da	± ppm	Start Seq.	End Seq.	Sequence	Ion Score	C. I. %	Modification	Rank	Result Type
707.3947	707.4028	0.0081	11	8	13	FGRTAR					Mascot
719.347	719.3843	0.0373	52	278	284	APGFGDR					Mascot
721.3991	721.391	-0.0081	-11	5	10	DVKFGR					Mascot
726.4257	726.4626	0.0369	51	445	451	AIQAPAR					Mascot
855.541	855.5997	0.0587	69	372	380	LAGGVAVIR					Mascot
875.4482	875.508	0.0598	68	278	285	APGFGDRR					Mascot
882.5268	882.5919	0.0651	74	444	451	RAIQAPAR					Mascot
1233.6508	1233.7322	0.0814	66	309	319	LESVTLDMLGR					Mascot
1245.6685	1245.7115	0.043	35	381	392	VGGATEVEVKEK					Mascot

Calc. Mass	Obsrv. Mass	± da	± ppm	Start Seq.	End Seq.	Sequence	Ion Score	C. I. %	Modification	Rank	Result Type
1249.6456	1249.7323	0.0867	69	309	319	LESVTLDMLGR			Oxidation (M)[8]		Mascot
1344.7004	1344.7805	0.0801	60	169	181	VGNEGVITVEEAK					Mascot
1455.755	1455.8564	0.1014	70	430	443	GVNADQEAGINIVR					Mascot
1514.7526	1514.8666	0.114	75	198	210	GYLSPYFVTNPEK					Mascot
1582.905	1583.0127	0.1077	68	119	133	GIDLAVNEVVAELLK					Mascot
1583.8751	1583.9923	0.1172	74	405	421	AAVEEGIVAGGGTALLR	129	100			Mascot
1583.8751	1583.9923	0.1172	74	405	421	AAVEEGIVAGGGTALLR					Mascot
1711	1711.1211	0.1211	71	119	134	GIDLAVNEVVAELLKK					Mascot
1741.7875	1741.9152	0.1277	73	351	364	QQIEETTSDYDREK					Mascot
1759.8934	1760.0272	0.1338	76	211	225	MVADLEDAYILLHEK					Mascot
1775.8884	1776.0223	0.1339	75	211	225	MVADLEDAYILLHEK			Oxidation (M)[1]		Mascot
1853.8585	1853.9952	0.1367	74	182	197	TAETELEVVEGMQFDR					Mascot
1869.8535	1870.0022	0.1487	80	182	197	TAETELEVVEGMQFDR			Oxidation (M)[12]		Mascot
2038.9572	2039.1057	0.1485	73	59	75	EVELEDKFENMGAQMLR					Mascot
2054.9521	2055.1101	0.158	77	59	75	EVELEDKFENMGAQMLR			Oxidation (M)[11]		Mascot
2070.947	2071.1055	0.1585	77	59	75	EVELEDKFENMGAQMLR			Oxidation (M)[11,15]		Mascot
2419.1946	2419.3821	0.1875	78	81	105	TNDTAGDGTTTATVLGQAIVQEGAK					Mascot

3 unnamed protein product [Brucella suis 1330] gi|23501035|ref|NP_697162.1| 14 75 99.959 .T. AlDahouk_6214_Z19769-18_T1 426

Peptide Information

Calc. Mass	Obsrv. Mass	± da	± ppm	Start Seq.	End Seq.	Sequence	Ion Score	C. I. %	Modification	Rank	Result Type
763.4196	763.4937	0.0741	97	2	8	STLSLDK					Mascot
775.4824	775.5327	0.0503	65	24	29	LFNLLR					Mascot
834.4104	834.4135	0.0031	4	275	281	SWESLGR					Mascot
951.4894	951.5537	0.0643	68	56	64	SIGFDASVR					Mascot
1037.5051	1037.5776	0.0725	70	447	454	YELNSFHK					Mascot
1173.615	1173.6964	0.0814	69	46	55	AAEWLVEDLK					Mascot
1239.6328	1239.7209	0.0881	71	245	255	ILADLHDETGR					Mascot
1301.71	1301.792	0.082	63	45	55	KAAEWLVEDLK					Mascot
1786.9585	1787.0953	0.1368	77	282	299	TAESFLGPIGLSIPAGEK					Mascot
1914.8684	1915.0559	0.1875	98	130	146	GTSDDKGQLMTFVEACR			Carbamidomethyl (C)[16]		Mascot
1930.8633	1931.0475	0.1842	95	130	146	GTSDDKGQLMTFVEACR			Carbamidomethyl (C)[16], Oxidation (M)[10]		Mascot
2066.0818	2066.2263	0.1445	70	225	244	DLHSGFFGGAAANPIHILTK					Mascot

2193.1326	2193.2937	0.1611	73	256	274	ITIPDFYEGVEETPTQILK				Mascot
2479.2839	2479.4509	0.167	67	221	244	AADRDLHSGFFGGAAAN PIHILTK				Mascot
2649.3406	2649.5225	0.1819	69	159	182	VTLLFEGEEESGSPSLKP FLEANR				Mascot

**Gel Idx/Pos**	192/H19	**Instr./Gel Origin**	AK043/Div_120507	**Process Status**	Analysis Succeeded
**Plate [#] Name**	[1] 1300017700	**Instrument Sample Name**		**Spectra**	4

Rank	Protein	Name	Accession No.	Pep. Count	Protein Score	Protein Score C. I. %	Total Ion Score	Best Ion Score	Best Ion C. I. %	Total Ion C. I. %	Confirmed	Sample Name	Customer sample Name
1	unnamed protein product [Brucella suis 1330]		gi|23501748|ref|NP_697875.1|	12	169	100	78	44	99.972	100	.T.	AlDahouk_6214_Z19769-19_T1	1105

Peptide Information

Calc. Mass	Obsrv. Mass	± da	± ppm	Start Seq.	End Seq.	Sequence	Ion Score	C. I. %	Modification	Rank	Result Type
816.4838	816.5282	0.0444	54	154	160	RLGFAPR					Mascot
933.4577	933.5154	0.0577	62	32	38	VWSEWAR	44	99.972			Mascot
933.4577	933.5154	0.0577	62	32	38	VWSEWAR					Mascot
1050.6306	1050.694	0.0634	60	63	72	LALPGVDPIR					Mascot
1118.5688	1118.6381	0.0693	62	131	140	TIVSAEDVER					Mascot
1192.5957	1192.6703	0.0746	63	195	204	TSHLSIESYR					Mascot
1206.7317	1206.7711	0.0394	33	62	72	RLALPGVDPIR					Mascot
1280.6732	1280.7493	0.0761	59	205	215	YLQLTIEESGK					Mascot
1419.6936	1419.7847	0.0911	64	141	153	GKPSPECFELGAK			Carbamidomethyl (C)[7]		Mascot
1426.8053	1426.8948	0.0895	63	106	118	WAIVTSAPLELAR	35	99.786			Mascot
1426.8053	1426.8948	0.0895	63	106	118	WAIVTSAPLELAR					Mascot
1758.965	1759.0721	0.1071	61	40	55	HGIDPVTFLPTIHGVR					Mascot
1915.066	1915.1687	0.1027	54	39	55	RHGIDPVTFLPTIHGVR					Mascot
3430.7058	3430.8838	0.178	52	161	194	DCLVFEDAPAGIVAGETA GASVVVVTATHPHSPR			Carbamidomethyl (C)[2]		Mascot

Rank	Protein	Name	Accession No.	Pep. Count	Protein Score	Protein Score C. I. %	Total Ion Score	Best Ion Score	Best Ion C. I. %	Total Ion C. I. %	Confirmed	Sample Name	Customer sample Name
2	unnamed protein product [Brucella suis 1330]		gi|23502071|ref|NP_698198.1|	13	143	100	64	64	100	100	.T.	AlDahouk_6214_Z19769-19_T1	1105

Peptide Information

Calc. Mass	Obsrv. Mass	± da	± ppm	Start Seq.	End Seq.	Sequence	Ion Score	C. I. %	Modification	Rank	Result Type
707.4198	707.3862	-0.0336	-47	3	8	FGSKIR					Mascot

Calc. Mass	Obsrv. Mass	± da	± ppm	Start Seq.	End Seq.	Sequence	Ion Score	C. I. %	Modification	Rank	Result Type
713.3651	713.3708	0.0057	8	1	6	MKFGSK			Oxidation (M)[1]		Mascot
778.377	778.4225	0.0455	58	236	241	YSFFSK					Mascot
816.4573	816.5282	0.0709	87	113	119	GKVEDLR					Mascot
910.5104	910.5045	-0.0059	-6	289	297	KALDAGHAK					Mascot
919.4883	919.4639	-0.0244	-27	242	250	DVVPAGAYK					Mascot
1032.5109	1032.5758	0.0649	63	281	288	VLWNEDTR					Mascot
1160.6058	1160.6741	0.0683	59	281	289	VLWNEDTRK					Mascot
1261.6827	1261.7384	0.0557	44	232	241	ILEKYSFFSK					Mascot
1527.8013	1527.9005	0.0992	65	150	164	VSLDEPGSGIIVDAR					Mascot
1709.9949	1710.0978	0.1029	60	120	134	LLATLYPETIHIVAR	64	100			Mascot
1709.9949	1710.0978	0.1029	60	120	134	LLATLYPETIHIVAR					Mascot
1820.9501	1821.0593	0.1092	60	303	320	LDSATSSLGIPLHPGAER					Mascot
2821.4729	2821.6274	0.1545	55	165	190	IVLEAYGLTEDDIKAEHLKPGPAGER					Mascot

3	TRAP transporter solute receptor TAXI family protein [Brucella suis ATCC 23445]	gi|163843459|ref|YP_001627863.1|	12	133	100	64	64	100	100	.T.	AlDahouk_6214_Z19769-19_T1	1105

Peptide Information

Calc. Mass	Obsrv. Mass	± da	± ppm	Start Seq.	End Seq.	Sequence	Ion Score	C. I. %	Modification	Rank	Result Type
707.4198	707.3862	-0.0336	-47	3	8	FGSKIR					Mascot
713.3651	713.3708	0.0057	8	1	6	MKFGSK			Oxidation (M)[1]		Mascot
778.377	778.4225	0.0455	58	236	241	YSFFSK					Mascot
816.4573	816.5282	0.0709	87	113	119	GKVEDLR					Mascot
910.5104	910.5045	-0.0059	-6	289	297	KALDAGHAK					Mascot
919.4883	919.4639	-0.0244	-27	242	250	DVVPAGAYK					Mascot
1032.5109	1032.5758	0.0649	63	281	288	VLWNEDTR					Mascot
1160.6058	1160.6741	0.0683	59	281	289	VLWNEDTRK					Mascot
1261.6827	1261.7384	0.0557	44	232	241	ILEKYSFFSK					Mascot
1709.9949	1710.0978	0.1029	60	120	134	LLATLYPETIHIVAR	64	100			Mascot
1709.9949	1710.0978	0.1029	60	120	134	LLATLYPETIHIVAR					Mascot
1820.9501	1821.0593	0.1092	60	303	320	LDSATSSLGIPLHPGAER					Mascot
2821.4729	2821.6274	0.1545	55	165	190	IVLEAYGLTEDDIKAEHLKPGPAGER					Mascot

4	rplC gene product [Brucella suis 1330]	gi|23502110|ref|NP_698237.1|	11	59	98.505					.T.	AlDahouk_6214_Z19769-19_T1	1105

Peptide Information

Calc. Mass	Obsrv. Mass	± da	± ppm	Start Seq.	End Seq.	Sequence	Ion Score	C. I. %	Modification	Rank	Result Type
709.3701	709.3979	0.0278	39	118	124	GFAGVMK					Mascot
725.365	725.3962	0.0312	43	118	124	GFAGVMK			Oxidation (M)[6]		Mascot
814.4933	814.5369	0.0436	54	199	205	GAWILVR					Mascot
824.391	824.4399	0.0489	59	126	132	HNFGGHR					Mascot
919.4003	919.4639	0.0636	69	228	237	AEAAATEGAE					Mascot
933.4723	933.5154	0.0431	46	64	71	AMRGHFAK			Oxidation (M)[2]		Mascot
933.4723	933.5154	0.0431	46	64	71	AMRGHFAK			Oxidation (M)[2]		Mascot
1020.4349	1020.5128	0.0779	76	162	170	MAGHMGQTR			Oxidation (M)[1,5]		Mascot
1079.5341	1079.6022	0.0681	63	133	142	ASHGNSITHR					Mascot
1243.567	1243.6473	0.0803	65	30	39	MENCHVVAQR			Carbamidomethyl (C)[4]		Mascot
1259.562	1259.6467	0.0847	67	30	39	MENCHVVAQR			Carbamidomethyl (C)[4], Oxidation (M)[1]		Mascot
1663.8134	1663.9196	0.1062	64	171	185	VTTQNIEVVSTDSDR					Mascot
1668.8704	1668.9729	0.1025	61	15	29	VYNDAGEHVPVTVLR					Mascot
2452.2717	2452.4075	0.1358	55	85	107	VSPDNLLEVGVEITAEHF VAGQK					Mascot

5	50S ribosomal protein L3 [Brucella suis ATCC 23445]	gi|163843499|ref|YP_001627903.1|	11	58	98.029	.T.	AlDahouk_6214_Z19769-19_T1	1105

Peptide Information

Calc. Mass	Obsrv. Mass	± da	± ppm	Start Seq.	End Seq.	Sequence	Ion Score	C. I. %	Modification	Rank	Result Type
709.3701	709.3979	0.0278	39	118	124	GFAGVMK					Mascot
725.365	725.3962	0.0312	43	118	124	GFAGVMK			Oxidation (M)[6]		Mascot
814.4933	814.5369	0.0436	54	199	205	GAWILVR					Mascot
824.391	824.4399	0.0489	59	126	132	HNFGGHR					Mascot
919.4003	919.4639	0.0636	69	228	237	AEAAATEGAE					Mascot
933.4723	933.5154	0.0431	46	64	71	AMRGHFAK			Oxidation (M)[2]		Mascot
933.4723	933.5154	0.0431	46	64	71	AMRGHFAK			Oxidation (M)[2]		Mascot
1020.4349	1020.5128	0.0779	76	162	170	MAGHMGQTR			Oxidation (M)[1,5]		Mascot
1079.5341	1079.6022	0.0681	63	133	142	ASHGNSITHR					Mascot
1243.567	1243.6473	0.0803	65	30	39	MENCHVVAQR			Carbamidomethyl (C)[4]		Mascot
1259.562	1259.6467	0.0847	67	30	39	MENCHVVAQR			Carbamidomethyl (C)[4], Oxidation (M)[1]		Mascot
1663.8134	1663.9196	0.1062	64	171	185	VTTQNIEVVSTDSDR					Mascot

1668.8704	1668.9729	0.1025	61	15	29	VYNDAGEHVPVTVLR	Mascot
2452.2717	2452.4075	0.1358	55	85	107	VSPDNLLEVGVEITAEHF VAGQK	Mascot

Gel Idx/Pos	193/H20	Instr./Gel Origin	AK043/Div_120507	Process Status	Analysis Succeeded
Plate [#] Name	[1] 1300017700	Instrument Sample Name		Spectra	4

Rank	Protein Name	Accession No.	Pep. Count	Protein Score	Protein Score C. I. %	Total Ion Score	Best Ion Score	Best Ion C. I. %	Total Ion C. I. %	Confirmed	Sample Name	Customer sample Name
1	chaperonin GroEL [Brucella suis ATCC 23445]	gi|163844383|ref|YP_001622038.1|	24	392	100	255	99	100	100	.T.	AlDahouk_6214_Z19769-20_T1	421

Peptide Information

Calc. Mass	Obsrv. Mass	± da	± ppm	Start Seq.	End Seq.	Sequence	Ion Score	C. I. %	Modification	Rank	Result Type
707.3947	707.3776	-0.0171	-24	8	13	FGRTAR					Mascot
719.347	719.3655	0.0185	26	278	284	APGFGDR					Mascot
721.3991	721.3831	-0.016	-22	5	10	DVKFGR					Mascot
726.4257	726.4446	0.0189	26	445	451	AIQAPAR					Mascot
762.4178	762.4173	-0.0005	-1	1	7	MAAKDVK					Mascot
778.4127	778.3924	-0.0203	-26	1	7	MAAKDVK			Oxidation (M)[1]		Mascot
802.4417	802.4487	0.007	9	363	368	EKLQER					Mascot
819.4934	819.4564	-0.037	-45	422	429	ASTKITAK					Mascot
855.541	855.5812	0.0402	47	372	380	LAGGVAVIR					Mascot
875.4482	875.4865	0.0383	44	278	285	APGFGDRR					Mascot
882.5268	882.5778	0.051	58	444	451	RAIQAPAR					Mascot
1233.6508	1233.7042	0.0534	43	309	319	LESVTLDMLGR					Mascot
1245.6685	1245.7043	0.0358	29	381	392	VGGATEVEVKEK					Mascot
1373.7028	1373.7891	0.0863	63	106	118	AVAAGMNPMDLKR					Mascot
1455.755	1455.8254	0.0704	48	430	443	GVNADQEAGINIVR	66	100			Mascot
1455.755	1455.8254	0.0704	48	430	443	GVNADQEAGINIVR					Mascot
1514.7526	1514.8307	0.0781	52	198	210	GYLSPYFVTNPEK					Mascot
1582.905	1582.9811	0.0761	48	119	133	GIDLAVNEVVAELLK					Mascot
1583.8751	1583.9564	0.0813	51	405	421	AAVEEGIVAGGGTALLR	91	100			Mascot
1583.8751	1583.9564	0.0813	51	405	421	AAVEEGIVAGGGTALLR					Mascot
1711	1711.0862	0.0862	50	119	134	GIDLAVNEVVAELLKK					Mascot
1741.7875	1741.8732	0.0857	49	351	364	QQIEETTSDYDREK					Mascot

1759.8934	1759.98	0.0866	49	211	225	MVADLEDAYILLHEK				Mascot
1853.8585	1853.9586	0.1001	54	182	197	TAETELEVVEGMQFDR	99	100		Mascot
1853.8585	1853.9586	0.1001	54	182	197	TAETELEVVEGMQFDR				Mascot
1869.8535	1869.9612	0.1077	58	182	197	TAETELEVVEGMQFDR			Oxidation (M)[12]	Mascot
1903.9834	1904.072	0.0886	47	211	226	MVADLEDAYILLHEKK			Oxidation (M)[1]	Mascot
2038.9572	2039.0575	0.1003	49	59	75	EVELEDKFENMGAQMLR				Mascot
2419.1946	2419.324	0.1294	53	81	105	TNDTAGDGTTTATVLGQ AIVQEGAK				Mascot

Gel Idx/Pos	194/H21	Instr./Gel Origin	AK043/Div_120507	Process Status	Analysis Succeeded
Plate [#] Name	[1] 1300017700	Instrument Sample Name		Spectra	4

Rank	Protein Name	Accession No.	Pep. Count	Protein Score	Protein Score C. I. %	Total Ion Score	Best Ion Score	Best Ion C. I. %	Total Ion C. I. %	Confirmed	Sample Name	Customer sample Name
1	katA gene product [Brucella suis 1330]	gi|23500108|ref|NP_699548.1|	29	453	100	214	78	100	100	.T.	AlDahouk_6214_Z19769-21_T1	377

Peptide Information

Calc. Mass	Obsrv. Mass	± da	± ppm	Start Seq.	End Seq.	Sequence	Ion Score	C. I. %	Modification	Rank	Result Type
729.4042	729.4213	0.0171	23	459	464	QLGHFK					Mascot
754.3954	754.4226	0.0272	36	41	46	LSHQNR					Mascot
757.4202	757.4455	0.0253	33	183	189	GLPTDVR					Mascot
799.442	799.4709	0.0289	36	47	52	ERIPER					Mascot
973.5464	973.5859	0.0395	41	450	458	GVPGFIVER	73	100			Mascot
973.5464	973.5859	0.0395	41	450	458	GVPGFIVER					Mascot
1065.5762	1065.6155	0.0393	37	440	449	LFSNIAAAMK					Mascot
1079.5269	1079.5723	0.0454	42	334	342	IFSYADAHR	63	100			Mascot
1079.5269	1079.5723	0.0454	42	334	342	IFSYADAHR					Mascot
1121.5421	1121.5914	0.0493	44	359	366	CLVHHYHR			Carbamidomethyl (C)[1]		Mascot
1132.5786	1132.6282	0.0496	44	140	148	FPDFIHTQK					Mascot
1164.5281	1164.5774	0.0493	42	417	426	IGNDDYSQPR					Mascot
1181.5619	1181.6136	0.0517	44	367	377	DGQMNVYGGIK					Mascot
1197.5569	1197.6124	0.0555	46	367	377	DGQMNVYGGIK			Oxidation (M)[4]		Mascot
1237.6576	1237.7104	0.0528	43	427	437	ALFNLFDAAQK					Mascot
1283.6742	1283.7299	0.0557	43	465	475	LIHPEYEAGVR	78	100			Mascot
1283.6742	1283.7299	0.0557	43	465	475	LIHPEYEAGVR					Mascot

1328.6263	1328.6854	0.0591	44	401	412	EPPLCISGNADR			Carbamidomethyl (C)[5]		Mascot
1381.7621	1381.8203	0.0582	42	79	91	VLQPGAQTPMLAR					Mascot
1397.757	1397.8212	0.0642	46	79	91	VLQPGAQTPMLAR			Oxidation (M)[10]		Mascot
1411.7692	1411.8306	0.0614	43	465	476	LIHPEYEAGVRK					Mascot
1493.723	1493.7891	0.0661	44	92	106	FSTVAGELGAADAER					Mascot
1510.7397	1510.8075	0.0678	45	223	235	HWTNAEAEQVIGR					Mascot
1547.8138	1547.8867	0.0729	47	28	40	GPILMQDYQLIEK					Mascot
1563.8087	1563.8822	0.0735	47	28	40	GPILMQDYQLIEK			Oxidation (M)[5]		Mascot
1582.8224	1582.9071	0.0847	54	345	358	LGTHYESIPVNQPK					Mascot
1585.8373	1585.9209	0.0836	53	136	148	DPLKFPDFIHTQK					Mascot
1631.766	1631.8456	0.0796	49	480	494	DAHGYDANTIALNEK					Mascot
1667.8387	1667.9109	0.0722	43	58	73	GWGAYGTLTITGDISR					Mascot
2040.9396	2041.0432	0.1036	51	238	255	ESTQEDLFSAIENGEFPK					Mascot
2222.1062	2222.1975	0.0913	41	281	299	VWPHADYPPIDIGVMELN R					Mascot
2238.1011	2238.2043	0.1032	46	281	299	VWPHADYPPIDIGVMELN R			Oxidation (M)[15]		Mascot
2482.1157	2482.2341	0.1184	48	378	400	TGNPDAYYEPNSFNGPV EQPSAK					Mascot
2503.2041	2503.3057	0.1016	41	115	135	FYTQEGNWDLVGNNTPV FFVR					Mascot
2662.3433	2662.4578	0.1145	43	258	280	VQVQIMPELDADKTPYNP FDLTK					Mascot
2713.321	2713.4353	0.1143	42	2	27	TDRPIMTTSAGAPIPDNQ NSLTAGER					Mascot
2729.3159	2729.4497	0.1338	49	2	27	TDRPIMTTSAGAPIPDNQ NSLTAGER			Oxidation (M)[6]		Mascot

2	hypothetical protein BSUIS_B0360 [Brucella suis ATCC 23445]	gi|163844531|ref|YP_001622186.1|	28	441	100	214	78	100	100	.T.	AlDahouk_6214_Z19769-21_T1	377

Peptide Information

Calc. Mass	Obsrv. Mass	± da	± ppm	Start Seq.	End Seq.	Sequence	Ion Score	C. I. %	Modification	Rank	Result Type
729.4042	729.4213	0.0171	23	459	464	QLGHFK					Mascot
754.3954	754.4226	0.0272	36	41	46	LSHQNR					Mascot
757.4202	757.4455	0.0253	33	183	189	GLPTDVR					Mascot
799.442	799.4709	0.0289	36	47	52	ERIPER					Mascot
973.5464	973.5859	0.0395	41	450	458	GVPGFIVER	73	100			Mascot
973.5464	973.5859	0.0395	41	450	458	GVPGFIVER					Mascot
1065.5762	1065.6155	0.0393	37	440	449	LFSNIAAAMK					Mascot
1079.5269	1079.5723	0.0454	42	334	342	IFSYADAHR	63	100			Mascot

Calc. Mass	Obsrv. Mass	± da	± ppm	Start Seq.	End Seq.	Sequence	Ion Score	C. I. %	Modification	Rank	Result Type
1079.5269	1079.5723	0.0454	42	334	342	IFSYADAHR					Mascot
1132.5786	1132.6282	0.0496	44	140	148	FPDFIHTQK					Mascot
1164.5281	1164.5774	0.0493	42	417	426	IGNDDYSQPR					Mascot
1181.5619	1181.6136	0.0517	44	367	377	DGQMNVYGGIK					Mascot
1197.5569	1197.6124	0.0555	46	367	377	DGQMNVYGGIK			Oxidation (M)[4]		Mascot
1237.6576	1237.7104	0.0528	43	427	437	ALFNLFDAAQK					Mascot
1283.6742	1283.7299	0.0557	43	465	475	LIHPEYEAGVR	78	100			Mascot
1283.6742	1283.7299	0.0557	43	465	475	LIHPEYEAGVR					Mascot
1328.6263	1328.6854	0.0591	44	401	412	EPPLCISGNADR			Carbamidomethyl (C)[5]		Mascot
1381.7621	1381.8203	0.0582	42	79	91	VLQPGAQTPMLAR					Mascot
1397.757	1397.8212	0.0642	46	79	91	VLQPGAQTPMLAR			Oxidation (M)[10]		Mascot
1411.7692	1411.8306	0.0614	43	465	476	LIHPEYEAGVRK					Mascot
1493.723	1493.7891	0.0661	44	92	106	FSTVAGELGAADAER					Mascot
1510.7397	1510.8075	0.0678	45	223	235	HWTNAEAEQVIGR					Mascot
1547.8138	1547.8867	0.0729	47	28	40	GPILMQDYQLIEK					Mascot
1563.8087	1563.8822	0.0735	47	28	40	GPILMQDYQLIEK			Oxidation (M)[5]		Mascot
1582.8224	1582.9071	0.0847	54	345	358	LGTHYESIPVNQPK					Mascot
1585.8373	1585.9209	0.0836	53	136	148	DPLKFPDFIHTQK					Mascot
1631.766	1631.8456	0.0796	49	480	494	DAHGYDANTIALNEK					Mascot
1667.8387	1667.9109	0.0722	43	58	73	GWGAYGTLTITGDISR					Mascot
2040.9396	2041.0432	0.1036	51	238	255	ESTQEDLFSAIENGEFPK					Mascot
2222.1062	2222.1975	0.0913	41	281	299	VWPHADYPPIDIGVMELN R					Mascot
2238.1011	2238.2043	0.1032	46	281	299	VWPHADYPPIDIGVMELN R			Oxidation (M)[15]		Mascot
2482.1157	2482.2341	0.1184	48	378	400	TGNPDAYYEPNSFNGPV EQPSAK					Mascot
2503.2041	2503.3057	0.1016	41	115	135	FYTQEGNWDLVGNNTPV FFVR					Mascot
2662.3433	2662.4578	0.1145	43	258	280	VQVQIMPELDADKTPYNP FDLTK					Mascot
2713.321	2713.4353	0.1143	42	2	27	TDRPIMTTSAGAPIPDNQ NSLTAGER					Mascot
2729.3159	2729.4497	0.1338	49	2	27	TDRPIMTTSAGAPIPDNQ NSLTAGER			Oxidation (M)[6]		Mascot

3 chaperonin GroEL [Brucella suis ATCC 23445] gi|163844383|ref|YP_001622038.1| 17 67 99.722 .T. AlDahouk_6214_Z19769-21_T1 377

Peptide Information

Calc. Mass	Obsrv. Mass	± da	± ppm	Start Seq.	End Seq.	Sequence	Ion Score	C. I. %	Modification	Rank	Result Type

707.3947	707.3763	-0.0184	-26	8	13	FGRTAR					Mascot
719.347	719.364	0.017	24	278	284	APGFGDR					Mascot
721.3991	721.3859	-0.0132	-18	5	10	DVKFGR					Mascot
726.4257	726.4406	0.0149	21	445	451	AIQAPAR					Mascot
802.4417	802.4291	-0.0126	-16	363	368	EKLQER					Mascot
855.541	855.5762	0.0352	41	372	380	LAGGVAVIR					Mascot
875.4482	875.48	0.0318	36	278	285	APGFGDRR					Mascot
882.5268	882.5699	0.0431	49	444	451	RAIQAPAR					Mascot
1233.6508	1233.7018	0.051	41	309	319	LESVTLDMLGR					Mascot
1245.6685	1245.6758	0.0073	6	381	392	VGGATEVEVKEK					Mascot
1249.6456	1249.6987	0.0531	42	309	319	LESVTLDMLGR			Oxidation (M)[8]		Mascot
1455.755	1455.8174	0.0624	43	430	443	GVNADQEAGINIVR					Mascot
1514.7526	1514.8198	0.0672	44	198	210	GYLSPYFVTNPEK					Mascot
1582.905	1582.9071	0.0021	1	119	133	GIDLAVNEVVAELLK					Mascot
1583.8751	1583.9302	0.0551	35	405	421	AAVEEGIVAGGGTALLR					Mascot
1741.7875	1741.8715	0.084	48	351	364	QQIEETTSDYDREK					Mascot
1853.8585	1853.9396	0.0811	44	182	197	TAETELEVVEGMQFDR					Mascot
1869.8535	1869.9463	0.0928	50	182	197	TAETELEVVEGMQFDR			Oxidation (M)[12]		Mascot
2038.9572	2039.0511	0.0939	46	59	75	EVELEDKFENMGAQMLR					Mascot

Gel Idx/Pos	195/H22	Instr./Gel Origin	AK043/Div_120507	Process Status	Analysis Succeeded
Plate [#] Name	[1] 1300017700	Instrument Sample Name		Spectra	4

Rank	Protein	Name	Accession No.	Pep. Count	Protein Score	Protein Score C. I. %	Total Ion Score	Best Ion Score	Best Ion C. I. %	Total Ion C. I. %	Confirmed	Sample Name	Customer sample Name
1	chaperonin GroEL [Brucella suis ATCC 23445]		gi|163844383|ref|YP_001622038.1|	33	686	100	420	164	100	100	.T.	AlDahouk_6214_Z19769-22_T1	519

Peptide Information

Calc. Mass	Obsrv. Mass	± da	± ppm	Start Seq.	End Seq.	Sequence	Ion Score	C. I. %	Modification	Rank	Result Type
707.3947	707.3667	-0.028	-40	8	13	FGRTAR					Mascot
719.347	719.3543	0.0073	10	278	284	APGFGDR					Mascot
726.4257	726.4296	0.0039	5	445	451	AIQAPAR					Mascot
855.541	855.5646	0.0236	28	372	380	LAGGVAVIR					Mascot

875.4482	875.472	0.0238	27	278	285	APGFGDRR				Mascot
882.5268	882.5526	0.0258	29	444	451	RAIQAPAR				Mascot
974.4901	974.5158	0.0257	26	396	404	VDDALNATR				Mascot
1000.5673	1000.5847	0.0174	17	19	28	GVDILADAVK				Mascot
1232.6117	1232.652	0.0403	33	328	339	ENTTIVDGAGQK				Mascot
1233.6508	1233.6832	0.0324	26	309	319	LESVTLDMLGR				Mascot
1245.6685	1245.6575	-0.011	-9	381	392	VGGATEVEVKEK				Mascot
1249.6456	1249.683	0.0374	30	309	319	LESVTLDMLGR			Oxidation (M)[8]	Mascot
1344.7004	1344.7445	0.0441	33	169	181	VGNEGVITVEEAK				Mascot
1373.7028	1373.7523	0.0495	36	106	118	AVAAGMNPMDLKR				Mascot
1389.6978	1389.713	0.0152	11	106	118	AVAAGMNPMDLKR			Oxidation (M)[6]	Mascot
1405.6926	1405.744	0.0514	37	106	118	AVAAGMNPMDLKR			Oxidation (M)[6,9]	Mascot
1455.755	1455.8005	0.0455	31	430	443	GVNADQEAGINIVR	122	100		Mascot
1455.755	1455.8005	0.0455	31	430	443	GVNADQEAGINIVR				Mascot
1484.65	1484.6998	0.0498	34	351	362	QQIEETTSDYDR				Mascot
1514.7526	1514.8091	0.0565	37	198	210	GYLSPYFVTNPEK				Mascot
1582.905	1582.9595	0.0545	34	119	133	GIDLAVNEVVAELLK				Mascot
1583.8751	1583.9298	0.0547	35	405	421	AAVEEGIVAGGGTALLR	164	100		Mascot
1583.8751	1583.9298	0.0547	35	405	421	AAVEEGIVAGGGTALLR				Mascot
1611.8561	1611.9164	0.0603	37	430	444	GVNADQEAGINIVRR				Mascot
1616.849	1616.9055	0.0565	35	452	467	QITTNAGEEASVIVGK				Mascot
1711	1711.0607	0.0607	35	119	134	GIDLAVNEVVAELLKK				Mascot
1739.0061	1739.0607	0.0546	31	118	133	RGIDLAVNEVVAELLK				Mascot
1741.7875	1741.8546	0.0671	39	351	364	QQIEETTSDYDREK				Mascot
1759.8934	1759.9587	0.0653	37	211	225	MVADLEDAYILLHEK				Mascot
1775.8884	1775.9602	0.0718	40	211	225	MVADLEDAYILLHEK			Oxidation (M)[1]	Mascot
1853.8585	1853.9224	0.0639	34	182	197	TAETELEVVEGMQFDR	133	100		Mascot
1853.8585	1853.9224	0.0639	34	182	197	TAETELEVVEGMQFDR				Mascot
1869.0188	1868.9661	-0.0527	-28	426	443	ITAKGVNADQEAGINIVR				Mascot
1869.8535	1869.9283	0.0748	40	182	197	TAETELEVVEGMQFDR			Oxidation (M)[12]	Mascot
1887.9884	1887.9819	-0.0065	-3	211	226	MVADLEDAYILLHEKK				Mascot
1903.9834	1904.0492	0.0658	35	211	226	MVADLEDAYILLHEKK			Oxidation (M)[1]	Mascot
2038.9572	2039.0254	0.0682	33	59	75	EVELEDKFENMGAQMLR				Mascot
2054.9521	2055.0408	0.0887	43	59	75	EVELEDKFENMGAQMLR			Oxidation (M)[11]	Mascot
2070.947	2071.0332	0.0862	42	59	75	EVELEDKFENMGAQMLR			Oxidation (M)[11,15]	Mascot
2286.2261	2286.314	0.0879	38	287	308	AMLEDIAILTGGQVISEDL				Mascot

Calc. Mass	Obsrv. Mass	± da	± ppm	Start Seq.	End Seq.	Sequence	Ion Score	C. I. %	Modification	Rank	Result Type
						GIK					
2302.2209	2302.3164	0.0955	41	287	308	AMLEDIAILTGGQVISEDL GIK			Oxidation (M)[2]		Mascot
2317.1516	2317.249	0.0974	42	138	160	INTSEEVAQVGTISANGE AEIGK					Mascot
2419.1946	2419.3037	0.1091	45	81	105	TNDTAGDGTTTATVLGQ AIVQEGAK					Mascot
2445.2466	2445.3538	0.1072	44	137	160	KINTSEEVAQVGTISANG EAEIGK					Mascot
3329.6423	3329.7878	0.1455	44	468	498	ILENTSETFGYNTANGEY GDLISLGIVDPVK					Mascot

2	unnamed protein product [Brucella suis 1330]	gi|23500285|ref|NP_699725.1|	17	96	100	.T.	AlDahouk_6214_Z19769-22_T1	519

Peptide Information

Calc. Mass	Obsrv. Mass	± da	± ppm	Start Seq.	End Seq.	Sequence	Ion Score	C. I. %	Modification	Rank	Result Type
759.4182	759.4648	0.0466	61	110	115	RLMDPK					Mascot
818.4003	818.4222	0.0219	27	466	472	TQDLGER					Mascot
1019.4792	1019.5106	0.0314	31	415	423	EDGPFDIAR					Mascot
1175.5844	1175.6158	0.0314	27	230	238	VNWIPFEDR					Mascot
1385.7498	1385.7667	0.0169	12	116	128	TAAGYASMLFIIK					Mascot
1389.6321	1389.713	0.0809	58	216	226	NPYYYEADQVK					Mascot
1455.6785	1455.8005	0.122	84	453	465	NADYDALMAKAEK			Oxidation (M)[8]		Mascot
1455.6785	1455.8005	0.122	84	453	465	NADYDALMAKAEK			Oxidation (M)[8]		Mascot
1513.8124	1513.8613	0.0489	32	274	286	MAPYLGVYYLPVK					Mascot
1523.7224	1523.7769	0.0545	36	52	65	DLYDGLTIQDADGK					Mascot
1529.8073	1529.8353	0.028	18	274	286	MAPYLGVYYLPVK			Oxidation (M)[1]		Mascot
1660.7965	1660.8494	0.0529	32	401	414	LNETEGASYFNFLR					Mascot
1863.9811	1864.0508	0.0697	37	138	154	KPVDQLGVEAVDDHTLK					Mascot
1887.9347	1887.9819	0.0472	25	399	414	ARLNETEGASYFNFLR					Mascot
1925.9028	1925.9619	0.0591	31	73	88	SWDISEDGTVYTFHLR					Mascot
1944.8875	1944.953	0.0655	34	93	109	WSNGDPVTAGDFQFTFR					Mascot
1946.0244	1945.9495	-0.0749	-38	271	286	EFRMAPYLGVYYLPVK					Mascot
2557.2026	2557.3015	0.0989	39	28	51	GNDTDPATLDHHHTSTV AEGNVLR					Mascot
2661.2578	2661.3542	0.0964	36	401	423	LNETEGASYFNFLREDG PFDIAR					Mascot

3	unnamed protein product [Brucella suis 1330]	gi|23501912|ref|NP_698039.1|	12	68	99.798	.T.	AlDahouk_6214_Z19769-22_T1	519

Peptide Information

Calc. Mass	Obsrv. Mass	± da	± ppm	Start Seq.	End Seq.	Sequence	Ion Score	C. I. %	Modification	Rank	Result Type
703.3733	703.3784	0.0051	7	2	7	SDKTPR					Mascot
705.3678	705.3624	-0.0054	-8	72	77	GWSLSR					Mascot
713.394	713.3269	-0.0671	-94	14	19	LEQPAR					Mascot
719.4199	719.3543	-0.0656	-91	8	13	KPTAFR					Mascot
726.4733	726.4296	-0.0437	-60	31	36	RPRAVK					Mascot
818.3638	818.4222	0.0584	71	147	154	DAADAAER					Mascot
834.4138	834.3339	-0.0799	-96	1	7	MSDKTPR					Mascot
1611.8197	1611.9164	0.0967	60	20	33	VSAASEQEEPRRPR					Mascot
1896.941	1896.934	-0.007	-4	14	30	LEQPARVSAASEQEEPR					Mascot
1991.0419	1991.0074	-0.0345	-17	318	335	IGIAAMDVVRPFPFNAEK			Oxidation (M)[6]		Mascot
2071.103	2071.0332	-0.0698	-34	182	199	QLLDSLTDDIIDGRDLIR					Mascot
3329.8362	3329.7878	-0.0484	-15	272	304	VIAHLAVTGTIAMGDSVIQ QLVGHGLASRLSAK			Oxidation (M)[13]		Mascot

4 unnamed protein product [Brucella suis 1330] gi|23501498|ref|NP_697625.1| 13 61 98.941 .T. AlDahouk_6214_Z19769-22_T1 519

Peptide Information

Calc. Mass	Obsrv. Mass	± da	± ppm	Start Seq.	End Seq.	Sequence	Ion Score	C. I. %	Modification	Rank	Result Type
703.3984	703.3784	-0.02	-28	481	487	AITAAEK					Mascot
741.4253	741.4042	-0.0211	-28	174	180	LIGADPR					Mascot
759.4611	759.4648	0.0037	5	181	187	TDLAVLK					Mascot
816.4726	816.4898	0.0172	21	384	390	AALTVWR					Mascot
851.3716	851.4033	0.0317	37	104	110	DFGMEPR					Mascot
867.3665	867.3984	0.0319	37	104	110	DFGMEPR			Oxidation (M)[4]		Mascot
982.6155	982.6416	0.0261	27	60	68	VRPAVVSVR					Mascot
1325.7576	1325.7977	0.0401	30	310	321	GWIGVQIQPVTK					Mascot
1529.7747	1529.8353	0.0606	40	195	207	FVYVAFGDDNKVR					Mascot
1599.8813	1599.9226	0.0413	26	491	504	KAVLLQLQSNDQSR					Mascot
1741.8715	1741.8546	-0.0169	-10	353	369	AGDVITAVNGETVQDPR					Mascot
1854.0232	1853.9224	-0.1008	-54	305	321	GSVERGWIGVQIQPVTK					Mascot
1854.0232	1853.9224	-0.1008	-54	305	321	GSVERGWIGVQIQPVTK					Mascot
2307.167	2307.2385	0.0715	31	80	100	GPQFFGPPGFDQLPDGH PLKR					Mascot

Calc. Mass	Obsrv. Mass	± da	± ppm	Start Seq.	End Seq.	Sequence	Ion Score	C. I. %	Modification	Rank	Result Type
2515.3303	2515.4019	0.0716	28	208	233	VGDWVVAVGNPFGLGG TVTSGIVSAR					Mascot

5 hypothetical protein BSUIS_B0534 [Brucella suis ATCC 23445] gi|163844697|ref|YP_001622352.1| 13 60 98.839 .T. AlDahouk_6214_Z19769-22_T1 519

Peptide Information

Calc. Mass	Obsrv. Mass	± da	± ppm	Start Seq.	End Seq.	Sequence	Ion Score	C. I. %	Modification	Rank	Result Type
759.4182	759.4648	0.0466	61	110	115	RLMDPK					Mascot
818.4003	818.4222	0.0219	27	466	472	TQDLGER					Mascot
1175.5844	1175.6158	0.0314	27	230	238	VNWIPFEDR					Mascot
1389.6321	1389.713	0.0809	58	216	226	NPYYYEADQVK					Mascot
1455.6785	1455.8005	0.122	84	453	465	NADYDALMAKAEK			Oxidation (M)[8]		Mascot
1455.6785	1455.8005	0.122	84	453	465	NADYDALMAKAEK			Oxidation (M)[8]		Mascot
1513.8124	1513.8613	0.0489	32	274	286	MAPYLGVYYLPVK					Mascot
1523.7224	1523.7769	0.0545	36	52	65	DLYDGLTIQDADGK					Mascot
1529.8073	1529.8353	0.028	18	274	286	MAPYLGVYYLPVK			Oxidation (M)[1]		Mascot
1660.7965	1660.8494	0.0529	32	401	414	LNETEGASYFNFLR					Mascot
1863.9811	1864.0508	0.0697	37	138	154	KPVDQLGVEAVDDHTLK					Mascot
1887.9347	1887.9819	0.0472	25	399	414	ARLNETEGASYFNFLR					Mascot
1925.9028	1925.9619	0.0591	31	73	88	SWDISEDGTVYTFHLR					Mascot
1946.0244	1945.9495	-0.0749	-38	271	286	EFRMAPYLGVYYLPVK					Mascot
2557.2026	2557.3015	0.0989	39	28	51	GNDTDPATLDHHHTSTV AEGNVLR					Mascot

6 glcD gene product [Brucella suis 1330] gi|23499942|ref|NP_699382.1| 13 60 98.756 .T. AlDahouk_6214_Z19769-22_T1 519

Peptide Information

Calc. Mass	Obsrv. Mass	± da	± ppm	Start Seq.	End Seq.	Sequence	Ion Score	C. I. %	Modification	Rank	Result Type
705.3348	705.3624	0.0276	39	477	482	CAELGR			Carbamidomethyl (C)[1]		Mascot
759.4511	759.4648	0.0137	18	470	475	VFPQLR					Mascot
807.3995	807.4134	0.0139	17	273	279	AAEDFVR					Mascot
820.4464	820.4623	0.0159	19	330	336	LAFWAGR					Mascot
1048.4629	1048.4961	0.0332	32	73	80	YCHDNAIR			Carbamidomethyl (C)[2]		Mascot
1056.5836	1056.6157	0.0321	30	488	497	GALAFPDLPR					Mascot
1132.5997	1132.6323	0.0326	29	111	119	ILEIDYPNR					Mascot

Calc. Mass	Obsrv. Mass	± da	± ppm	Start Seq.	End Seq.	Sequence	Ion Score	C. I. %	Modification	Rank	Result Type
1273.6536	1273.6929	0.0393	31	42	52	VFETDGLTVHR					Mascot
1582.8257	1582.9595	0.1338	85	2	16	SGLIMPEPDAGVLQR					Mascot
1738.9269	1739.0607	0.1338	77	2	17	SGLIMPEPDAGVLQRR					Mascot
1869.9673	1869.9283	-0.039	-21	1	17	MSGLIMPEPDAGVLQRR					Mascot
1885.9623	1885.9385	-0.0238	-13	1	17	MSGLIMPEPDAGVLQRR			Oxidation (M)[1]		Mascot
2230.3169	2230.3728	0.0559	25	53	72	QLPLVVVLPETVEQVAQV LR					Mascot
2731.4116	2731.5625	0.1509	55	81	107	VVPRGAGTSLSGGSMPL EDAVLLVMSR			Oxidation (M)[15,25]		Mascot

Rank	Protein Name	Accession No.	Pep. Count	Protein Score	Protein Score C. I. %	Total Ion Score	Best Ion Score	Best Ion C. I. %	Total Ion C. I. %	Confirmed	Sample Name	Customer sample Name
7	hypothetical protein BSUIS_A1077 [Brucella suis ATCC 23445]	gi|163843302|ref|YP_001627706.1|	11	59	98.47					.T.	AlDahouk_6214_Z19769-22_T1	519

Peptide Information

Calc. Mass	Obsrv. Mass	± da	± ppm	Start Seq.	End Seq.	Sequence	Ion Score	C. I. %	Modification	Rank	Result Type
703.3733	703.3784	0.0051	7	2	7	SDKTPR					Mascot
705.3678	705.3624	-0.0054	-8	72	77	GWSLSR					Mascot
707.4311	707.3667	-0.0644	-91	31	36	HPRAVK					Mascot
713.394	713.3269	-0.0671	-94	14	19	LEQPAR					Mascot
719.4199	719.3543	-0.0656	-91	8	13	KPTAFR					Mascot
818.3638	818.4222	0.0584	71	147	154	DAADAAER					Mascot
834.4138	834.3339	-0.0799	-96	1	7	MSDKTPR					Mascot
1896.941	1896.934	-0.007	-4	14	30	LEQPARVSAASEQEEPR					Mascot
1991.0419	1991.0074	-0.0345	-17	318	335	IGIAAMDVVRPFPFNAEK			Oxidation (M)[6]		Mascot
2071.103	2071.0332	-0.0698	-34	182	199	QLLDSLTDDIIDGRDLIR					Mascot
3329.8362	3329.7878	-0.0484	-15	272	304	VIAHLAVTGTIAMGDSVIQ QLVGHGLASRLSAK			Oxidation (M)[13]		Mascot

**Gel Idx/Pos**	196/H23	**Instr./Gel Origin**	AK043/Div_120507	**Process Status**	Analysis Succeeded
**Plate [#] Name**	[1] 1300017700	**Instrument Sample Name**		**Spectra**	4

Rank	Protein Name	Accession No.	Pep. Count	Protein Score	Protein Score C. I. %	Total Ion Score	Best Ion Score	Best Ion C. I. %	Total Ion C. I. %	Confirmed	Sample Name	Customer sample Name
1	chaperonin GroEL [Brucella suis ATCC 23445]	gi|163844383|ref|YP_001622038.1|	37	762	100	440	158	100	100	.T.	AlDahouk_6214_Z19769-23_T1	417

Peptide Information

Calc. Mass	Obsrv. Mass	± da	± ppm	Start Seq.	End Seq.	Sequence	Ion Score	C. I. %	Modification	Rank	Result Type

707.3947	707.3579	-0.0368	-52	8	13	FGRTAR				Mascot
719.347	719.3406	-0.0064	-9	278	284	APGFGDR				Mascot
726.4257	726.4175	-0.0082	-11	445	451	AIQAPAR				Mascot
855.541	855.5502	0.0092	11	372	380	LAGGVAVIR				Mascot
875.4482	875.457	0.0088	10	278	285	APGFGDRR				Mascot
882.5268	882.5349	0.0081	9	444	451	RAIQAPAR				Mascot
921.4532	921.4625	0.0093	10	161	168	MIAEAMQK				Mascot
937.4481	937.4614	0.0133	14	161	168	MIAEAMQK			Oxidation (M)[1]	Mascot
974.4901	974.5013	0.0112	11	396	404	VDDALNATR				Mascot
1000.5673	1000.5744	0.0071	7	19	28	GVDILADAVK				Mascot
1217.6017	1217.6243	0.0226	19	106	117	AVAAGMNPMDLK				Mascot
1228.5449	1228.5914	0.0465	38	66	75	FENMGAQMLR			Oxidation (M)[4,8]	Mascot
1232.6117	1232.6324	0.0207	17	328	339	ENTTIVDGAGQK				Mascot
1233.6508	1233.6644	0.0136	11	309	319	LESVTLDMLGR				Mascot
1245.6183	1245.6389	0.0206	17	394	404	DRVDDALNATR				Mascot
1249.6456	1249.6635	0.0179	14	309	319	LESVTLDMLGR			Oxidation (M)[8]	Mascot
1344.7004	1344.7207	0.0203	15	169	181	VGNEGVITVEEAK				Mascot
1373.7028	1373.7273	0.0245	18	106	118	AVAAGMNPMDLKR				Mascot
1389.6978	1389.7208	0.023	17	106	118	AVAAGMNPMDLKR			Oxidation (M)[6]	Mascot
1405.6926	1405.7218	0.0292	21	106	118	AVAAGMNPMDLKR			Oxidation (M)[6,9]	Mascot
1455.755	1455.7767	0.0217	15	430	443	GVNADQEAGINIVR	134	100		Mascot
1455.755	1455.7767	0.0217	15	430	443	GVNADQEAGINIVR				Mascot
1484.65	1484.6768	0.0268	18	351	362	QQIEETTSDYDR				Mascot
1514.7526	1514.7792	0.0266	18	198	210	GYLSPYFVTNPEK				Mascot
1582.905	1582.933	0.028	18	119	133	GIDLAVNEVVAELLK				Mascot
1583.8751	1583.9023	0.0272	17	405	421	AAVEEGIVAGGGTALLR	158	100		Mascot
1583.8751	1583.9023	0.0272	17	405	421	AAVEEGIVAGGGTALLR				Mascot
1611.8561	1611.8805	0.0244	15	430	444	GVNADQEAGINIVRR				Mascot
1616.849	1616.879	0.03	19	452	467	QITTNAGEEASVIVGK				Mascot
1711	1711.0336	0.0336	20	119	134	GIDLAVNEVVAELLKK				Mascot
1739.0061	1739.0302	0.0241	14	118	133	RGIDLAVNEVVAELLK				Mascot
1741.7875	1741.8193	0.0318	18	351	364	QQIEETTSDYDREK				Mascot
1759.8934	1759.9257	0.0323	18	211	225	MVADLEDAYILLHEK				Mascot
1775.8884	1775.9236	0.0352	20	211	225	MVADLEDAYILLHEK			Oxidation (M)[1]	Mascot
1853.8585	1853.8862	0.0277	15	182	197	TAETELEVVEGMQFDR	148	100		Mascot

Calc. Mass	Obsrv. Mass	± da	± ppm	Start Seq.	End Seq.	Sequence	Ion Score	C. I. %	Modification	Rank	Result Type
1853.8585	1853.8862	0.0277	15	182	197	TAETELEVVEGMQFDR					Mascot
1869.0188	1868.9811	-0.0377	-20	426	443	ITAKGVNADQEAGINIVR					Mascot
1869.8535	1869.8912	0.0377	20	182	197	TAETELEVVEGMQFDR			Oxidation (M)[12]		Mascot
1887.9884	1887.9702	-0.0182	-10	211	226	MVADLEDAYILLHEKK					Mascot
1903.9834	1903.9958	0.0124	7	211	226	MVADLEDAYILLHEKK			Oxidation (M)[1]		Mascot
2038.9572	2038.9861	0.0289	14	59	75	EVELEDKFENMGAQMLR					Mascot
2054.9521	2054.9966	0.0445	22	59	75	EVELEDKFENMGAQMLR			Oxidation (M)[11]		Mascot
2070.947	2070.9927	0.0457	22	59	75	EVELEDKFENMGAQMLR			Oxidation (M)[11,15]		Mascot
2286.2261	2286.2593	0.0332	15	287	308	AMLEDIAILTGGQVISEDL GIK					Mascot
2302.2209	2302.2488	0.0279	12	287	308	AMLEDIAILTGGQVISEDL GIK			Oxidation (M)[2]		Mascot
2317.1516	2317.1951	0.0435	19	138	160	INTSEEVAQVGTISANGE AEIGK					Mascot
2419.1946	2419.2534	0.0588	24	81	105	TNDTAGDGTTTATVLGQ AIVQEGAK					Mascot
2445.2466	2445.2927	0.0461	19	137	160	KINTSEEVAQVGTISANG EAEIGK					Mascot
2526.3848	2526.4192	0.0344	14	502	526	TALQNAASVAGLLITTEA MIAELPK					Mascot
3329.6423	3329.7024	0.0601	18	468	498	ILENTSETFGYNTANGEY GDLISLGIVDPVK					Mascot

2 Random sequence, was unnamed protein product [Brucella suis 1330] ###RND###gi|23501509|ref|NP_697636.1| 12 60 98.539 .T. AlDahouk_6214_Z19769-23_T1 417

Peptide Information

Calc. Mass	Obsrv. Mass	± da	± ppm	Start Seq.	End Seq.	Sequence	Ion Score	C. I. %	Modification	Rank	Result Type
704.4124	704.3825	-0.0299	-42	198	203	MITALR					Mascot
719.3682	719.3406	-0.0276	-38	2	8	SISEAGR					Mascot
875.4693	875.457	-0.0123	-14	1	8	RSISEAGR					Mascot
1000.5897	1000.5744	-0.0153	-15	23	31	RISAAVDIR					Mascot
1348.6313	1348.717	0.0857	64	34	46	GFNEAVPGAEAMR					Mascot
1405.6852	1405.7218	0.0366	26	2	14	SISEAGRMDVNAR					Mascot
1469.8872	1469.7833	-0.1039	-71	90	102	QASILKLPLIMAR			Oxidation (M)[11]		Mascot
1724.8887	1724.7943	-0.0944	-55	211	227	VYGLGSLNSVTMVAGEK					Mascot
1868.0177	1867.918	-0.0997	-53	305	320	QLVAIHYHFEGLALTR					Mascot
1868.9785	1868.9811	0.0026	1	210	227	KVYGLGSLNSVTMVAGE K			Oxidation (M)[13]		Mascot
2038.975	2038.9861	0.0111	5	52	70	VKFGGEAATLEGLMEEG ER			Oxidation (M)[14]		Mascot
2526.335	2526.4192	0.0842	33	172	194	FGEKAPAFSAVITLLPHLD TENR					Mascot

Gel Idx/Pos	201/I1	Instr./Gel Origin	AK043/Div_120507	Process Status	Analysis Succeeded
Plate [#] Name	[1] 1300017700	Instrument Sample Name		Spectra	4

Rank	Protein Name	Accession No.	Pep. Count	Protein Score	Protein Score C. I. %	Total Ion Score	Best Ion Score	Best Ion C. I. %	Total Ion C. I. %	Confirmed	Sample Name	Customer sample Name
1	chaperonin GroEL [Brucella suis ATCC 23445]	gi|163844383|ref|YP_001622038.1|	28	588	100	390	137	100	100	.T.	AlDahouk_6214_Z19769-24_T1	435

Peptide Information

Calc. Mass	Obsrv. Mass	± da	± ppm	Start Seq.	End Seq.	Sequence	Ion Score	C. I. %	Modification	Rank	Result Type
707.3947	707.3837	-0.011	-16	8	13	FGRTAR					Mascot
719.347	719.3701	0.0231	32	278	284	APGFGDR					Mascot
721.3991	721.3746	-0.0245	-34	5	10	DVKFGR					Mascot
726.4257	726.4484	0.0227	31	445	451	AIQAPAR					Mascot
762.4178	762.4395	0.0217	28	1	7	MAAKDVK					Mascot
778.4127	778.3975	-0.0152	-20	1	7	MAAKDVK			Oxidation (M)[1]		Mascot
855.541	855.5846	0.0436	51	372	380	LAGGVAVIR					Mascot
875.4482	875.4948	0.0466	53	278	285	APGFGDRR					Mascot
882.5268	882.5742	0.0474	54	444	451	RAIQAPAR					Mascot
974.4901	974.5585	0.0684	70	396	404	VDDALNATR					Mascot
1000.5673	1000.6149	0.0476	48	19	28	GVDILADAVK					Mascot
1233.6508	1233.7137	0.0629	51	309	319	LESVTLDMLGR					Mascot
1245.6685	1245.6865	0.018	14	381	392	VGGATEVEVKEK					Mascot
1249.6456	1249.7112	0.0656	52	309	319	LESVTLDMLGR			Oxidation (M)[8]		Mascot
1344.7004	1344.7697	0.0693	52	169	181	VGNEGVITVEEAK					Mascot
1373.7028	1373.7836	0.0808	59	106	118	AVAAGMNPMDLKR					Mascot
1405.6926	1405.771	0.0784	56	106	118	AVAAGMNPMDLKR			Oxidation (M)[6,9]		Mascot
1455.755	1455.8346	0.0796	55	430	443	GVNADQEAGINIVR	128	100			Mascot
1455.755	1455.8346	0.0796	55	430	443	GVNADQEAGINIVR					Mascot
1514.7526	1514.839	0.0864	57	198	210	GYLSPYFVTNPEK					Mascot
1582.905	1582.9891	0.0841	53	119	133	GIDLAVNEVVAELLK					Mascot
1583.8751	1583.9641	0.089	56	405	421	AAVEEGIVAGGGTALLR	137	100			Mascot
1583.8751	1583.9641	0.089	56	405	421	AAVEEGIVAGGGTALLR					Mascot
1711	1711.0974	0.0974	57	119	134	GIDLAVNEVVAELLKK					Mascot

1741.7875	1741.886	0.0985	57	351	364	QQIEETTSDYDREK				Mascot
1759.8934	1759.9954	0.102	58	211	225	MVADLEDAYILLHEK				Mascot
1775.8884	1775.9933	0.1049	59	211	225	MVADLEDAYILLHEK			Oxidation (M)[1]	Mascot
1853.8585	1853.9655	0.107	58	182	197	TAETELEVVEGMQFDR	125	100		Mascot
1853.8585	1853.9655	0.107	58	182	197	TAETELEVVEGMQFDR				Mascot
1869.8535	1869.9675	0.114	61	182	197	TAETELEVVEGMQFDR			Oxidation (M)[12]	Mascot
1903.9834	1904.1061	0.1227	64	211	226	MVADLEDAYILLHEKK			Oxidation (M)[1]	Mascot
2038.9572	2039.0739	0.1167	57	59	75	EVELEDKFENMGAQMLR				Mascot
2054.9521	2055.0784	0.1263	61	59	75	EVELEDKFENMGAQMLR			Oxidation (M)[11]	Mascot
2070.947	2071.0718	0.1248	60	59	75	EVELEDKFENMGAQMLR			Oxidation (M)[11,15]	Mascot
2317.1516	2317.2412	0.0896	39	138	160	INTSEEVAQVGTISANGE AEIGK				Mascot
2419.1946	2419.3372	0.1426	59	81	105	TNDTAGDGTTTATVLGQ AIVQEGAK				Mascot
2445.2466	2445.3884	0.1418	58	137	160	KINTSEEVAQVGTISANG EAEIGK				Mascot
3329.6423	3329.8467	0.2044	61	468	498	ILENTSETFGYNTANGEY GDLISLGIVDPVK				Mascot

2 pckA gene product [Brucella suis 1330] gi|23502937|ref|NP_699064.1| 16 80 99.985 .T. AlDahouk_6214_Z19769-24_T1 435

Peptide Information

Calc. Mass	Obsrv. Mass	± da	± ppm	Start Seq.	End Seq.	Sequence	Ion Score	C. I. %	Modification	Rank	Result Type
707.3359	707.3837	0.0478	68	490	495	STWADK					Mascot
756.3747	756.4128	0.0381	50	55	61	TGQHTGR					Mascot
758.4155	758.4293	0.0138	18	336	343	SGKGGQPK					Mascot
763.446	763.4443	-0.0017	-2	65	70	DKFVVR					Mascot
865.4413	865.46	0.0187	22	496	503	VAYDAQAK					Mascot
923.4832	923.5256	0.0424	46	33	39	LYEETIR					Mascot
1079.5844	1079.5868	0.0024	2	33	40	LYEETIRR					Mascot
1185.6011	1185.6705	0.0694	59	405	414	HPSEYGNLLR					Mascot
1344.6504	1344.7697	0.1193	89	505	515	LVDMFVSNFEK			Oxidation (M)[4]		Mascot
1436.8148	1436.8394	0.0246	17	202	214	SVFTALNYILPAK					Mascot
1527.8237	1527.8723	0.0486	32	40	54	RGEAELSAQGALVAR					Mascot
1582.8508	1582.9891	0.1383	87	187	201	IVLIGGTSYAGEMKK			Oxidation (M)[13]		Mascot
1634.8689	1634.9562	0.0873	53	126	138	VITEYAWHSLFIR					Mascot
1678.8282	1678.9304	0.1022	61	280	294	LSAEAEPEIYATTQR					Mascot
1745.9181	1746.004	0.0859	49	449	465	ALLAAALDGSLNNAEFR					Mascot

1934.0126	1934.0745	0.0619	32	344	361	NIIMLTADAFGVMPPIAK	Oxidation (M)[4,13]		Mascot

Gel Idx/Pos	202/I2	Instr./Gel Origin	AK043/Div_120507	Process Status	Analysis Succeeded
Plate [#] Name	[1] 1300017700	Instrument Sample Name		Spectra	4

Rank	Protein	Name	Accession No.	Pep. Count	Protein Score	Protein Score C. I. %	Total Ion Score	Best Ion Score	Best Ion C. I. %	Total Ion C. I. %	Confirmed	Sample Name	Customer sample Name
1	chaperonin GroEL [Brucella suis ATCC 23445]		gi|163844383|ref|YP_001622038.1|	35	695	100	397	140	100	100	.T.	AlDahouk_6214_Z19769-25_T1	425

Peptide Information

Calc. Mass	Obsrv. Mass	± da	± ppm	Start Seq.	End Seq.	Sequence	Ion Score	C. I. %	Modification	Rank	Result Type
707.3947	707.3907	-0.004	-6	8	13	FGRTAR					Mascot
719.347	719.378	0.031	43	278	284	APGFGDR					Mascot
726.4257	726.4564	0.0307	42	445	451	AIQAPAR					Mascot
855.541	855.5939	0.0529	62	372	380	LAGGVAVIR					Mascot
875.4482	875.4996	0.0514	59	278	285	APGFGDRR					Mascot
882.5268	882.5798	0.053	60	444	451	RAIQAPAR					Mascot
921.4532	921.5023	0.0491	53	161	168	MIAEAMQK					Mascot
937.4481	937.5074	0.0593	63	161	168	MIAEAMQK			Oxidation (M)[1]		Mascot
953.443	953.5021	0.0591	62	161	168	MIAEAMQK			Oxidation (M)[1,6]		Mascot
974.4901	974.5486	0.0585	60	396	404	VDDALNATR					Mascot
988.5309	988.5893	0.0584	59	381	390	VGGATEVEVK					Mascot
1000.5673	1000.6237	0.0564	56	19	28	GVDILADAVK					Mascot
1232.6117	1232.6876	0.0759	62	328	339	ENTTIVDGAGQK					Mascot
1233.6508	1233.7239	0.0731	59	309	319	LESVTLDMLGR					Mascot
1245.6685	1245.6969	0.0284	23	381	392	VGGATEVEVKEK					Mascot
1249.6456	1249.7196	0.074	59	309	319	LESVTLDMLGR			Oxidation (M)[8]		Mascot
1344.7004	1344.7816	0.0812	60	169	181	VGNEGVITVEEAK					Mascot
1373.7028	1373.7926	0.0898	65	106	118	AVAAGMNPMDLKR					Mascot
1389.6978	1389.7848	0.087	63	106	118	AVAAGMNPMDLKR			Oxidation (M)[6]		Mascot
1405.6926	1405.782	0.0894	64	106	118	AVAAGMNPMDLKR			Oxidation (M)[6,9]		Mascot
1455.755	1455.845	0.09	62	430	443	GVNADQEAGINIVR	134	100			Mascot
1455.755	1455.845	0.09	62	430	443	GVNADQEAGINIVR					Mascot
1484.65	1484.7445	0.0945	64	351	362	QQIEETTSDYDR					Mascot

1514.7526	1514.8462	0.0936	62	198	210	GYLSPYFVTNPEK				Mascot
1582.905	1583.002	0.097	61	119	133	GIDLAVNEVVAELLK				Mascot
1583.8751	1583.9757	0.1006	64	405	421	AAVEEGIVAGGGTALLR	140	100		Mascot
1583.8751	1583.9757	0.1006	64	405	421	AAVEEGIVAGGGTALLR				Mascot
1611.8561	1611.962	0.1059	66	430	444	GVNADQEAGINIVRR				Mascot
1616.849	1616.9496	0.1006	62	452	467	QITTNAGEEASVIVGK				Mascot
1711	1711.106	0.106	62	119	134	GIDLAVNEVVAELLKK				Mascot
1739.0061	1739.1139	0.1078	62	118	133	RGIDLAVNEVVAELLK				Mascot
1741.7875	1741.8983	0.1108	64	351	364	QQIEETTSDYDREK				Mascot
1759.8934	1760.001	0.1076	61	211	225	MVADLEDAYILLHEK				Mascot
1775.8884	1775.9982	0.1098	62	211	225	MVADLEDAYILLHEK			Oxidation (M)[1]	Mascot
1853.8585	1853.9714	0.1129	61	182	197	TAETELEVVEGMQFDR	123	100		Mascot
1853.8585	1853.9714	0.1129	61	182	197	TAETELEVVEGMQFDR				Mascot
1869.8535	1869.9707	0.1172	63	182	197	TAETELEVVEGMQFDR			Oxidation (M)[12]	Mascot
1887.9884	1888.0359	0.0475	25	211	226	MVADLEDAYILLHEKK				Mascot
1903.9834	1904.103	0.1196	63	211	226	MVADLEDAYILLHEKK			Oxidation (M)[1]	Mascot
2038.9572	2039.079	0.1218	60	59	75	EVELEDKFENMGAQMLR				Mascot
2054.9521	2055.0813	0.1292	63	59	75	EVELEDKFENMGAQMLR			Oxidation (M)[11]	Mascot
2070.947	2071.0823	0.1353	65	59	75	EVELEDKFENMGAQMLR			Oxidation (M)[11,15]	Mascot
2286.2261	2286.3679	0.1418	62	287	308	AMLEDIAILTGGQVISEDL GIK				Mascot
2302.2209	2302.3621	0.1412	61	287	308	AMLEDIAILTGGQVISEDL GIK			Oxidation (M)[2]	Mascot
2317.1516	2317.2981	0.1465	63	138	160	INTSEEVAQVGTISANGE AEIGK				Mascot
2419.1946	2419.3545	0.1599	66	81	105	TNDTAGDGTTTATVLGQ AIVQEGAK				Mascot
2445.2466	2445.4014	0.1548	63	137	160	KINTSEEVAQVGTISANG EAEIGK				Mascot
2542.3796	2542.532	0.1524	60	502	526	TALQNAASVAGLLITTEA MIAELPK			Oxidation (M)[19]	Mascot
3329.6423	3329.8496	0.2073	62	468	498	ILENTSETFGYNTANGEY GDLISLGIVDPVK				Mascot

**Gel Idx/Pos**	203/I3	**Instr./Gel Origin**	AK043/Div_120507	**Process Status**	Analysis Succeeded
**Plate [#] Name**	[1] 1300017700	**Instrument Sample Name**		**Spectra**	4

Rank	Protein Name	Accession No.	Pep. Count	Protein Score	Protein Score C. I. %	Total Ion Score	Best Ion Score	Best Ion C. I. %	Total Ion C. I. %	Confirmed	Sample Name	Customer sample Name
1	chaperonin GroEL [Brucella suis ATCC 23445]	gi|163844383|ref|YP_001622038.1|	34	692	100	411	143	100	100	.T.	AlDahouk_6214_Z19769-26_T1	420

Peptide Information

Calc. Mass	Obsrv. Mass	± da	± ppm	Start Seq.	End Seq.	Sequence	Ion Score	C. I. %	Modification	Rank	Result Type
707.3947	707.4081	0.0134	19	8	13	FGRTAR					Mascot
719.347	719.3806	0.0336	47	278	284	APGFGDR					Mascot
726.4257	726.4581	0.0324	45	445	451	AIQAPAR					Mascot
855.541	855.5967	0.0557	65	372	380	LAGGVAVIR					Mascot
875.4482	875.5042	0.056	64	278	285	APGFGDRR					Mascot
882.5268	882.5854	0.0586	66	444	451	RAIQAPAR					Mascot
937.4481	937.5097	0.0616	66	161	168	MIAEAMQK			Oxidation (M)[1]		Mascot
974.4901	974.5542	0.0641	66	396	404	VDDALNATR					Mascot
1000.5673	1000.6273	0.06	60	19	28	GVDILADAVK					Mascot
1232.6117	1232.6952	0.0835	68	328	339	ENTTIVDGAGQK					Mascot
1233.6508	1233.7299	0.0791	64	309	319	LESVTLDMLGR					Mascot
1245.6685	1245.704	0.0355	28	381	392	VGGATEVEVKEK					Mascot
1249.6456	1249.7283	0.0827	66	309	319	LESVTLDMLGR			Oxidation (M)[8]		Mascot
1344.7004	1344.7909	0.0905	67	169	181	VGNEGVITVEEAK					Mascot
1373.7028	1373.7991	0.0963	70	106	118	AVAAGMNPMDLKR					Mascot
1389.6978	1389.7811	0.0833	60	106	118	AVAAGMNPMDLKR			Oxidation (M)[6]		Mascot
1405.6926	1405.7916	0.099	70	106	118	AVAAGMNPMDLKR			Oxidation (M)[6,9]		Mascot
1455.755	1455.8534	0.0984	68	430	443	GVNADQEAGINIVR	134	100			Mascot
1455.755	1455.8534	0.0984	68	430	443	GVNADQEAGINIVR					Mascot
1484.65	1484.749	0.099	67	351	362	QQIEETTSDYDR					Mascot
1514.7526	1514.8595	0.1069	71	198	210	GYLSPYFVTNPEK					Mascot
1582.905	1583.0148	0.1098	69	119	133	GIDLAVNEVVAELLK					Mascot
1583.8751	1583.9875	0.1124	71	405	421	AAVEEGIVAGGGTALLR	143	100			Mascot
1583.8751	1583.9875	0.1124	71	405	421	AAVEEGIVAGGGTALLR					Mascot
1611.8561	1611.9735	0.1174	73	430	444	GVNADQEAGINIVRR					Mascot
1616.849	1616.9619	0.1129	70	452	467	QITTNAGEEASVIVGK					Mascot
1711	1711.1171	0.1171	68	119	134	GIDLAVNEVVAELLKK					Mascot
1739.0061	1739.1246	0.1185	68	118	133	RGIDLAVNEVVAELLK					Mascot
1741.7875	1741.9143	0.1268	73	351	364	QQIEETTSDYDREK					Mascot
1759.8934	1760.0153	0.1219	69	211	225	MVADLEDAYILLHEK					Mascot
1775.8884	1776.0162	0.1278	72	211	225	MVADLEDAYILLHEK			Oxidation (M)[1]		Mascot
1853.8585	1853.9871	0.1286	69	182	197	TAETELEVVEGMQFDR	134	100			Mascot

1853.8585	1853.9871	0.1286	69	182	197	TAETELEVVEGMQFDR				Mascot
1869.8535	1869.9906	0.1371	73	182	197	TAETELEVVEGMQFDR		Oxidation (M)[12]		Mascot
1903.9834	1904.0756	0.0922	48	211	226	MVADLEDAYILLHEKK		Oxidation (M)[1]		Mascot
2038.9572	2039.0966	0.1394	68	59	75	EVELEDKFENMGAQMLR				Mascot
2054.9521	2055.1042	0.1521	74	59	75	EVELEDKFENMGAQMLR		Oxidation (M)[11]		Mascot
2070.947	2071.0977	0.1507	73	59	75	EVELEDKFENMGAQMLR		Oxidation (M)[11,15]		Mascot
2286.2261	2286.3845	0.1584	69	287	308	AMLEDIAILTGGQVISEDL GIK				Mascot
2302.2209	2302.3818	0.1609	70	287	308	AMLEDIAILTGGQVISEDL GIK		Oxidation (M)[2]		Mascot
2317.1516	2317.3269	0.1753	76	138	160	INTSEEVAQVGTISANGE AEIGK				Mascot
2419.1946	2419.3813	0.1867	77	81	105	TNDTAGDGTTTATVLGQ AIVQEGAK				Mascot
2445.2466	2445.4114	0.1648	67	137	160	KINTSEEVAQVGTISANG EAEIGK				Mascot
2526.3848	2526.5632	0.1784	71	502	526	TALQNAASVAGLLITTEA MIAELPK				Mascot
2542.3796	2542.5574	0.1778	70	502	526	TALQNAASVAGLLITTEA MIAELPK		Oxidation (M)[19]		Mascot
3329.6423	3329.8831	0.2408	72	468	498	ILENTSETFGYNTANGEY GDLISLGIVDPVK				Mascot

2 unnamed protein product [Brucella suis 1330] gi|23501498|ref|NP_697625.1| 15 79 99.982 .T. AlDahouk_6214_Z19769-26_T1 420

Peptide Information

Calc. Mass	Obsrv. Mass	± da	± ppm	Start Seq.	End Seq.	Sequence	Ion Score	C. I. %	Modification	Rank	Result Type
703.3984	703.4177	0.0193	27	481	487	AITAAEK					Mascot
741.4253	741.4562	0.0309	42	174	180	LIGADPR					Mascot
816.4726	816.5179	0.0453	55	384	390	AALTVWR					Mascot
851.3716	851.4408	0.0692	81	104	110	DFGMEPR					Mascot
867.3665	867.4206	0.0541	62	104	110	DFGMEPR			Oxidation (M)[4]		Mascot
982.6155	982.6768	0.0613	62	60	68	VRPAVVSVR					Mascot
1274.6051	1274.6935	0.0884	69	195	205	FVYVAFGDDNK					Mascot
1325.7576	1325.8457	0.0881	66	310	321	GWIGVQIQPVTK					Mascot
1471.7864	1471.8804	0.094	64	492	504	AVLLQLQSNDQSR					Mascot
1529.7747	1529.8851	0.1104	72	195	207	FVYVAFGDDNKVR					Mascot
1599.8813	1599.9832	0.1019	64	491	504	KAVLLQLQSNDQSR					Mascot
1741.8715	1741.9143	0.0428	25	353	369	AGDVITAVNGETVQDPR					Mascot
1854.0232	1853.9871	-0.0361	-19	305	321	GSVERGWIGVQIQPVTK					Mascot
1854.0232	1853.9871	-0.0361	-19	305	321	GSVERGWIGVQIQPVTK					Mascot

2021.9814	2022.1123	0.1309	65	236	254	DIGAGPYDDFIQIDAAVN K					Mascot
2307.167	2307.3201	0.1531	66	80	100	GPQFFGPPGFDQLPDGH PLKR					Mascot
2515.3303	2515.4858	0.1555	62	208	233	VGDWVVAVGNPFGLGG TVTSGIVSAR					Mascot

Rank	Protein Name	Accession No.	Pep. Count	Protein Score	Protein Score C. I. %	Total Ion Score	Best Ion Score	Best Ion C. I. %	Total Ion C. I. %	Confirmed	Sample Name	Customer sample Name
3	unnamed protein product [Brucella suis 1330]	gi|23501912|ref|NP_698039.1|	11	60	98.698					.T.	AlDahouk_6214_Z19769-26_T1	420

Peptide Information

Calc. Mass	Obsrv. Mass	± da	± ppm	Start Seq.	End Seq.	Sequence	Ion Score	C. I. %	Modification	Rank	Result Type
703.3733	703.4177	0.0444	63	2	7	SDKTPR					Mascot
705.3678	705.4103	0.0425	60	72	77	GWSLSR					Mascot
713.394	713.3816	-0.0124	-17	14	19	LEQPAR					Mascot
719.4199	719.3806	-0.0393	-55	8	13	KPTAFR					Mascot
726.4733	726.4581	-0.0152	-21	31	36	RPRAVK					Mascot
834.4138	834.3671	-0.0467	-56	1	7	MSDKTPR					Mascot
1611.8197	1611.9735	0.1538	95	20	33	VSAASEQEEPRRPR					Mascot
1897.0442	1896.9958	-0.0484	-26	251	267	RLSQLYGGRPGTFGFIK					Mascot
1991.0419	1991.0983	0.0564	28	318	335	IGIAAMDVVRPFPFNAEK			Oxidation (M)[6]		Mascot
2071.103	2071.0977	-0.0053	-3	182	199	QLLDSLTDDIIDGRDLIR					Mascot
3329.8362	3329.8831	0.0469	14	272	304	VIAHLAVTGTIAMGDSVIQ QLVGHGLASRLSAK			Oxidation (M)[13]		Mascot

Gel Idx/Pos	204/I4	Instr./Gel Origin	AK043/Div_120507	Process Status	Analysis Succeeded
Plate [#] Name	[1] 1300017700	Instrument Sample Name		Spectra	4

Rank	Protein Name	Accession No.	Pep. Count	Protein Score	Protein Score C. I. %	Total Ion Score	Best Ion Score	Best Ion C. I. %	Total Ion C. I. %	Confirmed	Sample Name	Customer sample Name
1	unnamed protein product [Brucella suis 1330]	gi|23500285|ref|NP_699725.1|	24	467	100	296	136	100	100	.T.	AlDahouk_6214_Z19769-27_T1	494

Peptide Information

Calc. Mass	Obsrv. Mass	± da	± ppm	Start Seq.	End Seq.	Sequence	Ion Score	C. I. %	Modification	Rank	Result Type
759.4182	759.4421	0.0239	31	110	115	RLMDPK					Mascot
775.413	775.4618	0.0488	63	110	115	RLMDPK			Oxidation (M)[3]		Mascot
818.4003	818.4521	0.0518	63	466	472	TQDLGER					Mascot

	1004.5193	1004.5814	0.0621	62	301	309	QAISMAIDR							Mascot		
	1019.4792	1019.5521	0.0729	72	415	423	EDGPFDIAR							Mascot		
	1175.5844	1175.6658	0.0814	69	230	238	VNWIPFEDR	55	99.998					Mascot		
	1175.5844	1175.6658	0.0814	69	230	238	VNWIPFEDR							Mascot		
	1256.5464	1256.6393	0.0929	74	342	351	LDYSDDMLER							Mascot		
	1272.5413	1272.6326	0.0913	72	342	351	LDYSDDMLER			Oxidation (M)[7]				Mascot		
	1385.7498	1385.8258	0.076	55	116	128	TAAGYASMLFIIK							Mascot		
	1389.6321	1389.7354	0.1033	74	216	226	NPYYYEADQVK							Mascot		
	1401.7446	1401.8418	0.0972	69	116	128	TAAGYASMLFIIK			Oxidation (M)[8]				Mascot		
	1513.8124	1513.9185	0.1061	70	274	286	MAPYLGVYYLPVK							Mascot		
	1517.7271	1517.8486	0.1215	80	215	226	KNPYYYEADQVK							Mascot		
	1523.7224	1523.8357	0.1133	74	52	65	DLYDGLTIQDADGK							Mascot		
	1529.8073	1529.9138	0.1065	70	274	286	MAPYLGVYYLPVK			Oxidation (M)[1]				Mascot		
	1628.7108	1628.8365	0.1257	77	342	354	LDYSDDMLEREDK							Mascot		
	1629.8629	1629.9153	0.0524	32	383	398	NTMAAIADQLGNIGIK							Mascot		
	1644.7058	1644.844	0.1382	84	342	354	LDYSDDMLEREDK			Oxidation (M)[7]				Mascot		
	1645.8578	1645.9303	0.0725	44	383	398	NTMAAIADQLGNIGIK			Oxidation (M)[3]				Mascot		
	1660.7965	1660.9152	0.1187	71	401	414	LNETEGASYFNFLR	136	100					Mascot		
	1660.7965	1660.9152	0.1187	71	401	414	LNETEGASYFNFLR							Mascot		
	1863.9811	1864.1255	0.1444	77	138	154	KPVDQLGVEAVDDHTLK							Mascot		
	1925.9028	1926.0403	0.1375	71	73	88	SWDISEDGTVYTFHLR	104	100					Mascot		
	1925.9028	1926.0403	0.1375	71	73	88	SWDISEDGTVYTFHLR							Mascot		
	1944.8875	1945.0259	0.1384	71	93	109	WSNGDPVTAGDFQFTFR							Mascot		
	1946.0244	1946.0216	-0.0028	-1	271	286	EFRMAPYLGVYYLPVK							Mascot		
	2026.9097	2027.0844	0.1747	86	249	265	EVQICSDVPAEQMDYVK			Carbamidomethyl (C)[5], Oxidation (M)[13]				Mascot		
	2435.1936	2435.3821	0.1885	77	361	382	EAGVEPNTLSIELLYNTS ENNK							Mascot		
	2557.2026	2557.3945	0.1919	75	28	51	GNDTDPATLDHHHTSTV AEGNVLR							Mascot		
	2661.2578	2661.4482	0.1904	72	401	423	LNETEGASYFNFLREDG PFDIAR							Mascot		
	2865.5298	2865.7312	0.2014	70	155	179	ITLNSPAPYFLELLTHQTG FPLHQK							Mascot		
	3111.4482	3111.6719	0.2237	72	424	450	ASWIGDYNDPQNFLYISQ GNVSFNYAK							Mascot		
2	hypothetical protein BSUIS_B0534 [Brucella suis ATCC 23445]	gi|163844697|ref|YP_001622352.1|	20	420	100	296	136	100	100	.T.	AlDahouk_6214_Z19769-27_T1	494				

Peptide Information

Calc. Mass	Obsrv. Mass	± da	± ppm	Start	End	Sequence	Ion	C. I. %	Modification	Rank	Result Type

				Seq.	Seq.		Score				
759.4182	759.4421	0.0239	31	110	115	RLMDPK					Mascot
775.413	775.4618	0.0488	63	110	115	RLMDPK			Oxidation (M)[3]		Mascot
818.4003	818.4521	0.0518	63	466	472	TQDLGER					Mascot
1004.5193	1004.5814	0.0621	62	301	309	QAISMAIDR					Mascot
1175.5844	1175.6658	0.0814	69	230	238	VNWIPFEDR	55	99.998			Mascot
1175.5844	1175.6658	0.0814	69	230	238	VNWIPFEDR					Mascot
1256.5464	1256.6393	0.0929	74	342	351	LDYSDDMLER					Mascot
1272.5413	1272.6326	0.0913	72	342	351	LDYSDDMLER			Oxidation (M)[7]		Mascot
1389.6321	1389.7354	0.1033	74	216	226	NPYYYEADQVK					Mascot
1513.8124	1513.9185	0.1061	70	274	286	MAPYLGVYYLPVK					Mascot
1517.7271	1517.8486	0.1215	80	215	226	KNPYYYEADQVK					Mascot
1523.7224	1523.8357	0.1133	74	52	65	DLYDGLTIQDADGK					Mascot
1529.8073	1529.9138	0.1065	70	274	286	MAPYLGVYYLPVK			Oxidation (M)[1]		Mascot
1628.7108	1628.8365	0.1257	77	342	354	LDYSDDMLEREDK					Mascot
1629.8629	1629.9153	0.0524	32	383	398	NTMAAIADQLGNIGIK					Mascot
1644.7058	1644.844	0.1382	84	342	354	LDYSDDMLEREDK			Oxidation (M)[7]		Mascot
1645.8578	1645.9303	0.0725	44	383	398	NTMAAIADQLGNIGIK			Oxidation (M)[3]		Mascot
1660.7965	1660.9152	0.1187	71	401	414	LNETEGASYFNFLR	136	100			Mascot
1660.7965	1660.9152	0.1187	71	401	414	LNETEGASYFNFLR					Mascot
1863.9811	1864.1255	0.1444	77	138	154	KPVDQLGVEAVDDHTLK					Mascot
1925.9028	1926.0403	0.1375	71	73	88	SWDISEDGTVYTFHLR	104	100			Mascot
1925.9028	1926.0403	0.1375	71	73	88	SWDISEDGTVYTFHLR					Mascot
1946.0244	1946.0216	-0.0028	-1	271	286	EFRMAPYLGVYYLPVK					Mascot
2026.9097	2027.0844	0.1747	86	249	265	EVQICSDVPAEQMDYVK			Carbamidomethyl (C)[5], Oxidation (M)[13]		Mascot
2435.1936	2435.3821	0.1885	77	361	382	EAGVEPNTLSIELLYNTS ENNK					Mascot
2557.2026	2557.3945	0.1919	75	28	51	GNDTDPATLDHHHTSTV AEGNVLR					Mascot
2865.5298	2865.7312	0.2014	70	155	179	ITLNSPAPYFLELLTHQTG FPLHQK					Mascot
3111.4482	3111.6719	0.2237	72	424	450	ASWIGDYNDPQNFLYISQ GNVSFNYAK					Mascot

3 chaperonin GroEL [Brucella suis ATCC 23445] gi|163844383|ref|YP_001622038.1| 25 151 100 .T. AlDahouk_6214_Z19769-27_T1 494

Peptide Information

Calc. Mass	Obsrv. Mass	± da	± ppm	Start Seq.	End Seq.	Sequence	Ion Score	C. I. %	Modification	Rank	Result Type

707.3947	707.3786	-0.0161	-23	8	13	FGRTAR		Mascot
719.347	719.3826	0.0356	49	278	284	APGFGDR		Mascot
721.3991	721.3918	-0.0073	-10	5	10	DVKFGR		Mascot
726.4257	726.4611	0.0354	49	445	451	AIQAPAR		Mascot
855.541	855.5997	0.0587	69	372	380	LAGGVAVIR		Mascot
875.4482	875.5078	0.0596	68	278	285	APGFGDRR		Mascot
882.5268	882.5967	0.0699	79	444	451	RAIQAPAR		Mascot
974.4901	974.5614	0.0713	73	396	404	VDDALNATR		Mascot
1233.6508	1233.7339	0.0831	67	309	319	LESVTLDMLGR		Mascot
1245.6685	1245.7089	0.0404	32	381	392	VGGATEVEVKEK		Mascot
1249.6456	1249.7325	0.0869	70	309	319	LESVTLDMLGR	Oxidation (M)[8]	Mascot
1344.7004	1344.7948	0.0944	70	169	181	VGNEGVITVEEAK		Mascot
1389.6978	1389.7354	0.0376	27	106	118	AVAAGMNPMDLKR	Oxidation (M)[6]	Mascot
1455.755	1455.8568	0.1018	70	430	443	GVNADQEAGINIVR		Mascot
1484.65	1484.7611	0.1111	75	351	362	QQIEETTSDYDR		Mascot
1514.7526	1514.8798	0.1272	84	198	210	GYLSPYFVTNPEK		Mascot
1517.7693	1517.8486	0.0793	52	52	65	DGVSVAKEVELEDK		Mascot
1582.905	1583.0165	0.1115	70	119	133	GIDLAVNEVVAELLK		Mascot
1583.8751	1583.993	0.1179	74	405	421	AAVEEGIVAGGGTALLR		Mascot
1616.849	1616.9519	0.1029	64	452	467	QITTNAGEEASVIVGK		Mascot
1741.7875	1741.9178	0.1303	75	351	364	QQIEETTSDYDREK		Mascot
1759.8934	1760.019	0.1256	71	211	225	MVADLEDAYILLHEK		Mascot
1775.8884	1776.0232	0.1348	76	211	225	MVADLEDAYILLHEK	Oxidation (M)[1]	Mascot
1853.8585	1853.9945	0.136	73	182	197	TAETELEVVEGMQFDR		Mascot
1869.8535	1869.9968	0.1433	77	182	197	TAETELEVVEGMQFDR	Oxidation (M)[12]	Mascot
1903.9834	1904.1288	0.1454	76	211	226	MVADLEDAYILLHEKK	Oxidation (M)[1]	Mascot
2038.9572	2039.0992	0.142	70	59	75	EVELEDKFENMGAQMLR		Mascot
2054.9521	2055.155	0.2029	99	59	75	EVELEDKFENMGAQMLR	Oxidation (M)[11]	Mascot
2070.947	2071.1033	0.1563	75	59	75	EVELEDKFENMGAQMLR	Oxidation (M)[11,15]	Mascot
2419.1946	2419.3813	0.1867	77	81	105	TNDTAGDGTTTATVLGQ AIVQEGAK		Mascot

Gel Idx/Pos<br>Plate [#] Name	205/I5<br>[1] 1300017700	Instr./Gel Origin<br>Instrument Sample Name	AK043/Div_120507	Process Status<br>Spectra	Analysis Succeeded<br>4

Rank	Protein	Name	Accession No.	Pep. Count	Protein Score	Protein Score C. I. %	Total Ion Score	Best Ion Score	Best Ion C. I. %	Total Ion C. I. %	Confirmed	Sample Name	Customer sample Name
1	trkA gene product [Brucella suis 1330]		gi|23501992|ref|NP_698119.1|	27	414	100	199	93	100	100	.T.	AlDahouk_6214_Z19769-28_T1	482

Peptide Information

Calc. Mass	Obsrv. Mass	± da	± ppm	Start Seq.	End Seq.	Sequence	Ion Score	C. I. %	Modification	Rank	Result Type
709.3879	709.3927	0.0048	7	101	106	AQSYLK					Mascot
718.373	718.4089	0.0359	50	41	46	DTLDVR					Mascot
728.3474	728.3876	0.0402	55	256	260	EWHTR					Mascot
741.3817	741.444	0.0623	84	453	458	VSLEFF					Mascot
744.425	744.4522	0.0272	37	35	40	DIEIVR					Mascot
765.473	765.5267	0.0537	70	362	367	ILQHVR					Mascot
946.4563	946.5287	0.0724	76	446	452	HVEQMFR					Mascot
961.5101	961.5805	0.0704	73	192	199	NDSLFIPR					Mascot
976.5461	976.5751	0.029	30	271	279	AFAIADQLK					Mascot
1027.5419	1027.6177	0.0758	74	402	410	DLDLPEGLR					Mascot
1071.5834	1071.6605	0.0771	72	371	379	ISAVYSIYR					Mascot
1132.6473	1132.7312	0.0839	74	271	280	AFAIADQLKR					Mascot
1154.5841	1154.6694	0.0853	74	107	115	AEYQDLFLR	49	99.993			Mascot
1154.5841	1154.6694	0.0853	74	107	115	AEYQDLFLR					Mascot
1156.6685	1156.7285	0.06	52	138	148	IALQGATDVLR					Mascot
1374.6431	1374.766	0.1229	89	417	428	DGEMIRPNGDVR			Oxidation (M)[4]		Mascot
1404.7845	1404.8896	0.1051	75	433	445	DRVVIFATADAVK					Mascot
1444.8522	1444.9563	0.1041	72	236	250	IVIAGGGNIGLYVAK					Mascot
1568.818	1568.9353	0.1173	75	222	235	TLGLFGHEKPEANR					Mascot
1648.8475	1648.973	0.1255	76	3	18	VIVCGAGQVGYGIAER	58	100	Carbamidomethyl (C)[4]		Mascot
1648.8475	1648.973	0.1255	76	3	18	VIVCGAGQVGYGIAER			Carbamidomethyl (C)[4]		Mascot
1689.829	1689.9568	0.1278	76	19	34	LAAEENDVSVIDTSAR					Mascot
1810.8817	1811.019	0.1373	76	200	216	SSDELNAGDLAYVVTTR					Mascot
1958.0957	1958.2362	0.1405	72	174	191	QLTELFPDLTATVVGVVR	93	100			Mascot
1958.0957	1958.2362	0.1405	72	174	191	QLTELFPDLTATVVGVVR					Mascot

2069.1125	2069.2578	0.1453	70	382	401	AELIEAEALETSSLVGAPLR				Mascot
2296.2039	2296.2947	0.0908	40	154	173	IIGLAIECLDECPVINTPLR		Carbamidomethyl (C)[8,12]		Mascot
2333.2961	2333.4558	0.1597	68	116	136	ENLPIDVIISPEIEVGEVVLR				Mascot
2340.2405	2340.4038	0.1633	70	380	401	DRAELIEAEALETSSLVGAPLR				Mascot
2872.4583	2872.6357	0.1774	62	149	173	FADDKIIGLAIECLDECPVINTPLR		Carbamidomethyl (C)[13,17]		Mascot

2	hypothetical protein BSUIS_B0533 [Brucella suis ATCC 23445]	gi|163844696|ref|YP_001622351.1|	13	58	97.839	.T.	AlDahouk_6214_Z19769-28_T1	482

Peptide Information

Calc. Mass	Obsrv. Mass	± da	± ppm	Start Seq.	End Seq.	Sequence	Ion Score	C. I. %	Modification	Rank	Result Type
816.5189	816.527	0.0081	10	152	158	TLQITLK					Mascot
839.408	839.4673	0.0593	71	417	423	GMFDLTR					Mascot
953.4661	953.536	0.0699	73	82	88	VYTFHMR					Mascot
969.4611	969.5325	0.0714	74	82	88	VYTFHMR			Oxidation (M)[6]		Mascot
1241.7351	1241.8228	0.0877	71	475	485	ILAEAEEILLK					Mascot
1334.6951	1334.793	0.0979	73	52	63	DLYEGLLAQNAK					Mascot
1358.7889	1358.7961	0.0072	5	147	158	ALDDKTLQITLK					Mascot
1485.7908	1485.8984	0.1076	72	361	374	EAGVEPNTLSVTLR					Mascot
1522.6995	1522.8204	0.1209	79	227	238	IDTVNWMPFEDR					Mascot
1527.8457	1527.955	0.1093	72	274	286	LAPYLGIYYVDIK					Mascot
1538.6945	1538.8173	0.1228	80	227	238	IDTVNWMPFEDR			Oxidation (M)[7]		Mascot
1692.8075	1692.9459	0.1384	82	341	354	LDFANEDILDREDK					Mascot
1931.8558	1932.0099	0.1541	80	93	109	WSNGDPVTAGDFEFSFR					Mascot
2056.042	2056.1816	0.1396	68	486	504	EGAIVPLMYYSSTALVADR					Mascot
2295.0232	2295.252	0.2288	100	28	48	GNDTDPATLDQHHTSTVSENR					Mascot

**Gel Idx/Pos**	206/I6	**Instr./Gel Origin**	AK043/Div_120507	**Process Status**	Analysis Succeeded
**Plate [#] Name**	[1] 1300017700	**Instrument Sample Name**		**Spectra**	4

Rank	Protein Name	Accession No.	Pep. Count	Protein Score	Protein Score C. I. %	Total Ion Score	Best Ion Score	Best Ion C. I. %	Total Ion C. I. %	Confirmed	Sample Name	Customer sample Name
1	unnamed protein product [Brucella suis 1330]	gi|23502737|ref|NP_698864.1|	22	324	100	159	159	100	100	.T.	AlDahouk_6214_Z19769-29_T1	495

Peptide Information

Calc. Mass	Obsrv. Mass	± da	± ppm	Start Seq.	End Seq.	Sequence	Ion Score	C. I. %	Modification	Rank	Result Type
716.4049	716.4335	0.0286	40	2	8	AKSGTPR					Mascot
818.4618	818.4962	0.0344	42	257	263	LTSLQEK					Mascot
887.4581	887.5297	0.0716	81	422	429	DADALVQR					Mascot
956.5159	956.5918	0.0759	79	28	36	RVDEPAAAK					Mascot
1043.5592	1043.6433	0.0841	81	421	429	RDADALVQR					Mascot
1288.6532	1288.7635	0.1103	86	37	50	ATPAAEPVGGFSGK					Mascot
1289.6155	1289.7405	0.125	97	383	394	MEAALQAGDLDR					Mascot
1305.6104	1305.7245	0.1141	87	383	394	MEAALQAGDLDR			Oxidation (M)[1]		Mascot
1394.8002	1394.8885	0.0883	63	270	283	QPDAAVLIAANALK					Mascot
1425.7584	1425.8722	0.1138	80	15	27	NPVTINLDPSEVK					Mascot
1427.7166	1427.8141	0.0975	68	395	407	AIGEWEQLPADAK					Mascot
1556.8068	1556.938	0.1312	84	70	84	APFTAGVKPEAESPR					Mascot
1581.8595	1581.9869	0.1274	81	15	28	NPVTINLDPSEVKR					Mascot
1596.7612	1596.8955	0.1343	84	85	101	ETANAAPSAATPPADGR					Mascot
1726.9122	1727.0558	0.1436	83	344	359	LPADASLWDQLLASAR					Mascot
1826.9032	1827.0552	0.152	83	156	173	SNPAPAPLDDAGKAEFA R					Mascot
1871.9762	1872.1323	0.1561	83	319	336	GVPTIADLNAWFGAVAN R					Mascot
1880.9647	1881.1266	0.1619	86	139	155	VTAGQLAHMEQQIADLR					Mascot
1896.9596	1897.1272	0.1676	88	139	155	VTAGQLAHMEQQIADLR			Oxidation (M)[9]		Mascot
2120.0505	2120.2314	0.1809	85	294	313	AELDTYVSVAPQDASVE GLR	159	100			Mascot
2120.0505	2120.2314	0.1809	85	294	313	AELDTYVSVAPQDASVE GLR					Mascot
2152.1831	2152.3667	0.1836	85	360	382	GLVSVRPVAGNVSGTGV GPTTAR					Mascot
2431.1946	2431.4004	0.2058	85	198	221	QIEALPTTSENGTAAGDI ASLSER					Mascot
2482.3301	2482.5237	0.1936	78	337	359	IVATENKLPADASLWDQL LASAR					Mascot
3305.676	3305.9402	0.2642	80	51	84	AEAKPAAQAAPASSTGP DKAPFTAGVKPEAESPR					Mascot

2	gatB gene product [Brucella suis 1330]	gi|23501785|ref|NP_697912.1|	17	212	100	122	79	100	100	.T.	AlDahouk_6214_Z19769-29_T1	495

Peptide Information

Calc. Mass	Obsrv. Mass	± da	± ppm	Start Seq.	End Seq.	Sequence	Ion Score	C. I. %	Modification	Rank	Result Type

704.3937	704.4521	0.0584	83	2	7	SIIDTR					Mascot
727.4097	727.4514	0.0417	57	8	13	TPEPKR					Mascot
751.4097	751.4646	0.0549	73	93	98	SVFDRK					Mascot
773.4515	773.5098	0.0583	75	427	432	KLVEER					Mascot
859.4631	859.5344	0.0713	83	218	225	ADVNVSVR					Mascot
976.4958	976.574	0.0782	80	226	234	RPGGEFGTR	44	99.967			Mascot
976.4958	976.574	0.0782	80	226	234	RPGGEFGTR					Mascot
1134.5175	1134.6115	0.094	83	286	294	SKEEAHDYR					Mascot
1161.6262	1161.7179	0.0917	79	273	282	LFDPVKGETR					Mascot
1241.7212	1241.7861	0.0649	52	81	92	TGIGLNAQINLK					Mascot
1282.6426	1282.7485	0.1059	83	245	255	FVGQAIEYEAR	79	100			Mascot
1282.6426	1282.7485	0.1059	83	245	255	FVGQAIEYEAR					Mascot
1391.6802	1391.7971	0.1169	84	134	145	GQFEDVEIGIER					Mascot
1427.6802	1427.8141	0.1339	94	348	360	AIADYYEAVAEGR					Mascot
1596.8704	1596.8955	0.0251	16	478	493	ATGGKANPQAVNELVK					Mascot
1614.8883	1615.0223	0.134	83	171	185	SGVALMEIVSKPDLR					Mascot
1618.7748	1618.921	0.1462	90	413	426	DLFEIVWNEGGDPK					Mascot
1630.8833	1631.0167	0.1334	82	171	185	SGVALMEIVSKPDLR			Oxidation (M)[6]		Mascot
1648.8879	1649.0079	0.12	73	463	477	AKPTLAGWFVGQVMK			Oxidation (M)[14]		Mascot
1756.9076	1757.0549	0.1473	84	257	272	QIAILEDGGVIDQETR					Mascot

3 aspartyl/glutamyl-tRNA amidotransferase subunit B [Brucella suis ATCC 23445] gi|163843171|ref|YP_001627575.1| 17 212 100 122 79 100 100 .T. AlDahouk_6214_Z19769-29_T1 495

Peptide Information

Calc. Mass	Obsrv. Mass	± da	± ppm	Start Seq.	End Seq.	Sequence	Ion Score	C. I. %	Modification	Rank	Result Type
704.3937	704.4521	0.0584	83	2	7	SIIDTR					Mascot
727.4097	727.4514	0.0417	57	8	13	TPEPKR					Mascot
751.4097	751.4646	0.0549	73	93	98	SVFDRK					Mascot
773.4515	773.5098	0.0583	75	458	464	VEQAKAK					Mascot
859.4631	859.5344	0.0713	83	218	225	ADVNVSVR					Mascot
976.4958	976.574	0.0782	80	226	234	RPGGEFGTR	44	99.967			Mascot
976.4958	976.574	0.0782	80	226	234	RPGGEFGTR					Mascot
1134.5175	1134.6115	0.094	83	286	294	SKEEAHDYR					Mascot

1161.6262	1161.7179	0.0917	79	273	282	LFDPVKGETR				Mascot
1241.7212	1241.7861	0.0649	52	81	92	TGIGLNAQINLK				Mascot
1282.6426	1282.7485	0.1059	83	245	255	FVGQAIEYEAR	79	100		Mascot
1282.6426	1282.7485	0.1059	83	245	255	FVGQAIEYEAR				Mascot
1391.6802	1391.7971	0.1169	84	134	145	GQFEDVEIGIER				Mascot
1427.6802	1427.8141	0.1339	94	348	360	AIADYYEAVAEGR				Mascot
1596.8704	1596.8955	0.0251	16	480	495	ATGGKANPQAVNELVK				Mascot
1614.8883	1615.0223	0.134	83	171	185	SGVALMEIVSKPDLR				Mascot
1618.7748	1618.921	0.1462	90	413	426	DLFEIVWNEGGDPK				Mascot
1630.8833	1631.0167	0.1334	82	171	185	SGVALMEIVSKPDLR			Oxidation (M)[6]	Mascot
1648.8879	1649.0079	0.12	73	465	479	AKPTLAGWFVGQVMK			Oxidation (M)[14]	Mascot
1756.9076	1757.0549	0.1473	84	257	272	QIAILEDGGVIDQETR				Mascot

Gel Idx/Pos<br>Plate [#] Name	207/I7<br>[1] 1300017700	Instr./Gel Origin<br>Instrument Sample Name	AK043/Div_120507	Process Status<br>Spectra	Analysis Succeeded<br>4

Rank	Protein	Name	Accession No.	Pep. Count	Protein Score	Protein Score C. I. %	Total Ion Score	Best Ion Score	Best Ion C. I. %	Total Ion C. I. %	Confirmed	Sample Name	Customer sample Name
1	unnamed protein product [Brucella suis 1330]		gi|23502737|ref|NP_698864.1|	22	472	100	306	144	100	100	.T.	AlDahouk_6214_Z19769-30_T1	2070

Peptide Information

Calc. Mass	Obsrv. Mass	± da	± ppm	Start Seq.	End Seq.	Sequence	Ion Score	C. I. %	Modification	Rank	Result Type
710.3137	710.3809	0.0672	95	264	269	MSETAR			Oxidation (M)[1]		Mascot
887.4581	887.5286	0.0705	79	422	429	DADALVQR					Mascot
956.5159	956.5834	0.0675	71	28	36	RVDEPAAAK					Mascot
1043.5592	1043.6431	0.0839	80	421	429	RDADALVQR					Mascot
1288.6532	1288.7667	0.1135	88	37	50	ATPAAEPVGGFSGK					Mascot
1305.6104	1305.7224	0.112	86	383	394	MEAALQAGDLDR			Oxidation (M)[1]		Mascot
1316.7056	1316.8154	0.1098	83	185	197	AEAVAGTLSELQK					Mascot
1394.8002	1394.9078	0.1076	77	270	283	QPDAAVLIAANALK					Mascot
1425.7584	1425.8749	0.1165	82	15	27	NPVTINLDPSEVK					Mascot
1427.7166	1427.8171	0.1005	70	395	407	AIGEWEQLPADAK	53	99.997			Mascot
1427.7166	1427.8171	0.1005	70	395	407	AIGEWEQLPADAK					Mascot
1556.8068	1556.9335	0.1267	81	70	84	APFTAGVKPEAESPR					Mascot

Calc. Mass	Obsrv. Mass	± da	± ppm	Start Seq.	End Seq.	Sequence	Ion Score	C. I. %	Modification	Rank	Result Type
1581.8595	1581.9854	0.1259	80	15	28	NPVTINLDPSEVKR					Mascot
1596.7612	1596.8928	0.1316	82	85	101	ETANAAPSAATPPADGR					Mascot
1726.9122	1727.0406	0.1284	74	344	359	LPADASLWDQLLASAR	109	100			Mascot
1726.9122	1727.0406	0.1284	74	344	359	LPADASLWDQLLASAR					Mascot
1826.9032	1827.0525	0.1493	82	156	173	SNPAPAPLDDAGKAEFA R					Mascot
1871.9762	1872.1254	0.1492	80	319	336	GVPTIADLNAWFGAVAN R					Mascot
1880.9647	1881.1166	0.1519	81	139	155	VTAGQLAHMEQQIADLR					Mascot
1896.9596	1897.1176	0.158	83	139	155	VTAGQLAHMEQQIADLR			Oxidation (M)[9]		Mascot
2120.0505	2120.2249	0.1744	82	294	313	AELDTYVSVAPQDASVE GLR	144	100			Mascot
2120.0505	2120.2249	0.1744	82	294	313	AELDTYVSVAPQDASVE GLR					Mascot
2152.1831	2152.3604	0.1773	82	360	382	GLVSVRPVAGNVSGTGV GPTTAR					Mascot
2431.1946	2431.3967	0.2021	83	198	221	QIEALPTTSENGTAAGDI ASLSER					Mascot
2482.3301	2482.5144	0.1843	74	337	359	IVATENKLPADASLWDQL LASAR					Mascot
3305.676	3305.9209	0.2449	74	51	84	AEAKPAAQAAPASSTGP DKAPFTAGVKPEAESPR					Mascot

2 gatB gene product [Brucella suis 1330] gi|23501785|ref|NP_697912.1| 19 168 100 58 58 100 100 .T. AlDahouk_6214_Z19769-30_T1 2070

Peptide Information

Calc. Mass	Obsrv. Mass	± da	± ppm	Start Seq.	End Seq.	Sequence	Ion Score	C. I. %	Modification	Rank	Result Type
704.3937	704.4395	0.0458	65	2	7	SIIDTR					Mascot
751.4097	751.462	0.0523	70	93	98	SVFDRK					Mascot
976.4958	976.5728	0.077	79	226	234	RPGGEFGTR					Mascot
1134.5175	1134.6106	0.0931	82	286	294	SKEEAHDYR					Mascot
1161.6262	1161.719	0.0928	80	273	282	LFDPVKGETR					Mascot
1182.6477	1182.6567	0.009	8	483	493	ANPQAVNELVK					Mascot
1241.7212	1241.8184	0.0972	78	81	92	TGIGLNAQINLK					Mascot
1282.6426	1282.7473	0.1047	82	245	255	FVGQAIEYEAR					Mascot
1391.6802	1391.7949	0.1147	82	134	145	GQFEDVEIGIER					Mascot
1427.6802	1427.8171	0.1369	96	348	360	AIADYYEAVAEGR	58	100			Mascot
1427.6802	1427.8171	0.1369	96	348	360	AIADYYEAVAEGR					Mascot
1596.8704	1596.8928	0.0224	14	478	493	ATGGKANPQAVNELVK					Mascot
1614.8883	1615.019	0.1307	81	171	185	SGVALMEIVSKPDLR					Mascot
1618.7748	1618.9193	0.1445	89	413	426	DLFEIVWNEGGDPK					Mascot

Calc. Mass	Obsrv. Mass	± da	± ppm	Start Seq.	End Seq.	Sequence	Ion Score	C. I. %	Modification	Rank	Result Type
1630.8833	1631.0146	0.1313	81	171	185	SGVALMEIVSKPDLR			Oxidation (M)[6]		Mascot
1682.9224	1683.0483	0.1259	75	364	379	AAANWVINDLLGALNK					Mascot
1756.9076	1757.0546	0.147	84	257	272	QIAILEDGGVIDQETR					Mascot
1782.9232	1783.0702	0.147	82	446	462	AVDDVIAANPDKVEQAK					Mascot
1930.9908	1931.0144	0.0236	12	410	426	IAKDLFEIVWNEGGDPK					Mascot
1946.8484	1947.0137	0.1653	85	155	170	SMHDQHPTMSYVDLNR			Oxidation (M)[2]		Mascot
2108.0125	2108.1995	0.187	89	99	115	NYFYPDLPQGYQISQFK					Mascot

3	aspartyl/glutamyl-tRNA amidotransferase subunit B [Brucella suis ATCC 23445]	gi|163843171|ref|YP_001627575.1|	19	167	100	58	58	100	100	.T.	AlDahouk_6214_Z19769-30_T1 2070

Peptide Information

Calc. Mass	Obsrv. Mass	± da	± ppm	Start Seq.	End Seq.	Sequence	Ion Score	C. I. %	Modification	Rank	Result Type
704.3937	704.4395	0.0458	65	2	7	SIIDTR					Mascot
751.4097	751.462	0.0523	70	93	98	SVFDRK					Mascot
976.4958	976.5728	0.077	79	226	234	RPGGEFGTR					Mascot
1134.5175	1134.6106	0.0931	82	286	294	SKEEAHDYR					Mascot
1161.6262	1161.719	0.0928	80	273	282	LFDPVKGETR					Mascot
1182.6477	1182.6567	0.009	8	485	495	ANPQAVNELVK					Mascot
1241.7212	1241.8184	0.0972	78	81	92	TGIGLNAQINLK					Mascot
1282.6426	1282.7473	0.1047	82	245	255	FVGQAIEYEAR					Mascot
1391.6802	1391.7949	0.1147	82	134	145	GQFEDVEIGIER					Mascot
1427.6802	1427.8171	0.1369	96	348	360	AIADYYEAVAEGR	58	100			Mascot
1427.6802	1427.8171	0.1369	96	348	360	AIADYYEAVAEGR					Mascot
1596.8704	1596.8928	0.0224	14	480	495	ATGGKANPQAVNELVK					Mascot
1614.8883	1615.019	0.1307	81	171	185	SGVALMEIVSKPDLR					Mascot
1618.7748	1618.9193	0.1445	89	413	426	DLFEIVWNEGGDPK					Mascot
1630.8833	1631.0146	0.1313	81	171	185	SGVALMEIVSKPDLR			Oxidation (M)[6]		Mascot
1682.9224	1683.0483	0.1259	75	364	379	AAANWVINDLLGALNK					Mascot
1756.9076	1757.0546	0.147	84	257	272	QIAILEDGGVIDQETR					Mascot
1782.9232	1783.0702	0.147	82	446	462	AVDDVIAANPDKVEQAK					Mascot
1930.9908	1931.0144	0.0236	12	410	426	IAKDLFEIVWNEGGDPK					Mascot
1946.8484	1947.0137	0.1653	85	155	170	SMHDQHPTMSYVDLNR			Oxidation (M)[2]		Mascot
2108.0125	2108.1995	0.187	89	99	115	NYFYPDLPQGYQISQFK					Mascot

4	hisS gene product [Brucella suis 1330]	gi|23499949|ref|N	20	134	100	22	22	96.579	96.579	.T.	AlDahouk_6 2070

		P_699389.1|										214_Z19769-30_T1	

Peptide Information

Calc. Mass	Obsrv. Mass	± da	± ppm	Start Seq.	End Seq.	Sequence	Ion Score	C. I. %	Modification	Rank	Result Type
761.394	761.4504	0.0564	74	207	213	FGPEGVR					Mascot
770.4995	770.5563	0.0568	74	194	199	RLNVLR					Mascot
778.3994	778.458	0.0586	75	112	117	NGWVFR					Mascot
809.4152	809.4731	0.0579	72	337	343	YDGLVSR					Mascot
816.4573	816.5248	0.0675	83	450	456	DLVEGKR					Mascot
854.4478	854.5159	0.0681	80	118	125	NEKPGPGR					Mascot
915.4894	915.5618	0.0724	79	488	495	EILDAQAR					Mascot
973.4948	973.5813	0.0865	89	479	487	EDGLVDAVR					Mascot
991.5319	991.6085	0.0766	77	326	336	VVFGSVGGGGR					Mascot
1012.6149	1012.6746	0.0597	59	292	300	VKIDPSVVR					Mascot
1019.552	1019.6335	0.0815	80	86	94	YDLTAPLAR					Mascot
1156.6433	1156.7346	0.0913	79	469	478	ESRPAQITVR					Mascot
1224.5854	1224.6876	0.1022	83	219	229	GRLDESGDFTK					Mascot
1288.6644	1288.7667	0.1023	79	15	25	GFVDRVPDDLR					Mascot
1414.7107	1414.8284	0.1177	83	429	441	AAPCVVIQGSQER			Carbamidomethyl (C)[4]		Mascot
1432.7067	1432.8242	0.1175	82	457	468	LSAEIEDNVTWR					Mascot
1473.7695	1473.8873	0.1178	80	346	360	GEPVPATGFSIGVSR					Mascot
1726.8799	1727.0406	0.1607	93	95	108	YVAENFETLPKPYR	22	96.579			Mascot
1726.8799	1727.0406	0.1607	93	95	108	YVAENFETLPKPYR					Mascot
1776.9391	1777.084	0.1449	82	344	360	FRGEPVPATGFSIGVSR					Mascot
1840.9651	1841.1176	0.1525	83	175	193	VLDGVLDAIGLEGEGNAAK					Mascot

5	histidyl-tRNA synthetase [Brucella suis ATCC 23445]	gi|163844376|ref|YP_001622031.1|	20	130	100	22	22	96.579	96.579	.T.	AlDahouk_6214_Z19769-30_T1	2070

Peptide Information

Calc. Mass	Obsrv. Mass	± da	± ppm	Start Seq.	End Seq.	Sequence	Ion Score	C. I. %	Modification	Rank	Result Type
761.394	761.4504	0.0564	74	207	213	FGPEGVR					Mascot
770.4995	770.5563	0.0568	74	194	199	RLNVLR					Mascot
778.3994	778.458	0.0586	75	112	117	NGWVFR					Mascot

Calc. Mass	Obsrv. Mass	± da	± ppm	Start Seq.	End Seq.	Sequence	Ion Score	C. I. %	Modification	Rank	Result Type
809.4152	809.4731	0.0579	72	337	343	YDGLVSR					Mascot
816.4573	816.5248	0.0675	83	450	456	DLVEGKR					Mascot
854.4478	854.5159	0.0681	80	118	125	NEKPGPGR					Mascot
915.4894	915.5618	0.0724	79	488	495	EILDAQAR					Mascot
973.4948	973.5813	0.0865	89	479	487	EDGLVDAVR					Mascot
991.5319	991.6085	0.0766	77	326	336	VVFGSVGGGGR					Mascot
1012.6149	1012.6746	0.0597	59	292	300	VKIDPSVVR					Mascot
1019.552	1019.6335	0.0815	80	86	94	YDLTAPLAR					Mascot
1156.6433	1156.7346	0.0913	79	469	478	ESRPAQITVR					Mascot
1224.5854	1224.6876	0.1022	83	219	229	GRLDESGDFTK					Mascot
1288.6644	1288.7667	0.1023	79	15	25	GFVDRVPDDLR					Mascot
1414.7107	1414.8284	0.1177	83	429	441	AAPCVVIQGSQER			Carbamidomethyl (C)[4]		Mascot
1432.7067	1432.8242	0.1175	82	457	468	LSAEIEDNVTWR					Mascot
1473.7695	1473.8873	0.1178	80	346	360	GEPVPATGFSIGVSR					Mascot
1726.8799	1727.0406	0.1607	93	95	108	YVAENFETLPKPYR	22	96.579			Mascot
1726.8799	1727.0406	0.1607	93	95	108	YVAENFETLPKPYR					Mascot
1776.9391	1777.084	0.1449	82	344	360	FRGEPVPATGFSIGVSR					Mascot
1840.9651	1841.1176	0.1525	83	175	193	VLDGVLDAIGLEGEGNAAK					Mascot

6 hypothetical protein BSUIS_B0533 [Brucella suis ATCC 23445] gi|163844696|ref|YP_001622351.1| 15 76 99.968 .T. AlDahouk_6214_Z19769-30_T1 2070

Peptide Information

Calc. Mass	Obsrv. Mass	± da	± ppm	Start Seq.	End Seq.	Sequence	Ion Score	C. I. %	Modification	Rank	Result Type
816.5189	816.5248	0.0059	7	152	158	TLQITLK					Mascot
839.408	839.4739	0.0659	79	417	423	GMFDLTR					Mascot
953.4661	953.542	0.0759	80	82	88	VYTFHMR					Mascot
969.4611	969.5376	0.0765	79	82	88	VYTFHMR			Oxidation (M)[6]		Mascot
1241.7351	1241.8184	0.0833	67	475	485	ILAEAEEILLK					Mascot
1334.6951	1334.8013	0.1062	80	52	63	DLYEGLLAQNAK					Mascot
1485.7908	1485.9124	0.1216	82	361	374	EAGVEPNTLSVTLR					Mascot
1522.6995	1522.8274	0.1279	84	227	238	IDTVNWMPFEDR					Mascot
1527.8457	1527.9706	0.1249	82	274	286	LAPYLGIYYVDIK					Mascot
1538.6945	1538.8313	0.1368	89	227	238	IDTVNWMPFEDR			Oxidation (M)[7]		Mascot
1692.8075	1692.9498	0.1423	84	341	354	LDFANEDILDREDK					Mascot

1703.7224	1703.8656	0.1432	84	505	519	VQGCDDNLMNSHGTR	Carbamidomethyl (C)[4]	Mascot
1789.8239	1789.9777	0.1538	86	64	81	GEAIPGAASSWDISEDGK		Mascot
1931.8558	1932.0126	0.1568	81	93	109	WSNGDPVTAGDFEFSFR		Mascot
1948.9175	1949.0562	0.1387	71	401	416	LDEVEGTTYFNYLQEK		Mascot
1979.9644	1980.1213	0.1569	79	301	317	AISLAADREFMADEVWR		Mascot
2056.042	2056.2041	0.1621	79	486	504	EGAIVPLMYYSSTALVAD R		Mascot

Gel Idx/Pos<br>Plate [#] Name	208/I8<br>[1] 1300017700	Instr./Gel Origin<br>Instrument Sample Name	AK043/Div_120507	Process Status<br>Spectra	Analysis Succeeded<br>4

Rank	Protein Name	Accession No.	Pep. Count	Protein Score	Protein Score C. I. %	Total Ion Score	Best Ion Score	Best Ion C. I. %	Total Ion C. I. %	Confirmed	Sample Name	Customer sample Name
1	unnamed protein product [Brucella suis 1330]	gi|23502737|ref|NP_698864.1|	13	243	100	172	94	100	100	.T.	AlDahouk_6214_Z19769-50_T1	488

Peptide Information

Calc. Mass	Obsrv. Mass	± da	± ppm	Start Seq.	End Seq.	Sequence	Ion Score	C. I. %	Modification	Rank	Result Type
818.4618	818.472	0.0102	12	257	263	LTSLQEK					Mascot
887.4581	887.5172	0.0591	67	422	429	DADALVQR					Mascot
962.5417	962.5595	0.0178	18	169	176	AEFARLQK					Mascot
1427.7166	1427.8077	0.0911	64	395	407	AIGEWEQLPADAK					Mascot
1556.8068	1556.9307	0.1239	80	70	84	APFTAGVKPEAESPR					Mascot
1581.8595	1581.9821	0.1226	78	15	28	NPVTINLDPSEVKR					Mascot
1596.7612	1596.8942	0.133	83	85	101	ETANAAPSAATPPADGR					Mascot
1726.9122	1727.0442	0.132	76	344	359	LPADASLWDQLLASAR	94	100			Mascot
1726.9122	1727.0442	0.132	76	344	359	LPADASLWDQLLASAR					Mascot
1871.9762	1872.1187	0.1425	76	319	336	GVPTIADLNAWFGAVAN R	79	100			Mascot
1871.9762	1872.1187	0.1425	76	319	336	GVPTIADLNAWFGAVAN R					Mascot
1880.9647	1881.1144	0.1497	80	139	155	VTAGQLAHMEQQIADLR					Mascot
1896.9596	1897.1095	0.1499	79	139	155	VTAGQLAHMEQQIADLR			Oxidation (M)[9]		Mascot
2120.0505	2120.2173	0.1668	79	294	313	AELDTYVSVAPQDASVE GLR					Mascot
2152.1831	2152.3542	0.1711	80	360	382	GLVSVRPVAGNVSGTGV GPTTAR					Mascot
2431.1946	2431.3945	0.1999	82	198	221	QIEALPTTSENGTAAGDI ASLSER					Mascot

Rank	Protein Name	Accession No.	Pep. Count	Protein Score	Protein Score C. I. %	Total Ion Score	Best Ion Score	Best Ion C. I. %	Total Ion C. I. %	Confirmed	Sample Name	Customer sample Name
2	gatB gene product [Brucella suis 1330]	gi|23501785|ref|N	17	184	100	96	96	100	100	.T.	AlDahouk_6	488

		P_697912.1											214_Z19769-50_T1	

Peptide Information

Calc. Mass	Obsrv. Mass	± da	± ppm	Start Seq.	End Seq.	Sequence	Ion Score	C. I. %	Modification	Rank	Result Type
704.3937	704.432	0.0383	54	2	7	SIIDTR					Mascot
751.4097	751.4567	0.047	63	93	98	SVFDRK					Mascot
961.5134	961.5839	0.0705	73	428	435	LVEERGMK					Mascot
976.4958	976.572	0.0762	78	226	234	RPGGEFGTR					Mascot
1134.5175	1134.6132	0.0957	84	286	294	SKEEAHDYR					Mascot
1161.6262	1161.7162	0.09	77	273	282	LFDPVKGETR					Mascot
1182.6477	1182.6622	0.0145	12	483	493	ANPQAVNELVK					Mascot
1241.7212	1241.8033	0.0821	66	81	92	TGIGLNAQINLK					Mascot
1282.6426	1282.744	0.1014	79	245	255	FVGQAIEYEAR	96	100			Mascot
1282.6426	1282.744	0.1014	79	245	255	FVGQAIEYEAR					Mascot
1391.6802	1391.7908	0.1106	79	134	145	GQFEDVEIGIER					Mascot
1427.6802	1427.8077	0.1275	89	348	360	AIADYYEAVAEGR					Mascot
1596.8704	1596.8942	0.0238	15	478	493	ATGGKANPQAVNELVK					Mascot
1614.8883	1615.0146	0.1263	78	171	185	SGVALMEIVSKPDLR					Mascot
1618.7748	1618.9178	0.143	88	413	426	DLFEIVWNEGGDPK					Mascot
1648.8879	1648.9833	0.0954	58	463	477	AKPTLAGWFVGQVMK			Oxidation (M)[14]		Mascot
1682.9224	1683.0333	0.1109	66	364	379	AAANWVINDLLGALNK					Mascot
1756.9076	1757.0482	0.1406	80	257	272	QIAILEDGGVIDQETR					Mascot

3	aspartyl/glutamyl-tRNA amidotransferase subunit B [Brucella suis ATCC 23445]	gi|163843171|ref|YP_001627575.1|	17	183	100	96	96	100	100	.T.	AlDahouk_6214_Z19769-50_T1	488		

Peptide Information

Calc. Mass	Obsrv. Mass	± da	± ppm	Start Seq.	End Seq.	Sequence	Ion Score	C. I. %	Modification	Rank	Result Type
704.3937	704.432	0.0383	54	2	7	SIIDTR					Mascot
751.4097	751.4567	0.047	63	93	98	SVFDRK					Mascot
961.5134	961.5839	0.0705	73	428	435	LVEERGMK					Mascot
976.4958	976.572	0.0762	78	226	234	RPGGEFGTR					Mascot
1134.5175	1134.6132	0.0957	84	286	294	SKEEAHDYR					Mascot
1161.6262	1161.7162	0.09	77	273	282	LFDPVKGETR					Mascot

Calc. Mass	Obsrv. Mass	± da	± ppm	Start Seq.	End Seq.	Sequence	Ion Score	C. I. %	Modification	Rank	Result Type
1182.6477	1182.6622	0.0145	12	485	495	ANPQAVNELVK					Mascot
1241.7212	1241.8033	0.0821	66	81	92	TGIGLNAQINLK					Mascot
1282.6426	1282.744	0.1014	79	245	255	FVGQAIEYEAR	96	100			Mascot
1282.6426	1282.744	0.1014	79	245	255	FVGQAIEYEAR					Mascot
1391.6802	1391.7908	0.1106	79	134	145	GQFEDVEIGIER					Mascot
1427.6802	1427.8077	0.1275	89	348	360	AIADYYEAVAEGR					Mascot
1596.8704	1596.8942	0.0238	15	480	495	ATGGKANPQAVNELVK					Mascot
1614.8883	1615.0146	0.1263	78	171	185	SGVALMEIVSKPDLR					Mascot
1618.7748	1618.9178	0.143	88	413	426	DLFEIVWNEGGDPK					Mascot
1648.8879	1648.9833	0.0954	58	465	479	AKPTLAGWFVGQVMK			Oxidation (M)[14]		Mascot
1682.9224	1683.0333	0.1109	66	364	379	AAANWVINDLLGALNK					Mascot
1756.9076	1757.0482	0.1406	80	257	272	QIAILEDGGVIDQETR					Mascot

4	trkA gene product [Brucella suis 1330]	gi|23501992|ref|NP_698119.1|	22	152	100	.T.	AlDahouk_6214_Z19769-50_T1	488

Peptide Information

Calc. Mass	Obsrv. Mass	± da	± ppm	Start Seq.	End Seq.	Sequence	Ion Score	C. I. %	Modification	Rank	Result Type
709.3879	709.3984	0.0105	15	101	106	AQSYLK					Mascot
728.3474	728.3906	0.0432	59	256	260	EWHTR					Mascot
741.3817	741.4401	0.0584	79	453	458	VSLEFF					Mascot
744.425	744.4446	0.0196	26	35	40	DIEIVR					Mascot
765.473	765.5298	0.0568	74	362	367	ILQHVR					Mascot
946.4563	946.5319	0.0756	80	446	452	HVEQMFR					Mascot
961.5101	961.5839	0.0738	77	192	199	NDSLFIPR					Mascot
976.5461	976.572	0.0259	27	271	279	AFAIADQLK					Mascot
1027.5419	1027.6221	0.0802	78	402	410	DLDLPEGLR					Mascot
1071.5834	1071.6656	0.0822	77	371	379	ISAVYSIYR					Mascot
1132.6473	1132.7336	0.0863	76	271	280	AFAIADQLKR					Mascot
1154.5841	1154.6744	0.0903	78	107	115	AEYQDLFLR					Mascot
1156.6685	1156.7251	0.0566	49	138	148	IALQGATDVLR					Mascot
1568.818	1568.9434	0.1254	80	222	235	TLGLFGHEKPEANR					Mascot
1648.8475	1648.9833	0.1358	82	3	18	VIVCGAGQVGYGIAER			Carbamidomethyl (C)[4]		Mascot
1689.829	1689.9647	0.1357	80	19	34	LAAEENDVSVIDTSAR					Mascot
1810.8817	1811.027	0.1453	80	200	216	SSDELNAGDLAYVVTTR					Mascot

1958.0957	1958.2423	0.1466	75	174	191	QLTELFPDLTATVVGVVR				Mascot
2069.1125	2069.2659	0.1534	74	382	401	AELIEAEALETSSLVGAPLR				Mascot
2296.2039	2296.3208	0.1169	51	154	173	IIGLAIECLDECPVINTPLR			Carbamidomethyl (C)[8,12]	Mascot
2333.2961	2333.4719	0.1758	75	116	136	ENLPIDVIISPEIEVGEVVLR				Mascot
2340.2405	2340.406	0.1655	71	380	401	DRAELIEAEALETSSLVGAPLR				Mascot

Gel Idx/Pos	209/I9	Instr./Gel Origin	AK043/Div_120507	Process Status	Analysis Succeeded
Plate [#] Name	[1] 1300017700	Instrument Sample Name		Spectra	4

Rank	Protein	Name	Accession No.	Pep. Count	Protein Score	Protein Score C. I. %	Total Ion Score	Best Ion Score	Best Ion C. I. %	Total Ion C. I. %	Confirmed	Sample Name	Customer sample Name
1	metG gene product [Brucella suis 1330]		gi|23501876|ref|NP_698003.1|	30	435	100	193	76	100	100	.T.	AlDahouk_6214_Z19769-52_T1	554

Peptide Information

Calc. Mass	Obsrv. Mass	± da	± ppm	Start Seq.	End Seq.	Sequence	Ion Score	C. I. %	Modification	Rank	Result Type
705.3712	705.415	0.0438	62	57	62	MLQSAR					Mascot
721.3661	721.4046	0.0385	53	57	62	MLQSAR			Oxidation (M)[1]		Mascot
729.3752	729.4144	0.0392	54	325	329	YFLMR					Mascot
745.3701	745.4161	0.046	62	325	329	YFLMR			Oxidation (M)[4]		Mascot
800.4625	800.5033	0.0408	51	63	69	KEGITPR					Mascot
851.4482	851.4378	-0.0104	-12	75	81	NTSAFRR					Mascot
864.4573	864.5222	0.0649	75	318	324	YGLDQLR					Mascot
935.5196	935.5872	0.0676	72	193	200	NEIVSFVK					Mascot
1091.6207	1091.7024	0.0817	75	192	200	RNEIVSFVK					Mascot
1115.5269	1115.6122	0.0853	76	121	130	GGYAGWYSVR	60	100			Mascot
1115.5269	1115.6122	0.0853	76	121	130	GGYAGWYSVR					Mascot
1207.6371	1207.7285	0.0914	76	288	297	VFAHGFLFNR	76	100			Mascot
1207.6371	1207.7285	0.0914	76	288	297	VFAHGFLFNR					Mascot
1323.7994	1323.9022	0.1028	78	471	482	LLDILAVPADKR					Mascot
1337.6637	1337.7671	0.1034	77	425	435	YFAGQEPWALR	58	99.999			Mascot
1337.6637	1337.7671	0.1034	77	425	435	YFAGQEPWALR					Mascot
1353.6501	1353.7684	0.1183	87	363	374	SLSMIAKNCEGK			Carbamidomethyl (C)[9], Oxidation (M)[4]		Mascot
1451.7926	1451.9026	0.11	76	442	454	MGTVLYVTAEVLR					Mascot
1457.7594	1457.8732	0.1138	78	387	400	AILDQADAALETAR					Mascot

Calc. Mass	Obsrv. Mass	± da	± ppm	Start Seq.	End Seq.	Sequence	Ion Score	C. I. %	Modification	Rank	Result Type
1465.7587	1465.8701	0.1114	76	425	436	YFAGQEPWALRK					Mascot
1467.7876	1467.8984	0.1108	75	442	454	MGTVLYVTAEVLR			Oxidation (M)[1]		Mascot
1526.7903	1526.9122	0.1219	80	251	263	WAYWPANAHIIGK					Mascot
1561.7533	1561.8783	0.125	80	211	224	TTFDWGIPVPGDEK					Mascot
1573.822	1573.9436	0.1216	77	304	317	SVGNVIDPFELVER					Mascot
1585.8544	1585.9186	0.0642	40	387	401	AILDQADAALETARK					Mascot
1589.6602	1589.7864	0.1262	79	131	143	DEAYYGEEETEVR					Mascot
1626.7428	1626.8712	0.1284	79	82	95	MAEVLNSSNDDYIR					Mascot
1642.7378	1642.8759	0.1381	84	82	95	MAEVLNSSNDDYIR			Oxidation (M)[1]		Mascot
1643.8826	1643.9341	0.0515	31	456	470	VGIMVQPFIPQSAEK					Mascot
1799.9836	1800.1199	0.1363	76	455	470	RVGIMVQPFIPQSAEK					Mascot
1815.9785	1816.109	0.1305	72	455	470	RVGIMVQPFIPQSAEK			Oxidation (M)[5]		Mascot
1865.9214	1866.0789	0.1575	84	104	120	ASQAIWQAMVANGDIYK					Mascot
1992.0582	1992.1471	0.0889	45	437	454	TDPARMGTVLYVTAEVLR					Mascot
2004.9409	2005.1034	0.1625	81	330	347	EVPFGQDGSYSHEAIVNR					Mascot
2320.0557	2320.2285	0.1728	74	149	167	YGPQGTPVEWVEEESYFFR					Mascot
2396.239	2396.4082	0.1692	71	402	424	AMDDQALHLALGAIFAVVAEANR					Mascot
2564.3508	2564.5413	0.1905	74	483	507	QFADVLASPLAGGTDLPAPQPVFPR					Mascot
2684.3525	2684.5466	0.1941	72	375	400	VPQPGAFSEADKAILDQADAALETAR					Mascot

2	methionyl-tRNA synthetase [Brucella suis ATCC 23445] gi|163843264|ref|YP_001627668.1|	29	420	100	193	76	100	100	.T.	AlDahouk_6214_Z19769-52_T1	554

Peptide Information

Calc. Mass	Obsrv. Mass	± da	± ppm	Start Seq.	End Seq.	Sequence	Ion Score	C. I. %	Modification	Rank	Result Type
705.3712	705.415	0.0438	62	57	62	MLQSAR					Mascot
721.3661	721.4046	0.0385	53	57	62	MLQSAR			Oxidation (M)[1]		Mascot
729.3752	729.4144	0.0392	54	325	329	YFLMR					Mascot
745.3701	745.4161	0.046	62	325	329	YFLMR			Oxidation (M)[4]		Mascot
800.4625	800.5033	0.0408	51	63	69	KEGITPR					Mascot
851.4482	851.4378	-0.0104	-12	75	81	NTSAFRR					Mascot
864.4573	864.5222	0.0649	75	318	324	YGLDQLR					Mascot
935.5196	935.5872	0.0676	72	193	200	NEIVSFVK					Mascot
1091.6207	1091.7024	0.0817	75	192	200	RNEIVSFVK					Mascot

1115.5269	1115.6122	0.0853	76	121	130	GGYAGWYSVR	60	100			Mascot
1115.5269	1115.6122	0.0853	76	121	130	GGYAGWYSVR					Mascot
1207.6371	1207.7285	0.0914	76	288	297	VFAHGFLFNR	76	100			Mascot
1207.6371	1207.7285	0.0914	76	288	297	VFAHGFLFNR					Mascot
1323.7994	1323.9022	0.1028	78	471	482	LLDILAVPADKR					Mascot
1337.6637	1337.7671	0.1034	77	425	435	YFAGQEPWALR	58	99.999			Mascot
1337.6637	1337.7671	0.1034	77	425	435	YFAGQEPWALR					Mascot
1353.6501	1353.7684	0.1183	87	363	374	SLSMIAKNCEGK			Carbamidomethyl (C)[9], Oxidation (M)[4]		Mascot
1451.7926	1451.9026	0.11	76	442	454	MGTVLYVTAEVLR					Mascot
1457.7594	1457.8732	0.1138	78	387	400	AILDQADAALETAR					Mascot
1465.7587	1465.8701	0.1114	76	425	436	YFAGQEPWALRK					Mascot
1467.7876	1467.8984	0.1108	75	442	454	MGTVLYVTAEVLR			Oxidation (M)[1]		Mascot
1526.7903	1526.9122	0.1219	80	251	263	WAYWPANAHIIGK					Mascot
1561.7533	1561.8783	0.125	80	211	224	TTFDWGIPVPGDEK					Mascot
1573.822	1573.9436	0.1216	77	304	317	SVGNVIDPFELVER					Mascot
1585.8544	1585.9186	0.0642	40	387	401	AILDQADAALETARK					Mascot
1589.6602	1589.7864	0.1262	79	131	143	DEAYYGEEETEVR					Mascot
1626.7428	1626.8712	0.1284	79	82	95	MAEVLNSSNDDYIR					Mascot
1642.7378	1642.8759	0.1381	84	82	95	MAEVLNSSNDDYIR			Oxidation (M)[1]		Mascot
1643.8826	1643.9341	0.0515	31	456	470	VGIMVQPFIPQSAEK					Mascot
1799.9836	1800.1199	0.1363	76	455	470	RVGIMVQPFIPQSAEK					Mascot
1815.9785	1816.109	0.1305	72	455	470	RVGIMVQPFIPQSAEK			Oxidation (M)[5]		Mascot
1865.9214	1866.0789	0.1575	84	104	120	ASQAIWQAMVANGDIYK					Mascot
1992.0582	1992.1471	0.0889	45	437	454	TDPARMGTVLYVTAEVLR					Mascot
2004.9409	2005.1034	0.1625	81	330	347	EVPFGQDGSYSHEAIVNR					Mascot
2320.0557	2320.2285	0.1728	74	149	167	YGPQGTPVEWVEEESYFFR					Mascot
2396.239	2396.4082	0.1692	71	402	424	AMDDQALHLALGAIFAVVAEANR					Mascot
2564.3508	2564.5413	0.1905	74	483	507	QFADVLASPLAGGTDLPAPQPVFPR					Mascot

3	DEAD/DEAH box helicase [Brucella suis ATCC 23445]	gi|163843319|ref|YP_001627723.1|	17	63	99.362	.T.	AlDahouk_6214_Z19769-52_T1	554

Peptide Information

Calc. Mass	Obsrv. Mass	± da	± ppm	Start Seq.	End Seq.	Sequence	Ion Score	C. I. %	Modification	Rank	Result Type

705.4154	705.415	-0.0004	-1	42	46	QRVFR						Mascot
715.4348	715.401	-0.0338	-47	149	155	IIGLGDK						Mascot
728.4526	728.4246	-0.028	-38	157	162	RPKTAR						Mascot
731.4423	731.4117	-0.0306	-42	520	524	RPFRR						Mascot
732.4138	732.4071	-0.0067	-9	78	83	EIELTK						Mascot
743.4522	743.4324	-0.0198	-27	443	448	LRAVER						Mascot
748.4022	748.4331	0.0309	41	221	226	LTDLMR						Mascot
774.3965	774.4592	0.0627	81	527	533	RSNGGQR						Mascot
810.4216	810.4861	0.0645	80	471	478	GPAPQGQR						Mascot
816.4587	816.5278	0.0691	85	487	493	APAHKHR						Mascot
830.4842	830.4276	-0.0566	-68	445	451	AVERVTR						Mascot
868.5363	868.5928	0.0565	65	286	292	LLRDPVR						Mascot
1277.6821	1277.7983	0.1162	91	494	504	RPAQKAGEQHR						Mascot
1529.8243	1529.9052	0.0809	53	71	83	ECAVPLKEIELTK				Carbamidomethyl (C)[2]		Mascot
1643.8248	1643.9341	0.1093	66	355	369	YDVAAIHGNKSQNAR						Mascot
1660.9268	1660.9529	0.0261	16	84	100	ENTGGFAALGITGVLLK						Mascot
2009.1753	2009.1393	-0.036	-18	186	205	SAHISTALVLGGVSKLSQIK						Mascot

4	unnamed protein product [Brucella suis 1330]	gi|23501498|ref|NP_697625.1|	13	60	98.698	.T.	AlDahouk_6214_Z19769-52_T1	554

Peptide Information

Calc. Mass	Obsrv. Mass	± da	± ppm	Start Seq.	End Seq.	Sequence	Ion Score	C. I. %	Modification	Rank	Result Type
703.3984	703.3958	-0.0026	-4	481	487	AITAAEK					Mascot
741.4253	741.4478	0.0225	30	174	180	LIGADPR					Mascot
759.4611	759.448	-0.0131	-17	181	187	TDLAVLK					Mascot
816.4726	816.5278	0.0552	68	384	390	AALTVWR					Mascot
851.3716	851.4378	0.0662	78	104	110	DFGMEPR					Mascot
982.6155	982.6861	0.0706	72	60	68	VRPAVVSVR					Mascot
1274.6051	1274.7169	0.1118	88	195	205	FVYVAFGDDNK					Mascot
1471.7864	1471.8992	0.1128	77	492	504	AVLLQLQSNDQSR					Mascot
1529.7747	1529.9052	0.1305	85	195	207	FVYVAFGDDNKVR					Mascot
1599.8813	1600.0006	0.1193	75	491	504	KAVLLQLQSNDQSR					Mascot
1741.8715	1742.0129	0.1414	81	353	369	AGDVITAVNGETVQDPR					Mascot
2307.167	2307.3369	0.1699	74	80	100	GPQFFGPPGFDQLPDGH					Mascot

2515.3303	2515.5027	0.1724	69	208	233	PLKR VGDWVVAVGNPFGLGG TVTSGIVSAR					Mascot

Rank	Protein	Name	Accession No.	Pep. Count	Protein Score	Protein Score C. I. %	Total Ion Score	Best Ion Score	Best Ion C. I. %	Total Ion C. I. %	Confirmed	Sample Name	Customer sample Name
5	unnamed protein product [Brucella suis 1330]		gi|23501930|ref|NP_698057.1|	16	58	97.983					.T.	AlDahouk_6214_Z19769-52_T1	554

Peptide Information

Calc. Mass	Obsrv. Mass	± da	± ppm	Start Seq.	End Seq.	Sequence	Ion Score	C. I. %	Modification	Rank	Result Type
705.4154	705.415	-0.0004	-1	42	46	QRVFR					Mascot
715.4348	715.401	-0.0338	-47	149	155	IIGLGDK					Mascot
728.4526	728.4246	-0.028	-38	157	162	RPKTAR					Mascot
731.4423	731.4117	-0.0306	-42	520	524	RPFRR					Mascot
732.4138	732.4071	-0.0067	-9	78	83	EIELTK					Mascot
743.4522	743.4324	-0.0198	-27	443	448	LRAVER					Mascot
774.3965	774.4592	0.0627	81	527	533	RSNGGQR					Mascot
810.4216	810.4861	0.0645	80	471	478	GPAPQGQR					Mascot
816.4587	816.5278	0.0691	85	487	493	APAHKHR					Mascot
830.4842	830.4276	-0.0566	-68	445	451	AVERVTR					Mascot
868.5363	868.5928	0.0565	65	286	292	LLRDPVR					Mascot
1277.6821	1277.7983	0.1162	91	494	504	RPAQKAGEQHR					Mascot
1529.8243	1529.9052	0.0809	53	71	83	ECAVPLKEIELTK			Carbamidomethyl (C)[2]		Mascot
1643.8248	1643.9341	0.1093	66	355	369	YDVAAIHGNKSQNAR					Mascot
1660.9268	1660.9529	0.0261	16	84	100	ENTGGFAALGITGVLLK					Mascot
2009.1753	2009.1393	-0.036	-18	186	205	SAHISTALVLGGVSKLSQIK					Mascot

**Gel Idx/Pos**	210/I10	**Instr./Gel Origin**	AK043/Div_120507	**Process Status**	Analysis Succeeded
**Plate [#] Name**	[1] 1300017700	**Instrument Sample Name**		**Spectra**	4

Rank	Protein	Name	Accession No.	Pep. Count	Protein Score	Protein Score C. I. %	Total Ion Score	Best Ion Score	Best Ion C. I. %	Total Ion C. I. %	Confirmed	Sample Name	Customer sample Name
1	secB gene product [Brucella suis 1330]		gi|23502920|ref|NP_699047.1|	6	229	100	189	86	100	100	.T.	AlDahouk_6214_Z19769-55_T1	560

Peptide Information

Calc. Mass	Obsrv. Mass	± da	± ppm	Start Seq.	End Seq.	Sequence	Ion Score	C. I. %	Modification	Rank	Result Type

Calc. Mass	Obsrv. Mass	± da	± ppm	Start Seq.	End Seq.	Sequence	Ion Score	C. I. %	Modification	Rank	Result Type
863.5137	863.5877	0.074	86	116	122	LLFPFAR	38	99.946			Mascot
863.5137	863.5877	0.074	86	116	122	LLFPFAR					Mascot
887.4944	887.5656	0.0712	80	123	130	QIIADATR					Mascot
1741.9232	1742.0713	0.1481	85	30	45	DLSFESPGAPLSLRPR	86	100			Mascot
1741.9232	1742.0713	0.1481	85	30	45	DLSFESPGAPLSLRPR					Mascot
1742.9111	1743.0673	0.1562	90	82	96	DILFNTELVYGGVFR	65	100			Mascot
1742.9111	1743.0673	0.1562	90	82	96	DILFNTELVYGGVFR					Mascot
2213.1602	2213.3381	0.1779	80	77	96	AVDGKDILFNTELVYGGV FR					Mascot
2291.2039	2291.3818	0.1779	78	97	115	IQGIPQEHMLPLLFIECPR			Carbamidomethyl (C)[17]		Mascot
2307.1987	2307.3892	0.1905	83	97	115	IQGIPQEHMLPLLFIECPR			Carbamidomethyl (C)[17], Oxidation (M)[9]		Mascot

Gel Idx/Pos	211/I11	Instr./Gel Origin	AK043/Div_120507	Process Status	Analysis Succeeded
Plate [#] Name	[1] 1300017700	Instrument Sample Name		Spectra	4

Rank	Protein Name	Accession No.	Pep. Count	Protein Score	Protein Score C. I. %	Total Ion Score	Best Ion Score	Best Ion C. I. %	Total Ion C. I. %	Confirmed	Sample Name	Customer sample Name
1	chaperonin GroEL [Brucella suis ATCC 23445]	gi|163844383|ref|YP_001622038.1|	25	364	100	206	128	100	100	.T.	AlDahouk_6214_Z19769-59_T1	1416

Peptide Information

Calc. Mass	Obsrv. Mass	± da	± ppm	Start Seq.	End Seq.	Sequence	Ion Score	C. I. %	Modification	Rank	Result Type
707.3947	707.3979	0.0032	5	8	13	FGRTAR					Mascot
719.347	719.3949	0.0479	67	278	284	APGFGDR					Mascot
721.3991	721.3956	-0.0035	-5	5	10	DVKFGR					Mascot
726.4257	726.4737	0.048	66	445	451	AIQAPAR					Mascot
819.4934	819.4813	-0.0121	-15	422	429	ASTKITAK					Mascot
855.541	855.613	0.072	84	372	380	LAGGVAVIR					Mascot
875.4482	875.5212	0.073	83	278	285	APGFGDRR					Mascot
882.5268	882.6041	0.0773	88	444	451	RAIQAPAR					Mascot
921.4532	921.5233	0.0701	76	161	168	MIAEAMQK					Mascot
974.4901	974.5722	0.0821	84	396	404	VDDALNATR					Mascot
1233.6508	1233.7513	0.1005	81	309	319	LESVTLDMLGR					Mascot
1245.6685	1245.7301	0.0616	49	381	392	VGGATEVEVKEK					Mascot
1249.6456	1249.7499	0.1043	83	309	319	LESVTLDMLGR			Oxidation (M)[8]		Mascot
1344.7004	1344.8148	0.1144	85	169	181	VGNEGVITVEEAK					Mascot

1405.6926	1405.8127	0.1201	85	106	118	AVAAGMNPMDLKR			Oxidation (M)[6,9]		Mascot
1455.755	1455.8772	0.1222	84	430	443	GVNADQEAGINIVR	78	100			Mascot
1455.755	1455.8772	0.1222	84	430	443	GVNADQEAGINIVR					Mascot
1514.7526	1514.8835	0.1309	86	198	210	GYLSPYFVTNPEK					Mascot
1582.905	1583.0375	0.1325	84	119	133	GIDLAVNEVVAELLK					Mascot
1583.8751	1584.0121	0.137	86	405	421	AAVEEGIVAGGGTALLR	128	100			Mascot
1583.8751	1584.0121	0.137	86	405	421	AAVEEGIVAGGGTALLR					Mascot
1711	1711.1415	0.1415	83	119	134	GIDLAVNEVVAELLKK					Mascot
1741.7875	1741.9373	0.1498	86	351	364	QQIEETTSDYDREK					Mascot
1759.8934	1760.0426	0.1492	85	211	225	MVADLEDAYILLHEK					Mascot
1775.8884	1776.0463	0.1579	89	211	225	MVADLEDAYILLHEK			Oxidation (M)[1]		Mascot
1853.8585	1854.0133	0.1548	84	182	197	TAETELEVVEGMQFDR					Mascot
1869.8535	1870.0204	0.1669	89	182	197	TAETELEVVEGMQFDR			Oxidation (M)[12]		Mascot
2038.9572	2039.1238	0.1666	82	59	75	EVELEDKFENMGAQMLR					Mascot
2054.9521	2055.1331	0.181	88	59	75	EVELEDKFENMGAQMLR			Oxidation (M)[11]		Mascot
2070.947	2071.1282	0.1812	87	59	75	EVELEDKFENMGAQMLR			Oxidation (M)[11,15]		Mascot
2419.1946	2419.405	0.2104	87	81	105	TNDTAGDGTTTATVLGQ AIVQEGAK					Mascot
2445.2466	2445.4512	0.2046	84	137	160	KINTSEEVAQVGTISANG EAEIGK					Mascot

2 hslU gene product [Brucella suis 1330] gi|23502927|ref|NP_699054.1| 24 211 100 76 76 100 100 .T. AlDahouk_6214_Z19769-59_T1 1416

Peptide Information

Calc. Mass	Obsrv. Mass	± da	± ppm	Start Seq.	End Seq.	Sequence	Ion Score	C. I. %	Modification	Rank	Result Type
703.3733	703.4192	0.0459	65	139	145	TASPATR					Mascot
705.4293	705.4466	0.0173	25	16	21	FIIGQK					Mascot
707.347	707.3979	0.0509	72	2	7	SNFSPR					Mascot
713.4668	713.5154	0.0486	68	26	32	AVAIALR					Mascot
722.358	722.4039	0.0459	64	329	333	EDFRR					Mascot
761.4264	761.4563	0.0299	39	64	69	TEISRR					Mascot
802.4821	802.5312	0.0491	61	73	80	LAGAPFVK					Mascot
869.5679	869.6371	0.0692	80	25	32	RAVAIALR					Mascot
872.4836	872.555	0.0714	82	95	101	DVEQIIR					Mascot
888.5149	888.5861	0.0712	80	321	328	VELSALTR					Mascot
939.4642	939.5443	0.0801	85	123	130	AHLNAEER					Mascot

960.4996	960.5795	0.0799	83	8	15	EIVSELDR				Mascot
989.4833	989.5684	0.0851	86	38	45	QQLEGQMR				Mascot
1027.5208	1027.6071	0.0863	84	86	94	FTEVGYVGR				Mascot
1217.6987	1217.7925	0.0938	77	334	344	ILTETEASLIK				Mascot
1241.7463	1241.8497	0.1034	83	102	112	DLVEIAITLVR	76	100		Mascot
1241.7463	1241.8497	0.1034	83	102	112	DLVEIAITLVR				Mascot
1334.6838	1334.7939	0.1101	82	393	404	VLDEISFTAPDK				Mascot
1339.7256	1339.8373	0.1117	83	405	417	AGATFIIDAAYVK				Mascot
1373.7999	1373.8519	0.052	38	333	344	RILTETEASLIK				Mascot
1646.9111	1647.0461	0.135	82	8	21	EIVSELDRFIIGQK				Mascot
1707.8323	1707.9777	0.1454	85	237	251	VSEDEGIVFIDEIDK				Mascot
2119.0918	2119.2668	0.175	83	237	255	VSEDEGIVFIDEIDKIAAR				Mascot
2978.5735	2978.801	0.2275	76	290	316	TDHILFITSGAFHVSKPSD LLPELQGR				Mascot
3030.5266	3030.7681	0.2415	80	211	236	DSYPILINDESDKLLDQD QIVQEALR				Mascot

3 pyk gene product [Brucella suis 1330] gi|23502605|ref|NP_698732.1| 15 78 99.981 .T. AlDahouk_6214_Z19769-59_T1 1416

Peptide Information

Calc. Mass	Obsrv. Mass	± da	± ppm	Start Seq.	End Seq.	Sequence	Ion Score	C. I. %	Modification	Rank	Result Type
802.4669	802.5312	0.0643	80	363	369	QIAETLK					Mascot
819.4756	819.4813	0.0057	7	210	217	GKVGLMSK					Mascot
1041.6051	1041.691	0.0859	82	218	226	IEKPQAVTR					Mascot
1124.5735	1124.6674	0.0939	83	26	35	LFEAGADVFR					Mascot
1252.6685	1252.772	0.1035	83	25	35	KLFEAGADVFR					Mascot
1452.842	1452.9642	0.1222	84	393	406	TPIIALSPVVDTAR					Mascot
1596.8955	1597.0256	0.1301	81	9	24	ILATLGPASGEEAVIR					Mascot
1610.8749	1611.0094	0.1345	83	370	385	LSAIVTYTASGTTGLR					Mascot
1676.9694	1677.1058	0.1364	81	59	74	ELGRPIGILADLQGPK					Mascot
1687.8934	1688.031	0.1376	82	227	241	LDEIIELSDALMVAR					Mascot
1724.9905	1724.908	-0.0825	-48	9	25	ILATLGPASGEEAVIRK					Mascot
1915.047	1915.1434	0.0964	50	448	466	VIITAGVPFGTPGATNML R					Mascot
1931.0419	1931.1536	0.1117	58	448	466	VIITAGVPFGTPGATNML R			Oxidation (M)[17]		Mascot
1940.0388	1940.1957	0.1569	81	107	123	VFLPHPEILEAVEPGHR					Mascot
2219.1304	2219.3159	0.1855	84	328	346	IAEQVEREPTYSTIIDAQR					Mascot

Calc. Mass	Obsrv. Mass	± da	± ppm	Start Seq.	End Seq.	Sequence	Ion Score	C. I. %	Modification	Rank	Result Type
2404.1626	2404.363	0.2004	83	85	106	VDLVPGQTFTLDNNEAL GDETR					Mascot

Rank	Protein Name	Accession No.	Pep. Count	Protein Score	Protein Score C. I. %	Total Ion Score	Best Ion Score	Best Ion C. I. %	Total Ion C. I. %	Confirmed	Sample Name	Customer sample Name
4	pyruvate kinase [Brucella suis ATCC 23445]	gi|163845326|ref|YP_001622981.1|	14	68	99.807					.T.	AlDahouk_6214_Z19769-59_T1	1416

Peptide Information

Calc. Mass	Obsrv. Mass	± da	± ppm	Start Seq.	End Seq.	Sequence	Ion Score	C. I. %	Modification	Rank	Result Type
802.4669	802.5312	0.0643	80	363	369	QIAETLK					Mascot
819.4756	819.4813	0.0057	7	210	217	GKVGLMSK					Mascot
1041.6051	1041.691	0.0859	82	218	226	IEKPQAVTR					Mascot
1124.5735	1124.6674	0.0939	83	26	35	LFEAGADVFR					Mascot
1252.6685	1252.772	0.1035	83	25	35	KLFEAGADVFR					Mascot
1452.842	1452.9642	0.1222	84	393	406	TPIIALSPVVDTAR					Mascot
1596.8955	1597.0256	0.1301	81	9	24	ILATLGPASGEEAVIR					Mascot
1610.8749	1611.0094	0.1345	83	370	385	LSAIVTYTASGTTGLR					Mascot
1676.9694	1677.1058	0.1364	81	59	74	ELGRPIGILADLQGPK					Mascot
1687.8934	1688.031	0.1376	82	227	241	LDEIIELSDALMVAR					Mascot
1724.9905	1724.908	-0.0825	-48	9	25	ILATLGPASGEEAVIRK					Mascot
1915.047	1915.1434	0.0964	50	448	466	VIITAGVPFGTPGATNML R					Mascot
1931.0419	1931.1536	0.1117	58	448	466	VIITAGVPFGTPGATNML R			Oxidation (M)[17]		Mascot
1940.0388	1940.1957	0.1569	81	107	123	VFLPHPEILEAVEPGHR					Mascot
2404.1626	2404.363	0.2004	83	85	106	VDLVPGQTFTLDNNEAL GDETR					Mascot

Gel Idx/Pos	213/I13	Instr./Gel Origin	AK043/Div_120507	Process Status	Analysis Succeeded
Plate [#] Name	[1] 1300017700	Instrument Sample Name		Spectra	4

Rank	Protein Name	Accession No.	Pep. Count	Protein Score	Protein Score C. I. %	Total Ion Score	Best Ion Score	Best Ion C. I. %	Total Ion C. I. %	Confirmed	Sample Name	Customer sample Name
1	chaperonin GroEL [Brucella suis ATCC 23445]	gi|163844383|ref|YP_001622038.1|	29	568	100	364	128	100	100	.T.	AlDahouk_6214_Z19769-72_T1	420

Peptide Information

Calc. Mass	Obsrv. Mass	± da	± ppm	Start Seq.	End Seq.	Sequence	Ion Score	C. I. %	Modification	Rank	Result Type
707.3947	707.4149	0.0202	29	8	13	FGRTAR					Mascot

719.347	719.3977	0.0507	70	278	284	APGFGDR				Mascot
721.3991	721.4058	0.0067	9	5	10	DVKFGR				Mascot
726.4257	726.476	0.0503	69	445	451	AIQAPAR				Mascot
778.4127	778.4498	0.0371	48	1	7	MAAKDVK			Oxidation (M)[1]	Mascot
802.4417	802.4943	0.0526	66	363	368	EKLQER				Mascot
855.541	855.6165	0.0755	88	372	380	LAGGVAVIR				Mascot
875.4482	875.5244	0.0762	87	278	285	APGFGDRR				Mascot
882.5268	882.6068	0.08	91	444	451	RAIQAPAR				Mascot
974.4901	974.5751	0.085	87	396	404	VDDALNATR				Mascot
1000.5673	1000.649	0.0817	82	19	28	GVDILADAVK				Mascot
1233.6508	1233.755	0.1042	84	309	319	LESVTLDMLGR				Mascot
1245.6685	1245.7314	0.0629	50	381	392	VGGATEVEVKEK				Mascot
1249.6456	1249.7535	0.1079	86	309	319	LESVTLDMLGR			Oxidation (M)[8]	Mascot
1344.7004	1344.8185	0.1181	88	169	181	VGNEGVITVEEAK				Mascot
1373.7028	1373.8326	0.1298	94	106	118	AVAAGMNPMDLKR				Mascot
1389.6978	1389.8207	0.1229	88	106	118	AVAAGMNPMDLKR			Oxidation (M)[6]	Mascot
1405.6926	1405.8246	0.132	94	106	118	AVAAGMNPMDLKR			Oxidation (M)[6,9]	Mascot
1455.755	1455.881	0.126	87	430	443	GVNADQEAGINIVR	117	100		Mascot
1455.755	1455.881	0.126	87	430	443	GVNADQEAGINIVR				Mascot
1514.7526	1514.8899	0.1373	91	198	210	GYLSPYFVTNPEK				Mascot
1582.905	1583.0453	0.1403	89	119	133	GIDLAVNEVVAELLK				Mascot
1583.8751	1584.0173	0.1422	90	405	421	AAVEEGIVAGGGTALLR	128	100		Mascot
1583.8751	1584.0173	0.1422	90	405	421	AAVEEGIVAGGGTALLR				Mascot
1616.849	1616.9896	0.1406	87	452	467	QITTNAGEEASVIVGK				Mascot
1711	1711.15	0.15	88	119	134	GIDLAVNEVVAELLKK				Mascot
1741.7875	1741.9426	0.1551	89	351	364	QQIEETTSDYDREK				Mascot
1759.8934	1760.0504	0.157	89	211	225	MVADLEDAYILLHEK				Mascot
1775.8884	1776.0519	0.1635	92	211	225	MVADLEDAYILLHEK			Oxidation (M)[1]	Mascot
1853.8585	1854.0229	0.1644	89	182	197	TAETELEVVEGMQFDR	118	100		Mascot
1853.8585	1854.0229	0.1644	89	182	197	TAETELEVVEGMQFDR				Mascot
1869.8535	1870.0245	0.171	91	182	197	TAETELEVVEGMQFDR			Oxidation (M)[12]	Mascot
1903.9834	1904.1608	0.1774	93	211	226	MVADLEDAYILLHEKK			Oxidation (M)[1]	Mascot
2038.9572	2039.1353	0.1781	87	59	75	EVELEDKFENMGAQMLR				Mascot
2054.9521	2055.1379	0.1858	90	59	75	EVELEDKFENMGAQMLR			Oxidation (M)[11]	Mascot
2070.947	2071.1355	0.1885	91	59	75	EVELEDKFENMGAQMLR			Oxidation (M)[11,15]	Mascot
2419.1946	2419.4138	0.2192	91	81	105	TNDTAGDGTTTATVLGQ				Mascot

						AIVQEGAK	
2445.2466	2445.4722	0.2256	92	137	160	KINTSEEVAQVGTISANG EAEIGK	Mascot
3329.6423	3329.9519	0.3096	93	468	498	ILENTSETFGYNTANGEY GDLISLGIVDPVK	Mascot

TOPLAB
Gesellschaft für angewandte Biotechnologie mbH

Analysis Information

**Report Type**	Protein-Peptide Summary by Spot	**Analysis Type**	MS
**Sample Set Name**	Div_120507_MS	**Database**	Bruc_suis_DEC2011
**Analysis Name**	AlDahouk_6214 Analyse 5	**Creation Date**	08/16/2012 10:32:20
**Reported By**	08/21/2012 19:15:02 - admin	**Last Modified**	08/16/2012 10:54:37

MS Acq. : Proc. Methods (Unspecified) : (Unspecified)

**Gel Idx/Pos**	249/K1	**Instr./Gel Origin**	AK043/Div_120507	**Process Status**	Analysis Succeeded
**Plate [#] Name**	[1] 1300017700	**Instrument Sample Name**		**Spectra**	1

Rank	Protein Name	Species	Accession No.	Protein MW	Protein PI	Pep. Count	Protein Score	Protein Score C. I. %	Confirmed	Sample Name	Customer sample Name
1	sucA gene product [Brucella suis 1330]		gi|23502773|ref|NP_698900.1|	113009.4	6.24	29	143	100	.T.	AlDahouk_6214_Z19769-40_T1	93
2	2-oxoglutarate dehydrogenase E1 component [Brucella suis ATCC 23445]		gi|163843946|ref|YP_001628350.1|	113035.4	6.24	28	134	100	.T.	AlDahouk_6214_Z19769-40_T1	93

**Gel Idx/Pos**	253/K5	**Instr./Gel Origin**	AK043/Div_120507	**Process Status**	Analysis Succeeded
**Plate [#] Name**	[1] 1300017700	**Instrument Sample Name**		**Spectra**	1

Rank	Protein Name	Species	Accession No.	Protein MW	Protein PI	Pep. Count	Protein Score	Protein Score C. I. %	Confirmed	Sample Name	Customer sample Name
1	integral membrane sensor signal transduction histidine kinase [Brucella suis ATCC 23445]		gi|163842888|ref|YP_001627292.1|	50824.7	5.51	14	64	99.469	.T.	AlDahouk_6214_Z19769-43_T1	99
2	hypothetical protein BSUIS_A0965 [Brucella suis ATCC 23445]		gi|163843194|ref|YP_001627598.1|	122546.9	8.11	20	61	98.965	.T.	AlDahouk_6214_Z19769-43_T1	99
3	unnamed protein product [Brucella suis 1330]		gi|23501500|ref|N	50805.7	5.49	13	56	97.084	.T.	AlDahouk_6	99

		P_697627.1|							214_Z19769-43_T1	

Gel Idx/Pos	262/K14	Instr./Gel Origin	AK043/Div_120507	Process Status	Analysis Succeeded
Plate [#] Name	[1] 1300017700	Instrument Sample Name		Spectra	1

Rank	Protein Name	Species	Accession No.	Protein MW	Protein PI	Pep. Count	Protein Score	Protein Score C. I. %	Confirmed	Sample Name	Customer sample Name
1	ntrX gene product [Brucella suis 1330]		gi|23501993|ref|NP_698120.1|	50103.4	5.64	25	187	100	.T.	AlDahouk_6214_Z19769-53_T1	542 left part
2	nitrogen assimilation regulatory protein ntrX [Brucella suis ATCC 23445]		gi|163843383|ref|YP_001627787.1|	50094.4	5.58	24	176	100	.T.	AlDahouk_6214_Z19769-53_T1	542 left part

Gel Idx/Pos	269/K21	Instr./Gel Origin	AK043/Div_120507	Process Status	Analysis Succeeded
Plate [#] Name	[1] 1300017700	Instrument Sample Name		Spectra	1

Rank	Protein Name	Species	Accession No.	Protein MW	Protein PI	Pep. Count	Protein Score	Protein Score C. I. %	Confirmed	Sample Name	Customer sample Name
1	accC gene product [Brucella suis 1330]		gi|23501792|ref|NP_697919.1|	49617.3	5.84	13	73	99.928	.T.	AlDahouk_6214_Z19769-61_T1	584
2	unnamed protein product [Brucella suis 1330]		gi|23501539|ref|NP_697666.1|	84035.8	8.33	18	65	99.607	.T.	AlDahouk_6214_Z19769-61_T1	584
3	argG gene product [Brucella suis 1330]		gi|23500989|ref|NP_697116.1|	45225.6	6	11	55	96.244	.T.	AlDahouk_6214_Z19769-61_T1	584
4	RelA/SpoT family protein [Brucella suis ATCC 23445]		gi|163842924|ref|YP_001627328.1|	84031.8	8.55	16	55	95.881	.T.	AlDahouk_6214_Z19769-61_T1	584

Gel Idx/Pos<br>Plate [#] Name	270/K22<br>[1] 1300017700	Instr./Gel Origin<br>Instrument Sample Name	AK043/Div_120507	Process Status<br>Spectra	Analysis Succeeded<br>1

Rank	Protein	Name	Species	Accession No.	Protein MW	Protein PI	Pep. Count	Protein Score	Protein Score C. I. %	Confirmed	Sample Name	Customer sample Name
1	rpoB gene product [Brucella suis 1330]			gi|23502120|ref|NP_698247.1|	153829.1	5.04	32	159	100	.T.	AlDahouk_6214_Z19769-62_T1	25
	**Protein Group**											
	DNA-directed RNA polymerase subunit beta [Brucella suis ATCC 23445]			gi|163843508|ref|YP_001627912.1|	153857.1	5.03999996185303						

Analysis Information

**Report Type**	Protein-Peptide Summary by Spot	**Analysis Type**	MS
**Sample Set Name**	Div_120507_MS	**Database**	Bruc_suis_DEC2011
**Analysis Name**	AlDahouk_6214 Analyse 5	**Creation Date**	08/16/2012 10:32:20
**Reported By**	08/21/2012 19:13:39 - admin	**Last Modified**	08/16/2012 10:54:37

MS Acq. : Proc. Methods (Unspecified) : (Unspecified)

**Gel Idx/Pos**	249/K1	**Instr./Gel Origin**	AK043/Div_120507	**Process Status**	Analysis Succeeded
**Plate [#] Name**	[1] 1300017700	**Instrument Sample Name**		**Spectra**	1

Rank	Protein Name	Species	Accession No.	Protein MW	Protein PI	Pep. Count	Protein Score	Protein Score C. I. %	Confirmed	Sample Name	Customer sample Name
1	sucA gene product [Brucella suis 1330]		gi|23502773|ref|NP_698900.1|	113009.4	6.24	29	143	100	.T.	AlDahouk_6214_Z19769-40_T1	93

Peptide Information

Calc. Mass	Obsrv. Mass	± da	± ppm	Start Seq.	End Seq.	Sequence	Modification
1027.5353	1027.5692	0.0339	33	297	305	EVVLGMAHR	Oxidation (M)[6]
1063.5782	1063.5927	0.0145	14	906	914	GIDDVYLLR	
1102.6255	1102.6356	0.0101	9	699	707	YIPLNNLQK	
1191.6409	1191.6519	0.011	9	915	924	VEQLYPFPAK	
1249.5735	1249.5895	0.016	13	894	902	VYYDLYEER	
1330.6538	1330.6669	0.0131	10	583	594	ADWLDGAWAGLR	
1334.637	1334.6412	0.0042	3	392	402	TRDDMVPLSER	Oxidation (M)[5]
1468.7794	1468.7961	0.0167	11	991	1004	HLAQLAAFLEDALG	
1475.8118	1475.8148	0.003	2	621	632	LVEVPKDFHVHR	
1478.826	1478.7887	-0.0373	-25	308	320	LNVLSQVMGKPHR	
1494.8209	1494.8232	0.0023	2	308	320	LNVLSQVMGKPHR	Oxidation (M)[8]
1516.7139	1516.7308	0.0169	11	687	698	HTVLYDQENQNR	
1557.8119	1557.8135	0.0016	1	546	559	LIAEGLVTQDDIDR	
1620.6925	1620.7163	0.0238	15	36	48	YEDDPNSVDPQWR	

1637.842	1637.8567	0.0147	9	251	264	LIEAEGFEQFIDVK	
1656.8551	1656.866	0.0109	7	113	129	GAAGTPLTAEEITQAAR	
1664.8424	1664.856	0.0136	8	974	990	YAGRPAAASPATGLMSK	Oxidation (M)[15]
1695.7721	1695.7864	0.0143	8	344	358	YHLGASSDREFDGNK	
1704.8551	1704.8667	0.0116	7	847	862	AISTLAELSGESSFHR	
1807.8861	1807.8903	0.0042	2	863	877	LLWDDAQYNKDEGIK	
1832.9423	1832.9377	-0.0046	-3	546	561	LIAEGLVTQDDIDRMK	Oxidation (M)[15]
1886.0269	1886.0331	0.0062	3	271	288	FGLDGGESLIPALEQIVK	
1952.9712	1952.968	-0.0032	-2	566	582	QKLEGEFEAGQSYKPNK	
2042.1281	2042.1104	-0.0177	-9	271	289	FGLDGGESLIPALEQIVKR	
2112.9331	2112.9624	0.0293	14	513	530	FGHNEGDEPSFTQPLMYK	Oxidation (M)[16]
2386.1748	2386.137	-0.0378	-16	469	490	MIEAPIFHVNGDDPEAVVFAAK	Oxidation (M)[1]
2518.3201	2518.3113	-0.0088	-3	436	458	VAGTLHFIINNQIGFTTNPAFSR	
2805.533	2805.4258	-0.1072	-38	405	432	VLPLLLHGDAAFAGQGVVAECLGLS GLK	Carbamidomethyl (C)[21]
2921.4297	2921.3374	-0.0923	-32	768	793	WLRMSGLVCLLPHGFEGQGPEHS SAR	Carbamidomethyl (C)[9]
3201.564	3201.5671	0.0031	1	738	766	ALVLWEAQFGDFANGAQVVFDQFI SSGER	

2	2-oxoglutarate dehydrogenase E1 component [Brucella suis ATCC 23445]	gi|163843946|ref|YP_001628350.1|	113035.4	6.24	28	134	100	.T.	AlDahouk_6214_Z19769-40_T1	93

Peptide Information

Calc. Mass	Obsrv. Mass	± da	± ppm	Start Seq.	End Seq.	Sequence	Modification
1027.5353	1027.5692	0.0339	33	297	305	EVVLGMAHR	Oxidation (M)[6]
1063.5782	1063.5927	0.0145	14	906	914	GIDDVYLLR	
1102.6255	1102.6356	0.0101	9	699	707	YIPLNNLQK	
1191.6409	1191.6519	0.011	9	915	924	VEQLYPFPAK	
1249.5735	1249.5895	0.016	13	894	902	VYYDLYEER	
1330.6538	1330.6669	0.0131	10	583	594	ADWLDGAWAGLR	
1468.7794	1468.7961	0.0167	11	991	1004	HLAQLAAFLEDALG	
1475.8118	1475.8148	0.003	2	621	632	LVEVPKDFHVHR	
1478.826	1478.7887	-0.0373	-25	308	320	LNVLSQVMGKPHR	
1494.8209	1494.8232	0.0023	2	308	320	LNVLSQVMGKPHR	Oxidation (M)[8]
1516.7139	1516.7308	0.0169	11	687	698	HTVLYDQENQNR	
1557.8119	1557.8135	0.0016	1	546	559	LIAEGLVTQDDIDR	

1620.6925	1620.7163	0.0238	15	36	48	YEDDPNSVDPQWR	
1637.842	1637.8567	0.0147	9	251	264	LIEAEGFEQFIDVK	
1656.8551	1656.866	0.0109	7	113	129	GAAGTPLTAEEITQAAR	
1664.8424	1664.856	0.0136	8	974	990	YAGRPAAASPATGLMSK	Oxidation (M)[15]
1695.7721	1695.7864	0.0143	8	344	358	YHLGASSDREFDGNK	
1704.8551	1704.8667	0.0116	7	847	862	AISTLAELSGESSFHR	
1807.8861	1807.8903	0.0042	2	863	877	LLWDDAQYNKDEGIK	
1832.9423	1832.9377	-0.0046	-3	546	561	LIAEGLVTQDDIDRMK	Oxidation (M)[15]
1886.0269	1886.0331	0.0062	3	271	288	FGLDGGESLIPALEQIVK	
1952.9712	1952.968	-0.0032	-2	566	582	QKLEGEFEAGQSYKPNK	
2042.1281	2042.1104	-0.0177	-9	271	289	FGLDGGESLIPALEQIVKR	
2112.9331	2112.9624	0.0293	14	513	530	FGHNEGDEPSFTQPLMYK	Oxidation (M)[16]
2386.1748	2386.137	-0.0378	-16	469	490	MIEAPIFHVNGDDPEAVVFAAK	Oxidation (M)[1]
2518.3201	2518.3113	-0.0088	-3	436	458	VAGTLHFIINNQIGFTTNPAFSR	
2805.533	2805.4258	-0.1072	-38	405	432	VLPLLLHGDAAFAGQGVVAECLGLS GLK	Carbamidomethyl (C)[21]
2921.4297	2921.3374	-0.0923	-32	768	793	WLRMSGLVCLLPHGFEGQGPEHS SAR	Carbamidomethyl (C)[9]
3201.564	3201.5671	0.0031	1	738	766	ALVLWEAQFGDFANGAQVVFDQFI SSGER	

Gel Idx/Pos<br>Plate [#] Name	253/K5<br>[1] 1300017700	Instr./Gel Origin<br>Instrument Sample Name	AK043/Div_120507	Process Status<br>Spectra	Analysis Succeeded<br>1

Rank	Protein Name	Species	Accession No.	Protein MW	Protein PI	Pep. Count	Protein Score	Protein Score C. I. %	Confirmed	Sample Name	Customer sample Name
1	integral membrane sensor signal transduction histidine kinase [Brucella suis ATCC 23445]		gi|163842888|ref|YP_001627292.1|	50824.7	5.51	14	64	99.469	.T.	AlDahouk_6214_Z19769-43_T1	99

Peptide Information

Calc. Mass	Obsrv. Mass	± da	± ppm	Start Seq.	End Seq.	Sequence	Modification
856.5363	856.5348	-0.0015	-2	254	260	TPLTRLR	
993.4669	993.5526	0.0857	86	202	210	IMDGDLTGR	Oxidation (M)[2]
1028.5986	1028.5946	-0.004	-4	234	242	ILELNEGLK	
1035.5581	1035.5824	0.0243	23	414	421	FVRLEESR	
1106.6125	1106.5785	-0.034	-31	377	386	TATVTLLMEK	
1118.559	1118.5658	0.0068	6	151	159	DLGEPERFR	
1130.5702	1130.5947	0.0245	22	408	416	DHATERFVR	

1165.6398	1165.6449	0.0051	4	293	302	TFNAILMISR	
1187.6565	1187.6863	0.0298	25	223	233	LSGNLNVMLAR	
1316.7433	1316.6649	-0.0784	-60	60	71	SYARGGIPQLVR	
1340.7719	1340.7365	-0.0354	-26	133	145	AIAVVIALPNGMR	Oxidation (M)[12]
1365.7372	1365.7108	-0.0264	-19	393	406	IVVADNGPGIPADK	
1657.9305	1657.879	-0.0515	-31	422	438	TQPGSGLGLSLAKAVMK	
1796.921	1797.0972	0.1762	98	370	386	YAGGEGRTATVTLLMEK	

2 hypothetical protein BSUIS_A0965 [Brucella suis ATCC 23445] gi|163843194|ref|YP_001627598.1| 122546.9 8.11 20 61 98.965 .T. AlDahouk_6214_Z19769-43_T1 99

Peptide Information

Calc. Mass	Obsrv. Mass	± da	± ppm	Start Seq.	End Seq.	Sequence	Modification
864.5189	864.5431	0.0242	28	1109	1116	GLLGITYK	
875.5057	875.5406	0.0349	40	36	43	SARSIVSR	
973.5564	973.5773	0.0209	21	727	734	IKLEIDDK	
993.4748	993.5526	0.0778	78	571	579	GTAYTPNNR	
1001.5261	1001.5934	0.0673	67	253	261	IALDGDIER	
1028.5596	1028.5946	0.035	34	341	349	VDNVRGNVR	
1031.5731	1031.6449	0.0718	70	873	881	QISDLEAKK	
1033.4983	1033.5686	0.0703	68	709	717	VSMTEPVDR	
1065.5687	1065.6211	0.0524	49	263	272	ASDKALSAFR	
1107.6772	1107.5934	-0.0838	-76	561	570	GAYTTIKLLK	
1118.6681	1118.5658	-0.1023	-91	267	276	ALSAFRLNVK	
1193.6273	1193.6764	0.0491	41	862	872	GSQFDARALTK	
1201.6576	1201.6963	0.0387	32	541	551	FDVVGELPAVR	
1248.6444	1248.6882	0.0438	35	858	868	VNVRGSQFDAR	
1262.6964	1262.657	-0.0394	-31	350	360	LSLEHRVGSHK	
1300.7736	1300.703	-0.0706	-54	240	251	IAITGTVVWKGR	
1302.6648	1302.749	0.0842	65	1027	1037	DIDVSRVDVER	
1316.7421	1316.6649	-0.0772	-59	869	880	ALTKQISDLEAK	
1320.7634	1320.651	-0.1124	-85	207	218	VIRIANSSVSFK	
1390.78	1390.7473	-0.0327	-24	1045	1057	GQGYLNLSKGVVR	

3 unnamed protein product [Brucella suis 1330] gi|23501500|ref|NP_697627.1| 50805.7 5.49 13 56 97.084 .T. AlDahouk_6214_Z19769-43_T1 99

Peptide Information

Calc. Mass	Obsrv. Mass	± da	± ppm	Start Seq.	End Seq.	Sequence	Modification
856.5363	856.5348	-0.0015	-2	254	260	TPLTRLR	
993.4669	993.5526	0.0857	86	202	210	IMDGDLTGR	Oxidation (M)[2]
1028.5986	1028.5946	-0.004	-4	234	242	ILELNEGLK	
1035.5581	1035.5824	0.0243	23	414	421	FVRLEESR	
1106.6125	1106.5785	-0.034	-31	377	386	TATVTLLMEK	
1118.559	1118.5658	0.0068	6	151	159	DLGEPERFR	
1130.5702	1130.5947	0.0245	22	408	416	DHATERFVR	
1165.6398	1165.6449	0.0051	4	293	302	TFNAILMISR	
1187.6565	1187.6863	0.0298	25	223	233	LSGNLNVMLAR	
1316.7433	1316.6649	-0.0784	-60	60	71	SYARGGIPQLVR	
1340.7719	1340.7365	-0.0354	-26	133	145	AIAVVIALPNGMR	Oxidation (M)[12]
1657.9305	1657.879	-0.0515	-31	422	438	TQPGSGLGLSLAKAVMK	
1796.921	1797.0972	0.1762	98	370	386	YAGGEGRTATVTLLMEK	

Gel Idx/Pos<br>Plate [#] Name	262/K14<br>[1] 1300017700	Instr./Gel Origin<br>Instrument Sample Name	AK043/Div_120507	Process Status<br>Spectra	Analysis Succeeded<br>1

Rank	Protein Name	Species	Accession No.	Protein MW	Protein PI	Pep. Count	Protein Score	Protein Score C. I. %	Confirmed	Sample Name	Customer sample Name
1	ntrX gene product [Brucella suis 1330]		gi|23501993|ref|NP_698120.1|	50103.4	5.64	25	187	100	.T.	AlDahouk_6214_Z19769-53_T1	542 left part

Peptide Information

Calc. Mass	Obsrv. Mass	± da	± ppm	Start Seq.	End Seq.	Sequence	Modification
1119.6157	1119.7094	0.0937	84	417	425	EYLIAQINR	
1133.595	1133.6991	0.1041	92	253	261	VLVDQQFER	
1219.6906	1219.7946	0.104	85	326	336	HIAEQAGIKPR	
1314.6729	1314.7847	0.1118	85	98	108	GAYDFIEKPFK	
1327.7832	1327.8811	0.0979	74	61	72	LDGLALLDEIKK	
1419.8682	1419.9879	0.1197	84	299	312	LSVVPVQVPALAAR	
1442.8577	1442.8867	0.029	20	112	124	LILVAERALETSK	

Calc. Mass	Obsrv. Mass	± da	± ppm	Start Seq.	End Seq.	Sequence	Modification
1446.8104	1446.9298	0.1194	83	49	60	LVFLDIWLQGSR	
1470.7739	1470.8883	0.1144	78	97	108	RGAYDFIEKPFK	
1523.8217	1523.9268	0.1051	69	414	425	FEKEYLIAQINR	
1550.7445	1550.8744	0.1299	84	31	45	TAFDADSALAAINDR	
1611.7972	1611.9391	0.1419	88	16	30	DLVAGILSDEGHETR	
1615.8835	1615.9648	0.0813	50	166	181	IMITGPSGAGKELVAR	Oxidation (M)[2]
1626.8196	1626.9552	0.1356	83	313	325	REDIPSLVEFFMK	Oxidation (M)[12]
1678.8582	1678.9775	0.1193	71	394	408	APTESDQHIMALPLR	
1694.853	1694.9908	0.1378	81	394	408	APTESDQHIMALPLR	Oxidation (M)[10]
1757.9181	1758.0599	0.1418	81	190	206	ANGPFVTVNAATITPER	
1946.0011	1946.12	0.1189	61	136	153	TGDQLELVGTSLAMNQLR	
1961.9961	1962.1547	0.1586	81	136	153	TGDQLELVGTSLAMNQLR	Oxidation (M)[14]
2038.0273	2038.1345	0.1072	53	274	292	IISSTAQNLEGMIAEGTFR	
2049.0334	2049.2056	0.1722	84	338	356	IGPDAMAVLQAHSWPGNLR	Oxidation (M)[6]
2054.0222	2054.1943	0.1721	84	274	292	IISSTAQNLEGMIAEGTFR	Oxidation (M)[12]
2177.1284	2177.3101	0.1817	83	337	356	KIGPDAMAVLQAHSWPGNLR	Oxidation (M)[7]
2371.1599	2371.3416	0.1817	77	223	244	VGALEEAHGGILYLDEVADMPR	Oxidation (M)[20]
2422.1985	2422.3809	0.1824	75	371	393	GDDPDELVTADLLPAEIGDTLPR	
2499.2546	2499.4573	0.2027	81	222	244	KVGALEEAHGGILYLDEVADMPR	Oxidation (M)[21]
2507.2922	2507.3696	0.0774	31	270	292	VDVRIISSTAQNLEGMIAEGTFR	
2555.3398	2555.5195	0.1797	70	73	96	QHPELPVVMISGHGNIETAVSAIR	

2 nitrogen assimilation regulatory protein ntrX [Brucella suis ATCC 23445] gi|163843383|ref|YP_001627787.1| 50094.4 5.58 24 176 100 .T. AlDahouk_6214_Z19769-53_T1 542 left part

Peptide Information

Calc. Mass	Obsrv. Mass	± da	± ppm	Start Seq.	End Seq.	Sequence	Modification
1119.6157	1119.7094	0.0937	84	417	425	EYLIAQINR	
1133.595	1133.6991	0.1041	92	253	261	VLVDQQFER	
1314.6729	1314.7847	0.1118	85	98	108	GAYDFIEKPFK	
1327.7832	1327.8811	0.0979	74	61	72	LDGLALLDEIKK	
1419.8682	1419.9879	0.1197	84	299	312	LSVVPVQVPALAAR	
1442.8577	1442.8867	0.029	20	112	124	LILVAERALETSK	
1446.8104	1446.9298	0.1194	83	49	60	LVFLDIWLQGSR	
1470.7739	1470.8883	0.1144	78	97	108	RGAYDFIEKPFK	

1523.8217	1523.9268	0.1051	69	414	425	FEKEYLIAQINR	
1550.7445	1550.8744	0.1299	84	31	45	TAFDADSALAAINDR	
1611.7972	1611.9391	0.1419	88	16	30	DLVAGILSDEGHETR	
1615.8835	1615.9648	0.0813	50	166	181	IMITGPSGAGKELVAR	Oxidation (M)[2]
1626.8196	1626.9552	0.1356	83	313	325	REDIPSLVEFFMK	Oxidation (M)[12]
1678.8582	1678.9775	0.1193	71	394	408	APTESDQHIMALPLR	
1694.853	1694.9908	0.1378	81	394	408	APTESDQHIMALPLR	Oxidation (M)[10]
1757.9181	1758.0599	0.1418	81	190	206	ANGPFVTVNAATITPER	
1946.0011	1946.12	0.1189	61	136	153	TGDQLELVGTSLAMNQLR	
1961.9961	1962.1547	0.1586	81	136	153	TGDQLELVGTSLAMNQLR	Oxidation (M)[14]
2038.0273	2038.1345	0.1072	53	274	292	IISSTAQNLEGMIAEGTFR	
2049.0334	2049.2056	0.1722	84	338	356	IGPDAMAVLQAHSWPGNLR	Oxidation (M)[6]
2054.0222	2054.1943	0.1721	84	274	292	IISSTAQNLEGMIAEGTFR	Oxidation (M)[12]
2177.1284	2177.3101	0.1817	83	337	356	KIGPDAMAVLQAHSWPGNLR	Oxidation (M)[7]
2371.1599	2371.3416	0.1817	77	223	244	VGALEEAHGGILYLDEVADMPR	Oxidation (M)[20]
2422.1985	2422.3809	0.1824	75	371	393	GDDPDELVTADLLPAEIGDTLPR	
2499.2546	2499.4573	0.2027	81	222	244	KVGALEEAHGGILYLDEVADMPR	Oxidation (M)[21]
2507.2922	2507.3696	0.0774	31	270	292	VDVRIISSTAQNLEGMIAEGTFR	
2555.3398	2555.5195	0.1797	70	73	96	QHPELPVVMISGHGNIETAVSAIR	

Gel Idx/Pos<br>Plate [#] Name	269/K21<br>[1] 1300017700	Instr./Gel Origin<br>Instrument Sample Name	AK043/Div_120507	Process Status<br>Spectra	Analysis Succeeded<br>1

Rank	Protein	Name	Species	Accession No.	Protein MW	Protein PI	Pep. Count	Protein Score	Protein Score C. I. %	Confirmed	Sample Name	Customer sample Name
1	accC gene product [Brucella suis 1330]			gi|23501792|ref|NP_697919.1|	49617.3	5.84	13	73	99.928	.T.	AlDahouk_6214_Z19769-61_T1	584

Peptide Information

Calc. Mass	Obsrv. Mass	± da	± ppm	Start Seq.	End Seq.	Sequence	Modification
1076.4941	1076.5691	0.075	70	329	337	FSGHAIECR	Carbamidomethyl (C)[8]
1191.6256	1191.6742	0.0486	41	404	414	ALDEFVVDGVK	
1265.6776	1265.7332	0.0556	44	376	386	IPPYYDSLIGK	
1341.7373	1341.7899	0.0526	39	173	185	SAEELPIALATAR	
1347.7267	1347.7739	0.0472	35	403	414	RALDEFVVDGVK	

1642.782	1642.8672	0.0852	52	238	252	VWEEGNSPALNAEAR	
1680.8123	1680.9346	0.1223	73	28	43	TVAVHSTADADAMHVR	
1714.771	1714.9248	0.1538	90	255	269	IGMICANACAELGYR	Carbamidomethyl (C)[5,9], Oxidation (M)[3]
1899.9194	1900.0931	0.1737	91	238	254	VWEEGNSPALNAEAREK	
2038.045	2038.1331	0.0881	43	126	146	LGIPVVPGSDGGVTDEVEAAR	
2338.219	2338.3152	0.0962	41	91	111	FAEILEAHDITFIGPTASHIR	
2497.3408	2497.4358	0.095	38	292	313	LQVEHPVTEAITGIDLVHEQIR	
2930.5159	2930.6289	0.113	39	338	365	INAEDPLTFAPSPGLITHYHTPGGLGVR	

2	unnamed protein product [Brucella suis 1330]	gi|23501539|ref|NP_697666.1|	84035.8	8.33	18	65	99.607	.T.	AlDahouk_6214_Z19769-61_T1	584

Peptide Information

Calc. Mass	Obsrv. Mass	± da	± ppm	Start Seq.	End Seq.	Sequence	Modification
1128.6624	1128.6304	-0.032	-28	122	131	LLLAISEDVR	
1208.5762	1208.5925	0.0163	13	146	155	TLGVMCEDKR	Carbamidomethyl (C)[6]
1319.7067	1319.7853	0.0786	60	4	13	QYELVERVQR	
1337.7535	1337.7357	-0.0178	-13	590	602	VDPAATTPKPGKR	
1348.8423	1348.7812	-0.0611	-45	508	519	DILKPGLPRLAR	
1403.7529	1403.7941	0.0412	29	14	25	YKPDVNEALLNK	
1438.7471	1438.8185	0.0714	50	485	496	QYSGLGMRILER	Oxidation (M)[7]
1465.8234	1465.835	0.0116	8	305	317	SIHTTIIGPSRQR	
1638.8784	1638.9313	0.0529	32	276	290	ALGLIHTTWSMVPGR	
1649.8203	1649.91	0.0897	54	158	172	IAEETMDIYAPLAGR	
1654.8734	1654.9708	0.0974	59	276	290	ALGLIHTTWSMVPGR	Oxidation (M)[11]
1681.0623	1680.9346	-0.1277	-76	122	136	LLLAISEDVRVLLVK	
1703.8026	1703.9053	0.1027	60	141	154	LHNMRTLGVMCEDK	Carbamidomethyl (C)[11]
1771.8723	1772.0408	0.1685	95	385	398	LELFQDQVFCFTPK	Carbamidomethyl (C)[10]
1781.7911	1781.9525	0.1614	91	663	676	WDIDDQMSERFPAR	Oxidation (M)[7]
1852.0076	1852.0562	0.0486	26	454	471	AQVPPAAWESLVATGKAR	
2038.0498	2038.1331	0.0833	41	691	709	IAQIAAANDANIHNLSMVR	Oxidation (M)[17]
2138.0361	2138.1704	0.1343	63	710	727	TAPDFTEMIIDVEVWDLK	Oxidation (M)[8]
2230.0999	2230.3069	0.207	93	345	364	GSANNPHKISTETNAYAWLR	

3	argG gene product [Brucella suis 1330]	gi|23500989|ref|NP_697116.1|	45225.6	6	11	55	96.244	.T.	AlDahouk_6214_Z19769-61_T1	584

Peptide Information

Calc. Mass	Obsrv. Mass	± da	± ppm	Start Seq.	End Seq.	Sequence	Modification
861.4611	861.5438	0.0827	96	353	360	GNVMVIGR	Oxidation (M)[4]
1074.5077	1074.593	0.0853	79	75	82	DFVFPMFR	Oxidation (M)[6]
1265.7035	1265.7332	0.0297	23	350	360	LYKGNVMVIGR	Oxidation (M)[7]
1337.6995	1337.7357	0.0362	27	302	313	GAAHLKDELMPR	
1480.7682	1480.8253	0.0571	39	132	144	FELSAYALNPDIK	
1654.8911	1654.9708	0.0797	48	277	292	GVYETPGGTILLAAHR	
1680.8591	1680.9346	0.0755	45	62	74	EIFIEDVREEFVR	
1838.9103	1839.0072	0.0969	53	113	131	TGADAIAHGATGKGNDQVR	
1851.9963	1852.0562	0.0599	32	158	173	TQLLEFAEQHQIPVAK	
2388.1611	2388.3455	0.1844	77	329	349	EMLQAAIDHSQHHVEGEVTLK	Oxidation (M)[2]
2959.4683	2959.6013	0.133	45	26	52	WLQTELGAEVVTFTADLGQGEELEPAR	

4	RelA/SpoT family protein [Brucella suis ATCC 23445]	gi|163842924|ref|YP_001627328.1|	84031.8	8.55	16	55	95.881	.T.	AlDahouk_6214_Z19769-61_T1	584

Peptide Information

Calc. Mass	Obsrv. Mass	± da	± ppm	Start Seq.	End Seq.	Sequence	Modification
1128.6624	1128.6304	-0.032	-28	122	131	LLLAISEDVR	
1319.7067	1319.7853	0.0786	60	4	13	QYELVERVQR	
1337.7535	1337.7357	-0.0178	-13	590	602	VDPAATTPKPGKR	
1348.8423	1348.7812	-0.0611	-45	508	519	DILKPGLPRLAR	
1403.7529	1403.7941	0.0412	29	14	25	YKPDVNEALLNK	
1438.7471	1438.8185	0.0714	50	485	496	QYSGLGMRILER	Oxidation (M)[7]
1465.8234	1465.835	0.0116	8	305	317	SIHTTIIGPSRQR	
1638.8784	1638.9313	0.0529	32	276	290	ALGLIHTTWSMVPGR	
1649.8203	1649.91	0.0897	54	158	172	IAEETMDIYAPLAGR	
1654.8734	1654.9708	0.0974	59	276	290	ALGLIHTTWSMVPGR	Oxidation (M)[11]
1681.0623	1680.9346	-0.1277	-76	122	136	LLLAISEDVRVLLVK	
1771.8723	1772.0408	0.1685	95	385	398	LELFQDQVFCFTPK	Carbamidomethyl (C)[10]
1781.7911	1781.9525	0.1614	91	663	676	WDIDDQMSERFPAR	Oxidation (M)[7]
1852.0076	1852.0562	0.0486	26	454	471	AQVPPAAWESLVATGKAR	
2038.0498	2038.1331	0.0833	41	691	709	IAQIAAANDANIHNLSMVR	Oxidation (M)[17]

2138.0361	2138.1704	0.1343	63	710	727	TAPDFTEMIIDVEVWDLK	Oxidation (M)[8]
2230.0999	2230.3069	0.207	93	345	364	GSANNPHKISTETNAYAWLR	

Gel Idx/Pos<br>Plate [#] Name	270/K22<br>[1] 1300017700	Instr./Gel Origin<br>Instrument Sample Name	AK043/Div_120507	Process Status<br>Spectra	Analysis Succeeded<br>1

Rank	Protein	Name	Species	Accession No.	Protein MW	Protein PI	Pep. Count	Protein Score	Protein Score C. I. %	Confirmed	Sample Name	Customer sample Name
1	rpoB gene product [Brucella suis 1330]			gi|23502120|ref|NP_698247.1|	153829.1	5.04	32	159	100	.T.	AlDahouk_6214_Z19769-62_T1	25

Protein Group

DNA-directed RNA polymerase subunit beta [Brucella suis ATCC 23445]	gi|163843508|ref|YP_001627912.1|	153857.1	5.03999996185303

Peptide Information

Calc. Mass	Obsrv. Mass	± da	± ppm	Start Seq.	End Seq.	Sequence	Modification
1081.5175	1081.5914	0.0739	68	559	567	DVHPTHYGR	
1131.547	1131.6254	0.0784	69	594	602	YGFIESPYR	
1154.5841	1154.6577	0.0736	64	370	378	QEALFDIYR	
1259.6418	1259.7257	0.0839	67	594	603	YGFIESPYRK	
1286.6812	1286.7788	0.0976	76	730	741	RGGIVDQVDATR	
1341.6104	1341.7246	0.1142	85	465	475	SVGELMENQYR	Oxidation (M)[6]
1362.6796	1362.7717	0.0921	68	1246	1256	QVTMGYIYMLK	Oxidation (M)[4]
1421.7094	1421.8073	0.0979	69	929	941	MPPGTYGTVVEVR	Oxidation (M)[1]
1435.7314	1435.849	0.1176	82	108	120	LIVFDIDEDTGAK	
1453.8022	1453.8785	0.0763	52	1257	1268	LHHLVDDKIHAR	
1476.842	1476.8505	0.0085	6	95	107	DLTYSAPLKVTLR	
1644.8955	1644.9935	0.098	60	1269	1284	SIGPYSLVTQQPLGGK	
1660.8363	1660.9497	0.1134	68	705	720	AEAPFVGTGMEPIVAR	Oxidation (M)[10]
1672.8547	1672.9459	0.0912	55	768	781	SNQSTCINQRPLVR	Carbamidomethyl (C)[6]
1711.8208	1711.9164	0.0956	56	1185	1199	SYDDDAILMLANQVK	Oxidation (M)[9]
1725.9092	1725.9952	0.086	50	20	34	IPEVAEMPNLIEVQK	Oxidation (M)[7]
1816.7847	1816.9379	0.1532	84	79	92	YEFDPPKFDVDECR	Carbamidomethyl (C)[13]
1851.9269	1852.0475	0.1206	65	1185	1200	SYDDDAILMLANQVKR	
1855.9396	1856.0403	0.1007	54	789	807	GDIIADGPSTDLGDLALGR	

1937.9901	1938.0588	0.0687	35	929	945	MPPGTYGTVVEVRVFNR	Oxidation (M)[1]
2013.9916	2014.1031	0.1115	55	189	205	GSWLDIEFDSKDIVYAR	
2030.0051	2030.1199	0.1148	57	61	78	SVFPIQDFSGASMLEFVR	
2046.0001	2046.1338	0.1337	65	61	78	SVFPIQDFSGASMLEFVR	Oxidation (M)[13]
2096.0156	2096.1489	0.1333	64	1168	1184	QTLEHIYPDNDRNEPVR	
2156.1345	2156.2432	0.1087	50	669	688	QLVSVAAALIPFLENDDANR	
2234.1775	2234.2751	0.0976	44	786	807	IHKGDIIADGPSTDLGDLALGR	
2277.1396	2277.2766	0.137	60	831	850	IVSDDVFTSIHIEEFEVAAR	
2379.2554	2379.3835	0.1281	54	873	895	NLDEAGIVYIGAEVHPGDILVGK	
2424.3066	2424.3516	0.045	19	698	720	QAVPLVRAEAPFVGTGMEPIVAR	Oxidation (M)[17]
2483.2961	2483.437	0.1409	57	568	590	ICPIETPEGPNIGLINSLATFAR	Carbamidomethyl (C)[2]
2562.2622	2562.3765	0.1143	45	1017	1037	SQWWQFAVEDEKLQGELEALR	
2719.2781	2719.4265	0.1484	55	624	647	HSVAQANVELDEQGGFVDEFVICR	Carbamidomethyl (C)[23]
2784.4414	2784.613	0.1716	62	334	358	TLIDTGETEINVLDIDHVNIGAYIR	

Analysis Information

**Report Type**	Protein-Peptide Summary by Spot	**Analysis Type**	MS
**Sample Set Name**	Div_120507_MS	**Database**	Bruc_suis_DEC2011
**Analysis Name**	AlDahouk_6214 Analyse 6	**Creation Date**	08/16/2012 10:49:37
**Reported By**	08/21/2012 19:15:34 - admin	**Last Modified**	08/16/2012 10:55:00

MS Acq. : Proc. Methods (Unspecified) : (Unspecified)

**Gel Idx/Pos**<br>**Plate [#] Name**	321/M23<br>[1] 1300017700	**Instr./Gel Origin**<br>**Instrument Sample Name**	AK043/Div_120507	**Process Status**<br>**Spectra**	Analysis Succeeded<br>1

Rank	Protein Name	Species	Accession No.	Protein MW	Protein PI	Pep. Count	Protein Score	Protein Score C. I. %	Confirmed	Sample Name	Customer sample Name
1	unnamed protein product [Brucella suis 1330]		gi|23500964|ref|NP_697091.1|	156480.8	5.33	23	72	99.92	.T.	AlDahouk_6214_Z19769-66_T1_ZT1	37 upper

Peptide Information

Calc. Mass	Obsrv. Mass	± da	± ppm	Start Seq.	End Seq.	Sequence	Modification
1084.5746	1084.6798	0.1052	97	593	603	ADVPQGTLAGR	
1100.6211	1100.7057	0.0846	77	408	417	ITLAVNGAWR	
1103.6056	1103.7156	0.11	100	59	68	LSVQSLTAER	
1116.6008	1116.6825	0.0817	73	204	214	VALALDTNGSR	
1152.5896	1152.702	0.1124	98	215	224	TLTGEFTLDR	
1259.6743	1259.757	0.0827	66	42	51	IENVKLDWSR	
1272.6808	1272.7875	0.1067	84	142	153	RLDGPGGNFALR	
1286.7216	1286.8232	0.1016	79	455	467	VASLAPFGALANR	
1342.7477	1342.7749	0.0272	20	36	46	EGVWLRIENVK	
1355.7642	1355.8662	0.102	75	509	522	ILDGAVNLSGALGR	
1416.719	1416.8578	0.1388	98	937	949	GTSANERLNIDAR	
1434.7852	1434.8807	0.0955	67	47	58	LDWSRTALFLGR	
1466.7961	1466.9081	0.112	76	748	762	DLLGVPVVDGNAAAR	
1525.8889	1526.0035	0.1146	75	239	252	FNGPIAVLVPPVFR	

1556.8795	1557.0059	0.1264	81	1288	1301	LDVVVSAPNQIFVR
1598.8649	1598.9907	0.1258	79	1008	1022	ITGPLTNPAANFNLR
1680.8915	1681.0212	0.1297	77	641	655	VDLGSLIDIAHDEKR
1969.1229	1969.2648	0.1419	72	1315	1332	LTGPITNIRPVGSFDLIR
1991.9668	1992.1376	0.1708	86	840	858	GDEITFGDIVVNTGDGQVR
2184.2134	2184.3064	0.093	43	455	476	VASLAPFGALANRNLAGSIDVK
2307.2568	2307.4089	0.1521	66	1073	1095	IPFSGQGLGVNVNGNVPLALANR
2319.2402	2319.4009	0.1607	69	12	35	ISISGISGVLSSEATVGSITVADR
2555.3252	2555.5063	0.1811	71	1342	1364	ITFDEGHVTLVGDLNPQLNFVAR

2	hypothetical protein BSUIS_A0052 [Brucella suis ATCC 23445]	gi|163842325|ref|YP_001626729.1|	163428.3	5.22	22	63	99.377	.T.	AlDahouk_6 214_Z19769-66_T1_ZT1 37 upper

Peptide Information

Calc. Mass	Obsrv. Mass	± da	± ppm	Start Seq.	End Seq.	Sequence	Modification
1084.5746	1084.6798	0.1052	97	657	667	ADVPQGTLAGR	
1100.6211	1100.7057	0.0846	77	472	481	ITLAVNGAWR	
1103.6056	1103.7156	0.11	100	123	132	LSVQSLTAER	
1116.6008	1116.6825	0.0817	73	268	278	VALALDTNGSR	
1152.5896	1152.702	0.1124	98	279	288	TLTGEFTLDR	
1259.6743	1259.757	0.0827	66	106	115	IENVKLDWSR	
1272.6808	1272.7875	0.1067	84	206	217	RLDGPGGNFALR	
1286.7216	1286.8232	0.1016	79	519	531	VASLAPFGALANR	
1342.7477	1342.7749	0.0272	20	100	110	EGVWLRIENVK	
1355.7642	1355.8662	0.102	75	573	586	ILDGAVNLSGALGR	
1416.719	1416.8578	0.1388	98	1001	1013	GTSANERLNIDAR	
1434.7852	1434.8807	0.0955	67	111	122	LDWSRTALFLGR	
1466.7961	1466.9081	0.112	76	812	826	DLLGVPVVDGNAAAR	
1525.8889	1526.0035	0.1146	75	303	316	FNGPIAVLVPPVFR	
1556.8795	1557.0059	0.1264	81	1352	1365	LDVVVSAPNQIFVR	
1680.8915	1681.0212	0.1297	77	705	719	VDLGSLIDIAHDEKR	
1969.1229	1969.2648	0.1419	72	1379	1396	LTGPITNIRPVGSFDLIR	
1991.9668	1992.1376	0.1708	86	904	922	GDEITFGDIVVNTGDGQVR	
2184.2134	2184.3064	0.093	43	519	540	VASLAPFGALANRNLAGSIDVK	
2319.2402	2319.4009	0.1607	69	76	99	ISISGISGVLSSEATVGSITVADR	

2555.3252	2555.5063	0.1811	71	1406	1428	ITFDEGHVTLVGDLNPQLNFVAR
2584.4365	2584.3838	-0.0527	-20	4	26	LFAIIAFFVFVVAAAVFAWPEQK
