# Supplementary material for: RegA Plays a Key Role in Oxygen-Dependent Establishment of Persistence and in Isocitrate Lyase Activity, a Critical Determinant of In vivo Brucella suis Pathogenicity
Source: Front Cell Infect Microbiol. 2017 May 18;7:186. doi: 10.3389/fcimb.2017.00186 (PMC5435760; doi:10.3389/fcimb.2017.00186)
Supplement: Supplementary file 8 [file Image1.PDF]

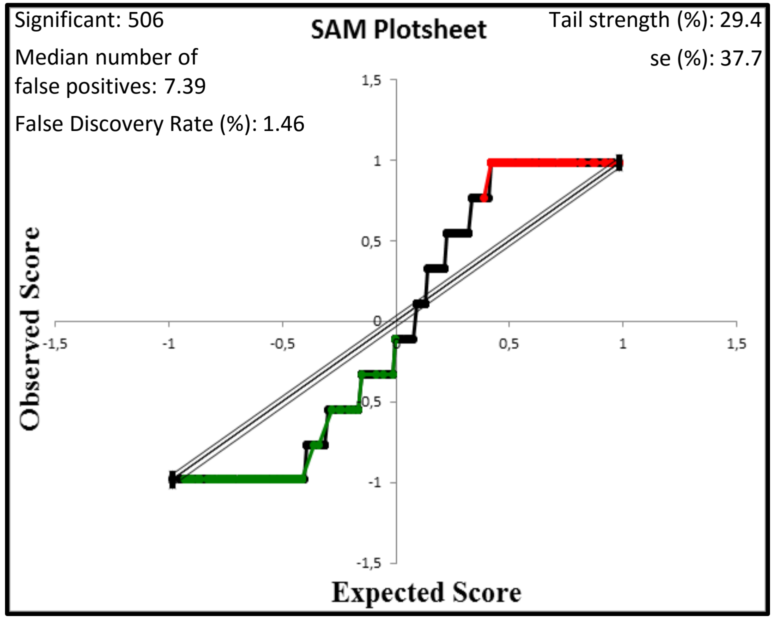

S1 Fig. Significance Analysis of Microarrays (SAM). SAM plot represents 506 genes, differentially expressed between the *B. suis* WT group and the *B. suis* Δ*regA* mutant group at a False Discovery Rate of 1,46% and with a Fold Change ≥ 2. Over-expressed genes are illustrated in red, and down-regulated genes are depicted in green.
